# Diversifying the triquinazine scaffold of a Janus kinase inhibitor

**DOI:** 10.1039/d5md00921a

**Published:** 2025-11-13

**Authors:** Kleni Mulliri, Kris Meier, Johanna-Dorothea Feuchter, Sacha Javor, Matheus A. Meirelles, Jean-Louis Reymond

**Affiliations:** a Department of Chemistry, Biochemistry and Pharmaceutical Sciences, University of Bern Freiestrasse 3 Bern CH-3012 Switzerland jean-louis.reymond@unibe.ch

## Abstract

The exploration of novel three-dimensional scaffolds remains essential for expanding chemical space and discovering new bioactive molecules. Here, we describe a robust synthetic strategy that enables modulation of Janus Kinase activity through systematic diversification of the triquinazine skeleton, a highly sp^3^-rich scaffold derived from generated databases (GDBs). By employing ring enlargement and deconstruction approaches, four unprecedented chiral scaffolds were accessed, leading to the synthesis of 26 analogues. Biological evaluation against the Janus kinase family demonstrated how subtle modifications to the triquinazine skeleton influence the activity against JAK1, JAK2, JAK3, and TYK2. Notably, compound (*S*,*R*,*R*)-40a emerged as a potent JAK1 inhibitor (IC_50_ = 18 nM), with similar potency as the FDA-approved inhibitors abrocitinib and upadacitinib. These findings highlight the potential of GDB-inspired molecules as a source for drug discovery.

## Abstract 2

Diversifying the sp^3^-rich triquinazine core, we prepared four new chiral scaffolds and 26 analogues that reveal how subtle structural changes modulate Janus Kinase activity, yielding a potent JAK1 inhibitor comparable to FDA-approved drugs.

## Graphical abstract

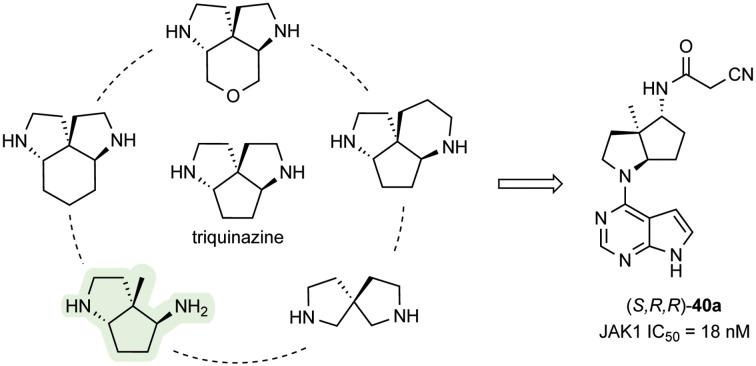

## Introduction

**Fig. 1:**
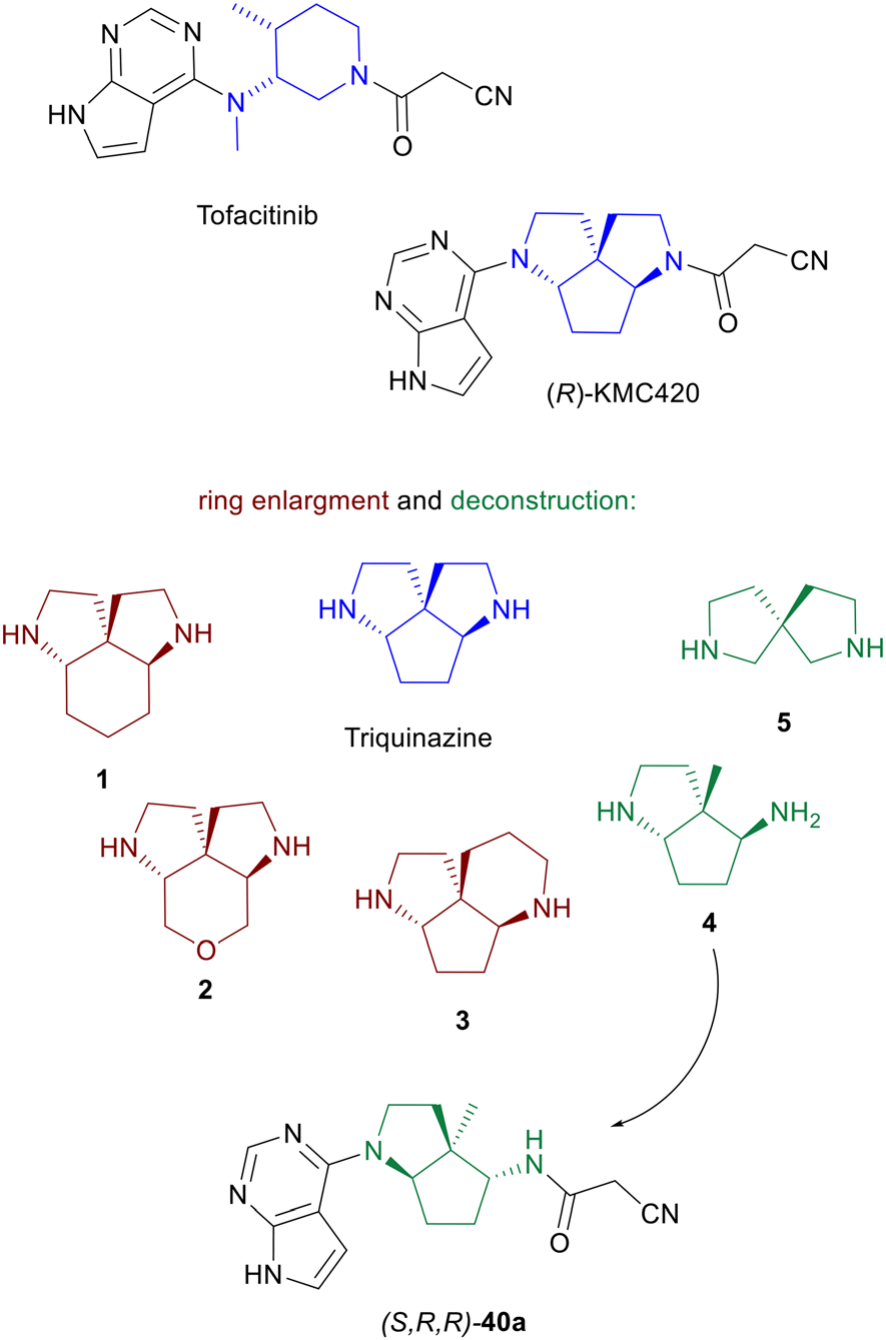
Ring enlargement and deconstruction of the triquinazine scaffold. While drawings are shown matching the stereochemistry of the parent inhibitor (*R*)-**KMC420**, the new scaffolds were synthesized and tested as racemates. The more potent enantiomer of the novel Janus kinase inhibitor 40a corresponds to (*S*)-KMC-420.

Piperazines and related analogs bearing two differentially functionalizable amine handles, related to piperazine or amino-cyclohexane, are particularly popular scaffolds for small-molecule drugs, often used as rigid bioisosteres of aromatic rings.^1–14^ We recently showed that the generated databases (GDBs),^15–17^ which enumerate all possible organic small molecules up to a certain size considering simple rules of chemical stability and synthetic feasibility, can be used to identify novel and remarkably simple piperazine analogs and related diamine scaffolds.^18,19^ One striking example was triquinazine, a typical GDB molecule featuring a 3D-shaped and intrinsically chiral framework, which we functionalized with the cyanoacetyl and 7-deazapurine groups of the immune-regulating drug tofacitinib^20^ to obtain (*R*)-**KMC420** as a nanomolar inhibitor of Janus kinases (JAKs).^21^ JAKs are a family of non-receptor tyrosine kinases implicated in the signaling of various cytokines and growth factors.^22–24^ Dysregulation of JAK activity is associated with a wide range of inflammatory and autoimmune disorders, as well as certain cancers, making JAKs attractive therapeutic targets.^25–30^ Herein, we report a synthetic study aimed at expanding the structural diversity of triquinazine by inserting or removing an atom in the central cyclopentane or in one of the pyrrolidine rings. Testing derivatives of the resulting scaffolds functionalized with 7-deazapurine and acyl groups provided a new nanomolar Janus kinase inhibitor in the form of 40a (Fig. 1).

## Results and discussion

### Design and synthesis

**Scheme 1:**
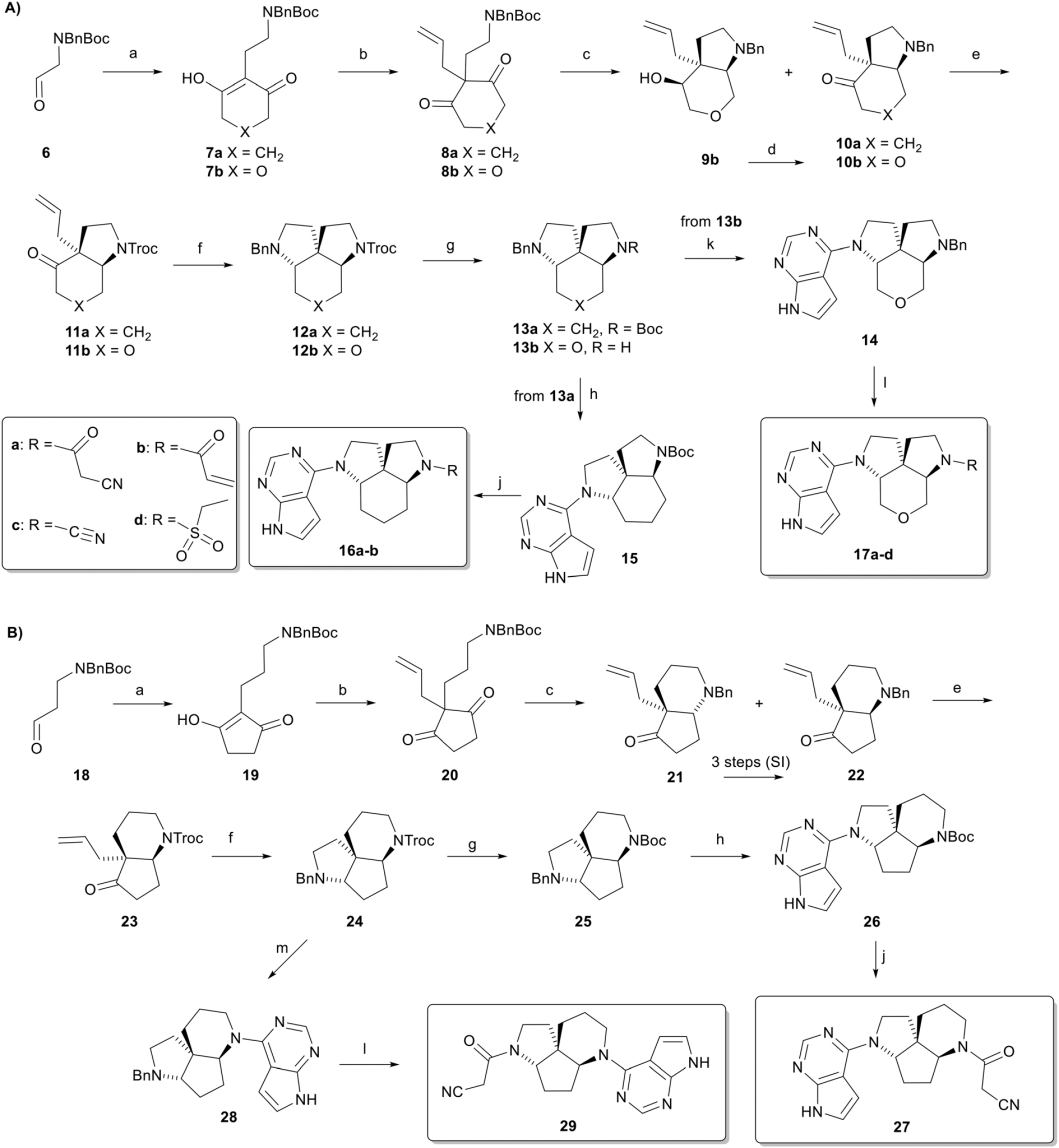
Synthesis of ring expanded triquinazine scaffolds. A. Cyclopentane expansion. B. Pyrrolidine ring expansion. Conditions: a) Diketone, Hantzsch ester, l-proline, THF, 22 °C, 16 h (7a: 89%, 7b: 49%, 19: 78%); b) allyl acetate, cat. Pd(PPh_3_)_4_, toluene, 22 °C, 48 h (8a: 84%, 8b: 89%, 20: 97%); c) i) CF_3_CO_2_H, CH_2_Cl_2_, 0–22 °C, 1 h; ii) NaBH(OAc)_3_, AcOH, CH_2_Cl_2_, 4 Å MS, 19 h, −12 °C (10a: 83%, 9b: 38% + 10b: 30%, 21: 32% + 22: 55%); d) DMP, CH_2_Cl_2_, 1 h, 0 °C, (10b: 53%); e) TrocCl, CH_3_CN, 4 Å MS, 60 °C, 16 h (11a: 77%, 11b: 88%, 23: 70%); f) i) O_3_, DCM/MeOH, 30 min, then DMS, 17 h, −78 °C to 22 °C, ii) BnNH_2_, AcOH, CH_2_Cl_2_, 4 Å MS. 22 °C, 2 h, then NaBH(OAc)_3_, 0–22 °C, 16 h (12a: 61%, 12b: 43%, 24: 72%); g) for 13a and 25: i) Zn dust, ClCH_2_CH_2_Cl/AcOH, 60 °C, 2 h, ii) CH_2_Cl_2_, Boc_2_O, Et_3_N, 2 h, 22 °C, (13a: 69%, 25: 98%); for 13b: Zn dust, ClCH_2_CH_2_Cl/AcOH, 60 °C, 2 h, 69%; h) i) MeOH, Pd/C, AcOH, H_2_, 22 °C, 1 h. ii) NMP, 6-chloro-7-deazapurine, Et_3_N, 110 °C, 18 h (15: 44%, 26: 68%). j) i) for 16a and b: CF_3_CO_2_H, CH_2_Cl_2_, 22 °C, 1 h; for 27: MeOH/HCl (1.25 M), 22 °C, 16 h; ii) for 16a and 27: 1-cyanoacetyl-3,5-dimethylpyrazole, DIPEA, MeCN, 75 °C, 4 h (16a: 12%, 27: 9%), for 16b: acrylic acid, EDC, DMAP, DIPEA, CH_2_Cl_2_, 22 °C, 16 h, 8%; k) *t*-BuOH, 6-chloro-7-deazapurine, K_2_PO_4_, 120 °C, 18 h, 88%; l) i) for 17a–d: Pd(OH)_2_, H_2_ 1 atm, THF/MeOH, 1 h, 22 °C; for 29: Pd(OH)_2_, H_2_ 10 atm, MeOH, 2 h, 22 °C; ii) for 17a and 29: 1-cyanoacetyl-3,5-dimethylpyrazole, DIPEA, MeCN, 75 °C, 4 h (17a: 66%, 29: 32%), for 17b and 17d: coupling acyl chloride, NaHCO_3_, DIPEA, CH_2_Cl_2_, 0 °C to 22 °C (17b: 18%, 17d: 26%), for 17c: cyanogen bromide, NaHCO_3_, MeOH, 0–22 °C, 56%; m) i) DCE/AcOH, Zn dust, 60 °C, 1 h, ii) NMP, 6-chloro-7-deazapurine, 110 °C, 15 h, 45%.

We first set out to adapt our synthesis of triquinazine replacing the cyclopentane-1,3-dione starting material with cyclohexane-1,3-dione to form the expanded scaffold 1 featuring a central cyclohexane (Scheme 1A). Reductive condensation with the protected aminoacetaldehyde 6 catalyzed by l-proline in the presence of Hantzsch ester to intermediate 7a followed by palladium catalyzed allylation with allyl acetate, provided diketone 8a. Boc deprotection and intramolecular reductive amination then gave aminoketone 10a with the expected *syn* ring fusion stereochemistry. The benzyl protecting group was exchanged to a trichloroethyl carbamate (Troc) to form 11a and ozonolysis followed by double reductive amination with benzylamine effected the second ring closure, which proceeded with complete *syn* stereoselectivity, to afford 12a featuring an orthogonally protected form of scaffold 1.

The Troc group, which is sensitive to debenzylation conditions, was then exchanged for Boc to form 13a. Debenzylation and arylation of the resulting secondary amine with 6-chloro-7-deazapurine followed by Boc deprotection and acylation with cyanoacetyl chloride afforded 16a as ring expanded analog of **KMC420**, as well as analog 16b upon acylation with acryloyl chloride. A similar synthesis starting with pyrane-3,5-dione afforded the corresponding protected scaffold 13b as the oxa-analog of 13a, and the corresponding versions functionalized with the above mentioned cyanoacetyl (17a) and acryl groups (17b), as well as with cyano (17c) and ethylsulfonyl (17d) groups. These functional groups have been reported as important substituents in JAK inhibitors, enhancing their potency and selectivity through polar interaction with the binding site.^27^ In some cases, the acryl group can also engage in a covalent interaction with JAK3, the only kinase in this family that contains a cysteine residue (Cys909) in the binding site.^25,31^ One striking example is ritlecitinib, a FDA-approved covalent JAK3 inhibitor used to treat severe alopecia areata.^32^

Adapting the approach to ring expansion of one of the pyrrolidines to form a piperidine ring proved more challenging due to a non-stereoselective piperidine ring closure and the formation of two regioisomeric forms (Scheme 1B). Reductive condensation of cyclopentane-1,3-dione with the protected 3-aminopropanal 18 and allylation of the resulting intermediate 19 provided diketone 20 as anticipated. However, the ensuing Boc deprotection and intramolecular reductive amination were not stereoselective and afforded the bicyclic aminoketones 21 and 22 as a mixture of diastereomers. Nevertheless, the undesired *anti*-diastereomer 21 could be converted to its *syn*-diastereomer 22 using *m*-CPBA, followed by imine formation with trifluoroacetic anhydride and reduction with the sterically hindered sodium tris(2-ethylhexanoyloxy)borohydride.^33,34^ Protecting group exchange on 22 then provided aminoketone 23, which was subjected to ozonolysis and reductive amination with benzylamine to form 24 as a protected form of the ring-expanded scaffold 3.

Further protecting group exchange as above gave the corresponding Boc-protected scaffold 25 and its functionalized version 27 with its piperidine ring bearing the cyanoacetyl group. On the other hand, Troc removal on intermediate 24 and arylation with 6-chloro-7-deazapurine afforded intermediate 28, which was hydrogenated and acylated to afford analog 29 with the piperidine ring carrying the 7-deazapurine group. In this case, we did not prepare other acyl analogs.

**Scheme 2:**
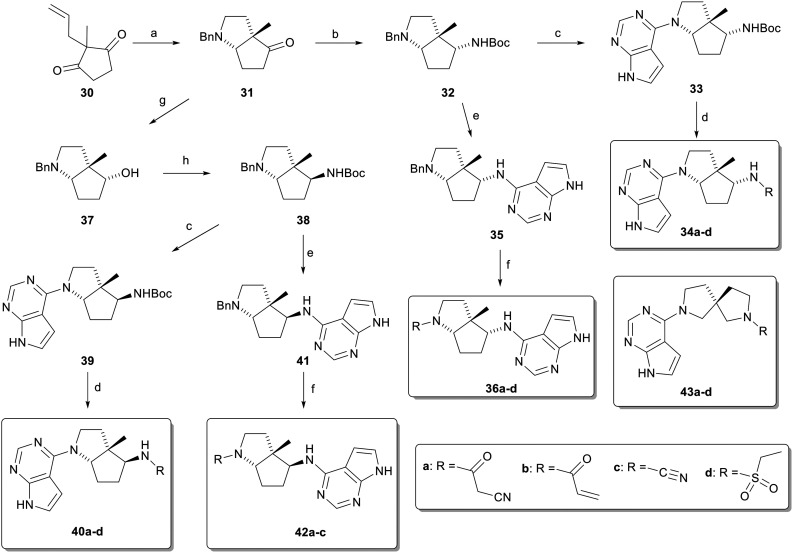
Synthesis of deconstructed triquinazine scaffolds. Conditions: a) i) O_3_, DCM/MeOH, 30 min, then DMS, 17 h, −78 °C to 22 °C, ii) BnNH_2_, AcOH, CH_2_Cl_2_, 4 Å MS, 22 °C, 1 h, then NaBH(OAc)_3_, 22 °C, 14 h, 61%; b) i) ammonium acetate, NaBH_3_CN, MeOH, 50 °C, 19 h, ii) Boc_2_O, Et_3_N, CH_2_Cl_2_, 22 °C, 1 h, 63%; c) i) cat. Pd/C, H_2_ (1 atm.), AcOH, MeOH, 22 °C, 30 min, ii) 6-chloro-7-deazapurine, K_3_PO_4_, *t*-BuOH/H_2_O, 120 °C, 18 h (33: 92%, 39: 77%); d) i) CF_3_CO_2_H, CH_2_Cl_2_, 22 °C, 1 h; ii) for 34a and 40a: 1-cyanoacetyl-3,5-dimethylpyrazole, DIPEA, MeCN, 75 °C, 4 h (34a: 95%, 40a: 69%), for 34b, 45d, 40b, 40d: coupling acyl chloride, NaHCO_3_, DIPEA, CH_2_Cl_2_, 0 °C to 22 °C (34b: 46%, 34d: 76%, 40b: 31%, 40d: 20%), for 34c, 40c: cyanogen bromide, NaHCO_3_, MeOH, 0–22 °C, (34c: 15%, 40c: 14%); e) i) DCM/TFA, 22 °C, 1 h, ii) K_3_PO_4_, *t*-BuOH/H_2_O, 6-chloro-7-deazapurine, 80 °C, 6 days (35: 76%, 2 steps), for 41: i) DCM/TFA, 22 °C, 1 h, ii) K_3_PO_4_, *t*-BuOH/H_2_O, 6-chloro-7-deazapurine, 120 °C, 72 h, 50%; f) i) Pd(OH)_2_, MeOH/THF, 22 °C, 2 h, ii) for 36a, 42a: 1-cyanoacetyl-3,5-dimethylpyrazole, DIPEA, MeCN, 75 °C, 4 h (36a: 21%, 42a: 36%), for 36d, 42b: coupling acyl chloride, NaHCO_3_, DIPEA, CH_2_Cl_2_, 0 °C to 22 °C (36b: 17%, 36d: 18%, 42b: 28%), for 36c, 42c: cyanogen bromide, NaHCO_3_, MeOH, 0–22 °C, (36c: 77%, 42c: 42%); special case 36b: i) Boc_2_O, CH_2_Cl_2_, DMAP, 22 °C, 2 h, ii) Pd(OH)_2_, MeOH/THF, 22 °C, 2 h, iii) acryloyl chloride, NaHCO_3_, DIPEA, CH_2_Cl_2_, iv) TFA, DCM, 0 °C to 22 °C, 17%; g) NaBH_4_, MeOH, 0–22 °C, 45 min, 92%; h) i) DPPA, DIAD, PPh_3_, THF, 0–22 °C, 4 h, then PPh_3_, H_2_O, 22 °C, 16 h, ii) CH_2_Cl_2_, Boc_2_O, Et_3_N, 0–22 °C, 1 h, 46%.

In a third approach, we considered the deconstruction of the triquinazine scaffold (Scheme 2). Our first deconstructed scaffold 4 featured the opening of one of the pyrrolidine rings by the removal of a methylene group. The synthesis started with cyclopentane-1,3-dione 30 obtained by palladium-catalyzed allylation of 2-methyl-cyclopentane-1,3-dione with allyl acetate. Ozonolysis and double reductive amination with benzylamine, as above, provided aminoketone 31 stereoselectively. Reductive amination with ammonium acetate and Boc protection afforded the orthogonally protected diamine 32, resulting from stereoselective reduction of the iminium intermediate from the *exo*-face of the bicyclic system. Although this stereochemistry did not match the targeted scaffold 4, we proceeded with functionalization as described for scaffold 2 to form the two regioisomeric products, each with the four different acyl groups as 34a–d and 36a–d.

On the other hand, the desired scaffold 4 stereochemistry was accessed by first reducing aminoketone 31 with NaBH_4_, which similarly to the reductive amination, proceeded stereoselectively from the *exo*-face, to form alcohol 37. Then, a Mitsunobu reaction with diphenylphosphoryl azide followed by reduction of the azide and Boc protection proceeded with clean inversion of configuration to provide 38 as an orthogonally protected form of scaffold 4 with the correct stereochemistry, which was functionalized as above to the pair of regioisomers 40a–d and 42a–c. To address the deconstruction of the cyclopentane ring of triquinazine, we considered the removal of its cyclizing ethylene group, which results in the spirocyclic diamine 5, available commercially in the mono-Boc protected form, from which we prepared the four acyl derivatives 43a–d.

### Kinase screening and identification of 40a as a potent JAK1 inhibitor

Given the structural similarities between the expanded ring analogues 16a and 16b and the previously described inhibitor **KMC420**, we initially evaluated their activity at 0.1 μM against JAK1, JAK2, JAK3, and TYK2, using biochemical enzymatic assays provided by Eurofins Cerep SA. The incorporation of a cyclohexane ring proved detrimental to activity, as both compounds showed no inhibition at 0.1 μM across all four kinases. We therefore increased test concentrations to 1 μM when next testing the piperidine analogues 27 and 29, which displayed modest selectivity for JAK1, but overall low activity against the panel. The oxygenated cyclohexane derivatives 17a–d, designed based on synthetic accessibility, also exhibited poor inhibitory activity. These results suggest that the size of the tricyclic core is a critical factor in potency against the targeted kinases.

We next turned our attention to the deconstructed analogues, beginning with a series featuring the methyl and amine groups in *anti* configuration, and a deazapurine moiety linked to the pyrrolidine ring. Compounds 34a–d exhibited only modest JAK1 inhibition at 1 μM (<90% inhibition) and were therefore not pursued further. We then evaluated additional deconstructed analogues at 10 μM across four kinases.

Notably, the series with the *syn* configuration containing the deazapurine in the pyrrolidine ring (40a–d) showed >90% inhibition against most targets at 10 μM (Table S2, SI). Based on these results, these four analogues were selected for testing at 0.1 μM, and compound 40a displayed 80% inhibition for JAK1 (Table 1), and a good selectivity with <30% inhibition for the other three kinases.

**Table 1:** Janus kinase inhibition screening

Compound	Conc. (μM)	% of inhibition^a^			
		JAK1	JAK2	JAK3	TYK2
16a	0.1	15 ± 1	17 ± 5	3 ± 5	−6 ± 9
16b	0.1	17 ± 1	−1 ± 10	−3 ± 10	−6 ± 3
27	1	70 ± 2	29 ± 6	38 ± 5	44 ± 4
29	1	83 ± 0	25 ± 4	22 ± 3	21 ± 3
17a	10	84 ± 2	37 ± 2	52 ± 1	61 ± 0
17b	10	85 ± 3	28 ± 1	43 ± 4	57 ± 0
17c	10	74 ± 1	24 ± 3	62 ± 1	49 ± 8
17d	10	91 ± 2	63 ± 3	59 ± 3	90 ± 0
34a	1	85 ± 1	41 ± 5	43 ± 5	41 ± 6
34b	1	73 ± 1	27 ± 3	38 ± 1	23 ± 5
34c	1	89 ± 2	45 ± 8	43 ± 3	53 ± 5
34d	1	88 ± 1	61 ± 1	45 ± 6	56 ± 1
36a	10	94 ± 0	60 ± 0	54 ± 4	75 ± 2
36b	10	84 ± 2	47 ± 2	63 ± 1	52 ± 0
36c	10	92 ± 0	80 ± 3	38 ± 2	74 ± 3
36d	10	86 ± 1	51 ± 3	42 ± 3	67 ± 1
40a	**0.1**	**80 ± 3**	**3 ± 1**	**27 ± 1**	**29 ± 3**
40b	0.1	59 ± 2	8 ± 1	4 ± 1	16 ± 2
40c	0.1	70 ± 2	18 ± 6	58 ± 1	61 ± 0
40d	0.1	28 ± 6	1 ± 8	−10 ± 11	11 ± 12
42a	10	94 ± 0	65 ± 0	76 ± 1	74 ± 0
42b	10	90 ± 1	49 ± 4	64 ± 3	64 ± 1
42c	10	95 ± 1	66 ± 4	75 ± 2	91 ± 0
43a	0.1	61 ± 1	16 ± 5	−4 ± 3	14 ± 6
43c	1	93 ± 1	59 ± 4	26 ± 1	63 ± 2
43d	1	87 ± 1	54 ± 6	22 ± 1	34 ± 8

a Data obtained from a biochemical enzymatic assay performed by Eurofins Cerep SA. Each experiment was performed in duplicate; SD values represent range divided by 
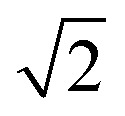
.

**Fig. 2:**
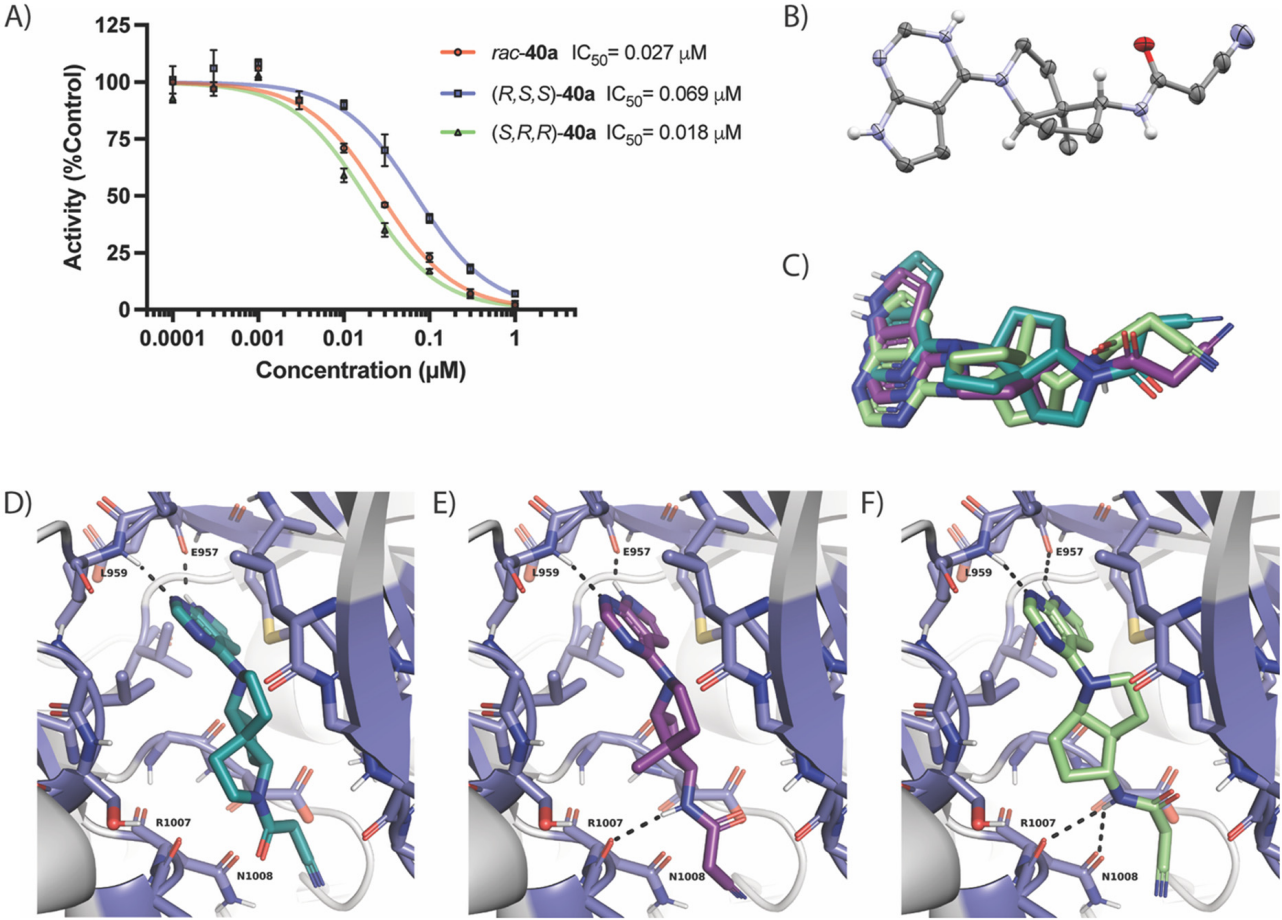
A) IC_50_ curves for *rac*-40a and its pure enantiomers; B) ellipsoid representation of X-ray structure of (*S*,*R*,*R*)-40a; C–F) docking results of (*R*)-**KMC420** (cyan blue), (*R*,*S*,*S*)-40a (purple), and (*S*,*R*,*R*)-40a (green). Protein residues relevant for inhibitor binding are shown as sticks. Panel C shows the superposition of the three compounds, and panels D–F show the individual poses and interaction of the three compounds with the JAK1.

Spiro diamines containing a deazapurine ring are known JAK inhibitors.^35^ Noji and co-workers showed that 43a has an IC_50_ of 0.64 μM against JAK3. However, this compound was not tested against the other JAK enzymes. Due to its similarity to our potent compound 40a, we decided to investigate its inhibitory activity against the four enzymes at a concentration of 0.1 μM. Our screening indicated that 43a inhibited JAK1 but did not outperform 40a, and the same trend was observed for the other spiro analogues containing the nitrile (43c) and the ethylsulfonyl group (43d). Compound 43b was found to be unstable and, therefore, was not tested. Compound 40a positive screening results led us to perform the IC_50_ determination, which confirmed *rac*-40a as a potent JAK1 inhibitor, with a value of 27 nM (Fig. 2A). Given this promising activity, *rac*-40a was subjected to enantiomeric separation by chiral HPLC, and the assignment of the absolute configurations of the enantiomers was achieved by X-ray crystallography of the hydrochloride salt of the most potent enantiomer (Fig. 2B). The enantiomer (*S*,*R*,*R*)-40a exhibited an IC_50_ of 18 nM, approximately four times more potent than (*R*,*S*,*S*)-40a, which has an IC_50_ of 69 nM. Surprisingly, the most potent enantiomer exhibited the opposite absolute configuration to the tricyclic (*R*)-**KMC420**. To investigate the binding differences between 40a and (*R*)-**KMC420**, we performed docking studies with DOCK6 using the JAK1 co-crystal structure with tofacitinib (PDB 3EYG) as receptor.

Both enantiomers, (*S*,*R*,*R*)-40a and (*R*,*S*,*S*)-40a, docked in a manner closely resembling (*R*)-**KMC420**. Their good structural superposition suggested a shared binding mode (Fig. 2C). In all three cases, the deazapurine ring formed hydrogen bonds with the hinge region residues Glu-957 and Leu-959 (Fig. 2D–F). However, docking revealed distinct orientations of the cyanoacetyl group. In the less potent enantiomer, the NH interacted with Arg-1007, whereas with (*S*,*R*,*R*)-40a, the cyanoacetyl group was rotated by ∼65°, enabling the NH group to form a new interaction with Asn-1008. This difference was consistent with the superior docking electrostatic score (Table S3, SI) of (*S*,*R*,*R*)-40a, supporting its higher potency.

The most potent enantiomer (*S*,*R*,*R*)-40a has similar potency to abrocitinib (IC_50_ = 29 nM) and upadacitinib (IC_50_ = 47 nM),^30^ two clinically used JAK1 inhibitors. These results underscore the use of GDBs as a powerful source for scaffold diversification in drug discovery.

## Conclusions

In summary, we developed a robust synthetic route that allowed the diversification of the triquinazine skeleton. The ozonolysis reaction, followed by intramolecular reductive amination, proved highly efficient for accessing four novel complex chiral scaffolds containing high sp3 content, a feature characteristic of GDB compounds. In total, 26 analogues were synthesized and evaluated against JAK1, JAK2, JAK3, and TYK2. Although expansion of the trycliclic core generally reduced the activity against the tested panel, removing an atom of one of the pyrrolidine rings resulted in potent JAK1 inhibitors. The stereochemistry of the cyclopentanamine ring in the deconstructed analogues had a critical effect on activity, where the relative *syn* configuration between the amine and the methyl groups was more active than their *anti* configuration. Furthermore, the position of the deazapurine moiety was essential for the activity, with attachment to the pyrrolidine ring yielding the most potent analogues. The cyclopentane ring was also crucial for high activity, since its removal, represented by the spiro compounds, resulted in weaker inhibitors. The combination of these structural features led to the discovery of (*S*,*R*,*R*)-40a, a potent JAK1 inhibitor (IC_50_ = 18 nM), providing more insights into how triquinazine skeleton diversification influences the Janus kinase activity. These findings highlight how minor stereochemical and topological modifications within the diamine framework can modulate biological activity, underscoring the intrinsic value of GDB as a source of diverse three-dimensional scaffolds for drug discovery.

## Author contributions

The project was conceived and designed by KlM and JLR, and supervised by KrM, MM, and JLR. KlM and JDF performed chemical synthesis. SJ performed docking studies. MM and JLR wrote the paper with the input of all authors.

## Conflicts of interest

There are no conflicts to declare.

## Supplementary Material

MD-017-D5MD00921A-s001

MD-017-D5MD00921A-s002

## Data Availability

The data supporting this article have been included as part of the supplementary information (SI). Supplementary information: synthetic procedures and characterization of all compounds, X-ray crystal data, chiral separation, biochemical assays, docking score results, images of NMR spectra. See DOI: https://doi.org/10.1039/d5md00921a. CCDC 2482160–2482168 contain the supplementary crystallographic data for this paper.^36*a*–*i*^
