# Supplementary material for: Diversifying the triquinazine scaffold of a Janus kinase inhibitor
Source: RSC Med Chem. 2025 Nov 13;17(1):310–6. doi: 10.1039/d5md00921a (PMC12679645; doi:10.1039/d5md00921a)

Supplementary Information (SI) for RSC Medicinal Chemistry.
This journal is © The Royal Society of Chemistry 2025

Diversifying the Triquinazine Scaffold of a Janus Kinase Inhibitor

Kleni Mulliri, Kris Meier, Johanna-Dorothea Feuchter, Sacha Javor, Matheus A. Meirelles, Jean-Louis Reymond*

Department of Chemistry, Biochemistry and Pharmaceutical Sciences, University of Bern, Freiestrasse 3, Bern CH-3012, Switzerland

E-mail: jean-louis.reymond@unibe.ch

Supporting Information

Table of Contents

EXPERIMENTAL SECTION **2**

GENERAL REMARKS 2
TRICYCLIC ANALOGUES 8
DECONSTRUCTED ANALOGUES 23
SPIRO ANALOGUES 37

X-RAY CRYSTAL DEPOSITION **40**

CHIRAL SEPARATION **45**

BIOCHEMICAL ASSAYS: **47**

JANUS KINASE INHIBITION COMPLEMENTARY DATA **48**

DOCKING SCORE RESULTS **48**

NMR DATA **49**

Experimental Section

General remarks

Commercially available chemicals were purchased from commercial suppliers: Sigma Aldrich, Fluorochem, Alfa Aesar, Combi-Blocks, and others. They were used without further purification unless otherwise stated. Dry solvents for reaction (DCM, THF, toluene, CH_3CN) were obtained by an in-house dry solvent system (filtration over alumina under a positive pressure of argon). Other dry solvents (DMF, pyridine) were purchased from commercial sources. Dry MeOH and EtOH were prepared by storing absolute solvents over 3 Å MS (25% m/v) for at least 5 days. Solvents used for non-anhydrous reactions, extractions, and chromatography (acetic acid, acetone, $CHCl_3$, CH_3CN, DCM, EtOAc, EtOH, heptane, *i*PrOH, MeOH, THF) were bought in technical quality and distilled before use or bought in HPLC grade from commercial suppliers. TLC was conducted on pre-coated aluminium sheets (0.2mm silica gel 60 with fluorescent indicator, ALUGRAM®Xtra SIL G/UV254 by Macherey-Nagel). Visualization was performed by either UV light (254 nm) or by staining with potassium permanganate or ninhydrin solution. Column chromatography was carried out with SiO2 (pore size: 60 Å, 230-400 mesh particle size by Sigma Aldrich) and distilled technical solvents. Medium-pressure liquid chromatography (MPLC) purifications were carried out with Teledyne Isco RediSep® Rf cartridges (4 g–220 g SiO2 as indicated, particle size 0.035–0.070 mm, 230–400 mesh) on an Interchim puriFlash®430 system with integrated UV and UV-vis detector and fraction collector. The solvent gradient is indicated individually in parentheses. NMR spectra were recorded at 22 °C unless otherwise stated. Chemical shifts (δ) are reported in ppm relative to the signal of tetramethylsilane (TMS) and residual solvent signals in ^{1}H and ^{13}C NMR spectra were used as internal reference. Coupling constants (J) are given in Hz. The apparent resonance multiplicity is described as s (singlet), d (doublet), t (triplet), q (quartet), quint. (quintet), m (multiplet), or combinations thereof and broad signals are indicated as br (broad). Atom to peak assignment was performed through standard 2D NMR techniques such as COSY, HSQC, HMBC, and NOESY. ^{1}H and ^{13}C-NMR spectra were measured either on a Bruker Avance 300 spectrometer (at 300 MHz and 75 MHz, respectively) or on a Bruker Avance II 400 spectrometer (at 400 MHz and 101 MHz, respectively). Analytical RP-HPLC was

performed with an Ultimate 3000 Rapid Separation LC-MS System (DAD-3000RS diode array detector) using an Acclaim RSLC 120 C18 column (2.2 µm, 120 Å, 3×50 mm) from Dionex. Data recording and processing were done with Dionex Chromeleon Management System Version 6.80 (analytical RP-HPLC). All RP-HPLC systems used HPLC-grade acetonitrile and Milli-Q deionized water. The elution solutions were A: MilliQ deionized water containing 0.05% TFA; D: MilliQ deionized water/acetonitrile (10:90, v/v) containing 0.05% TFA. HRMS analyses were performed on an LTQ Orbitrap XL with nano ESI (Thermo). Single crystal X-ray diffractions were carried out using Oxford Diffraction (now Agilent) SuperNova, equipped with Mo micro-source and Oxford cryosystem 700 for low/high temperature measurements.

General Procedure A. The commercially available diketone (1 equiv) was dissolved in DCM (C = 0.3 M), and Hantzsch ester (1.3 equiv) was added. The corresponding aldehyde (1.3 equiv) and L-proline (0.26 equiv) were added to the reaction mixture and stirred at 22 °C till full consumption of the starting material (16-48 h). The product was purified by silica column chromatography to give the pure title compound.

General Procedure B. Under argon atmosphere, the monoalkylated 1,3-diketon (1 equiv), was dissolved in dry THF (C = 0.5 M). Allyl acetate (1.1 equiv) and $Pd(PPh_3)_4$ (1 mol%) were added to the solution, and the reaction mixture was stirred at 22 °C till full consumption of the starting material. Removal under reduced pressure of the solvent and purification by silica column chromatography with Hept/EtOAc (8:2) afforded the product.

General Procedure C. To an ice-cold solution of the dialkylated 1,3- diketone (1 equiv) in DCM (C = 0.1M) was added trifluoroacetic acid (20%). The cooling bath was removed, and the solution was stirred for 1 h at 22 °C. The solvent and excess trifluoroacetic acid were removed under reduced pressure, co-evaporated with toluene, and dried in high vacuum. The oily residue was dissolved in anhydrous DCM (C = 0.3M), and freshly activated 4 Å molecular sieves (3.0 g), and reducing reagent (2 equiv) were added successively at (-12) °C. The reaction mixture was stirred for 19 hours at (-12) °C. After the reaction was finished, the mixture was filtered over Celite, and the solvent was evaporated to around one-fifth of the original volume. The solution was basified using 2

M NaOH solution, extracted with DCM, dried over Na_2SO_4, filtered, and the solvent was reduced to yield a yellow oil. Purification by column chromatography SiO_2: DCM, afforded the title compound.

General Procedure D. The *cis* isomer (1.0 equiv) was dissolved in dry MeCN (C = 0.5 M) under an argon atmosphere. Freshly activated molecular 4 Å molecular sieves and Troc-chloride (2 equiv) were added to the reaction and the mixture was then stirred at 60 ° for 16 h. The reaction was cooled to room temperature, filtered through Celite, and the solvent was removed under reduced pressure. Purification by silica column chromatography with heptane/ EtOAc (9:1) and 0.1% of triethylamine, afforded the pure title compound.

General Procedure E. A stream of O_3/O_2 (≈3 g O_3/h) was introduced into a solution of the Troc-protected bicyclic amine (1 equiv), in a 1:1 mixture of DCM/MeOH (C = 0.26 M), for 30 minutes, at -78 °C. After the characteristic blue color, indicating the end of the ozonolysis, the excess ozone was removed with a stream of O_2. Dimethyl sulfide (10 equiv) was added to the reaction, and the mixture was allowed to slowly warm to room temperature over 16 h. The solvent was removed under reduced pressure, and the crude was co-evaporated 3 times with toluene and dried in high vacuum. The material was dissolved in dry DCM (C = 0.26 M), MS 4 Å was added, followed by benzyl amine (1 equiv). Then, the mixture was stirred for 2 h at 22 °C. The reaction mixture was cooled to 0 °C, and AcOH (1 equiv.) was added. After portion-wise addition of $NaBH(OAc)_3$ (2 equiv.), the reaction was stirred for 16 h at 0 °C – 22 °C. The mixture was filtered through Celite, washed with MeOH, evaporated the solvent, and then dissolved in DCM followed by quenching the reaction with NaOH (2 M). Extraction with DCM afforded the crude product, which was purified by silica column chromatography.

General Procedure F. The benzyl-troc protected tricyclic diamine was dissolved in (1:1) mixture of DCE/AcOH (C = 0.1M), Zn dust (30 equiv) was added. The mixture was stirred for 2 h at 60 °C. After cooling down to room temperature, the reaction crude was filtered through Celite with MeOH, and the solvent was removed under reduced pressure. The residue was dissolved in DCM (C = 0.1M), followed by the addition of Boc-anhydride (1.5

equiv) and triethylamine (10 equiv). Stirring for 2 h at 22 °C, followed by removal of the solvent, gave the crude material, which was purified by silica column chromatography.

General Procedure G. To a solution of diamine (1 equiv) in MeOH (C = 0.1M) was added Pd/C (10 mol%) and AcOH (2 equiv). The reaction was stirred under H_2 atmosphere (1 atm, balloon) at 22 °C for 1 h. The reaction mixture was filtered over Celite, washed with MeOH, and the solvent was removed under reduced pressure. The residue was dissolved in *N*-methyl-pyrrolidone (0.5M), and then 6-chloro-7-deazapurine (1.1 equiv) and triethylamine (0.35 ml, 2.5 mmol, 3 equiv) were added. The reaction was then stirred at 110 °C for 18 h. After cooling down to room temperature, the mixture was poured into water and extracted three times with DCM. The solvent was evaporated, and the crude was purified by column chromatography.

General Procedure H. The Boc-protected amine (1 equiv) was dissolved in DCM, and then 20% of TFA was added dropwise at 0 °C. The reaction was stirred at 22 °C for 1-2 hours, and the solvent was evaporated under reduced pressure and dried in high vacuum. Half of the residue (1 equiv) was suspended in DCM, and *N*,*N*-diisopropylethylamine (2.0 equiv) was added to form a clear solution. Then cyanoacetic acid (1.0 equiv), *N*,*N*-diisopropylcarbodiimide (1.0 equiv), and DMAP (0.3 equiv) were added subsequently, and the reaction was first sonicated for 5 minutes, then stirred at 22 °C for 18-20 hours. The organic phase was basified with NaOH (2 M) and extracted with EtOAc (3x10 mL). The united organic phases were dried over Na2SO4, filtered, and then reduced in vacuo. The product was purified by RP-HPLC (gradient: 0–20% D in 40 min).

General Procedure J. In a solution of diamine (1 equiv), in 5 ml of DCE/AcOH (1:1) was added Zn (dust) (30 equiv). The reaction was stirred at 60 °C for 1 h. The reaction mixture was cooled down to room temperature and it was then filtered out through celite, co-evaporated with toluene, and dried in high vacuum. The residue was then dissolved in NMP (C = 0.1 M), where was added 6-chloro-7-deazapurine (1.1 equiv) and Et_3N (3 equiv). The reaction was stirred at 110 °C for 15 h. After cooling down to room temperature, the reaction mixture was poured into water and extracted three times with

DCM. The combined organic layers were dried over Na_2SO_4, filtered, and evaporated. The crude was purified by column chromatography.

General Procedure K. The benzyl-protected compound (1 equiv) was dissolved in MeOH (C = 0.01M). AcOH (6 equiv), and $Pd(OH)_2$ (1.5 equiv) were added, and the reaction was stirred at 22 °C for 2 h under H_2 (10 bar). The reaction was filtered over celite, washed with MeOH, and the solvent was evaporated under reduced pressure. The residue was then dissolved in MeCN (C = 0.5M), and DIPEA (4 equiv) and 1-cyanoacetyl-3,5-dimethylpyrazole (1.5 equiv) were added. The reaction was stirred at 75 °C for 4 hours. After cooling down to room temperature, the mixture was dissolved in DCM, washed with saturated $NaHCO_3$, and extracted 3 times with DCM. The combined organic layers were dried over Na_2SO_4, filtered, and the solvent was removed under reduced pressure. The crude was purified by column chromatography.

General Procedure L. To a solution of the benzyl-protected bicyclic diamine (1 equiv), in MeOH (C = 0.16M), was added Pd/C (10 wt%) and AcOH (0.138 ml, 2.42 mmol, 2 equiv). The reaction was stirred at 22 °C under hydrogen atmosphere (balloon, 1 atm) for 1 h. The material was filtered over Celite, washed with MeOH, and the solvent was removed under reduced pressure. The residue was transferred to a sealed tube with *t*-BuOH (3 ml), followed by the addition of 6-chloro-7-deazapurine (1.1 equiv). K_3PO_4 (5 equiv) was dissolved in 5 mL of deionized water and transferred to the sealed tube. The reaction was stirred at 120 °C for 18 h, cooled down to room temperature, deionized water was added, and the material was extracted 3 times with DCM. The organic layers were combined, dried over Na_2SO_4, filtered, and the solvent was removed under reduced pressure. Purification by column chromatography with DCM / MeOH and 0.1% NH_3 yielded the desired compound.

General Procedure M. The Boc-protected diamine (1 equiv.) was dissolved in DCM. The solution was cooled down to 0 °C, and then 30% (v/v) of TFA was added. The reaction was stirred at room temperature for 1 h, and then the solvent was evaporated and dried in high vacuum. The residue was dissolved in DCM (unless stated) under argon and cooled to 0 °C. Acryloyl chloride (1 equiv), was added followed by $NaHCO_3$ (4 equiv.) and DIPEA (2 equiv). The reaction was slowly warmed to room temperature over 16 h.

Saturated $NaHCO_3$ was added, and the mixture was extracted 3 times with DCM. The combined organic layers were dried over sodium sulfate, filtered out, and the solvent was removed under reduced pressure. Purification by column chromatography using DCM /MeOH and 0.1% NH_3, yielded the desired compound.

General Procedure N. The Boc-protected diamine (1 equiv.) was dissolved in DCM. The solution was cooled down to 0 °C, and then 30% (v/v) of TFA was added. The reaction was stirred at room temperature for 1 hour and then the solvent was evaporated and dried in high vacuum. The residue was dissolved in DCM (unless stated) under argon, and cooled to 0 °C. Ethanesulfonyl chloride (1 equiv), $NaHCO_3$ (5 equiv) and DIPEA (2-6 equiv) were added. The reaction was slowly warmed to room temperature over 16 h. Saturated $NaHCO_3$ was added, and the mixture was extracted 3 times with DCM. The combined organic layers were dried over Na_2SO_4, filtered, and the solvent was removed under reduced pressure. The crude was purified by column chromatography.

General Procedure O. The Boc-protected diamine (1 equiv.) was dissolved in DCM. The solution was cooled down to 0 °C, and then 30% (v/v) of TFA was added. The reaction was stirred at room temperature for 1 hour, and then the solvent was evaporated and dried in high vacuum. The residue was dissolved in MeOH or DCM under argon, cooled to 0 °C, followed by the addition of $NaHCO_3$ (2 equiv) and cyanogen bromide (1 equiv). The reaction was slowly warmed to room temperature over 16 h. Then, the mixture was poured into water and extracted three times with DCM. The combined organic layers were dried over sodium sulfate, filtered, and the solvent was removed under reduced pressure.

General Procedure P. The Boc-protected diamine (1 equiv.) was dissolved in DCM. The solution was cooled down to 0 °C, and then 30% (v/v) of TFA was added. The reaction was left to stir at room temperature for 1 hour, and the solvent was evaporated under reduced pressure. The residue was dissolved in acetonitrile (1 ml), and 1-cyanoacetyl-3,5-dimethylpyrazole (1.5 equiv) and DIPEA (4 equiv) were added. The reaction was stirred for 4 h at 75 °C. Saturated sodium bicarbonate was added to the mixture and then extracted with DCM. The combined organic layers were dried over sodium sulfate,

filtered, and the solvent was removed under reduced pressure. The crude was purified by column chromatography.

Tricyclic analogues

*tert-butyl benzyl(2-(2-hydroxy-6-oxocyclohex-1-en-1-yl)ethyl)carbamate (**7a**).* Synthesized according to **general procedure A**, using cyclohexane-1,3-dione (2.75 g, 24.5 mmol, 1 equiv), Hantzsch ester (8.1 g, 32 mmol, 1,3 equiv), **6** (8 g, 32 mmol, 1.3 equiv) and L-proline (988 mg, 6.37 mmol, 0.26 equiv) in THF. Purification by column chromatography with Hept/EtOAc (8: 2) to (1:1) yielded the desired compound (7.5 g, 21 mmol) in 89% yield as a yellow oil. **^{1}H-NMR** (400 MHz, CD_2Cl_2, 298 K): δ [ppm] = 7.35-7.23 (m, 5H); 4.44 (s, 2H); 3.85 (t, *J* = 7.66, 2H); 2.49 (t, *J* = 7.66, 2H); 2.42 (m, 2H); 2.23 (m, 2H); 1.89-1.86 (m, 2H); 1.48 (s, 9H). **^{13}C-NMR** (100 MHz, CD_2Cl_2, 298 K): δ [ppm] = 198.0; 174.7; 157.7; 138.6; 129.4; 127.8; 127.7; 112.7; 81.7; 52.7; 48.0; 36.7; 29.5; 28.5; 21.1.

*tert-butyl benzyl(2-(5-hydroxy-3-oxo-3,6-dihydro-2H-pyran-4-yl)ethyl)carbamate (**7b**).* Synthesized according to **general procedure A**, using 2-pyran-3,5-dione (2.41 g, 21.2 mmol, 1 equiv) Hantzsch ester (6.98 g, 27.5 mmol, 1,2 equiv), **6** (6.9 g, 27.6 mmol, 1.3 equiv) and L-proline (634 mg, 5.5 mmol, 0.26 equiv) in THF. Purification by column chromatography with Hept/EtOAc (1:1) and then with DCM/MeOH afforded the desired compound in 49% yield (4.7 g, 13.5 mmol) as a sticky oil. **^{1}H-NMR** (400 MHz, CD_2Cl_2, 298 K): δ [ppm] = 7.36-7-23 (m, 5H); 4.46 (s, 2H); 4.13 (s, 4H); 3.15-3.11 (m, 2H); 2.52-2.49 (m, 2H); 1.49 (s, 9H). **^{13}C-NMR** (100 MHz, CD_2Cl_2, 298 K): δ [ppm] = 193.9; 172.3; 157.9; 138.3; 128.9; 127.8 110.7 82.2; 71.5; 66.4; 52.8; 47.8; 28.5; 20.3. **HRMS** (ESI): (m/z) = calculated for $C_{19}H_{26}NO_5^+$ $[M+H]^+$: 348.1805 found: 348.1805.

*tert-butyl (2-(1-allyl-2,6-dioxocyclohexyl)ethyl)(benzyl)carbamate (**8a**).* Synthesized following the **general procedure B** using **7a** (3 g, 8.68 mmol, 1 equiv), allyl acetate (1.03 ml, 9.5 mmol, 1.1equiv) and palladiumtetrakis (100 mg, 0.086 mmol, 1 mol%) in dry toluene for 48h. Purification by silica column chromatography with Hept/EtOAc (8: 2) furnished the desire compound **8a** (2.8 g, 7.2 mmol) in 84% yield. **^{1}H-NMR** (400 MHz, CD_2Cl_2, 298 K): δ [ppm] = 7.35-7.20 (m, 5H), 5.61-5.50 (m, 1H), 5.06-5.00 (m, 2H), 4.33

(s, 2H), 3.01 (br, 2H), 2.56 (br, 4H), 2.42 (d, J = 7.30, 2H),1.95 (br, 4H), 1.42 (br, 9H). ^{13}**C-NMR** (100 MHz, CD_2Cl_2, 298 K): δ [ppm] = 209.4, 155.8, 139.0, 132.8, 128.8, 127.9, 127.4, 119.3, 80.0, 67.5, 50.2, 42.7, 39.1, 28.5, 17.4.

*tert-butyl(2-(4-allyl-3,5-dioxotetrahydro-2H-pyran-4-yl)ethyl)(benzyl)carbamate (**8b**).* Synthesized following the **general procedure B** using **7b** (1 g, 2.87 mmol, 1 equiv), allyl acetate (0.34 ml, 3.1 mmol, 1.1 equiv), and palladium triphenyl phosphine (33 mg, 0.028 mmol, 0.01 equiv) in dry toluene for 48 h. Purification by silica column chromatography with Hept/EtOAc (8:2) furnished the **8b** (993 mg, 2.6 mmol) in 89% yield. ^{1}**H-NMR** (400 MHz, MeOD-d4, 298 K): δ [ppm] = 7.33-7.21 (m, 5H); 5.68-5.58 (m, 1H); 5.09-5.05 (m, 2H); 4.36-4.32 (m, 6H); 3.15-2.91 (m, 2H); 2.60 (d, *J* = 2.69, 1H); 2.07-2.04 (m, 2H); 1.50-1.45 (m, 9H). ^{13}**C-NMR** (100 MHz, MeOD-d4, 298 K): δ [ppm] = 208.2; 157.5; 139.5; 132.9; 129.5; 128.3; 120.0; 74.2; 66.8; 51.0; 43.3; 28.7. **HRMS (ESI)**: (m/z) = calculated for $C_{22}H_{30}NO_5^+$ $[M+H]^+$: 388.2118 found: 288.2118.

(3aS,7aS*)-3a-allyl-1-benzyloctahydro-4H-indol-4-one (**10a**).* The title compound was prepared according to the **General Procedure C** using **8a** (2.23 g, 5.8 mmol, 1 equiv), DCM (50 mL), trifluoroacetic acid (20 mL). Then, 4 Å molecular sieves (4.0 g), and $NaBH(AcO)_3$ (2.5 g, 11.6 mmol, 2 equiv). Column chromatography SiO_2: Hept/EtOAc (8:2) gave the desired ketone **10a** (1.27g mg, 4.71 mmol) in 83% yield as an oil. ^{1}**H-NMR** (400 MHz, CD_2Cl_2, 298 K): δ [ppm] = 7.30-7.19 (m, 5H); 5.69-5.58 (m, 1H); 5.06-4.99 (m, 2H); 3.99 (d, *J* = 13.19, 1H); 3.84 (d, *J* = 13.18, 1H); 2.78 (s, *J* = 4.60, 1H); 2.64 (m, 1H); 2.53 2.39 (m, 3H); 2.35-2.28 (m, 2H); 2.20-2.05 (m, 2H); 1.99-1.92 (m, 2H); 1.82-1.75 (m, 1H); 1.39-1.32 (m, 1H). ^{13}**C-NMR** (100 MHz, CD_2Cl_2, 298 K): δ [ppm] = 212.3; 139.8; 134.2; 128.8; 128.5; 127.0; 117.5; 70.1; 58.9; 57.4; 50.8; 40.5; 39.2; 29.0; 23.7; 21.4.

(3aS,7aR*)-3a-allyl-1-benzylhexahydropyrano[3,4-b]pyrrol-4(5H)-one (**10b**).* The title compound was prepared according to the **General Procedure C** using **8b** (1,76 g, 4.5 mmol, 1 equiv), DCM (50 mL), trifluoroacetic acid (20 mL). Then, 4 Å molecular sieves (4.0 g), and NaBH(AcO)3 (1.9 g, 9 mmol, 2.0 equiv). Column chromatography SiO_2: Hept/EtOAc (1:1) gave the desired ketone **10b** (365 mg, 1.34 mmol) in 30% yield, and the corresponding alcohol **9b** (471 mg, 1.73 mmol) in 38% yield.

9b: **^{1}H-NMR** (400 MHz, CD_2Cl_2, 298 K): δ [ppm] = 7.31-7.22 (m, 5H); 5-73-5.62 (m, 1H); 5-10-5-05 (H-C(16)), 4.83 (q, *J* = 6.68, 2H); 3.87 (d, *J* = 13.08, 1H); 3.81 (dd, *J* = 5.6, 1H); 3.65 (dd, *J* = 5.77, 1H); 3.41 (d, *J* = 13.09, 1H); 2.91-2.82 (m, 2H); 2.56-2.51 (m, 1H); 2.39-2.29 (m, 2H); 2.24-2.16 (m, 1H); 1.68-1-63 (m 1H). **^{13}C-NMR** (100 MHz, CD_2Cl_2, 298K): δ [ppm] = 212.2; 139.3; 133.8; 129.1; 128.6; 127.4; 118.7; 73.8; 68.9; 67.5; 58.8; 56.6; 51.9; 41.9;32.4. **HRMS (ESI)**: (m/z) = calculated for $C_{17}H_{24}NO_2^+$ [M+H]$^+$: 274.1802 found: 274.1798.

10b: **^{1}H-NMR** (400 MHz, CD_2Cl_2, 298 K): δ [ppm] = 7.33-7.20 (m, 5H; (H-C(arom)), 5.98-5.87 (m, 1H); 5.16-5.09 (m, 2H); 3.93 (d, *J* = 13.38, 1H); 3.84 (q, *J* = 3.92, 1H); 3.74-3.68 (m, 2H); 3.59 (dd, *J* = 5.33 1H); 3.50-3.45 (m, 1H); 3.31 (d, *J* = 13.38, 1H); 3.01-2.96 (m, 1H); 2.53-2.48 (m, 1H);, 2.44-2.35 (m, 2H); 2.29-2.24 (m, 1H); 1.84-1.72 (m, 2H). **^{13}C-NMR** (100 MHz, CD_2Cl_2, 298 K): δ [ppm] = 140.3; 135.7; 128.8; 128.4; 127.0; 117.9; 70.6; 68.3; 66.2; 64.4; 58.3; 51.5; 46.9; 35.9; 30.5. **LCMS (ESI)**: (m/z) = calculated for $C_{17}H_{22}NO_2^+$ [M+H]$^+$: 272.16, found: 217.18.

Oxidation of **9b** to **10b** (Scheme 1, reaction d): Intermediate **9b** (2.24g, 8.2 mmol) was dissolved in DCM (0.1 M, 80 ml) and cool down to 0 °C. DMP (3.47 g, 8.2 mmol, 1 equiv) was added portion-wise to the reaction solution, and it was stirred at 22 °C for 1h. A 1:1 $NaHCO_3$: Na_2S_2O solution was added to the solution and then extracted three times with DCM. The combined organic layers were dried over sodium sulfate, filtered, and the solvent was removed under reduced pressure. Purification by column chromatography using Heptane /EtOAc (9:1) furnished intermediate **10b** (4.35 mmol, 1.18g) in 53% yield.

2,2,2-trichloroethyl (3aS,7aS*)-3a-allyl-4-oxooctahydro-1H-indole-1-carboxylate **(11a)***. Synthesized according to **general procedure D** using **10a** (1.19 g, 4.4 mmol, 1 equiv), Troc-Cl (0.91 ml, 6.62 mmol, 1.5 equiv), and 1 g of freshly activated molecular sieves 4Å. Purification by column chromatography with Hept/EtOAc (8: 2) yielded **11a** (1.2 g, 3.3 mmol) in 77% yield as a yellow oil. **^{1}H-NMR** (400 MHz, CD_2Cl_2, 298 K): δ [ppm] = 5.72-5.61 (m, 1H); 5.10-5.03 (m, 2H); 4.83-4.68(m, 2H); 4.08-4.01 (m, 1H); 3.63-3.51 (1H); 3.49-3.40 (m, 2H); 2.59 (m, 1H); 2.39-2.35 (m, 3H) 2.27-2.24 (m, 2H); 2.18-2.13 (m, 1H); 2.00-1.78 (m, 2H); 1.75-1.65 (m, 3H). **^{13}C-NMR** (100 MHz, CD_2Cl_2, 298 K): δ [ppm] =

211.7-211.4; 153.0; 134.0-133.8; 119.0-118.9; 96.4-96.2; 75.0-74.9; 63.5; 62.6; 58.5-58.0; 44.9; 39.3-38.6; 32.7; 28.2; 26.5; 20.8-20.7. **HRMS (ESI)**: (m/z) = calculated for $C_{14}H_{19}Cl_3NO_3^+$ $[M+H]^+$:354.0425 found: 354.0425.

2,2,2-trichloroethyl (3aS,7aR*)-3a-allyl-4-oxohexahydropyrano[3,4-b]pyrrole-1(2H)-carboxylate (**11b**).* Synthesized according to **general procedure D** using **10b** (2.07 g, 7.63 mmol, 1 equiv), Troc-Cl (1.58 ml, 11.4 mmol, 1.5 equiv), and 1 g of freshly activated molecular sieves 4Å in 25 ml of anhydrous MeCN. Purification by column chromatography with Hept/EtOAc (2: 8) yielded **11b** (2.4 g, 6.74 mmol) in 88% yield as yellow oil. **^{1}H-NMR** (400 MHz, CD_2Cl_2, 298 K): δ [ppm] = 5.77-5.66 (m, 1H); 5.14-5.09 (m, 2H); 4.84-4.70 (m, 2H); 4.22-3.78 (m, 5H); 3.67-3.48 (m, 2H); 2.65-2.57 (m, 1H); , 2.47-2.39 (m, 1H); 2.35-2.25 (m, 1H); 1.79 (m, 1H). **^{13}C-NMR** (100 MHz, CD_2Cl_2, 298 K): δ [ppm] = 209.5; 209.2; 152.7-152.3; 132.8; 119.2-119.2; 74.7; 73.5-73.3; 69.4-68.4; 61.1-60.1; 56.4-55.6; 45.2-45.0; 39.3-39.2; 33.5-32.6. **HRMS** (ESI): (m/z) = calculated for $C_{13}H_{17}Cl_3NO_4^+$ $[M+H]^+$: 356.0218 found: 356.0218.

2,2,2-trichloroethyl(3aR,6aS*,9aS*)-6-benzyldecahydro-3H-pyrrolo[3,2-d]indole-1-carboxylate (**12a**).* Synthesized according to **general procedure E** using **11a** (1.17 g, 3.29 mmol, 1 equiv), O_3 (flow = 60 L/h), DMS (5.32 ml, 33 mmol, 10 equiv), benzyl amine (0.72 ml, 6.6 mmol, 2 equiv), 3 g of freshly dried molecular sieves, AcOH (188 µl, 3.3 mmol, 1 equiv) and $NaBH(AcO)_3$ (1.4 g, 6.58 mmol, 2 equiv). Purification by column chromatography with EtOAc/Hept (2:8) yielded the orthogonal protected tricyclic diamine **12a** (868 mg, 2 mmol) in 61% yield as a yellow oil. **^{1}H-NMR** (400 MHz, CD_2Cl_2, 298 K): δ [ppm] = 7.34-7.21 (m, 5H), 5.04-5.02 (m, 0.3H), 4.91 (m, 1H), 4.77-4.60 (m, 2H), 4.07-4.02 (m, 1H), 3.69-3.65 (m, 1H), 3.60-3.51 (m, 1H), 3.49-3.38 (m, 1.5H), 3.36-3.28 (m, 1.5H), 3.11-3.07 (m, 1H), 3.03-2.96 (m, 1H), 2.54 (m, 1H), 2.27-2.06 (m, 4H), 1.92-1.88 (m, 1H), 1.80-1.65 (m, 2H), 1.61-1.47 (m, 4H), 1.44-1.35 (m, 2H), 1.33-1.26 (m, 1H), 1.23-1.08 (m, 1H). **^{13}C-NMR** (100 MHz, CD_2Cl_2, 298 K): δ [ppm] = could not be assigned because of rotamers. **HRMS (ESI)**: (m/z) = calculated for $C_{20}H_{26}Cl_3N_2O_2^+$ $[M+H]^+$: 431.1054 found: 431.1054.

2,2,2-trichloroethyl(3aR,6aRS*,9aR*)-3-benzyloctahydro-1H,7H-pyrano[3,4-b:5,4-b']dipyrrole-7-carboxylate (**12b**).* Synthesized according to **general procedure E** using

11b (2.4 g, 7.27 mmol, 1 equiv), O_3 (flow = 60 L/h), DMS (5.32 ml, 73 mmol, 10 equiv), benzyl amine (0.8 ml, 7.27 mmol, 1 equiv), 3 g of freshly dried molecular sieves, AcOH (0.42 ml, 7.27 mmol, 1 equiv) and $NaBH(AcO)_3$ (3.0 g, 14.55 mmol, 2 equiv). Purification by column chromatography with EtOAc/Hept (3: 7) yielded the orthogonal protected tricyclic diamine **12b** (1.34 g, 3.1 mmol) in 43% yield as a yellow oil. **^{1}H-NMR** (400 MHz, CD_2Cl_2, 298 K): δ [ppm] = 7.37 (m, 5H); 4.89-4.62 (m, 1.3H), 4.14-3.91 (m, 2H), 3.81-3.72 (m, 2H), 3.57-3-38 (m, 3H), 3.15-3.02 (m, 1H) 2.45-2.34 (m, 1H), 2.27-2.13 (m, 1H), 1.94-1.77 (m, 1H), 1.72-1.62 (m, 2H). **^{13}C-NMR** (100 MHz, CD_2Cl_2, 298 K): δ [ppm] = carbon could not be solved due to rotamers.

tert-butyl(3aR,6aS*,9aS*)-6-benzyldecahydro-1H-pyrrolo[3,2-d]indole-1-carboxylate (13a)*. Synthesized according to **general procedure F** using **12a** (71 mg, 0.16 mmol, 1 equiv), and Zn dust (144 mg, 4.8 mmol, 30 equiv). Then, Et_3N (0.24 ml, 1.76 mmol, 11 equiv), and Boc_2O (80 mg, 0.36 mmol, 1,1 equiv). Purification by silica gel column chromatography furnished the benzyl-boc protected diamine **13a** (41 mg, 0.11 mmol) in 70% yield. **^{1}H-NMR** (400 MHz, CD_2Cl_2, 298 K): δ [ppm] =7.34 (m, 5H), 4.07-4.02 (m, 1H), 3.59-3.55 (m, 0.5H), 3.46-3.34 (m, 1.5H), 3.30-3.22 (m, 1H), 3.11-2.95 (m, 2H), 2.50.2.47 (m, 1H), 2.25-2.03 (m, 3H), 1.91 (m, 1H), 1.77-1.65 (m, 3H), 1.59-1.50 (m, 3H), 1.43 (s, 9H). **^{13}C-NMR** (100 MHz, CD_2Cl_2, 298 K): δ [ppm] = 155.1-154.8, 140.8-140.6, 129.0, 128.7, 128.4, 127.0, 78.8, 65.8-65.5, 62.0-61.3, 58.5-58.2, 52.0-51.7, 50.4, 49.7, 44.3, 43.8, 37.1-36.8, 33.1, 32.4, 30.2, 29.6, 28.7, 24.5.

(3aR,6aR*,9aR*)-3-benzyloctahydro-1H,6H-pyrano[3,4-b:5,4-b']dipyrrole* ***(13b)***. Compound **12b** (200 mg, 0.461 mmol, 1 equiv) was dissolved in DCM:AcOH (1:1, 5mL) and heated at 60 °C. Then, Zn dust (904 mg, 13.8 mmol, 30 equiv) was added and the mixture was stirred for 1h. The reaction was cooled to room temperature, and the mixture was filtered over Celite. The solvent was evaporated, and the product was purified by silica gel column chromatography (DCM:MeOH, 95:5) to afford **13b** (82 mg, 0.32 mmol) in 69% yield. **^{1}H NMR** (400 MHz, $CDCl_3$) δ 7.31 (s, 5H), 3.89 (d, J = 13.2 Hz, 1H), 3.66 (d, J = 13.2 Hz, 1H), 3.63 – 3.49 (m, 3H), 3.36 (dd, J = 12.1, 7.2 Hz, 1H), 3.13 – 2.96 (m, 3H), 2.94 (dd, J = 4.9, 4.0 Hz, 1H), 2.63 – 2.54 (m, 2H), 1.92 (s, 3H), 1.72 – 1.52 (m, 2H). **^{13}C NMR** (101 MHz, $CDCl_3$) δ 139.6, 128.9, 128.3, 127.1, 68.2, 68.1, 63.3, 59.8, 59.7, 52.1, 49.0, 44.2, 38.9, 35.1.

(3aR,6aR*,9aR*)-3-benzyl-7-(7H-pyrrolo[2,3-d]pyrimidin-4-yl)octahydro-1H,6H-pyrano[3,4-b:5,4-b']dipyrrole (**14**)*. Compound **13b** (80 mg, 0.31 mmol, 1 equiv) was dissolved in *t*-BuOH (1 mL), followed by the addition of 6-chloro-7-deazapurine (52 mg, 0.34 mmol, 1.1 equiv) and a solution of K_2PO_4 (328 mg, 1.55 mmol, 5 equiv) in water (2 mL). The mixture was heated at 120 °C and stirred for 18h. After cooling to room temperature, the mixture was extracted with DCM (10 mL, 3x), dried over Na_2SO_4, filtered, and the solvent was removed under reduced pressure. Purification by column chromatography (DCM:MeOH, 95:5) gave **14** (102 mg, 0.272 mmol) in 88% yield. **^{1}H NMR** (300 MHz, $CDCl_3$) δ 9.74 (s, 1H), 8.29 (s, 1H), 7.41 – 7.26 (m, 5H), 7.04 (t, *J* = 2.9 Hz, 1H), 6.59 (s, 1H), 4.49 – 4.26 (m, 2H), 4.08 (d, *J* = 13.4 Hz, 1H), 4.04 – 3.78 (m, 3H), 3.57 (dd, *J* = 12.8, 3.0 Hz, 1H), 3.41 (d, *J* = 13.4 Hz, 1H), 3.26 (q, *J* = 8.4 Hz, 1H), 3.12 (td, *J* = 8.9, 4.5 Hz, 1H), 2.51 (s, 1H), 2.48 – 2.31 (m, 2H), 1.95 – 1.76 (m, 2H), 1.61 (s, 3H).

*3-((3aR,6aR,9aR)-3-(1H-pyrrolo[2,3-b]pyridin-4-yl)octahydro-1H,7H-pyrano[3,4-b:5,4-b']dipyrrol-7-yl)-3-oxopropanenitrile (**17a**)*. In a solution of benzyl-protected amine **14** (99.7 mg, 0.26 mmol, 1 equiv) was dissolved in MeOH/THF (4/2) ml. AcOH (0.1 ml, 1.59 mmol, 6 equiv) and $Pd(OH)_2$ (93 mg, 0.66 mmol, 2.5 equiv). The reaction was left to stir for 1h at room temperature under a H_2 atmosphere (1 atm). The reaction solution was filtered through Celite, and the crude was purified by column chromatography using DCM/MeOH (9:1) and 0.1% of NH_3. The deprotected amine was isolated in 95% (72 mg, 0.25 mmol). **^{1}H NMR** (300 MHz, MeOD) δ 8.08 (s, 1H), 7.10 (d, *J* = 3.6 Hz, 1H), 6.67 (d, *J* = 3.6 Hz, 1H), 4.28 (q, *J* = 4.8 Hz, 2H), 4.04 (s, 1H), 3.98 – 3.79 (m, 2H), 3.72 (dd, *J* = 12.7, 2.8 Hz, 1H), 3.26 – 3.08 (m, 2H), 3.07 – 2.99 (m, 1H), 2.98 (t, *J* = 2.3 Hz, 1H), 2.51 (dt, *J* = 12.6, 9.7 Hz, 1H), 2.03 – 1.86 (m, 2H), 1.78 (ddd, *J* = 12.9, 9.5, 7.9 Hz, 1H).

The deprotected amine (25 mg, 0.087 mmol, 1 equiv) was diluted in MeCN (2 mL) and DIPEA (0.18 ml, 1.04 mmol, 12 equiv) was then added. The reaction was sonicated till a clear solution, and 1-cyanoacetyl-3,5-dimethylpyrazole (22 mg, 0.13 mmol, 1.5 equiv) was added. The reaction was stirred at 75 °C for four hours. After cooling down at room temperature, the mixture was dissolved in DCM, washed with saturated $NaHCO_3$, and extracted 3 times with DCM. The combined organic layers were dried over Na_2SO_4, filtered, and remove the solvent under reduced pressure. Purification by column

chromatography with DCM/MeOH (98: 02) and 0.1% of NH_3 to yield the final compound in 66% (20 mg, 0.063 mmol) as a white powder. **^{1}H-NMR** (400 MHz, CD_2Cl_2, 353 K): δ [ppm] = 11.40 (s, 1H), 8.11 (s, 1H), 7.12 (s, 1H), 6.56-6.50 (br, 1H), 4.14 (m, 1H), 4.00-3.87 (m, 5H), 3.79-3.53 (m, 5H), 2.34-2.27 (m, 1H), 2.13-2.06 (m, 1H), 1.91-1.76 (m, 2H). **^{13}C-NMR** (100 MHz, CD_2Cl_2, 353 K): δ [ppm] = 162.2, 162.0, 155.3, 151.2, 150.8, 150.6, 121.2, 121.1, 115.9, 102.7, 100.6, 100.4, 65.6, 65.3, 64.4, 58.2, 57.5, 57.3, 56.2, 48.2, 47.9, 46.8, 46.2, 45.2, 44.1, 34.7, 33.2, 32.6, 25.8, 25. **HRMS (ESI)**: (m/z) = calculated for $C_{18}H_{20}N_6O_2^+$ $[M+H]^+$: 353.1721, found: 353.1721.

*1-((3aR,6aR,9aR)-3-(1H-pyrrolo[2,3-b]pyridin-4-yl)octahydro-1H,7H-pyrano[3,4-b:5,4-b']dipyrrol-7-yl)prop-2-en-1-one (**17b**).* In a solution of benzyl protected amine **14** (30 mg, 0.079 mmol) in THF/MeOH (3: 1.5), $Pd(OH)_2$ (28 mg, 0.19 mmol, 2.5 equiv) and AcOH (18 µl, 0.47 mmol, 6 equiv) was added in the reaction mixture and was stirred at 22 °C for 1 h. The reaction was filtered through célite, washed out with MeOH, evaporate the solvent under reduced pressure and dry in high vacuum. The residue was then dissolved in DCM and DIPEA (55 µl, 0.31 mmol, 4equiv), $NaHCO_3$ (33 mg, 0.39 mmol, 5 equiv) was added and the reaction was sonicated till clear solution. Acryloyl chloride (6.3 µl, 0.079 mmol, 1 equiv) was added at 0 °C and the reaction was let to run for 17 h at 0 °C-22 °C. The reaction was poured into water and quenched with sat. $NaHCO_3$. The aqueous layer was extracted with DCM and the combined organic layers were dried over sodium sulfate, filtered out and remove the solvent under reduced pressure. Purification by silica gel column chromatography with DMC/ MeOH (98:02) and 0.01% NH_3 furnished the final compound in 18% (8 mg, 0.014 mmol). **^{1}H-NMR** (400 MHz, DMSO, 298 K): δ [ppm] = 11.64 (m, 1H), 8.12-8.10 (m, 1H), 7.18-7.15 (m, 2H), 6.20-6.14 (m, 1H), 5.72-5.66 (m, 1H), 4.12-4.06 (m, 1H), 3.99-3.87 (m, 3.5H), 3.82-3.80 (m, 1H), 3.73-3.61 (m, 3H), 3.52-3.49 (m, 0.5H), 3.26-3.16 (m, 1H), 2.33-2.26 (m, 1H), 2.08 (m, 1H), 1.91-1.74 (m, 2H). **^{13}C-NMR** (100 MHz, DMSO, 298 K): δ [ppm] = 164.4, 151.2, 150.9, 129.9, 127.2, 127.0, 121.2, 121.1, 100.7, 64.7, 58.1, 57.3, 57.1, 55.7, 45.2, 33.6, 32.2. **HRMS (ESI)**: (m/z) = calculated for $C_{18}H_{22}N_5O_2^+$ $[M+H]^+$: 340.1768 found: 340.1768.

(3aR,6aR*,9aR*)-3-(1H-pyrrolo[2,3-b]pyridin-4-yl)octahydro-1H,7H-pyrano[3,4-b:5,4-b']dipyrrole-7-carbonitrile (**17c**).* In a solution of benzyl protected amine **14** (30 mg, 0.079 mmol) in THF/MeOH (3: 1.5), $Pd(OH)_2$ (28 mg, 0.19 mmol, 2.5 equiv) and AcOH (18

µl, 0.47 mmol, 6 equiv) were added in the reaction mixture and stirred at 22 °C for 1 h. The reaction was filtered through Celite, washed out with MeOH, the solve was evaporated under reduced pressure and the compound was dried in high vacuum. The residue was then dissolved in DCM and DIPEA (55 µl, 0.31 mmol, 4 equiv), $NaHCO_3$ (33 mg, 0.39 mmol, 5 equiv) was added and the reaction was sonicated until clear solution. Cyanogen bromide (8.3 mg, 0.079 mmol, 1 equiv) was added at 0 °C and the reaction was stirred for 17 h at 0 °C-22 °C. The reaction was poured into water and quenched with sat. $NaHCO_3$. The aqueous layer was extracted with DCM and the combined organic layers were dried over sodium sulfate, filtered out and remove the solvent under reduced pressure. Purification by silica gel column chromatography with DMC/ MeOH (98: 02) and 0.01% NH_3 furnished the final compound in 56% (13 mg, 0.04 mmol) as white prouder. **^{1}H-NMR** (400 MHz, DMSO, 298 K): δ [ppm] = 11.64 (s, 1H), 8.08 (s, 1H), 7.14 (m, 1H), 6.57 (s, 1H), 4.23-4.19 (m, 1H), 4.11-4.08 (m, 1H), 3.96-3.80 (m, 3H), 3.66 (dd, *J* = 5.09, 1H), 3.59-3.48 (m, 3H), 3.11 (t, *J* = 10.69, 1H), 2.47 (1H), 1.93-1.77 (m, 3H). **^{13}C-NMR** (100 MHz, DMSO, 298 K): δ [ppm] = 155.1, 151.2, 151.0, 121.2, 115.8, 102.2, 100.6, 65.5, 63.3, 59.8, 56.1, 47.7, 45.9, 34.9. **HRMS (ESI)**: (m/z) = calculated for $C_{16}H_{19}N_6O^+$ $[M+H]^+$: 311.1615 found: 311.1620.

(3aR,6aR*,9aR*)-3-(ethylsulfonyl)-7-(1H-pyrrolo[2,3-b]pyridin-4-yl)octahydro-1H,6H-pyrano[3,4-b:5,4-b']dipyrrole (**17d**)*. In a solution of benzyl protected amine **14** (30 mg, 0.079 mmol) in THF/MeOH (3: 1.5), $Pd(OH)_2$ (28 mg, 0.19 mmol, 2.5 equiv) and AcOH (18 µl, 0.47 mmol, 6 equiv) were added to the reaction mixture and were stirred at 22 °C for 1 h. The reaction was filtered through Celite, washed out with MeOH, the solvent was removed under reduced pressure, and dry in high vacuum. The residue was then dissolved in DCM and DIPEA (55 µl, 0.31 mmol, 4equiv), $NaHCO_3$ (33 mg, 0.39 mmol, 5 equiv) was added, and the reaction was sonicated till clear solution. Ethanesulfonyl chloride (7.4 µl, 0.079 mmol, 1 equiv) was added at 0 °C, and the mixture was stirred for 17 h at 0 °C-22 °C. The reaction was poured into water and quenched with sat. $NaHCO_3$. The aqueous layer was extracted with DCM, and the combined organic layers were dried over sodium sulfate, filtered out, and the solvent was removed under reduced pressure. Purification by silica gel column chromatography with DMC/ MeOH (98: 02) and 0.01% NH_3 furnished the final compound in 26% (8 mg, 0.02 mmol) as white powder. **^{1}H-NMR** (400 MHz, DMSO, 298 K): δ [ppm] = 11.64 (s, 1H), 8.09 (s, 1H), 7.15 (m, 1H), 6.56 (m, 1H),

5.76 (DCM), 4.13 (q, 1H), 4.04-3.97 (m, 2H), 3.90-3.81 (m, 2H), 3.66-3.61 (m, 2H), 3.58-3.48 (m, 2H),3.38-3.33 (m, 1H), 3.23-3.11 (m, 2H), 2.35-2.28 (m, 1H), 2.02-1.89 (m, 2H), 1.79-1.66 (m, 1H), 1.25 (t, *J* = 7.32, 3H). ^{13}**C-NMR** (100 MHz, DMSO, 298 K): δ [ppm] = 155. 2, 151.2, 150.9, 121.1, 102.6, 100.6, 66.9, 65.3, 58.5, 57.1, 54.9 (DCM), 47.6, 46.4, 42.7, 34.0, 7.5. **HRMS (ESI)**: (m/z) = calculated for $C_{17}H_{24}N_5O_3S^+$ [M+H]$^+$: 378.1594, found: 378.1594.

tert-butyl(3aS,6aS*,9aR*)-7-(7H-pyrrolo[2,3-d]pyrimidin-4-yl)decahydro-3H-pyrrolo [3,2-d]indole-3-carboxylate (**15**)*. Synthesised according to general procedure **G** using **13a** (170 mg, 0.47 mmol, 1 equiv), Pd/C (17 mg, 10 wt%), AcOH (55 µl, 0.95 mmol, 2 equiv), 6-chloro-7-deazapurine (80 mg, 0.51 mmol, 1.1 equiv), and Et_3N (196 µl, 1.41 mmol, 3 equiv). The desired product was isolated by silica column chromatography in 44% (80 mg, 0.2 mmol). ^{1}**H-NMR** (400 MHz, CD_2Cl_2, 298 K): δ [ppm] = 10.63 (s, 1H), 8.23 (m, 1H), 7.07 (m, 1H), 6.4 (m, 1H), 4.18-4.17 (m, 1H), 4.00-3.91 (m, 2H), 3.67-3.63 (m, 1H) 3.59-3.53 (m, 1H), 3.42-3.32 (m, 1.5H), 2.26-2.16 (m, 2H), 2.08 (m, 1.5H), 1.91-1.90 (br, 1H), 1.81-1.80 (m, 5H), 1.66-1.59 (m, 2H), 1.46 (s, 9H), 1.44-1.42 (m, 3H). ^{13}**C-NMR** (100 MHz, CD_2Cl_2, 298 K): δ [ppm] = 199.63, 172.55, 157.71, 138.85, 138.74, 138.13, 128.43, 127.17, 127.14, 114.45, 80.97, 60.22, 50.45, 45.82, 39.79, 36.93, 29.63, 28.07, 20.88, 19.35.

3-((3aS,6aS*,9aR*)-7-(7H-pyrrolo[2,3-d]pyrimidin-4-yl)decahydro-3H-pyrrolo[3,2-d]indol-3-yl)-3-oxopropanenitrile (**16a**)*. Compound **15** (35 mg, 0.14 mmol, 1.0 equiv) was dissolved in HCl in MeOH (1.25 M, 2.0 ml) and stirred at 22 °C for 23 hours. After the deprotection was finished, the volatiles were evaporated under reduced pressure. The dry residue was suspended in DCM (1.0mL) and N,N-diisopropylethylamine (26 µL, 0.15 mmol, 2.0 equiv.) was added to form a clear solution. Then, cyanoacetic acid (12 mg, 0.14 mmol, 1.0 equiv.), *N,N*-diisopropylcarbodiimide (DIC) (22 µL, 0.14 mmol, 1.0 equiv), and DMAP (5 mg, 0.04 mmol, 0.3 equiv) were added subsequentially and the reaction was first sonicated for 5 minutes, then stirred at 22 °C for 20 hours. The organic phase was basified with NaOH (2 M, 5mL), and extracted with DCM (3x10 mL). The united organic phases were dried over Na_2SO_4, filtered, and then reduced in vacuo. RP-HPLC (gradient: 0–20% MeCN in water, 40 min.) yielded the final compound **16a** as a TFA salt (4 mg, 0.01 mmol, 12%). ^{1}**H-NMR** (400 MHz, MeOD-d_4, 298 K): δ [ppm] = 8.26 (s,

1H), 7.39 (br,1H), 6.92 (br, 1H), 4.23 (br, 2H), 3.98 (q, J = 4.59, 1H), 3.87-3.83 (m, 2H), 3.72-3.59 (m, 2H), 3.52-3.39 (m, 1H), 2.28-2.22 (m, 0.5H), 2.21-2-10 (m, 2H), 2.08-2.02 (m , 1H), 1.99-1.93 (m, 0.5H), 1.89-1.80 (m, 2H), 1.72-1.63 (m, 1H) 1.57-1.49 (m, 2H). **^{13}C-NMR** (100 MHz, MeOD-d_4, 298 K): δ [ppm] = 164.2, 143.4, 125.3, 115.8, 105.5, 63.6, 62.0, 47.1, 45.7, 35.1, 28.3, 26.6, 25.2, 19.2, 19.0. **HRMS (ESI):** (m/z) = calculated for $C_{19}H_{23}N_6O^+$ [M+H]$^+$: 351.1928, found: 351.1928.

*1-((3aS,6aS,9aR)-7-(7H-pyrrolo[2,3-d]pyrimidin-4-yl)decahydro-3H-pyrrolo[3,2-d]indol-3-yl)prop-2-en-1-one (**16b**).* Compound **15** (35 mg, 0.14 mmol, 1.0 equiv.) was dissolved in a mixture of HCl in MeOH (1.25 M, 2.0 ml) and the solution was stirred at 22 °C for 24 hours. The volatiles were evaporated under reduced pressure and the dry residue was suspended in DCM (1.0 ml). DIPEA (26 µL, 0.14 mmol, 1.0 equiv.) was added to form a clear solution, then acrylic acid (21 mg, 0.29 mmol, 2 equiv), EDCl (85 mg, 0.44 mmol, 2.3 equiv.), and DMAP (3.7 mg, 0.03 mmol, 0.2 equiv.) were added sequentially and the reaction was stirred at 22 °C for 20 hours. The organic phase was diluted with DCM (5 mL), washed with sat. Na_2CO_3 (3x 5 mL). and aqueous phase was then extracted with DCM (3x 5 mL). The united organic phases were dried over Na_2SO_4, filtered and the reduced. RP-HPLC (gradient: 0—50% D in 40 min) yielded the final compound **16b** as a TFA salt (2.5 mg, 0.007 mmol, 8%). **^{1}H-NMR** (400 MHz, MeOD-d_4, 298 K): δ [ppm] = 8.27 (br, 1H), 7.40 (s, 1H), 6.93 (br, 1H), 6.71-6.59, (m, 1H), 6.35-6.26 (m, 1H), 5.77 (br, 1H), 4.06-4.04 (br, 2H), 3.94-3-66 (m, 3H), 3.24-3-14 (m, 1H), 2.90-2.88 (m, 1H), 2.47-2.40 (m, 1H), 2.23-2.09 (m, 3H), 2.08-2.03 (m, 1H9, 1.91-1.83 (m, 2H), 1.68-1.66 (m, 1H), 1.53 (br, 2H). **^{13}C-NMR** (100 MHz, MeOD-d_4, 298 K): δ [ppm] = 167.7, 143.2, 130.6, 128.4, 105.5, 63.7, 61.2, 47.2, 45.6, 43.5, 35.3, 25.3, 19.2. **LCMS (ESI):** (m/z) = calculated for $C_{19}H_{24}N_5O^+$ [M+H]$^+$: 338.20, found: 338.43.

tert-butyl benzyl(3-oxopropyl)carbamate (**18**). In a solution of benzaldehyde (51 ml, 50 mmol, 1 equiv) and 3-aminopropan-1-ol (42 ml, 55 mmol, 1.1 equiv) in toluene was stir at 5 h at 150 °C using a Dean Stark. The reaction mixture was then cooled down at room temperature and the solvent was evaporated under reduced pressure. The residue was dissolved in MeOH and $NaBH_4$ was added at 0 °C and the reaction was stirred for 12 h at 0 °C. The reaction mixture was poured into water, stirred for 15 min, and extracted with DCM. The combined organic layers were dried over sodium sulfate, filtered out, and

the solvent was evaporated under reduced pressure. The benzyl-protected amine (65 g, 393 mmol, 1 equiv) was dissolved in DCM, and Boc anhydride (85 g, 393 mmol, 1 equiv) was added at 0 °C. The reaction was left to stir for 1 h at 22 °C, and the solvent was evaporated under reduced pressure. Purification by silica gel column chromatography furnished benzyl Boc-protected amine in 50% (50 g, 188.43 mmol).

The previous product (2.65 g, 10 mmol, 1 equiv) was dissolved in DCM, cooled to 0°C, DMP (4.3 g, 10 mmol, 1 equiv) was added, and the reaction was left to stir at 22 °C for 1 hour. The reaction was quenched with a 1:1 mixture of 10% $Na_2S_2O_3$ and saturated $NaHCO_3$ and was left to stir for 15 min. The mixture was extracted with DCM (3 x 50 ml), and the combined organic layer was dried over sodium sulfate, filtered out, and the solvent was evaporated under reduced pressure. Purification by silica gel column chromatography with Hept/EtOAc (8: 2), furnished the aldehyde **18** in 99% (2.6 g, 9.8 mmol). **^{1}H-NMR** (300 MHz, $CDCl_3$, 298 K): δ [ppm] = 9.75 (s, 1H), 7.38-7.24 (m, 5H), 4.47 (s, 2H), 3.53 (br, 2H), 2.66 (br, 2H), 1.49 (s, 9H). **^{13}C-NMR** (100 MHz, $CDCl_3$, 298 K): δ [ppm] = 202.0, 155.7, 128.7, 127.9, 127.5, 177.6, 80.4, 43.3, 28.5.

*tert-butyl benzyl(3-(2-hydroxy-5-oxocyclopent-1-en-1-yl)propyl)carbamate (**19**).* The title compound was prepared according to the **General Procedure A** using 1,3-cyclopentadione (2.8 g, 29 mmol, 1 equiv), Hantzsch ester (9.6 g, 38 mmol, 1.3 equiv), aldehyde **18** (10 g, 38 mmol, 1.3 equiv) and *L*-proline (869 mg, 7.57 mmol, 0.26 equiv). Purification by silica column chromatography with Hept/EtOAc from (8:2) – (1:1) gave the monoalkylated diketone in 78% yield (7.86 g, 22.7 mmol) as light-yellow coloured oil. **^{1}H-NMR** (400 MHz, CD_2Cl_2, 298 K): δ [ppm] = 7.33-7.18 (m, 5H), 5.60-5.50 (m, 1H), 5.05-4.99 (m, 2H), 4.35 (s, 2H), 3.05 (s, 1H), 2.61 (s, 4H), 2.28 (d, J = 7.45, 2H), 1.55-1.50 (m, 2H), 1.44 (s, 9H), 1.31-1.27 (m, 2H). **^{13}C-NMR** (100 MHz, CD_2Cl_2, 298 K): δ [ppm] = 216.6, 156.0, 139.1, 132.1, 128.8, 127.9, 127.4, 119.8, 79.9, 60.9, 50.3, 46.9, 39.7, 36.6, 32.0, 28.5.

*tert-butyl (3-(1-allyl-2,5-dioxocyclopentyl)propyl)(benzyl)carbamate (**20**).* The title compound was prepared according to the **General Procedure B** using **19** (15 g, 43.4 mmol, 1 equiv), allyl acetate (5.2 ml, 47.7 mmol, 1.1 equiv), and $Pd(PPh_3)_4$ (500 mg, 0.43 mmol, 0.01 equiv, 1 mol%). Purification by silica column chromatography with Hept/EtOAc (8:2) gave the final compound as an oil in 97% yield (16.2 g, 42 mmol). **^{1}H-NMR** (400 MHz, CD_2Cl_2, 298 K): δ [ppm] = 7.33-7.18 (m, 5H) 5.60-5.50 (m, 1H), 5.05-4.99

(m, 2H), 4.35 (s, 2H), 3.05 (s, 1H), 2.61 (s, 4H), 2.28 (d, J = 7.45, 2H), 1.55-1.50 (m, 2H), 1.44 (s, 9H), 1.31-1.27 (m, 2H). ^{13}**C-NMR** (100 MHz, CD_2Cl_2, 298 K): δ [ppm] = 216.6 (2C), 156.0, 139.1; 132.0; 128.8; 127.8; 127.4; 119.7; 79.9; 60.8; 50.3; 46.9; 39.7; 36.5 (2C); 32.0; 28.5.

*(cis)-4a-allyl-1-benzyloctahydro-5H-cyclopenta[b]pyridin-5-one (**22**).* The title compound was prepared according to the **General Procedure C** using **20** (2.89 g, 7.5 mmol, 1 equiv), DCM (15 mL), trifluoroacetic acid (3 mL). Then, 4 Å molecular sieves (3.0 g), and $NaBH(OEH)_3$ (3.2 g, 15 mmol, 2 equiv). The reaction gave a mixture of **22** and **21** isomers, which were separated by column chromatography SiO_2:DCM, affording the *cis* and *trans* isomers in 55% and 32%. **Note**: this reaction is performed at -12 °C to afford the *cis* isomer as the major product (Table S1, entry 3).

Table S1. Intramolecular reductive amination optimization to prepare **22**. $NaBH(OEH)_3$ was prepared in situ the day before from 2-ethylhexanoic acid and $NaBH_4$.

Entry	Temperature (°C)	Reducing agent	*Trans*-21 (%)	*Cis*-22 (%)	Overall yield (%)
1	0 - 22	$NaBH(OAc)_3$	45	38	83
2	0 - 22	$NaBH(OEH)_3$	38	36	74
3	-12	$NaBH(OEH)_3$	32	55	87
5	-12	$NaBH(OAc)_3$	22	44	66

cis-isomer (**22**): ^{1}**H-NMR** (400 MHz, CD_2Cl_2, 298 K): δ [ppm] = 7.32-7.19 (m, 5H); 5.74-5.63 (m, 1H); 5.06-5.01 (m, 2H); 3.87 (d, J = 14.06, 1H 3.36 (d, J = 14.06, 1H); 2.95 (t, J = 5.23, 1H;, 2.62-2.56 (m, 1H);, 2.52-2.42 (m, 1H; (H-C(5)), 2.29-2.09 (m, 5H; (H-C(4,5,8 and 10)), 2.00-1.90 (m, 1H); 1.75-1.69 (m, 1H); 1.56-1.46 (m, 1H); 1.37-1.23 (m, 2H); . ^{13}**C-NMR** (100 MHz, CD_2Cl_2, 298 K): δ [ppm] = 219.2; 140.1; 137.1; 128.8, (2C); 128.5 (2C); 127.1;118.1; 64.3; 59.6M; 52.6; 49.5; 38.6; 35.0; 27.4; 22.4; 19.5. **HRMS** (ESI): (m/z) = calculated for $C_{18}H_{24}NO^+$ $[M+H]^+$: 270.1852, found: 270.1852.

trans-isomer (**21**): ^{1}**H-NMR** (400 MHz, CD_2Cl_2, 298 K): δ [ppm] = 7.38-7.32 (m, 5H); 5.71-5.60 (m, 1H); 5.09-5.03 (m, 2H); 3.92 (d, J = 13.32, 1H); 3.04 (d, J = 13.32, 1H); 2.90-2.84 (m, 2H); 2.53-2.45 (m, 1H); 2.26-2.21 (m, 1H); 2.14-2.04 (m, 3H); 1.92 (t, J = 2.84, 0.5H); 1.89 (t, J = 2.77, 0.5H); 1.86-1.80 (m, 1H); 1.79-1.72 (m, 1H); 1.70-1.58 (m, 1H); 1.45-1.38 (m, 1H); 1.12-1.04 (m, 1H); . ^{13}**C-NMR** (100 MHz, CD_2Cl_2, 298 K): δ [ppm] = 217.3; 140.1;

133.7; 129.7 (2C); 128.5 (2C); 127.0; 117.5; 70.1; 58.8; 54.9; 52.2; 34.9; 30.1; 27.3; 23.3; 20.9. **HRMS** (ESI): (m/z) = calculated for $C_{18}H_{24}NO^+$ $[M+H]^+$: 270.1852, found: 270.1852.

Conversion of *trans*-isomer (**21**) to *cis*-isomer (**22**). To a solution of **21** (310 mg, 1.17 mmol, 1 equiv) in DCM (25 ml) cooled at -10 °C was added a 10 ml solution of *m*-CPBA (283 mg, 1.63 mmol, 1.4 equiv) in DCM. The reaction was left to run for 2 h at -10 °C, and anhydrous K_2CO_3 was added. The mixture was stirred for 15 min and then filtered through Celite. The solvent was evaporated under reduced pressure, and the crude was purified by silica gel column chromatography to furnish the *N*-oxide in 80% (263 mg, 1.05 mmol). To a cold solution (0 °C) of *N*-oxide (125 mg, 0.43 mmol, 1 equiv) in DCM, trifluoroacetic anhydride (TFAA) (0.12 ml, 0,86 mmol, 2 equiv) was added, and the reaction was stirred for 1 hour. $NaBH(OEH)_3$ solution was prepared from 2-Ethylhexanoic acid (0.836 ml, 5.23 mmol, 12 equiv) and $NaBH_4$ (151 mg, 1.72 mmol, 4 equiv) in DCM for 12 h. The imine reaction mixture was then cooled to -12 °C, and the $NaBH(OEH)_3$ solution was added to the mixture. The reaction was stirred at -12 °C and sat. Na_2CO_3 was added. The aqueous layer was then extracted with DCM, and the combined organic layers were dried over sodium sulfate, filtered, and the solvent was evaporated under reduced pressure. The crude was purified by silica gel column chromatography with Hept/EtOAc (9:1) and 0.1% of Et_3N to isolate the *cis*-**22** in 55% yield (64 mg, 0.23 mmol) and *trans*-**21** in 34% (40 mg, 0.14 mmol) in 89% overall yield.

2,2,2-trichloroethyl(4aR,7aS)-4a-allyl-5-oxooctahydro-1H-cyclopenta[b]pyridine-1-carboxylate (**23**). The title compound was prepared according to the **General Procedure D** using **22** (3.11 g, 11.5 mmol, 1.0 equiv) and Troc-Cl (3.69 ml, 23 mmol, 2 equiv). Purification by silica column chromatography with heptane/ EtOAc (9: 1) and 0.1% of triethylamine gave the protected compound as yellow oil in 70% yield (2.8 g, 7.93 mmol). **^{1}H-NMR** (400 MHz, $CDCl_3$, 298 K): δ [ppm] = 5.76-5.65 (m, 1H); 5.11-5.05 (m, 2H); 4.84-4.69 (m, 3H); 4.16 (d, *J* = 13.53, 1H); 2.96 (s, 1H); 2.57-2.50 (m, 1H); 2.40-2.28 (m, 2H); 2.20-2.04 (m, 3H); 1.72-1.61 (m, 2H); 1.52-1.47 (m, 2H). **^{13}C-NMR** (100 MHz, $CDCl_3$, 298 K): δ [ppm] = 217.3; 154.0; 133.1; 119.6; 75.1; 54.3; 49.1; 39.1; 36.4; 36.0; 26.7; 21.4; 19.3.

*2,2,2-trichloroethyl(5aS,9aS)-3-benzyloctahydro-1H-pyrrolo[3',2':2,3]cyclopenta [1,2-b]pyridine-6(7H)-carboxylate (**24**).* The title compound was prepared according to the **General Procedure E** using **23** (861 mg, 2.4 mmol, 1 equiv), dimethyl sulfide (1.7 ml, 24 mmol, 10 equiv.), MS 4 Å (4 g), benzyl amine (0.26 ml, 2.4 mmol, 1 equiv), AcOH (0.14 ml, 2.4 mmol, 1 equiv) and $NaBH(OAc)_3$ (1.07 g, 4.8 mmol, 2 equiv.). Purification by silica column chromatography with heptane/EtOAc (8:2) afforded **24** in 72% yield (756 mg, 1.75 mmol). **^{1}H-NMR** (400 MHz, CD_2Cl_2, 298 K): δ [ppm] = 7.29-7.21 (m, 5H); 4.80-4.71 (m, 2H); 4.48 (t, J = 9.35, 0.5H); 4.37-4.34 (m, 0.5H) 4.09-4.01 (m, 1H) 3.83 (d, J = 13.24, 1H); 3.16-3.10 (m, 1H);2.98-2.84 (m, 2H); 2.46 (m, 1H); 2.27 (q, J = 8.61, 1H); 1.98-1.75 (m, 4H); 1.64-1.47 (m, 6H). **^{13}C-NMR** (100 MHz, $CDCl_3$, 298 K): δ [ppm] = 129.1; 128.9; 128.4; 127.0; 75.3-74.9; 61.7; 57.9; 54.8; 50.3; 40.1; 35.1; 31.3-31.1 27.8; 27.3; 26.4; 22.8. **HRMS (ESI)**: (m/z) = calculated for $C_{20}H_{26}Cl_3N_2O_2^+$ [M+H]$^+$: 431.1054, found: 431.1054.

tert-butyl(3aS,5aS*,9aS*)-3-benzyloctahydro-1H-pyrrolo[3',2':2,3]cyclopenta [1,2-b]pyridine-6(7H)-carboxylate (**25**).* Synthesized according to **general procedure F** using **24** (726 mg, 1.7 mmol, 1 equiv), and Zn dust (3.3 g, 50.44 mmol, 30 equiv). Then, Et_3N (2.3 ml, 17 mmol, 10 equiv), and Boc_2O (556.5 mg, 2.55 mmol, 1.5 equiv). Purification by silica gel column chromatography with heptane/EtOAc (8: 2), yielded the Boc-protected compound **25** in 98% (571 mg, 1.6 mmol). **^{1}H-NMR** (400 MHz, CD_2Cl_2): δ [ppm] = 7.33-7.19 (m, 5H), 4.25 (s, 1H), 3.92 (s, 1H), 3.81 (d, *J* = 13.14, 1H), 3.12 (d, *J* = 13.02, 1H), 2.97-2.92 (m, 1H), 2.74-2.71 (m, 1H), 2.43- 2.40 (m, 1H), 2.26 (q, *J* = 8.87, 1H), 1.93-1.80 (m, 3H), 1.78-1.70 (m, 1H), 1.60-1.40 (m, 16H). **^{13}C-NMR** (100 MHz, $CDCl_3$, 298 K): δ [ppm] = 156.1, 140.29, 129.13, 128.43, 127.04, 79.18, 75.45, 61.9, 58.39, 54.85, 50.06, 39.7, 35.49, 31.70, 28.57, 26.74, 23.08. **HRMS (ESI):** (m/z) = calculated for $C_{22}H_{33}N_2O_2^+$ [*M*+H]$^+$: 357.2537, found: 357.2537.

tert-butyl(3aS,5aS*,9aS*)-3-(7H-pyrrolo[2,3-d]pyrimidin-4-yl)octahydro-1H-pyrrolo[3',2':2,3]cyclopenta[1,2-b]pyridine-6(7H)-carboxylate (**26**).* Synthesized according to **General Procedure G** using **25** (300 mg, 0.84 mmol, 1 equiv.), MeOH (C = 0.1M), Pd/C (30 mg, 10 mol%), AcOH (100 µl, 1.68 mmol, 2 equiv). Then, *N*-methyl-pyrrolidone (0.5M), 6-chloro-7-deazapurine (142 mg, 0.92 mmol, 1.1 equiv), and triethylamine (0.35 ml, 2.5 mmol, 3 equiv). Purification by column chromatography with

DCM/MeOH yielded the desired compound in 68% (222 mg, 0.84 mmol) as a white powder. **^{1}H-NMR** (400 MHz, MeOD-d_4, 298 K): δ [ppm] = 8.08 (s, 1H), (7.08 (d, *J* = 3.56, 1H), 6.63 (d, *J* = 3.57, 1H), 4.34 (q, *J* = 6.31, 1H), 4.32-4.17 (m, 2H), 4.01 (d, *J* = 13.36, 1H), 3.91 ($s_{(broad)}$), 1H), 2.90 (t, *J* = 12.69, 1H), 2.52 ($m_{(sym)}$), 1H), 2.20-2.17 (m, 1H), 2.10-1.99 (m, 1H), 1.95-1.87 (m, 1H), 1.81-1.74 (m, 1H), 1.71-1.61 (m, 3H), 1.56-1.52 (m, 1H), 1.48 (s, 9H). **^{13}C-NMR** (100 MHz, MeOD-d_4, 298 K): δ [ppm] = 157.4, 155.9, 151.8, 151.3, 122.0, 104.7, 102.7, 81.3, 69.0, 57.9, 51.2, 49.0, 34.1, 31.8, 30.9, 28.7, 26.7, 23.7. **HRMS (ESI):** (m/z) = calculated for $C_{21}H_{30}N_5O_2^+$ [*M*+H]$^+$: 384.2394, found: 384.2299.

3-((3aS,5aS*,9aS*)-3-(7H-pyrrolo[2,3-d]pyrimidin-4-yl)octahydro-1H-pyrrolo [3',2':2,3]cyclopenta[1,2-b]pyridin-6(7H)-yl)-3-oxopropanenitrile (**27**).* Compound **26** (198 mg, 0.5 mmol, 1 equiv) was dissolved in 12 ml of MeOH/HCl (1.25 M). The reaction was left to stir at room temperature for 16 h. The solvent was then evaporated to dryness, and half of the crude (62 mg, 0.2 mmol, 1 equiv), DCM, *N,N*-diisopropylethylamine (73 µl, 0.42 mmol, 2.0 equiv), cyanoacetic acid (24 mg, 0.2 mmol, 1.0 equiv), *N,N*-diisopropylcarbodiimide (25 mg, 0.2 mmol, 1.0 equiv), and DMAP (7 mg, 0.06 mmol, 0.3 equiv). The product was purified by RP-HPLC (gradient: 0–20% MeCN in water, 40 min.), affording the final compound in 9% as a TFA salt. **^{1}H-NMR** (400 MHz, MeOD-d_4, 298 K): δ [ppm] = 8.23 (s, 1H), 7.37 (m, 1H), 6.94 (m, 1H), 4.83-4.78 (m, 1H), 4.48 (m, 1H), 4.03-3.89 (m, 1H), 3.66 (m, 1H), 3.27 (m, 1H), 2.83 (t, *J* = 12.49, 0.5H), 2.65 (q, *J* = 7.90, 1H), 2.46-2.43 (m, 0.5H), 2.32-2.27 (m, 1H), 2.18-2.10 (m, 1H), 2.07-1.98 (m, 1.5H), 1.93-1.84 (m, 2H), 1.80-1.65 (m, 4H), 1.55-1.49 (m, 0.5H). **^{13}C-NMR** (100 MHz, MeOD-d_4, 298 K): δ [ppm] = 165.5, 164.9, 144.0, 125.3, 116.3, 105.7, 70.4, 60.9, 56.6, 43.0, 38.9, 33.9, 32.7, 31.2, 27,9, 26.8, 24.0, 23.3. **HRMS (ESI):** (m/z) = calculated for $C_{19}H_{23}N_6O^+$ [*M*+H]$^+$: 351.1928, found: 351.1928.

(3aS,5aS*,9aS*)-3-benzyl-6-(7H-pyrrolo[2,3-d]pyrimidin-4-yl)decahydro-1H-pyrrolo [3',2':2,3]cyclopenta[1,2-b]pyridine (**28**).* Synthesized according to **General Procedure J** using **24** (210 mg, 0.48 mmol, 1 equiv), 5 ml of DCE/AcOH (1: 1), Zn (dust) (941 mg, 14 mmol, 30 equiv). Then NMP (C = 0.1 M), 6-chloro-7-deazapurine (81 mg, 0.53 mmol, 1.1 equiv), and Et_3N (200 µl, 1.44 mmol, 3 equiv). Purification by column chromatography with DCM/ MeOH to afford the desired compound in 45% (80 mg, 0.21 mmol). **^{1}H-NMR** (400 MHz, CD_2Cl_2, 298 K): δ [ppm] = 11.16, (s,1H, 8.27 (s, 1H), 7.38-7.31 (m, 4H), 7.26-

7.23 (m, 1H), 7.11 (d, *J* = 3.56, 1H; (H-C(19)), 6.57 (d, *J* = 3.52, 1H; (H-C(20)), 5.00 (q, *J* = 6.14, 1H), 4.83 (d, *J* = 12.13, 1H), 3.89 (d, *J* = 13.08, 1H), 3.18, (d, *J* = 13.02, 1H), 3.07 (t, *J* = 11.82, 1H), 2.98 (t, *J* = 8.07, 1H), 2.53 (d, *J* = 3.64, 1H), 2.31 (q, *J* = 8.98, 1H), 2.20-1.92 (m, 3H), 1.85-1.53 (m, 7H). **^{13}C-NMR** (100 MHz, CD_2Cl_2, 298 K): δ [ppm] = 158.3, 152.5, 151.2, 140.2, 129.2, 128.5, 127.1, 120.3, 102.9, 102.2, 75.3, 63.4, 58.4, 54.8, 50.8, 40.5, 35.6, 27.4, 26.5, 23.2. **HR-MS (ESI):** (m/z) = calculated for $C_{23}H_{28}N_5^+$ [*M*+H]$^+$: 374.2339, found: 374.2349.

3-((3aS,5aS*,9aR*)-6-(7H-pyrrolo[2,3-d]pyrimidin-4-yl)decahydro-3H-pyrrolo [3',2':2,3]cyclopenta[1,2-b]pyridin-3-yl)-3-oxopropanenitrile (**29**).* Synthesized according to **General Procedure K** using **28** (40 mg, 0.1 mmol, 1 equiv), AcOH (37 µl, 0.6 mmol, 6 equiv), $Pd(OH)_2$ (21 mg, 0.15mmol, 1.5 equiv). Then DIPEA (70 µl, 0.4 mmol, 4 equiv), and 1-cyanoacetyl-3,5-dimethylpyrazole (24 mg, 0.15 mmol, 1.5 equiv). Purification by column chromatography with DCM/MeOH (98: 02) and a 0.1% of NH_3 to yield the final compound in 32% (11 mg, 0.03 mmol). **^{1}H-NMR** (400 MHz, CD_2Cl_2, 298 K): δ [ppm] = 11.59 (s, 1H), 8.29 (m, 1H), 7.14 (d, *J* = 3.40, 1H), 6.52-6.49 (m, 1H; (H-C(15)), 5.05-4.98 (m, 1H), 4.74 (m, 1H), 3.95-3.92 (m, 1H), 3.75-3.68 (m, 1H), 3.60-3.55 (m, 1H), 3.52-3.38 (m, 2H), 3.26-3.16 (m, 1H), 2.51-2.40 (m, 1H), 2.19-1.88 (m, 4H), 1.82-1.78 (m, 1H), 1.71-1.63 (m, 3H). **^{13}C-NMR** (100 MHz, CD_2Cl_2, 298 K): δ [ppm] = 159.8, 159.4, 157.9, 152.3, 150.7, 120.6, 114.2, 102.7, 101.4, 67.2, 66.7, 57.6, 51.9, 49.6, 46.8, 45.7, 40.9, 33.9, 33.0, 31.7, 31.0, 30.7, 28.8, 26.0, 25.6, 25.4, 22.7, 22.6. **HR-MS (ESI):** (m/z) = calculated for $C_{19}H_{23}N_6O^+$ [*M*+H]$^+$: 351.1928 found: 351.1928

Deconstructed analogues

*2-allyl-2-methylcyclopentane-1,3-dione (**30**).* Synthesized following the **general procedure B** using 2-methylcyclopentane-1,3-dione (10 g, 89 mmol, 1 equiv), allyl acetate (10.5 ml, 98 mmol, 1.1 equiv), and palladiumtetrakis (1.0 g, 0.89 mmol, 0.01 equiv). Column chromatography SiO_2: Hept/EtOAc (8:2) gave **30** (11 g, 72 mmol) in 80% yield. **^{1}H-NMR** (400 MHz, CD_2Cl_2, 298 K): δ [ppm] = 5.65-5.54 (m, 1H), 5.07-5-01 (m, 2H), 2.77-2.62 (m (sym), 4H), 2.32-2.32 (d, *J* = 7.4, 2H), 1.07 (s, 3H). **^{13}C-NMR** (100 MHz, CD_2Cl_2, 298 K): δ [ppm] = 215.8, 131.8, 119.2, 56.4, 39.8, 35.3, 18.3.

(3aS,6aS*)-1-benzyl-3a-methylhexahydrocyclopenta[b]pyrrol-4(1H)-one* ***(31)***. Synthesized according to **general procedure E** using **30** (5.0 g, 33 mmol, 1 equiv), O_3 (flow = 60 L/h), DMS (2.40 ml, 328 mmol, 10 equiv), benzyl amine (3.57 ml, 32.8 mmol, 1 equiv), 3 g of freshly dried molecular sieves, AcOH (1.87 ml, 32.8 mmol, 1 equiv) and $NaBH(AcO)_3$ (14 g, 65.6 mmol, 2 equiv). Purification by column chromatography with EtOAc/Hept (1: 9) yielded the **31** (4.75 g, 20 mmol) in 61% yield. **^{1}H-NMR** (400 MHz, CD_2Cl_2, 298 K): δ [ppm] = 7.32-7.20 (m, 5H); 4.05-4-02 (d, *J* = 13.4, 1H); 3.26-3.23 (d, *J* = 13.39, 1H); 2.84-2.79 (m (sym), 1H); 2.74 (d, *J* = 4.4, 1H); 2.60-2.50 (m, 1H); 2.27-2.16 (m, 2H); 2.06-1.97 (m, 2H); 1.95-1.84 (m, 1H); 1.64-1.56 (m, 1H); 1.09 (s, 3H. **^{13}C-NMR** (100 MHz, CD_2Cl_2, 298 K): δ [ppm] = 223.3, 139.8, 128.8, 128.5, 127.1, 73.6, 57.9, 56.1, 52.9, 34.4, 34.3, 22.0, 19.9. **HRMS (ESI):** (m/z) = calculated for $C_{15}H_{20}NO^+$ [*M*+H]$^+$: 230.1539, found 230.1539.

tert-butyl((3aR,4R*,6aS*)-1-benzyl-3a-methyloctahydrocyclopenta[b]pyrrol-4-yl) carbamate* ***(32)***. To a solution of compound **31** (1.00 g, 4.35 mmol, 1 equiv) in MeOH (C = 0.3M) was added ammonium acetate salt (3.4 g, 43.5 mmol, 10 equiv) and $NaBH_3CN$ (546.7 mg, 8.7 mmol, 2 equiv). The reaction was stirred at 50 °C for 19 h and was then cooled down to room temperature. The solvent was evaporated to dryness, and the residue was dissolved in DCM and Et_3N (0.5 ml, 5.2 mmol, 1.2 equiv), Boc_2O (950 mg, 4.35 mmol, 1 equiv) was added. The reaction was stirred at 22 °C for 1 h, and the solvent was evaporated under reduced pressure. The crude was purified by silica gel column chromatography with Hept/EtOAc (8:2) to furnish the Boc-protected amine **32** (913 mg, 2.7 mmol) in 63% yield. **^{1}H-NMR** (400 MHz, CD_2Cl_2): δ [ppm] = 7.32-7.20 (m, 5H, 3.88-3.85 (d, *J* = 13.0, 1H), 3.60-3.54 (m, 1H), 3.29-3.26 (d, *J* =13.0, 1H), 2.80-2.76 (t, *J* = 7.8, 1H), 2.48 (t, *J* = 3.2, 1H), 2.24-2.18 (m, 1H), 1.91-1.82 (m, 1H), 1.73-1.63 (m, 2.5H), 1.54-1.50 (m, 2H), 1.42 (s, 9H), 1.30-1.25 (m, 1.5H), 1.14 (s, 3H). **^{13}C-NMR** (100 MHz, CD_2Cl_2, 298 K): δ [ppm] = 156.0, 140.4, 129.1, 128.5, 127.1, 78.9, 76.3, 60.4, 59.2, 52.9, 51,8, 33.6, 31.6, 28.5, 27.9, C26.3. **HRMS (ESI):** (m/z) = calculated for $C_{20}H_{30}N_2O_2^+$ [*M*+H]$^+$: 331.2380, found 331.2380.

tert-butyl((3aR,4R*,6aS*)-3a-methyl-1-(7H-pyrrolo[2,3-d]pyrimidin-4-yl)octahydro cyclopenta[b]pyrrol-4-yl)carbamate* ***(33)***. Synthesized according to **General Procedure L** using **32** (400 mg, 1.21 mmol, 1 equiv.), MeOH (C = 0.16 M), Pd/C (40 mg, 10 wt%), and

AcOH (0.138 ml, 2.42 mmol, 2 equiv). Then, 6-chloro-7-deazapurine (204 mg, 1.33 mmol, 1.1 equiv), and K_3PO_4 (1.28 g, 6.05 mmol, 5 equiv). Purification by column chromatography with DCM / MeOH and 0.1% NH_3 yielded the desired compound in 92% (400 mg, 1.1 mmol) as a white solid. **^{1}H-NMR** (400 MHz, CD2Cl2): δ [ppm] = 11.47 (s, 1H), 8.26 (s, 1H), 7.09 (d, *J* = 3.43, 1H), 6.54-6-53 (d, *J* = 3.43, 1H), 4.64-4.62 (d, *J* = 7.76, 1H), 4.26-4.24 (m, 1H), 4.04-3.98 (m, 1H), 3.93-3.86 (m, 2H), 2.19-2.00 (m, 3H), 1.85-1.78 (m, 2H), 1.69-1.56 (m, 2H), 1.45 (s, 9H), 1.28 (s, 1H). **^{13}C-NMR** (100 MHz, CD_2Cl_2, 298 K): δ [ppm] = 156.1, 155.5, 151.8, 151.6, 120.5, 103.3, 101.7, 79.4, 69.5, 59.4, 52.4, 48.7, 32.2, 30.7, 29.9, 28.5, 24.6. **HR-MS (ESI):** (m/z) = calculated for $C_{19}H_{28}N_5O_2^+$ $[M+H]^+$: 358.2238 found: 358.2238.

2-cyano-N-((3aR,4R*,6aS*)-3a-methyl-1-(7H-pyrrolo[2,3-d]pyrimidin-4-yl)octahydro cyclopenta[b]pyrrol-4-yl)acetamide (**34a**).* Synthesized according to **General Procedure P** using **33** (24 mg, 0.067 mmol, 1 equiv), DCM (0.6 ml), TFA (0.2 ml). Then, 1-cyanoacetyl-3,5-dimethylpyrazole (16.4 mg, 0.1 mmol 1.5 equiv) and DIPEA (47 µL, 0.26 mmol, 4 equiv). Purification by silica gel column chromatography, furnished the final compound 95% (20 mg, 0.06 mmol). **^{1}H-NMR** (400 MHz, MeOD-d_4): δ [ppm] = 8.22 (s, 1H), 7.39 (m, 1H), 6.93 (s, H), 4.44-4.07 (br, 4H), 3.68-3.57 (m, 2H), 2.33-2.28 (m, 2H), 2.12-2.08 (m, 1H), 1.88-1.83 (m, 2H), 1.35 (s, 3H). **^{13}C-NMR** (100 MHz, MeOD-d_4, 298 K): δ [ppm] = 165.0, 143.3, 125.2, 116.1, 105.4, 72.0, 59.0, 30.0, 26.0, 24.4. **HR-MS (ESI):** (m/z) = calculated for $C_{17}H_{21}N_6O^+$ $[M+H]^+$: 325.1771, found: 325.1771.

N-((3aR,4R*,6aS*)-3a-methyl-1-(7H-pyrrolo[2,3-d]pyrimidin-4-yl)octahydrocyclo penta[b]pyrrol-4-yl)acrylamide (**34b**)*. Synthesized according to **General Procedure M** using **33** (50 mg, 0.139 mmol. 1 equiv), DCM/TFA (0.6 /0.2 ml). Then acryloyl chloride (10 µl, 0.125 mmol. 0.99 equiv), $NaHCO_3$ (47 mg, 0.55 mmol, 4 equiv) and DIPEA (50 µl, 0.27 mmol, 2 equiv). Purification by column chromatography using DCM /MeOH (9.5-0.5) and 0.1% NH_3, yielded the desired compound in 46% (21 mg, 0.067 mmol) as a pale brown colour. **^{1}H-NMR** (400 MHz, CD_2Cl_2): δ [ppm] = 8.22 (s, 1H), 7.38 (d, *J* = 3.58, 1H), 6.93 (d, *J* = 3.55, 1H), 6.40-6.25 (m, 2H), 5.71 (dd, *J* = 9.08, 2.19, 1H), 4.36 (m, 1H), 4.10-4.05 (broad, 2H), 2.35-2.27 (m, 2H), 2.12-2.05 (m, 1H), 1.90-1.83 (m, 2H), 1.36 (s, 3H). **^{13}C-NMR** (100 MHz, CD_2Cl_2, 298 K): δ [ppm] = 167.0, 148.9, 142.0, 130.3, 125.9, 123.7, 104.0,

70.8, 57.1, 53.5, 30.7, 28.8, 23.2. **HR-MS (ESI):** (m/z) = calculated for $C_{17}H_{22}N_5O^+$ [*M*+H]$^+$: 312.1819, found: 312.1817.

N-((3aR,4R*,6aS*)-3a-methyl-1-(7H-pyrrolo[2,3-d]pyrimidin-4-yl)octahydrocyclo penta[b]pyrrol-4-yl)cyanamide (**34c**)*. Synthesized according to **General Procedure O** using **33** (50 mg, 0.139 mmol. 1 equiv.), DCM/TFA (0.6/0.2). Then $NaHCO_3$ (27.8 mg, 0.278 mmol, 2 equiv), and cyanogen bromide (14.7 mg, 0.139 mmol, 1 equiv). Purification with RP-HPLC (gradient, 0-20% MeCN in water, 40 minutes) furnished the final compound as TFA salt 15% (6 mg, 0.02 mmol). **^{1}H-NMR** (400 MHz, MeOD-d$_4$): δ [ppm] = 8.23 (br, 1H), 7.37 (d, *J* = 3.56, 1H), 6.92 (br, 1H), 4.17-4.03 (br, 4H), 2.33-2.26 (m, 2H), 2.17-2.03 (m, 1H), 1.97-1.66 (m, 3H), 1.39 (s, 1H), 1.33 (s, 2H). **^{13}C-NMR** (100 MHz, MeOD-d$_4$, 298 K): δ [ppm] = 162.0, 143.4, 125.1, 105.4, 71.9, 64.3, 59.0, 32.2, 31.7, 30.9, 29.8, 24.3, 24.0, 19.8. **LCMS (ESI):** (m/z) = calculated for $C_{15}H_{19}N_6^+$ [*M*+H]$^+$: 283.17, found: 283.23.

N-((3aS,4R*,6aS*)-3a-methyl-1-(7H-pyrrolo[2,3-d]pyrimidin-4-yl)octahydrocyclo penta[b]pyrrol-4-yl)ethanesulfonamide (**34d**)*. Synthesized according to **General Procedure N** using **33** (20 mg, 0.055 mmol, 1 equiv), DCM/TFA (0.6 /0.2 ml). Then ethanesulfonyl chloride (5.2 µL, 0.055 mmol, 1 equiv.), $NaHCO_3$ (23 mg, 0.27 mmol, 5 equiv) and DIPEA (2-6 equiv). Purification by silica gel chromatography with DCM/MeOH (9.5: 0.5) and 0.1% ammonia yielded the final product as a white solid in 76% (14.6 mg, 0.04 mmol). **^{1}H-NMR** (400 MHz, MeOD-d$_4$, 298 K): δ [ppm] = 8.08 (s, 1H), 7.08 (d, *J* = 3.6, 1H), 6.63-6.61 (m, 1H), 4.27-4.24 (m, 1H), 4.09-3.98 (m, 1H), 3.93-3.86 (m, 1H), 3.69-3.65 (m, 0.4H), 3.61-3.53 (m, 0.6H), 3.13-3.06 (m, 2H), 2.46-2.38 (m, 0.4H), 2.32-2.19 (m, 1H), 2.18-2.03 (m, 2H), 1.84-1.68 (m, 2.5H); 1.60-1.50 (m, 0.5H), 1.37-1.33 (m, 3H), 1.30 (s, 3H), 1.21 (s, 1H). **^{13}C-NMR** (100 MHz, MeOD-d$_4$, 298 K): δ [ppm] = 156.2, 151.8, 151.3, 121.9, 104.3, 102.7, 70.1, 70.0, 62.9, 60.6, 54.8, 53.3, 48.2, 48.0, 36.2, 32.8, 32.0, 31.3, 30.3, 24.5, 20.6, 8.9. **HR-MS (ESI):** (m/z) = calculated for $C_{16}H_{24}N_5O_2^+$ [*M*+H]$^+$: 350.1645 found: 350.1643.

N-((3aR,4R*,6aS*)-1-benzyl-3a-methyloctahydrocyclopenta[b]pyrrol-4-yl)-7H-pyrrolo[2,3-d]pyrimidin-4-amine (**35**)*. To a cold solution of compound **32** (890 mg, 2.68 mmol, 1 equiv.) in DCM (6 ml), TFA (2 ml) was added dropwise, and the reaction was left

to stir at room temperature for 1h. The reaction was quenched with $NaHCO_3$ and extracted three times with DCM. The combined organic layers were dried over sodium sulfate, filtered, and the solvent was evaporated under reduced pressure. The desired de-protected amine was isolated in 95% (594 mg). **^{1}H NMR** (300 MHz, CD_2Cl_2) δ 7.26 – 7.05 (m, 5H), 3.75 (d, *J* = 13.0 Hz, 1H), 3.24 (d, *J* = 13.0 Hz, 1H), 2.72 – 2.63 (m, 1H), 2.60 (dd, *J* = 10.9, 5.8 Hz, 1H), 2.41 – 2.34 (m, 1H), 2.12 (ddd, *J* = 11.5, 8.6, 5.1 Hz, 1H), 1.72 – 1.52 (m, 2H), 1.52 – 1.39 (m, 1H), 1.36 – 1.26 (m, 2H), 1.06 (ddd, *J* = 12.0, 5.1, 1.0 Hz, 1H), 0.99 (s, 3H). **Second step**. To a solution of the deprotected amine (50 mg, 0.21 mmol, 1 equiv), in *t*-BuOH (C = 0.7M, 0.3 ml), was added 6-chloro-7-deazapurine (33 mg, 0.214 mmol, 1.02 equiv) and K_3PO_4 (222.3 mg, 1.05 mmol, 5 equiv) dissolved in 0.6 ml of deionised water. The reaction was stirred in a sealed tube at 80 °C for 6 days. The reaction mixture was then dissolved in DCM and water. The aqueous layer was washed 3 times with DCM and the combined organic layer was dried over Na_2SO_4, filtered, and the solvent was removed under reduced pressure Purification by silica gel column chromatography with DCM/ MeOH (97: 03) and 0.1% NH_3 yielded the desired compound **35** (58 mg, 0.17 mmol) in 80% yield. **^{1}H-NMR** (400 MHz, CD_2Cl_2): δ [ppm] = 11.42 (s, 1H), 8.28 (s, 1H), 7.37-7.25 (m, 5H), 7.07 (d, *J* = 3.4, 1H), 6.24 (d, *J* = 3.05, 1H), 5.89-5.87 (d, J = 9.2, 1H), 4.46-4.41 (m, 1H), 3.97-3.94 (d, *J* = 12.8, 1H), 3.25-3.23 (d, *J* = 12.8, 1H), 2.89-2.85 (t, *J* = 8.06, 1H), 2.65 (d, *J* = 4.35, 1H), 2.34-2.26 (m, 1H), 2.21-2.12 (m, 1H), 2.01-187 (m, 2H), 1.85-1.70, (m, 2H), 1.42-1.37 (dddd, *J* = 2.9, 1H), 1.24 (s, 3H). **^{13}C-NMR** (100 MHz, CD_2Cl_2, 298 K): δ [ppm] = 156.9, 152.16, 151.07, 140.32, 129.31, 128.61, 127.31, 121.11, 103.16, 98.20, 76.90, 59.99, 59.20, 54.24, 52.93, 33.04, 32.46, 27.71, 26.06. **HR-MS (ESI):** (m/z) = calculated for $C_{21}H_{26}N_5^+$ $[M+H]^+$: 348.2183, found: 348.2182.

3-((3aR,4R*,6aS*)-4-((7H-pyrrolo[2,3-d]pyrimidin-4-yl)amino)-3a-methylhexahydro cyclopenta[b]pyrrol-1(2H)-yl)-3-oxopropanenitrile (**36a**)*. Compound **35** (39 mg,0.11 mmol, 1 equiv) was dissolved in (MeOH/THF) (1:2) 3 ml. $Pd(OH)_2$ (38 mg, 0.27 mmol, 2.5 equiv) and AcOH (37 µl,0.66 mmol, 6 equiv) was added to the reaction mixture, and it was stirred under 1 atm of H_2 for 2 h. After full consumption of the starting material, the solution was filtered through Celite, and the solvent was evaporated under reduced pressure. The benzyl deprotected amine (0.11 mmol, 1equiv) was dissolved in MeCN (C = 0.03M), and 1-cyanoacetyl-3,5-dimethylpyrazole (27 mg, 0.165 mmol, 1.5 equiv) and DIPEA (77 µl, 0.44 mmol, 4 equiv) were added. The reaction was then stirred at 75 °C for

4 h. The reaction was cooled down to room temperature, and it was then poured into water and extracted three times with DCM. The solvent was evaporated under reduced pressure, and purification by preparative HPLC furnished the **36a** (7.9 mg, 0.024 mmol) in 21% yield over 2 steps as a TFA salt. **^{1}H-NMR** (400 MHz, MeOD-d_4): δ [ppm] = 8.31 (s, 1H), 7.35 (d, *J* = 2.73, 1H), 7.00-6.99 (d, *J* = 2.83, 1H), 4.63-4.58 (t, *J* = 8.58, 0.3H), 4.54-4.49 (t, *J* = 8.24, 0.7H), 4.02-3.96 (m, 1H), 3.92-3.60 (m, 4H), 2.36-2.22 (m, 1H), 2.20-1.99 (m, 3H), 1.87-1.82 (m, 1H), 1.71-1.60 (m, 1H), 1.30 (s, 3H). **^{13}C-NMR** (100 MHz, MeOD-d_4, 298 K): δ [ppm] = 163.5-163.3 $C(7)_{rot}$, 144.0, 125.3, 115.7, 102.8, 69.8-69.3, 60.9 $C(4)_{rot}$, 55.8, 53.5, 49.8, 48.2, 47.7, 33.1, 31.2, 30.2, 29.7, 29.1, 25.9, 24.0. *Due to rotamers, the full carbon could not be resolved.* **HR-MS (ESI):** (m/z) = calculated for $C_{17}H_{21}N_6O^+$ [*M*+H]$^+$: 325.1771, found: 325.1771.

1-((3aR,4R*,6aS*)-4-((7H-pyrrolo[2,3-d]pyrimidin-4-yl)amino)-3a-methylhexahydro cyclopenta[b]pyrrol-1(2H)-yl)prop-2-en-1-one (**36b**).* To a solution of compound **35** (290 mg, 0.83 mmol, 1 equiv) in DCM (5 ml), was added DMAP (101 mg, 0.83 mmol, 1 equiv) and Boc anhydride (364 mg, 1.66 mmol, 2 equiv). The reaction was allowed to stir for 3 hours at room temperature, followed by the addition of another portion of Boc-anhydride (182 mg, 1 equiv) and DMAP (101 mg, 1 equiv). The reaction was then allowed to stir for 16h at room temperature. The solvent was evaporated, and the crude was purified by column chromatography using DCM/MeOH (98:2) + 0.1% of ammonia. The desired Boc-benzyl-protected compound (364 mg, 0.81 mmol) was isolated in 97% yield. **^{1}H NMR** (400 MHz, CD_2Cl_2) δ 8.34 (s, 1H), 7.40 – 7.32 (m, 5H), 7.32 – 7.23 (m, 1H), 6.18 (d, *J* = 4.1 Hz, 1H), 5.96 (d, *J* = 9.2 Hz, 1H), 4.40 (dt, *J* = 10.1, 5.8 Hz, 1H), 3.95 (d, *J* = 12.8 Hz, 1H), 3.19 (d, *J* = 12.8 Hz, 1H), 2.85 (t, *J* = 8.5 Hz, 1H), 2.65 – 2.54 (m, 1H), 2.29 (td, *J* = 9.5, 7.5 Hz, 1H), 2.18 (ddt, *J* = 10.5, 7.0, 5.4 Hz, 1H), 1.95 – 1.82 (m, 2H), 1.78 (dtd, *J* = 10.9, 7.5, 5.7 Hz, 2H), 1.63 (s, 10H), 1.39 (ddd, *J* = 12.5, 7.6, 1.6 Hz, 1H), 1.22 (s, 3H). **^{13}C NMR** (101 MHz, CD_2Cl_2) δ 156.7, 154.3, 151.7, 148.1, 140.3, 129.3, 128.6, 127.4, 122.9, 105.4, 101.1, 84.5, 76.9, 59.9, 59.1, 32.8, 32.4, 28.2, 27.5, 25.8. **HRMS (ESI):** (m/z) = calculated for $C_{26}H_{34}O_2N_5^+$ [*M*+H]$^+$: 448.2707, found: 448.2707.

The boc-benzyl protected compound (150 mg, 0.33 mmol, 1 equiv) was dissolved in MeOH/THF (6 mL:5 mL). Then, AcOH (6 equiv,198 mmol, 15 µl) and $Pd(OH)_2$ (2.5 equiv, 0.83 mmol, 17.6 mg) were added. The reaction solution was purged with H_2, and it was

allowed to stir at room temperature for 1 hour. The solution was filtered out through Celite, and the desired benzyl de-protected compound was isolated in quantitative yield. To a solution of the benzyl deprotected amine (40 mg, 0.11 mmol, 1 equiv), in DCM (C = 0.03M) was added $NaHCO_3$ (18 mg, 0.22 mmol, 2 equiv), and DIPEA (180 µl, 1.43 mmol, 13 equiv). The reaction mixture was cooled down to 0 °C, and then acryloyl chloride (8 µl, 0.1 mmol, 0.9 equiv) was added dropwise. The reaction was stirred for 4 h at 0 °C –22 °C and then was poured into water and extracted three times with DCM. The combined organic layers were dried over sodium sulfate, filtered, and the solvent was removed under reduced pressure. The residue was dissolved in DCM (2 ml), cooled down at 0 °C, and TFA (1 ml) was added dropwise. The reaction mixture was stirred at rt for 1 h, and then the solvent was evaporated. The purification was done by preparative HPLC and the compound was isolated as TFA salt (7.8 mg, 0.018 mmol, 17%). **^{1}H-NMR** (400 MHz, MeOD-d_4, 298 K): δ [ppm] = 8.32 (br, 1H), 7.35 (s, 1H), 7.00 (1H), 6.67-6.55 (m, 1H), 6.33-3.26 (m, 1H), 5.78-5.74 (m, 1H), 4.62-4.50 (tt, *J* = 10.20, 1H), 4.13-4.06 (m, 1H), 3.94-3.88 (m, 0.5H), 3.82-3.57 (m, 2H), 2.36-2.00 (m, 4H), 1.87-1.61 (m, 2H), 1.32-1.29 (m, 3H). **^{13}C-NMR** (100 MHz, MeOD-d4, 298 K): δ [ppm] = 165.12, 143.01, 128.54, 128.52, 127.18, 126.98, 123.91, 1.01.35, 68.18, 67.61, 59.61, 57.68, 54.14, 51.77, 48.44, 46.59, 45.88, 34.44, 31.64, 29.95, 29.76, 28.30, 28.07, 22.68. **HR-MS (ESI):** (m/z) = calculated for $C_{17}H_{22}N_5O^+$ [*M*+H]$^+$: 312.1819, found: 312.1819.

(3aR,4R*,6aS*)-4-((7H-pyrrolo[2,3-d]pyrimidin-4-yl)amino)-3a-methylhexahydro cyclopenta[b]pyrrole-1(2H)-carbonitrile (**36c**)*. To a solution of **35** (10 mg, 0.03 mmol, 1 equiv) in MeOH/THF (0.7 ml/0.3 ml) was added AcOH (6 equiv, 0.168 mmol,10 µl) followed by $Pd(OH)_2$ (2.5 equiv, 0.07 mmol, 10 mg). The reaction was stirred at room temperature for 1 hour and was filtered through Celite. The solvent was evaporated, and the deprotected bicyclic diamine (9 mg, 0.03 mmol, 1 equiv) was dissolved in 1 mL of MeOH in inert atmosphere. The solution was cooled down to 0 °C, and $NaHCO_3$ (7.5 mg, 0.09 mmol, 3 equiv) was added. After sonication till clear solution, cyanogen bromide (3.1 mg, 0.03 mmol, 1 equiv) was added. The reaction was left to stir at 0 °C – 22 °C, for 16 h and then it was poured into water and extracted three times with DCM. The combined organic layers were dried over sodium sulfate, filtered, and the solvent was removed under reduced pressure. The product was purified using flash column chromatography (SiO_2: DCM/MeOH 9.8: 0.2 and 0.1% of NH_3). The desired product was

isolated in 77% (7.6 mg, 0.026 mmol) as a white solid. **^{1}H-NMR** (400 MHz, DMSO): δ [ppm] = 8.09 (s, 1H), 7.12-7.10 (d, *J* = 8.16, 0.5H), 7.08-7.07 (m, 1H), 6.67-6.66 (d, *J* = 3.14, 1H), 4.55-4.49 (m, 1H), 3.58-3.57 (d, *J* = 4.11, 1H), 3.54-3.49 (m, 1H), 3.42-3.31 (m, 1 H), 1.97-189 (m, 2H), 1.87-1.76 (m, 3H), 1.46-1.39 (quint, 1H), 1.29 (s, 3H). **^{13}C-NMR** (100 MHz, DMSO, 298 K): δ [ppm] = 156.0, 151.1, 150.1-149.9, 120.8-120.7, 116.2, 102.3, 98.8-98.7, 71.9, 58.2-58.1, 52.5, 50.5, 33.6, 29.6, 27.2, 25.4. **HR-MS (ESI):** (m/z) = calculated for $C_{15}H_{19}N_6^+$ [*M*+H]$^+$: 283.1666, found: 283.1660.

N-((3aR,4R*,6aS*)-1-(ethylsulfonyl)-3a-methyloctahydrocyclopenta[b]pyrrol-4-yl)-7H-pyrrolo[2,3-d]pyrimidin-4-amine (**36d**)*. Boc-Bn protected intermediate (170 mg, 0.39 mmol, 1 equiv), was dissolved in MeOH/THF (3: 3) ml and then AcOH (134 µl, 2.34 mmol, 6 equiv). Finally, $Pd(OH)_2$ (137 mg, 0.97 mmol, 2.5 equiv) was added to the reaction mixture, and the reaction was stirred at room temperature for 1 h. The reaction was filtered out through celite, washed out with MeOH and the crude was evaporated to dryness. 40 mg of the product from previous step (0.11 mmol, 1 equiv) was dissolved in DCM (C = 0.03M), and $NaHCO_3$ (17 mg, 0.22 mmol, 2 equiv) and DIPEA (180 µl, 1.43 mmol, 13 equiv) were added. The reaction mixture was cooled down to 0 °C, and then ethanesulfonyl chloride (9.5 µl, 0.1 mmol, 0.9 equiv) was added dropwise. The reaction was left to stir for 6 h at 0 °C –22 °C, and then was poured into water and extracted three times with DCM. The combined organic layers were dried over sodium sulfate, filtered, and the solvent was removed under reduced pressure. The residue was dissolved in DCM (2 ml), cooled down at 0 °C, and TFA (1 ml) was added dropwise. The reaction mixture was stirred at rt for 1 h, and then the solvent was evaporated. The product was purified by preparative HPLC (100% water to 50:50 water:MeCN in 55 min, 40 mL/min run). The compound was isolated as TFA salt (9.2 mg, 0.019 mmol, 18%). **^{1}H-NMR** (400 MHz, MeOD-d_4): δ [ppm] = 8.31 (s, 1H), 7.36-7.35 (d, *J* = 3.46, 1H), 7.00-6.99 (d, *J* = 3.55, 1H), 4.46-4.41 (t, *J* = 5.58, 1H), 3.79-3.77 (m, 1H), 3.55-3.50 (dtd, *J* = 3.28, 1H), 3.44-3.37 (m, 1H), 3.16-3.10 (dd, *J* = 3.41, 2H), 2.27-2.20 (m, 1H), 2.15-1.96 (m, 4H), 1.64-1.58 (dd, *J* = 3.02, 1H), 1.38-1.33 (m, 6H). **^{13}C-NMR** (100 MHz, MeOD-d_4, 298 K): δ [ppm] = 143.8, 102.7, 71.0, 61.3, 54.9, 49.3, 44.0, 33.5, 31.2, 29.8, 24.8, 8.0. **HR-MS (ESI):** (m/z) = calculated for $C_{16}H_{23}N_5O_2S^+$ [*M*+H]$^+$: 349.1572, found: 350.1645.

(3aS,4R*,6aS*)-1-benzyl-3a-methyloctahydrocyclopenta[b]pyrrol-4-ol (**37**)*. To a solution of the bicyclic ketone **31** (500 mg, 2.17 mmol, 1 equiv) in methanol (C = 0.5 M), cooled down to 0 °C, $NaBH_4$ (82 mg, 2.17 mmol, 1 equiv) was added portion-wise. The ice bath was removed, and the reaction mixture was stirred at 22 °C for 45 min. After full consumption of the starting material, the reaction was quenched with HCl (1M) and then extracted with an extensive amount of DCM. The combined organic phases were dried over sodium sulfate, filtered, and the solvent was removed under reduced pressure. Purification by column chromatography using DCM/MeOH/0.1% NH_3, gave **37** (462 mg, 1.99 mmol) in 92% yield. **^{1}H-NMR** (400 MHz, CD_2Cl_2): δ [ppm] = 7.32-7.21 (m, 5H), 3.93-3.90 (d, *J* = 13.0, 1H.), 3.54-3.52 (t, *J* = 4.52, 1H), 3.14-3.10 (d, *J* = 13.0, 1H), 2.84-2.80 (m, 1H), 2.51-2-49 (m, 1H), 2.25-2.13 (m, 2H), 2.05-1.96 (m, 1H), 1.92-1.84 (m, 1H), 1.77-1.65 (m, 2H), 1.39-1.30 (m, 1H), 1.05 (s, 3H). **^{13}C-NMR** (100 MHz, CD_2Cl_2, 298 K): δ [ppm] = 139.5, 128.8, 128.3, 126.9, 80.7, 76.1, 58.5, 53.7, 39.9, 31.0, 25.7 24.3. **HRMS (ESI):** (m/z) = calculated for $C_{15}H_{22}NO^+$ $[M+H]^+$: 232.1696, found: 232.1702.

tert-butyl((3aR,4S*,6aS*)-1-benzyl-3a-methyloctahydrocyclopenta[b]pyrrol-4-yl) carbamate (**38**)*. To a solution of **37** (1.7 g, 7.34 mmol, 1 equiv) in dry THF, cooled down to 0 °C were added triphenylphosphine (2.5 g, 9.5 mmol, 1.3 equiv) and DPPA (2 ml, 9.5 mmol, 1.3 equiv. Then, DIAD (1.88 ml, 9.5 mmol, 1.3 equiv) was added dropwise, and the reaction was stirred at room temperature for 2.5 h until the solution became a clear yellow color. Then, PPh_3 (5.7 g, 22 mmol, 3 equiv) and 20 equiv of deionized water were added, and the reaction was stirred at room temperature for 16 h. The solvent was evaporated under reduced pressure, and the crude was filtered through silica gel. The solvent was evaporated to dryness, and the residue was dissolved in DCM. Boc anhydride (1.38 g, 6.35 mmol, 1.1 equiv) and Et_3N (0.97 ml, 6.92, 1.2 equiv) were added at 0 °C, and the reaction was stirred at 22 °C for 1 hour. Evaporation of the solvent and purification by silica gel column chromatography with DCM/MeOH +0.1% NH_3, yielded the desired amine **38** (1.11 g, 3.35 mmol) in 46% yield. **^{1}H-NMR** (400 MHz, CD_2Cl_2): δ [ppm] = 7.32-7.22 (m, 5H), 4.54 (s, 1H), 3.84-3.78 (m, 2H), 3.80 (d, *J* = 13.12, 1H), 2.86-2.81 (m, 1H), 2.53-2.51 (m, 1H), 2.35-2.29 (q, *J* = 8.43, 1H), 2.16-2.08 (m, 1H), 1.84-1.77 (m, 1H), 1.66-1.59 (m, 1H), 1.57-1.47 (m, 3H), 1.42 (s, 9H), 0.99 (s, 3H). **^{13}C-NMR** (100 MHz, CD_2Cl_2, 298 K): δ [ppm] = 156.0, 140.4, 129.1, 128.4, 127.0, 78.9, 76.4, 60.4, 58.9, 53.4, 52.4, 39.1, 32.2, 28.5, 28,1.

tert-butyl((3aR,4S*,6aS*)-3a-methyl-1-(7H-pyrrolo[2,3-d]pyrimidin-4-yl)octahydro cyclopenta[b]pyrrol-4-yl)carbamate (**39**)*. Synthesised according to **General procedure L** using **38** (429 mg, 1.29 mmol, 1 equiv) in MeOH (8 ml), Pd/C (110 mg, 10 m%) and AcOH (148 µl, 2.6 mmol, 2 equiv). The reaction was stirred at 22 °C under a hydrogen atmosphere (balloon, 1 atm) for 30 minutes. Then, *t*-BuOH (3 ml), 6-chloro-7-deazapurine (200 mg, 1.3 mmol, 1.01 equiv), K_3PO_4 (1.37 g, 6.45 mmol, 5 equiv) in 5 ml of H_2O were used. Purification by silica gel column chromatography with DCM / MeOH (9.5:0.5) and 0.1% NH_3 yielded the desired compound **39** in 77% (360 mg, 1mmol) as a white powder. **^{1}H-NMR** (400 MHz, MeOD-d$_4$, 298 K): δ [ppm] = 8.07 (s, 1H), 7.08-7.09 (d, *J* = 3.60, 1H), 6.63-6-64 (d, *J* = 3.57, 1H), 4.25-4.22 (q, *J* = 4.02, 1H), 4.00-3.95 (m, 3H), 3.35 (MeOH), 2.43-2.35 (m, 1H), 2.20-2.14 (q, *J* = 6.33, 1H), 2.11-2.02 (m, 1H), 1.86-1.79 (m, 1H), 1.74-1.62 (m, 1H), 1.60-1.53 (m, 1H), 1.47 (s, 9H), 1.13 (s, 3H). **^{13}C-NMR** (100 MHz, MeOD-d$_4$, 298 K): δ [ppm] = 158.4, 156.2, 151.8, 151.3, 121.9, 104.5, 102.8, 80.0, 70.8, 58.3, 53.7, 49.8, 37.3, 31.5, 31.2, 28.8, 20.5. **HR-MS (ESI):** (m/z) = calculated for $C_{19}H_{28}N_5O_2^+$ [*M*+H]$^+$: 358.2238, found: 358.2238.

2-cyano-N-((3aR,4S*,6aS*)-3a-methyl-1-(7H-pyrrolo[2,3-d]pyrimidin-4-yl)octahydro cyclopenta[b]pyrrol-4-yl)acetamide (**40a**)*. The Boc protected amine **39** (30 mg, 0.083 mmol, 1 equiv) was dissolved in 2 ml DCM. The solution was cooled down to 0 °C, and then 25% (0.5 ml) of TFA was added. The reaction was stirred at room temperature for 1 hour, and the solvent was evaporated under reduced pressure. The residue was dissolved in acetonitrile (1 ml), and 1-cyanoacetyl-3,5-dimethylpyrazole (20 mg, 0.12 mmol, 1.5 equiv) and DIPEA (55 µl, 0.32 mmol, 4 equiv) were added, and the reaction was stirred for 4 h at 75 °C. Saturated sodium bicarbonate solution was added to the mixture and then extracted with DCM. The combined organic layers were dried over sodium sulfate, filtered, and the solvent was removed under reduced pressure. Purification by silica gel column chromatography using DCM/MeOH (9.5-0.5) and 0.1% NH_3, furnished the final compound **40a** (19mg, 0.05 mmol) in 69% yield as a white powder. **^{1}H-NMR** (400 MHz, DMSO): δ [ppm] = 11.57 (s, 1H), 8.23 (d, *J* = 8.74, 1H), 8.08 (s, 1H), 7.13- 7.11 (m, 1H), 6.54 (d, J = 2.49, (1H), 4.21-4.07 (m, 2H), 3.91-3.88 (m, 2H), 3.73-3.63 (q, *J* = 5.32, 2H), 2.37.2.28 (m, 1H), 2.10-1.97 (m, 2H), 1.75-1.71 (m, 1H), 1.64-1.55 (m, 1H), 1.54-1.45 (m, 1H), 1.05 (s, 3H). **^{13}C-NMR** (100 MHz, DMSO, 298 K): δ [ppm] = 161.9, 154.4,151.0, 120.7, 116.3, 102.3, 100.7, 68.7, 55.6, 51.8, 47.0, 35.8, 30.2, 29.9,

25.3, 20.0. **HR-MS (ESI):** (m/z) = calculated for $C_{17}H_{21}N_6O^+$ [M+H]$^+$: 325.1771, found: 325.1771.

N-((3aR,4S*,6aS*)-3a-methyl-1-(7H-pyrrolo[2,3-d]pyrimidin-4-yl)octahydrocyclo penta[b]pyrrol-4-yl)acrylamide (**40b**)*. The Boc protected diamine **39** (30 mg, 0.084 mmol. 1 equiv) was dissolved in DCM (1.5 ml). The solution was cooled down to 0 °C, and then 0.5 ml of TFA was added. The reaction was stirred at room temperature for 1 hour, and then the solvent was evaporated under reduced pressure. The material was dissolved in 2 ml of DCM under argon and cooled to 0 °C before the addition of acryloyl chloride (6 µl, 0.075 mmol, 0.9 equiv), $NaHCO_3$ (14 mg, 0.16 mmol, 2 equiv), and DIPEA (100 µl, 9 equiv). The reaction was stirred for 16 h, slowly warming from 0 °C to 22 °C. Then, saturated $NaHCO_3$ was added, and the solution was extracted 3 times with DCM. The combined organic layers were dried over sodium sulfate, filtered, and the solvent was removed under reduced pressure. Purification by column chromatography using DCM:MeOH (9.5:0.5) and 0.1% NH_3, yielded the desired compound **40b** (8.2 mg, 0.026 mmol) in 31% yield. **^{1}H-NMR** (400 MHz, DMSO): δ [ppm] = 11.57 (s, 1H), 8.08 (s, 1H), 8.06-8.04 (d, J = 8.87, 1H), 7.13-7.11 (t, J = 2.80, 1H), 6.56-6.55 (d, J = 1.60, 1H), 6.36-6.29 (m, 1H), 6.14 (dd, J = 2.23, 0.6H), 6.09 (d, J = 2.24, 0.4H); 5.63, (d, J = 2.24, 0.5H), 5.60 (d, J = 2.28, 0.5H); 4.28-4.14 (m, 2H), 3.90 (s$_{(broad)}$, 2H), 2.40-2.31 (m, 1H), 2.14-2.08 (m, 1H), 2.05-1.97 (m, 1H), 1.75-1.68 (m, 1H), 1.66-1.59 (m, 1H), 1.54-1.47 (m, 1H), 1.04 (s, 3H). **^{13}C-NMR** (100 MHz, DMSO, 298 K): δ [ppm] = 164.3, 154.4, 151.0, 131.7, 125.2, 120.6, 102.3, 100.7, 68.8, 56.0, 55.2, 47.0, 36.0, 30.1, 20.1. **HR-MS (ESI):** (m/z) = calculated for $C_{17}H_{22}N_5O^+$ [M+H]$^+$: 311.1819, found: 312.1823.

N-((3aR,4S*,6aS*)-3a-methyl-1-(7H-pyrrolo[2,3-d]pyrimidin-4-yl)octahydrocyclo penta[b]pyrrol-4-yl)cyanamide (**40c**)*. The Boc-protected amine **39** (40 mg, 0.11 mmol, 1 equiv) was dissolved in 2 ml of DCM. The solution was cooled down to 0 °Cm and then 0.5 ml of TFA was added. The reaction was stirred at room temperature for 1 hour, and the solvent was evaporated under reduced pressure. The material was dissolved in DCM, followed by the addition of $NaHCO_3$, (50 mg, 0.6 mmol, 5 equiv) and cyanogen bromide (17 mg, 0.16 mmol, 1.5 equiv). The reaction was stirred at 0 °C to rt for 16 h. After completion, the reaction mixture was poured into water and was extracted three times with DCM. The combined organic layers were dried over sodium sulfate, filtered

and the solvent was removed under reduced pressure. Column chromatography with DCM/MeOH (9.5-0.5) and 0.1% ammonia gave **40c** in 14% yield (4.6 mg, 0.016 mmol). **^{1}H-NMR** (400 MHz, MeOD-d_4): δ [ppm] = 8.08 (s, 1H), 7.09 (d, *J* = 3.61, 1H), 6.63-6.62 (d, *J* = 3.62, 1H), 4.34-4.31 (q, *J* = 4.03, 1H), 4.08-4.02 (m, 1H), 3.98-3.92 (m, 1H), 3.54-3.50 (t, *J* = 6.99, 1H), 2.51-2.42 (m, 1H), 2.24-2.09 (m, 2H), 1.94-1.81 (m, 2H), 1.65-1.56 (m, 1H), 1.26 (s, 3H). **^{13}C-NMR** (100 MHz, MeOD-d_4, 298 K): δ [ppm] = 156.1, 151.8, 151.3, 122.0, 118.2, 104.4, 102.6, 70.5, 63.5, 54.4, 48.4, 36.9, 31.1, 20.3. **HR-MS (ESI):** (m/z) = calculated for $C_{15}H_{19}N_6^+$ [*M*+H]$^+$: 283.1666, found: 283.1662.

N-((3aS,4S*,6aS*)-3a-methyl-1-(7H-pyrrolo[2,3-d]pyrimidin-4-yl)octahydrocyclo penta[b]pyrrol-4-yl)ethanesulfonamide (**40d**).* The Boc-protected amine **39** (30 mg, 0.083 mmol, 1 equiv) was dissolved in 2 ml DCM. The solution was cooled to 0 °C, and then 0.5 ml of TFA was added. The reaction was stirred at room temperature for 1 hour, and then the solvent was evaporated under reduced pressure and dried in high vacuum. The residue was dissolved in DCM under argon and cooled to 0 °C, followed by the addition of DIPEA (80 µl, 0.45 mmol, 4 equiv), and ethanesulfonyl chloride (7.6 µl, 0.08 mmol, 1 equiv). The reaction was stirred for 16h, slowly warming from 0 °C to RT. Then, water and saturated $NaHCO_3$ were added, and the mixture was extracted 3 times with DCM. The combined organic layers were dried over sodium sulfate, filtered, and the solvent was removed under reduced pressure. Purification by column chromatography with DCM/MeOH (9.8:0.2) and 0.1% ammonia furnished the final compound **40d** (6 mg, 0.017 mmol) in 20%. **^{1}H-NMR** (400 MHz, MeOD-d_4): δ [ppm] = 8.07 (s, 1H), 7.07 (d, *J* = 3.60, 1H), 6.625 (d, *J* = 3.61, 1H), 4.27.4.24 (q, *J* = 3.94, 1H), 4.09-4.03 (m, 1H), 3.94 (broad peak, 1H), 3.69-3-65 (q, *J* = 5.44, 1H), 3.12-3.06 (q, *J* = 7.36, 2H), 2.46-3.38 (m, 1H), 2.26-2.21 (m, 1H), 2.15-2.07 (m, 1H), 1.85-1.74 (m, 2H), 1,60-1.50 (m, 1H), 1.36-1.33 (t, *J* = 7.36, 3H), 1.21 (s, 3H). **^{13}C-NMR** (100 MHz, MeOD-d_4, 298 K): δ [ppm] = 156.1, 151.7, 151.3, 12.9, 104.5, 102.7, 69.9, 60.5, 58.3, 49.8, 48.1, 36.1, 31.9, 30.6, 20.6, 8.6. **HR-MS (ESI):** (m/z) = calculated for $C_{16}H_{24}N_5SO_2^+$ [*M*+H]$^+$: 350.1645, found: 350.1645.

N-((3aR,4S*,6aS*)-1-benzyl-3a-methyloctahydrocyclopenta[b]pyrrol-4-yl)-7H-pyrrolo[2,3-d]pyrimidin-4-amine (**41**).* Boc-protected amine **38** (415 mg, 1.25 mmol, 1 equiv) was dissolved in DCM, cooled down to 0 °C, and then 1.5 ml of TFA was added. The reaction was stirred at room temperature for one hour until the starting material was

fully consumed. The reaction was quenched with sodium carbonate solution and extracted with DCM. The combined organic layers were dried over sodium sulfate, filtered, and the solvent was removed under reduced pressure. A part of the deprotected amine (120 mg, 0.521 mmol, 1 equiv), was dissolved in *t*-BuOH (C = 0.17M, 3 ml), and 6-chloro-7-deazapurine (160 mg, 1 mmol, 2 equiv) was added. A solution of K_2PO_4 (552 mg, 2.6 mmol, 5 equiv) in 2.5 ml of deionized water was then added to the reaction solution. The reaction was stirred in a sealed tube at 120 °C for 72 h. The reaction mixture was then dissolved in DCM and quenched with deionized water. The aqueous layer was washed 3 times with DCM and dried over Na_2SO_4, filtered, and the solvent was evaporated under reduced pressure. Purification by column chromatography with DCM / MeOH (97: 03) and 0.1% NH_3 yielded the desired compound in 50% yield (92 mg, 0.26 mmol) as white powder. **^{1}H-NMR** (400 MHz, CD_2Cl_2): δ [ppm] = 11.74 (s, 1H), 8.31 (s, 1H), 7.38-7.32 (m, 5H), 7.14-7.13 (d, *J* = 3.48, 1H), 6.43 (d, *J* = 3.29, 1H), 3.88-3.85 (d, *J* = 13.01, 1H), 3.36-3.33 (d, *J* = 13.01, 1H), 2.93 (m(sym), 1H), 2.65-2.62 (m, 1H), 2.41-2.28 (m, 2H), 2.05-1.95 (m, 1H), 1.86-1-57 (m, 4H), 1.10 (s, 3H). **^{13}C-NMR** (100 MHz, CD_2Cl_2, 298 K): δ [ppm] = 157.1, 151.9, 151.3, 140.3, 129.2, 128.4, 127.1, 121.4, 102.8, 98.3, 76.7, 60.3, 58.9, 54.0, 52.8, 39.0, 32.6, 28.1, 21.8. **HR-MS (ESI):** (m/z) = calculated for $C_{21}H_{26}N_5^+$ [*M*+H]$^+$: 348.2183, found: 348.2183.

3-((3aR,4S*,6aS*)-4-((7H-pyrrolo[2,3-d]pyrimidin-4-yl)amino)-3a-methylhexahydro cyclopenta[b]pyrrol-1(2H)-yl)-3-oxopropanenitrile (**42a**).* Compound **41** (24 mg,0.07 mmol, 1 equiv) was dissolved in (MeOH/THF) (1:2) 0.9 ml. $Pd(OH)_2$ (25.2 mg, 0.17 mmol, 2.5 equiv) and AcOH (24 µl,0.42 mmol, 6 equiv) was added to the reaction mixture, and it was stirred under 1 atm of H_2 for 2 h. After full consumption of the starting material, the solution was filtered through Celite and the solvent was evaporated under reduced pressure. The benzyl deprotected amine (21 mg, 0.081 mmol, 1 equiv) was dissolved in MeCN (2 ml), followed by the addition of DIPEA (170 µl, 0.97 mmol, 12 equiv) and 1-cyanoacetyl-3,5-dimethylpyrazole (20 mg, 0.12 mmol, 1.5 equiv). The reaction was stirred at 75 °C for 4 h and then cooled to room temperature. The mixture was diluted in DCM and quenched with sat $NaHCO_3$. The aqueous layer was extracted three times with DCM, and the combined organic layer was dried over sodium sulfate, filtered, and the solvent was removed under reduced pressure. Purification by column chromatography with DCM/MeOH (98: 02) and 0.1% NH_3 gave compound **42a** (9.4 mg, 0.028 mmol) in

36% yield. ^{1}**H-NMR** (400 MHz, DMSO, 80 °C): δ [ppm] = 11.23 (s, 1H), 8.08 (s, 1H), 7.03 (m, 1H), 6.83 (m, 1H), 6.65 (m, 1H), 4.78 (q, *J* = 8.04, 1H), 3.89-3.44 (m, 5H), 2.33-2.19 (m, 2H), 2.07 (m, 1H), 1.88-1.78 (m, 1H), 1.76-1.60 (m, 1H), 1.58-1.49 (m, 1H), 1.04 (s, 3H). ^{13}**C-NMR** (100 MHz, DMSO, 298 K): δ [ppm] = 160.7, 156.0, 151.2, 150.1, 120.6, 116.0, 102.3, 98.7, 68.3-68.0, 55.9-55.4, 53.2, 51.4, 46.08-45.3, 36.5-35.8, 30.7, 30.4, 29.9, 28.9, 25.54-25.2, 20.7-20.0. **HR-MS (ESI):** (m/z) = calculated for $C_{17}H_{21}N_6O^+$ [*M*+H]$^+$: 325.1771, found: 325.1776.

1-((3aR,4S*,6aS*)-4-((7H-pyrrolo[2,3-d]pyrimidin-4-yl)amino)-3a-methylhexahydro cyclopenta[b]pyrrol-1(2H)-yl)prop-2-en-1-one (**42b**)*. To a solution of compound **41** (16 mg, 0.046 mmol, 1 equiv) in MeOH/THF (1:2) was added $Pd(OH)_2$ (15 mg, 0.11 mmol, 2.5 equiv) and AcOH (37 µl, 0.66 mmol, 6 equiv). The reaction was stirred for 2 h at 22 °C under H_2 atmosphere (1 atm). After complete consumption of the starting material, the reaction was filtered through Celite, washed with MeOH, and the solvent was evaporated under reduced pressure. The residue was dissolved in DCM (3 ml), and DIPEA (88 µl, 0.5 mmol, 12 equiv) was added. The reaction was sonicated for 5 minutes and then cooled to 0 °C. Acryloyl chloride (3.2 µl, 0.04 mmol, 1 equiv) was added slowly, and the reaction was stirred for 16 h, slowly warming from 0 °C to 22 °C. The reaction mixture was then diluted with DCM, and deionized water was added. The aqueous layer was washed three times with DCM, and the combined organic layer was dried over Na_2SO_4, filtered, and the solvent was removed under reduced pressure. Purification by column chromatography with DCM:MeOH (97:03) and 0.1% NH_3 yielded the desired compound in 28% yield (4 mg, 0.012 mmol). ^{1}**H-NMR** (400 MHz, MeOD-d$_4$): δ [ppm] = 8.10 (d, *J* = 4.77, 1H), 7.07-7.06 (m, 1H), 6.68-6.58 (m, 2H), 6.32-6.24 (m, 1H), 5.76-5.72 (m, 1H), 4.82-4.77 (m, 1H), 4.09-4.01 (m, 1H), 3.96-3.89 (m, 0.5H), 3.87-3.80 (m, 1H), 3.74-3.67 (m, 0.5H), 2.46-2.38 (m, 1H), 2.34-2.11 (m, 2H), 1.98-1.86 (m, 1H), 1.83-1.72 (m, 1H), 1.65-1.56 (m, 1H), 1.14 (s, 1H), 1.12 (s, 2H). ^{13}**C-NMR** (100 MHz, MeOD-d$_4$, 298 K): δ [ppm] = 165.2, 156.7, 150.8, 149.1, 128.8, 128.6, 126.7, 126.5, 120.8, 102.9, 98.4, 68.5, 68.4, 56.1-55.9, 53.5, 51.4, 46.3, 45.1, 36.5, 35.1, 31.1, 30.5, 30.4, 29.1, 19.5, 19.3.

(3aR,4S*,6aS*)-4-((7H-pyrrolo[2,3-d]pyrimidin-4-yl)amino)-3a-methylhexahydro cyclopenta[b]pyrrole-1(2H)-carbonitrile (**42c**)*. To a solution of compound **41** (40 mg, 0.116 mmol, 1 equiv) in 3.5 mL of MeOH/THF (1.5: 2) were added $Pd(OH)_2$ (40 mg, 0.28

mmol, 2.5 equiv) and AcOH (37 µl, 0.66 mmol, 6 equiv). The reaction was stirred for 1 h at 22 °C under H_2 atmosphere (1 atm). After full consumption of the starting material, the reaction was filtered out through Celite, washed with MeOH, and the solvent was evaporated under reduced pressure. The residue was dissolved in DCM (3 ml), DIPEA (76 µl, 0.44 mmol, 4 equiv) and $NaHCO_3$ (46 mg, 0.55 mmol, 5 equiv) were added. The reaction was sonicated for 5 minutes and was cooled down to 0 °C. Cyanogen bromide (11 mg, 0.11 mmol, 1 equiv) was added slowly, and the reaction was stirred for 16 h at 0 °C- 22 °C. A saturated solution of $NaHCO_3$ was added to the mixture, and the aqueous layer was extracted with DCM. The combined organic layer was dried over sodium sulfate, filtered out, evaporated, and the crude was purified by silica gel column chromatography to yield the final compound in 42% yield (13.8 mg, 0.048 mmol). **^{1}H-NMR** (400 MHz, MeOD-d_4): δ [ppm] = 8.09 (s, 1H), 7.06 (d, *J* = 3.52, 1H), 6.65 (d, *J* = 3.51, 1H), 4.77 (t, *J* =7.67, 1H), 3.78-3.72 (m , 1H), 3.69-3.67 (m, 1H), 3.56-3.51 (m, 1H), 2.31-2.18 (m, 3H), 1.99-1.73 (m, 3H), 1.11 (s, 1H). **^{13}C-NMR** (100 MHz, MeOD-d_4, 298 K): δ [ppm] = 158.10, 152.20, 150.61, 122.30, 117.99, 1.04.30, 99.87, 73.67, 57.27, 55.05, 51.26, 38.15, 31.40, 28.65, 19.86. **HR-MS (ESI):** (m/z) = calculated for $C_{15}H_{19}N_6^+$ [*M*+H]$^+$: 282.1593, found: 283.1666.

Spiro analogues

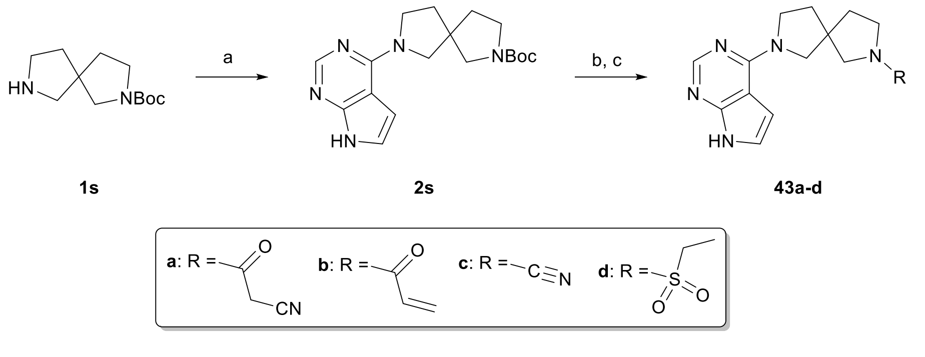

Scheme S1. Synthesis of analogues **43a-d**.

*tert-butyl(RS)-7-(7H-pyrrolo[2,3-d]pyrimidin-4-yl)-2,7-diazaspiro[4.4]nonane-2-carboxylate (**2s**)*. Commercially available **1s** (400 mg, 1.76 mmol, 1 equiv) was dissolved in *t*-BuOH (6 ml). 6-chloro-7-deazapurine (434 mg, 2.82 mmol, 1.1 equiv) was then added to the reaction mixture, followed by the addition of K_3PO_4 (1.86 g, 8.8 mmol, 5 equiv) in 8 ml of deionized water. The reaction was stirred in a sealed tube at 120 °C for

72 h. The reaction mixture was then dissolved in DCM and quenched with deionized water. The aqueous layer was washed 3 times with DCM and dried over Na_2SO_4, filtered, and the organic solvent was evaporated under reduced pressure. Purification by column chromatography with DCM / MeOH (97:03) and 0.1% NH3 yielded the desired compound **2s** as a white powder in 49 % yield (0.87 mmol). **^{1}H NMR** (300 MHz, $CDCl_3$) δ 11.98 (s, 1H), 8.33 (s, 1H), 7.09 (d, *J* = 3.5 Hz, 1H), 6.54 (d, *J* = 3.5 Hz, 1H), 4.13 – 3.87 (m, 2H), 3.87 – 3.66 (m, 2H), 3.61 – 3.23 (m, 4H), 2.17 – 1.81 (m, 4H), 1.45 (s, 9H).

*(RS)-3-(7-(7H-pyrrolo[2,3-d]pyrimidin-4-yl)-2,7-diazaspiro[4.4]nonan-2-yl)-3-oxopropanenitrile (**43a**)*. Compound **2s** (201.7 mg, 0.3631 mmol, 1 eq) was suspended in 4 ml DCM (0.1 M). DIPEA (0.126 ml, 0.726 µmol, 2 eq) was added to the mixture, followed by cyanoacetic acid (30.9 mg, 0.579 mmol, 1 eq), DIC (57 µl, 0.58 mmol, 1 eq.), and DMAP (21.2 mg, 0.174 mmol. 0.3 eq.). The reaction mixture was stirred at 22 °C for 18 hours. The solvent was evaporated under reduced pressure, and the crude was purified by flash column chromatography (12.5 g golden silica column, gradient 5% to 20% MeOH in DCM). An additional RP-LC (12.5 g column, gradient 5% to 100% acetonitrile in water, no addition of TFA) was done, giving the **43a** (8.2 mg, 26.42 µmol) as a white solid in a yield of 7% yield. **^{1}H NMR** (300 MHz, MeOD) δ 8.09 (s, 1H), 7.10 (d, *J* = 3.6 Hz, 1H), 6.70 (dd, *J* = 3.6, 1.7 Hz, 1H), 3.98 (d, *J* = 9.9 Hz, 2H), 3.82 (d, *J* = 9.2 Hz, 3H), 3.71 – 3.44 (m, 4H), 2.18 – 1.96 (m, 4H). **^{13}C NMR** (101 MHz, DMSO) δ 161.8, 161.6, 155.2, 151.4, 151.4, 121.1, 116.5, 102.9, 101.4, 56.4, 55.4, 54.8, 47.2, 45.6, 34.8, 33.4, 25.7. The characterization is in accordance with the literature. [J. Med. Chem. 2020, 63, 7163−7185].

*(RS)-1-(7-(7H-pyrrolo[2,3-d]pyrimidin-4-yl)-2,7-diazaspiro[4.4]nonan-2-yl)prop-2-en-1-one (**43b**)*. Compound **2s** (175 mg, 0.315 mmol, 1 eq.) was suspended in 3.5 ml DCM (0.1 M), and DIPEA (0.11 ml, 0.63 mmol, 2 eq.) was added. Then, acrylic acid (22 µl, 0.31 mmol, 1 eq.), DIC (49 µl, 0.31 mmol, 1 eq.), and DMAP (11 mg, 94 µmol, 0.3 eq.) were added to the reaction mixture, and the reaction was stirred for 16h. The solvent was removed under reduced pressure, and the crude was purified by flash column chromatography (gradient elution from 5% to 10% MeOH in DCM), affording the desired product with some impurities. Further purification with RP-LC (Combi flash, 15 g column, gradient 5% to 100% acetonitrile in water, no addition of TFA) afforded **43b** a

white solid in a yield of 13% (12 mg, 0.041 mmol). **^{1}H NMR** (400 MHz, MeOD) δ 8.22 (s, 1H), 7.38 (d, *J* = 3.7 Hz, 1H), 7.00 (d, *J* = 3.1 Hz, 1H), 6.69 – 6.53 (m, 1H), 6.29 (ddd, *J* = 16.8, 5.6, 2.0 Hz, 1H), 5.76 (ddd, *J* = 14.9, 10.4, 2.0 Hz, 1H), 4.17 (d, *J* = 61.5 Hz, 2H), 3.98 – 3.81 (m, 2H), 3.81 – 3.65 (m, 3H), 3.65 – 3.55 (m, 1H), 2.33 – 2.05 (m, 4H). ^{13}C NMR (101 MHz, MeOD) δ 166.9, 166.9, 143.1, 129.8, 129.4, 128.7, 128.5, 125.1, 105.4, 103.4, 56.0, 55.2, 47.0, 46.2, 35.5, 34.0. The compound started to decompose after a few hours at room temperature.

7-(7H-pyrrolo[2,3-d]pyrimidin-4-yl)-2,7-diazaspiro[4.4]nonane-2-carbonitrile ***(43c)***. Synthesized according to general Procedure O using **2s** (50 mg, 0.14 mmol, 1equiv), cyanogen bromide (14 mg, 0.14 mmol, 1 equiv), $NaHCO_3$ (47 mg, 0.56 mmol, 4 equiv) and DIPEA (0.2 ml, 0.84 mmol, 6 equiv). Purification by silica gel chromatography with DCM/MeOH (95: 05) + 0.1% NH_3, furnished the final compound **43c** in 66% yield (25.8 mg, 0.09 mmol) as white powder. **^{1}H-NMR** (400 MHz, DMSO): δ [ppm] = 11.59 (s, 1H), 8.08 (s, 1H), 7.11 (q, *J* = 1.88), 6.59-6.58 (m, 1H), 3.38 (br, 4H), 3.57-3.48 (m, 2H), 3.04-3.65 (m, 2H), 2.01-1.88 (m, 4H). **^{13}C-NMR** (100 MHz, DMSO, 298 K): δ [ppm] = 154.75, 151.09, 150.96, 120.63, 117.41, 102.39, 100.78, 58.12, 55.63, 49.39, 46.63, 34.35. **HRMS (ESI):** (m/z) = calculated for $C_{14}H_{17}N_6^+$ [*M*+H]$^+$: 269.1509, found: 269.1509.

4-(7-(ethylsulfonyl)-2,7-diazaspiro[4.4]nonan-2-yl)-7H-pyrrolo[2,3-d]pyrimidine ***(43d)***. Synthesised according to general procedure N using **2s** (100 mg, 0.14 mmol, 1equiv), ethanesulfonyl chloride (27 µl, 0.29 mmol, 1 equiv), $NaHCO_3$ (97 mg, 1.16 mmol, 4 equiv) and DIPEA (0.3 ml, 1.7 mmol, 6 equiv). Purification by silica gel chromatography with DCM/MeOH (95: 05) + 0.1% NH3, furnished the final compound **43d** in 76% yield (74.3 mg, 0.22 mmol) as a white powder. **^{1}H-NMR** (400 MHz, DMSO): δ [ppm] = 8.08 (s, 1H), 7.12-7.11 (m, 1H), 6.58-6.57 (m, 1H), 3.83-3.69 (br, 4H), 3.42 (t, *J* = 6.95, 2H), 3.33-3.26 (m, 3H), 3.13 (q, *J* = 7.36, 2H), 2.02-1.93 (m, 4H), 1.22 (t, *J* = 7.35, 3H). **^{13}C-NMR** (100 MHz, DMSO, 298 K): δ [ppm] = 154.78, 151.10, 150.96, 102.62, 102.39, 100.75, 55.94, 55.59, 42.20, 34.55, 7.63. **HR-MS (ESI):** (m/z) = calculated for $C_{15}H_{22}N_5O_2S^+$ [*M*+H]$^+$: 336.1489, found: 336.1489

X-ray crystal deposition

The structures are shown as ORTEP with ellipsoids drawn at the 50% probability level. Hydrogen atoms (white spheres, arbitrary radius) were located in the difference Fourier map and refined freely.

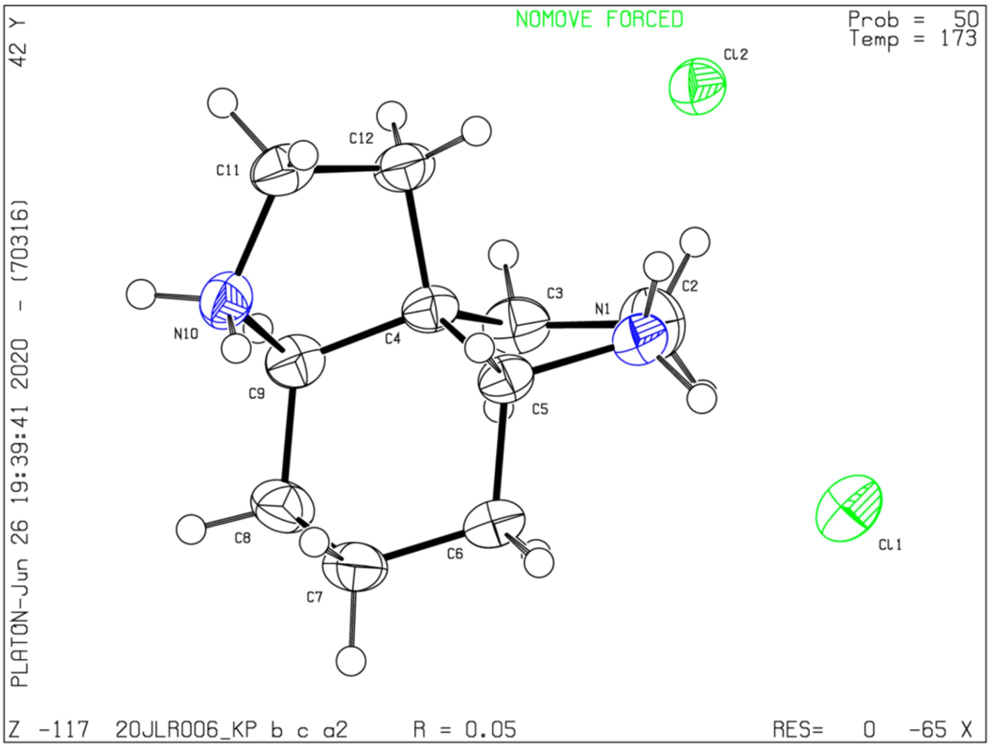

Figure S1. X-ray structure of deprotected **12a** (CCDC 2482160)

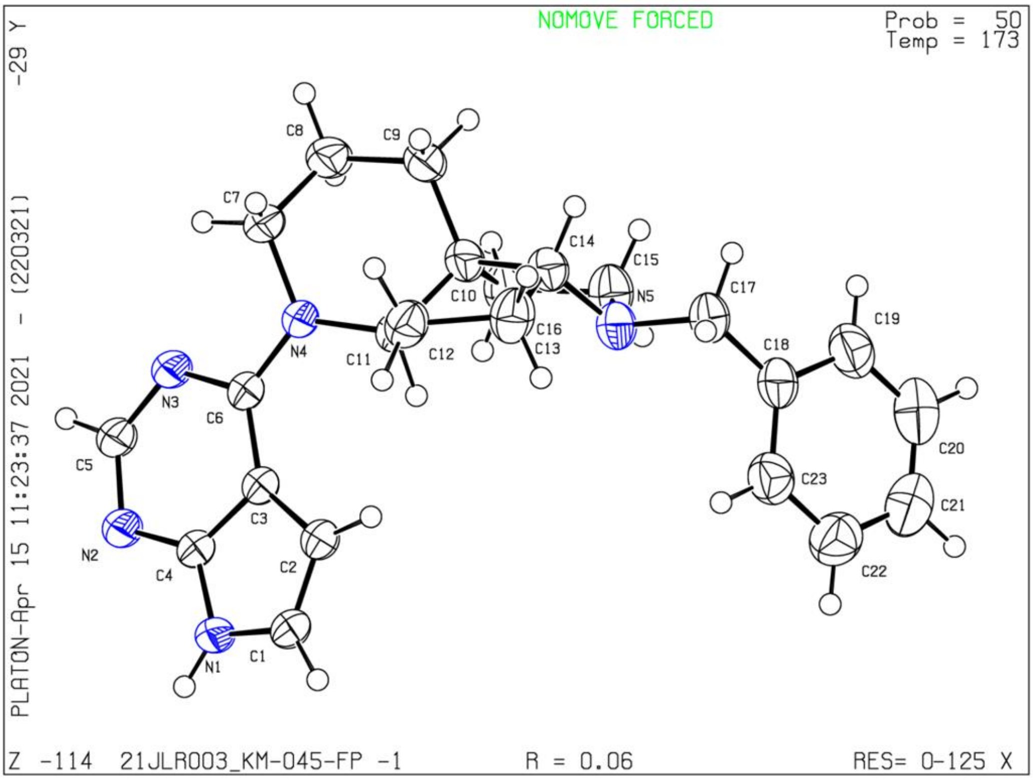

Figure S2. X-ray structure of deprotected **28** (CCDC 2482161)

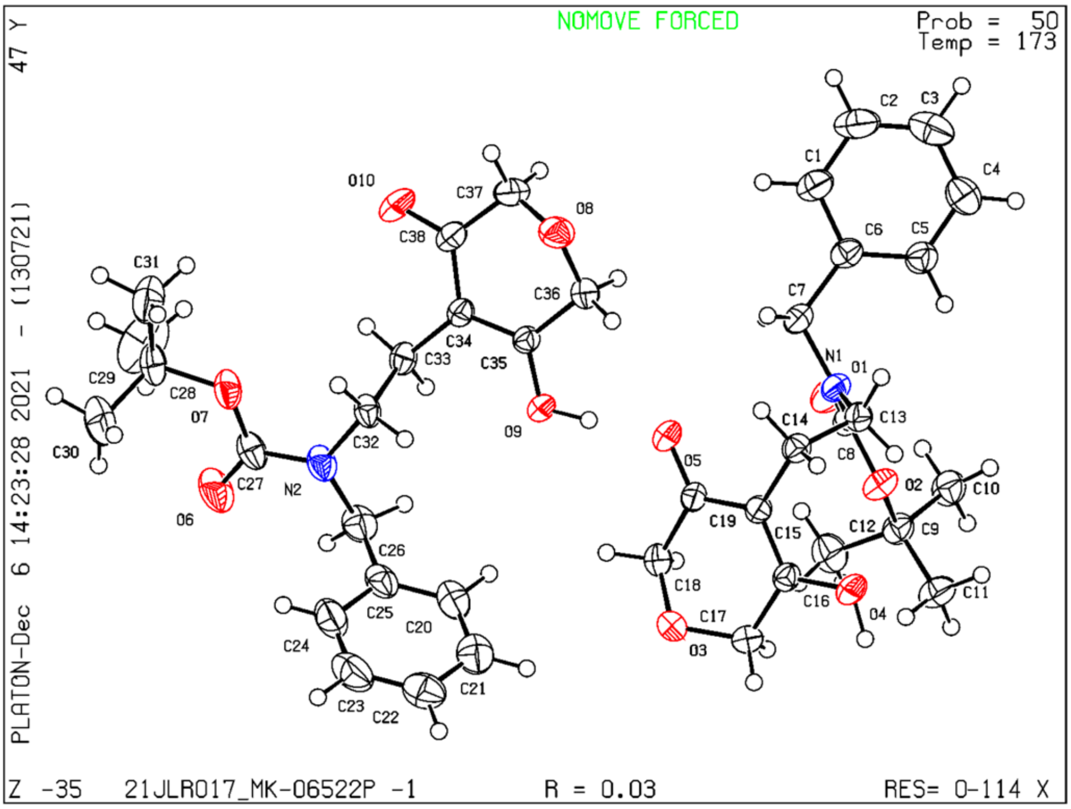

Figure S3. X-ray structure of deprotected **7b** (CCDC 2482162)

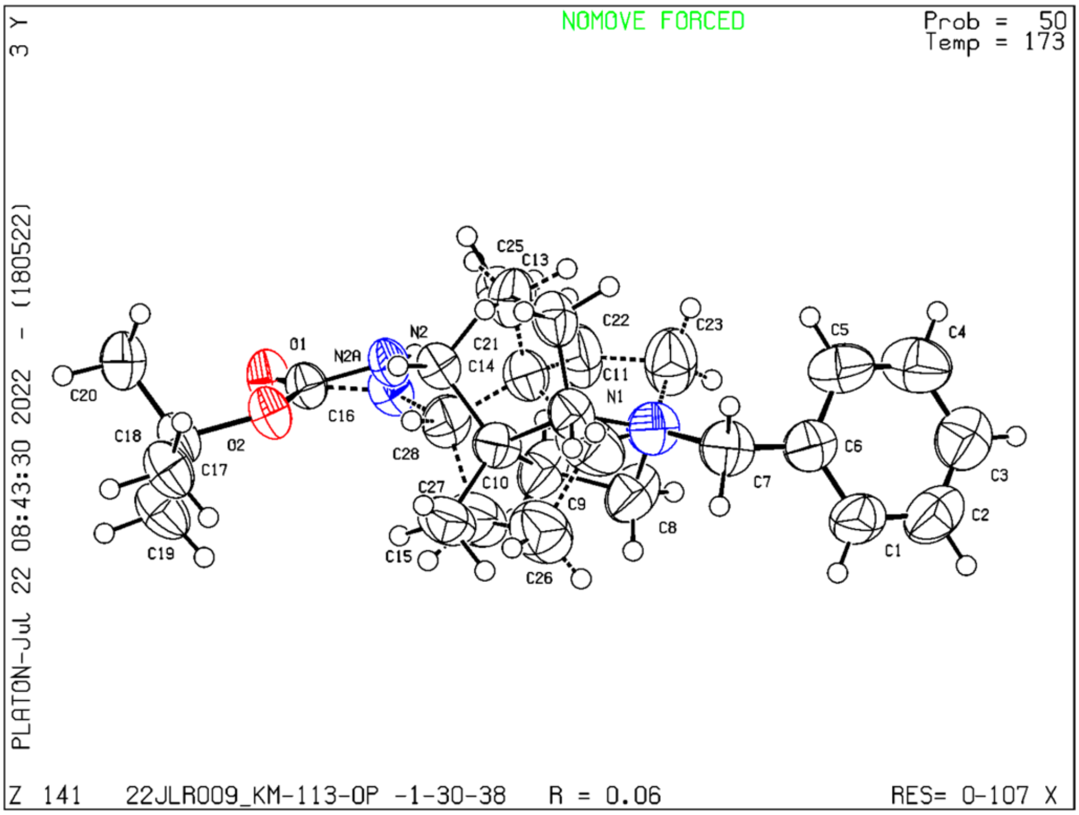

Figure S4. X-ray structure of deprotected **32** (CCDC 2482163)

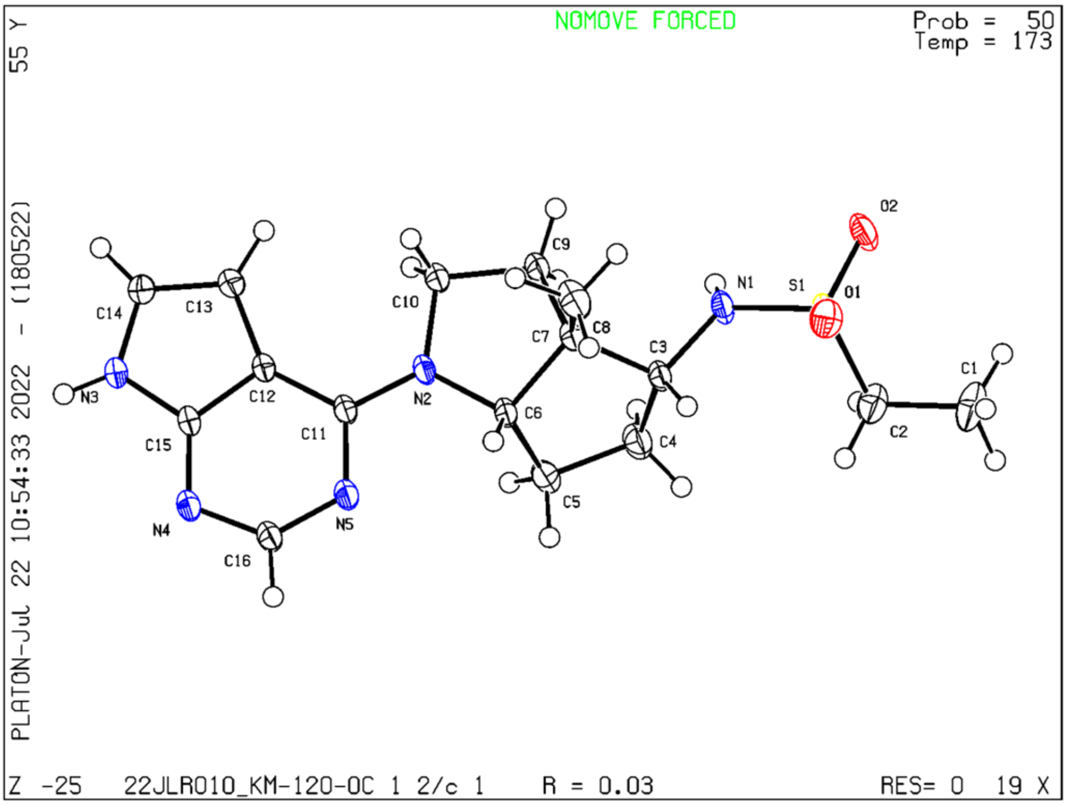

Figure S5. X-ray structure of deprotected **34c** (CCDC 2482164)

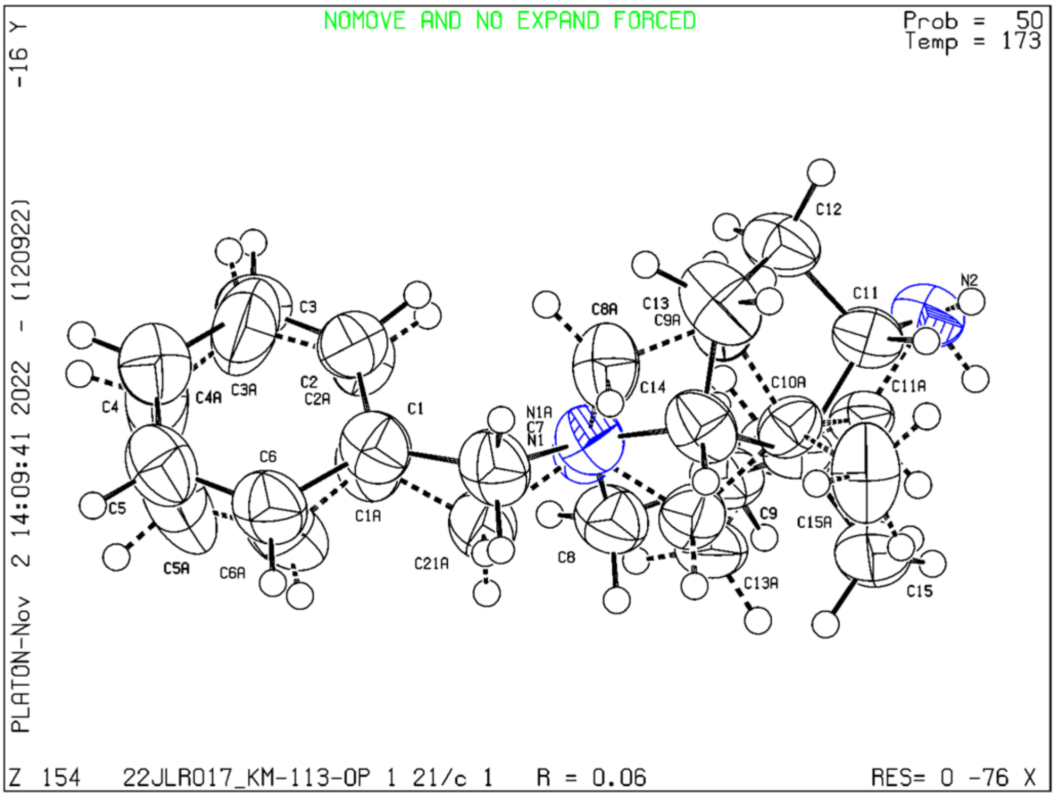

Figure S6. X-ray structure of deprotected Boc-deprotected **32** (CCDC 2482168)

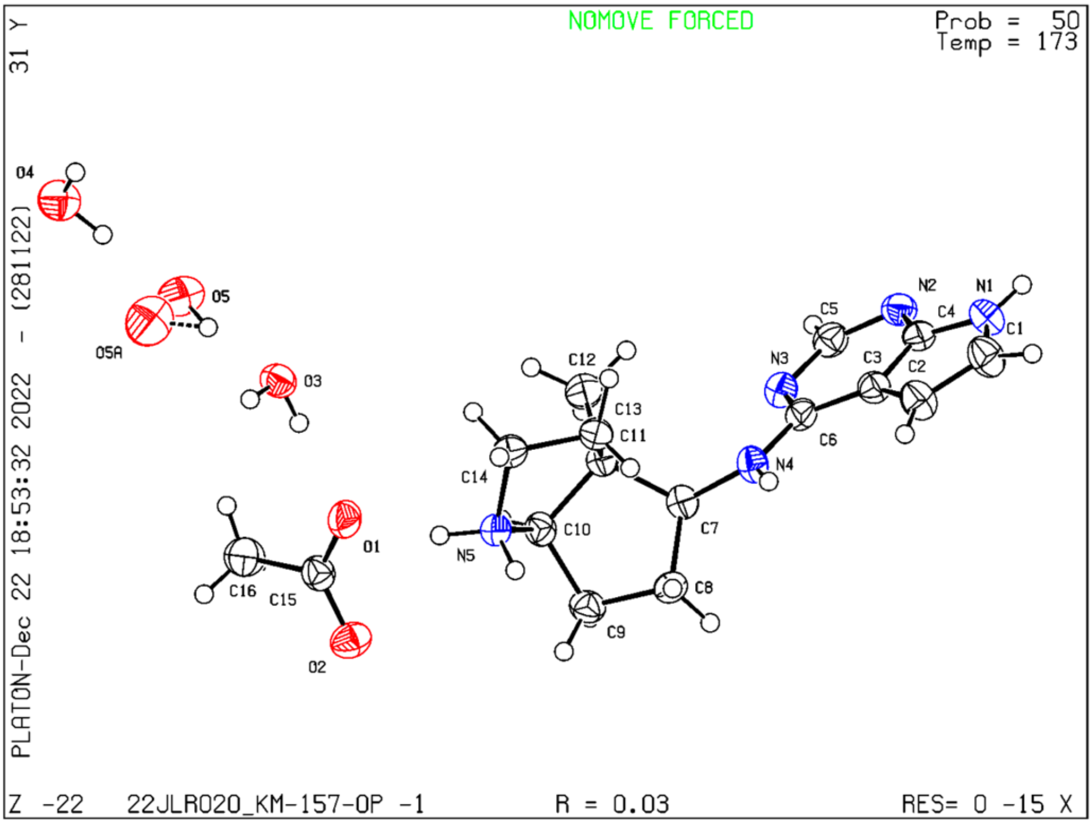

Figure S7. X-ray structure of deprotected **35** (CCDC 2482165)

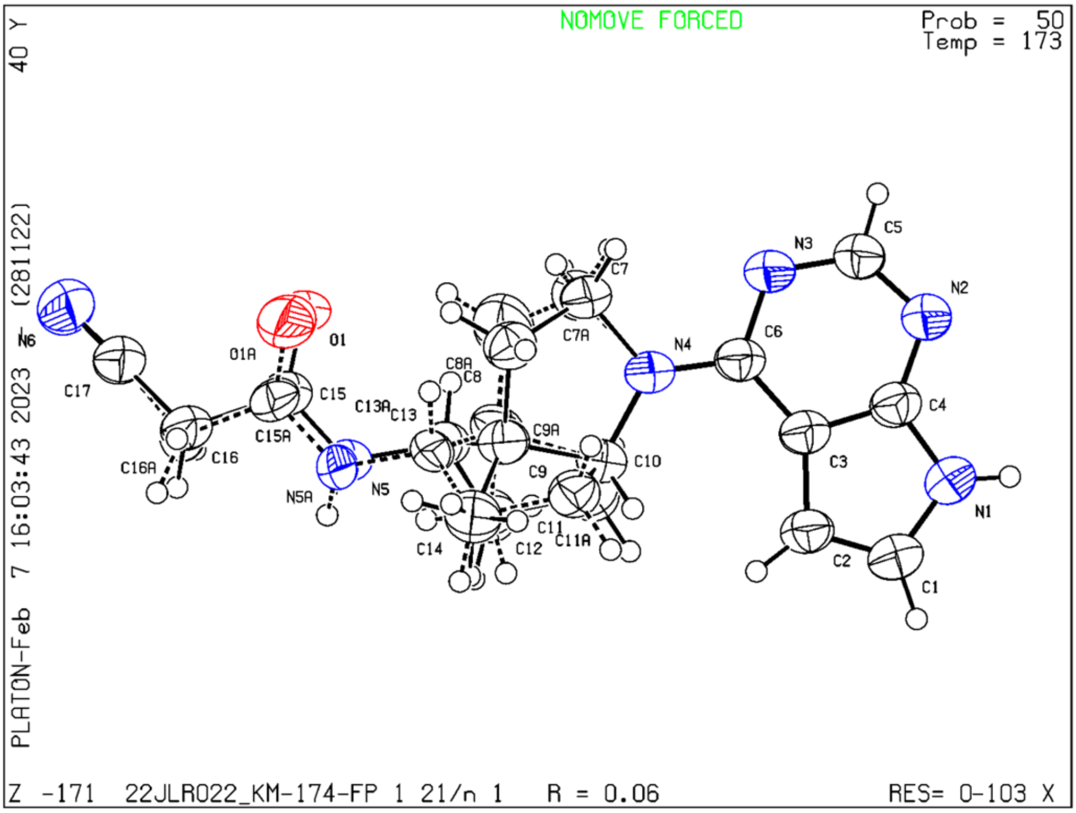

Figure S8. X-ray structure of *rac*-**40a** (CCDC 2482166)

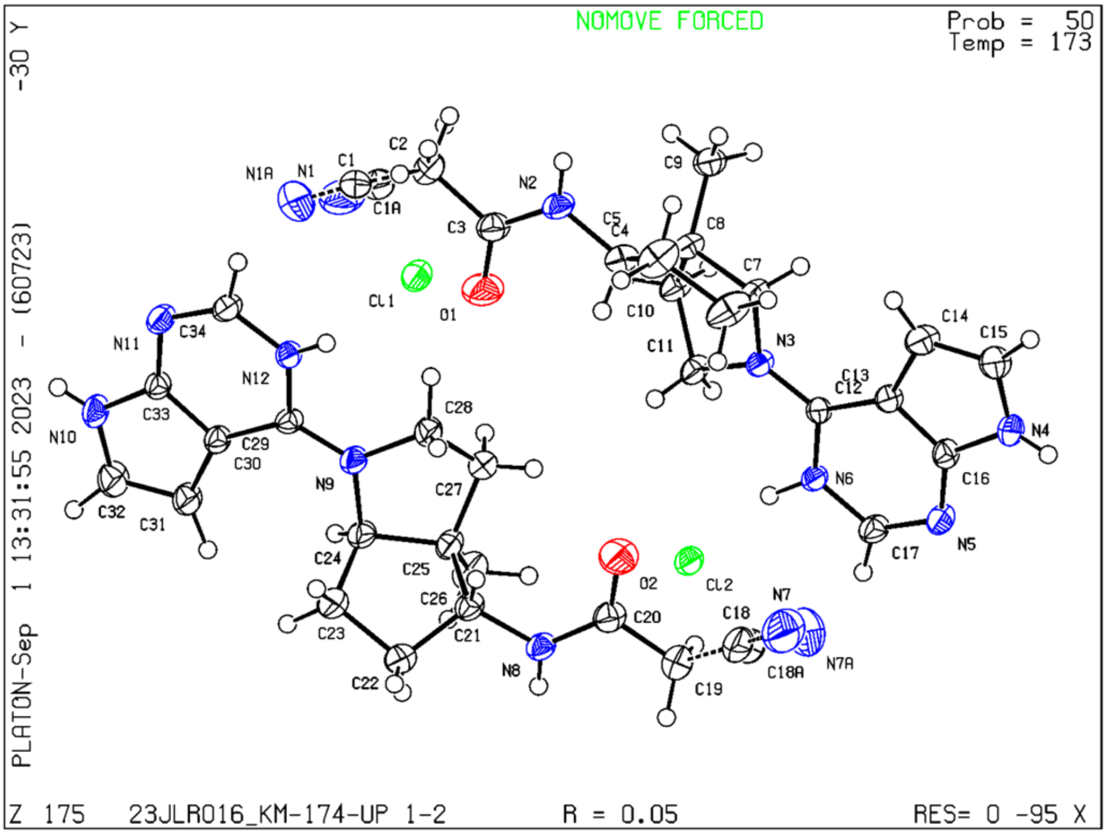

Figure S9. X-ray structure of (*S,R,R*)-**40a** (CCDC 2482167)

Chiral Separation

Compound **40a** was dissolved to 13 mg/mL in 1:2 DCM:MeOH and purified by SFC. Combined fractions of each of (*R,S,S*)-**40a** and (*S,R,R*)-**40a** were then evaporated to near dryness using a rotary evaporator. The resultant solids were then transferred into final vessels with DCM, and the solvent was removed on a Biotage V10 at 35 °C before being stored in a vacuum oven at 35 °C and 5 mbar until constant weight to afford (*R,S,S*)-**40a** as a white solid. Fractions containing (*S,R,R*)-**40a** were combined, concentrated, and repurified and isolated as above to afford the pure compound as a clear film. Conditions: Chiralpak IA column (21mm x 250mm, 5um), column temperature of 40 °C, flow rate 50 mL/min, BPR of 100 BarG, detector wavelength of 210 nm, injection volume of 300uL (3.9 mg), isocratic run 25:75 MeOH:CO_2 (0.2% v/v NH_3).

Compound *rac*-**40a**

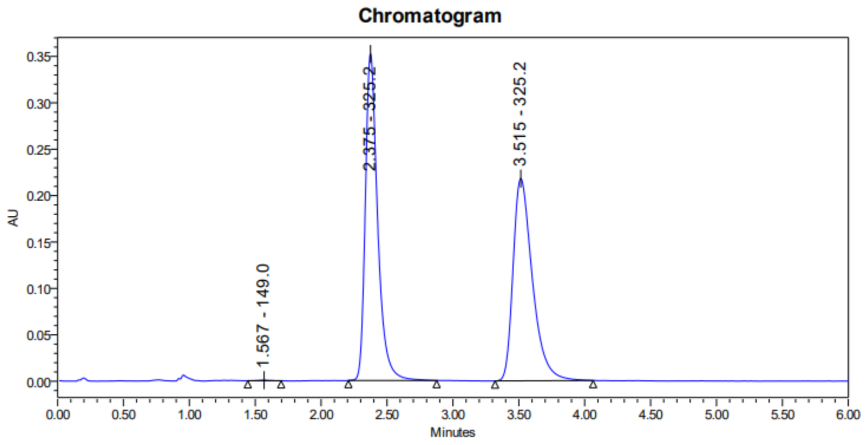

Peak Results

	Retention Time (min)	Area (µV*sec)	% Area	Width @ 50%
1	1.57	4001	0.1	0.09058
2	2.37	2284688	50.7	0.09501
3	3.52	2221520	49.3	0.15226

(*R*,*S*,*S*)-**40a**

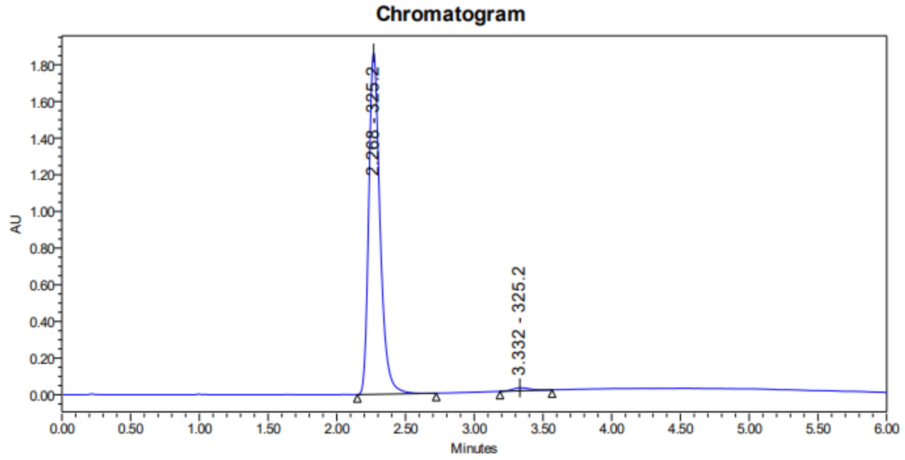

Peak Results

	Retention Time (min)	Area (µV*sec)	% Area	Width @ 50%
1	2.27	10609500	98.6	0.08481
2	3.33	145510	1.4	0.13105

(*S*,*R*,*R*)-**40a**

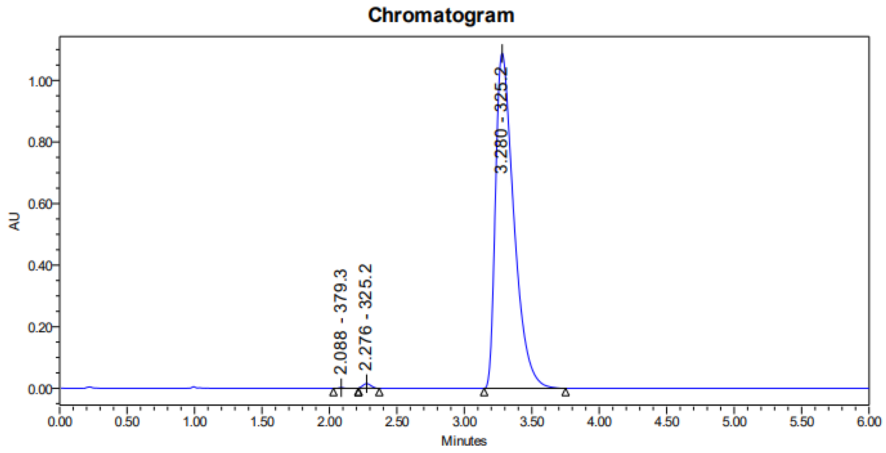

Peak Results

	Retention Time (min)	Area (µV*sec)	% Area	Width @ 50%
1	2.09	4309	0.0	0.03668
2	2.28	60527	0.6	0.06669
3	3.28	10261801	99.4	0.14332

Biochemical assays:

JAK1 inhibition assay: JAK1 (h) is incubated with 20 mM Tris/HCl pH 7.5, 0.2 mM EDTA, 500 µM GEEPLYWSFPAKKK, 10 mM Magnesium acetate, and [γ-33P]-ATP (specific activity and concentration as required). The reaction is initiated by the addition of the Mg/ATP mix. After incubation for 40 minutes at room temperature, the reaction is stopped by the addition of phosphoric acid to a concentration of 0.5%. An aliquot of the reaction is then spotted onto a filter and washed four times for 4 minutes in 0.425% phosphoric acid and once in ethanol prior to drying and scintillation counting.

JAK2 inhibition assay: JAK2 (h) is incubated with 8 mM MOPS pH 7.0, 0.2 mM EDTA, 100 µM KTFCGTPEYLAPEVRREPRILSEEEQEMFRDFDYIADWC, 10 mM Magnesium acetate and [γ-33P]-ATP (specific activity and concentration as required). The reaction is initiated by the addition of the Mg/ATP mix. After incubation for 40 minutes at room temperature, the reaction is stopped by the addition of phosphoric acid to a concentration of 0.5%. An aliquot of the reaction is then spotted onto a filter and washed four times for 4 minutes in 0.425% phosphoric acid and once in ethanol prior to drying and scintillation counting.

JAK3 inhibition assay: JAK3 (h) is incubated with 8 mM MOPS pH 7.0, 0.2 mM EDTA, 500 µM GGEEEEYFELVKKKK, 10 mM Magnesium acetate and [γ-33P]-ATP (specific activity and concentration as required). The reaction is initiated by the addition of the Mg/ATP mix. After incubation for 40 minutes at room temperature, the reaction is stopped by the addition of phosphoric acid to a concentration of 0.5%. An aliquot of the reaction is then spotted onto a filter and washed four times for 4 minutes in 0.425% phosphoric acid and once in ethanol prior to drying and scintillation counting.

TYK2 inhibition assay: TYK2 (h) is incubated with 8 mM MOPS pH 7.0, 0.2 mM EDTA, 250 µM GGMEDIYFEFMGGKKK, 10 mM Magnesium acetate, and [γ-33P]-ATP (specific activity and concentration as required). The reaction is initiated by

the addition of the Mg/ATP mix. After incubation for 40 minutes at room temperature, the reaction is stopped by the addition of phosphoric acid to a concentration of 0.5%. An aliquot of the reaction is then spotted onto a filter and washed four times for 4 minutes in 0.425% phosphoric acid and once in ethanol prior to drying and scintillation counting.

Janus kinase inhibition complementary data

Table S2. Kinase inhibition of **36d** and **40a-d** at higher concentrations.

		% of inhibition			
**Entry**	**Conc. (μM)**	**JAK1**	**JAK2**	**JAK3**	**TYK2**
**36d**	1	45	11	44	53
**40a**	10	99	98	96	97
	1	99	72	79	82
**40b**	10	99	91	89	95
	1	93	52	55	70
**40c**	10	100	96	99	100
	1	95	58	96	92
**40d**	10	100	85	79	91
	1	76	38	11	44

Docking score results

Table S3. Docking Score for compound (*R*)-**KMC420**, (*RSS*)-**40a**, and (*SRR*)-**40a**.

	(*R*)-**KMC420**	(*RSS*)-**40a**	(*SRR*)-**40a**
Grid Score	-60.214027	-64.467567	-60.248623
Grid van der Waals energy	-58.195213	-62.913582	-58.540413
Grid electrostatic energy	-2.018813	-1.553985	-1.708211
Internal energy repulsive	14.861233	12.444166	21.912323

NMR data

Compound 7a

^{1}H-NMR

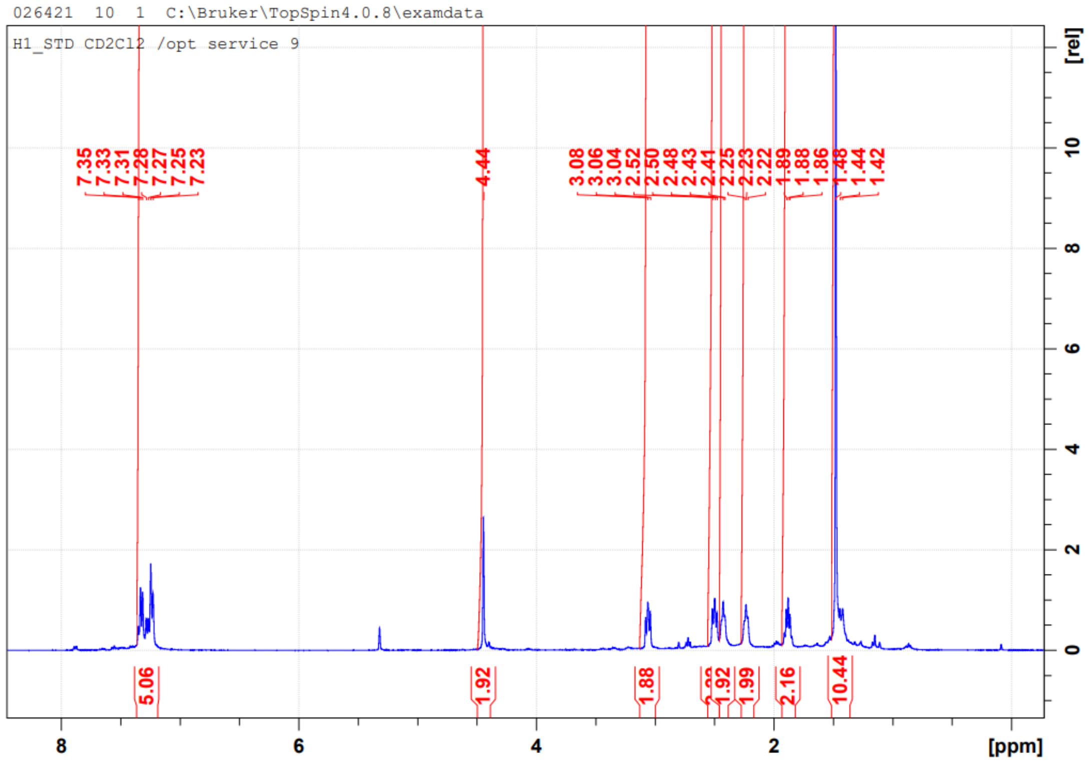

^{13}C-NMR

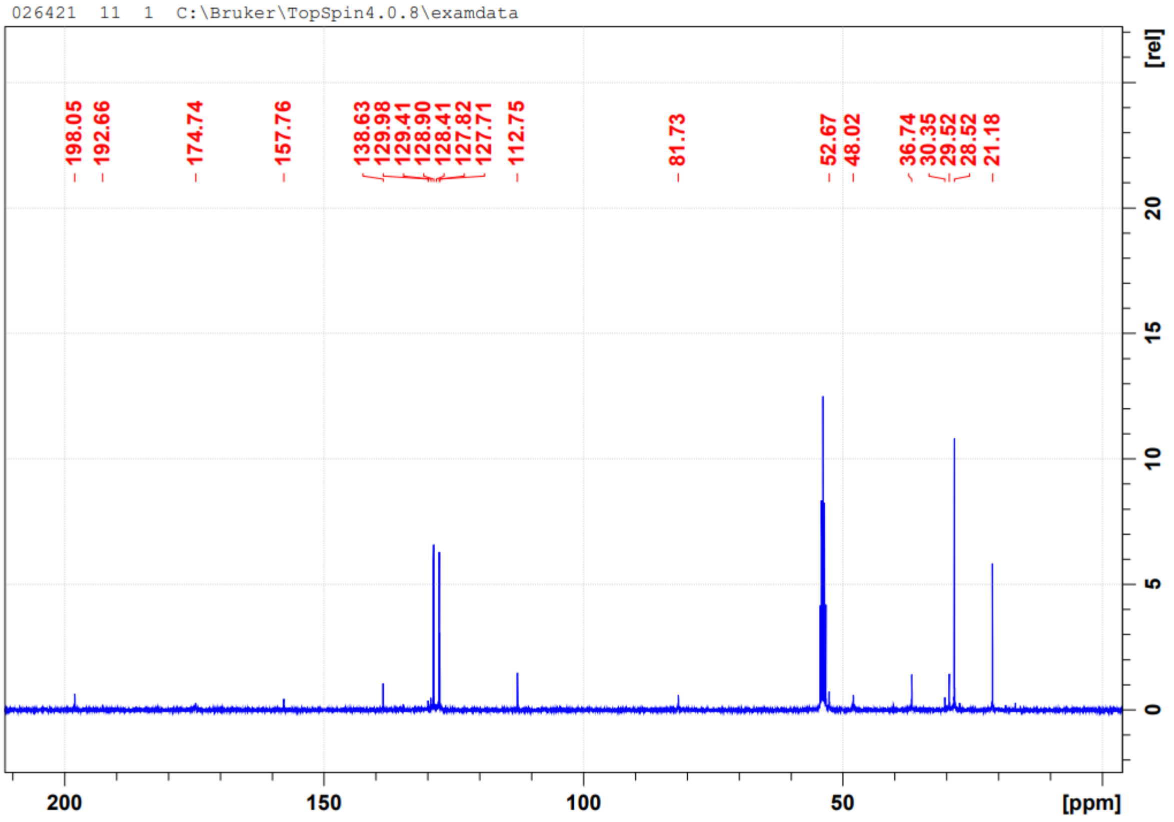

Compound 8a

¹H-NMR

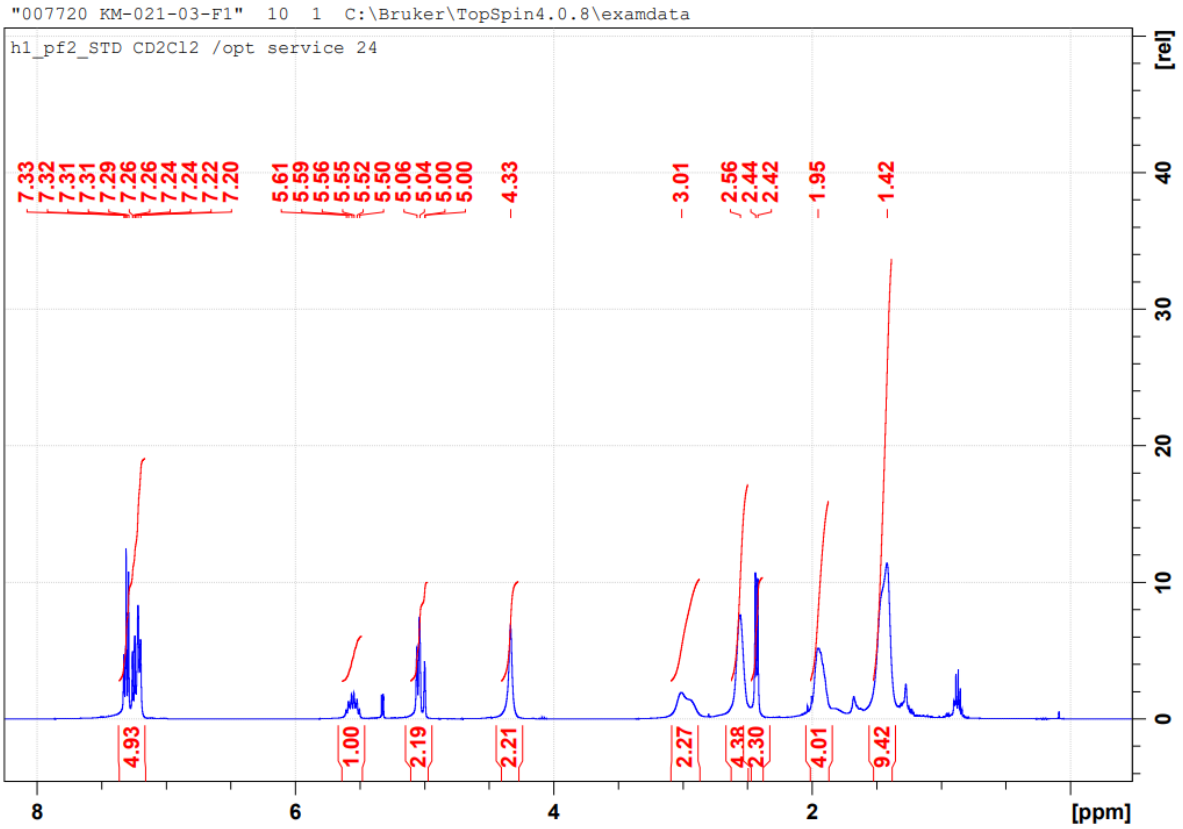

¹³C-NMR

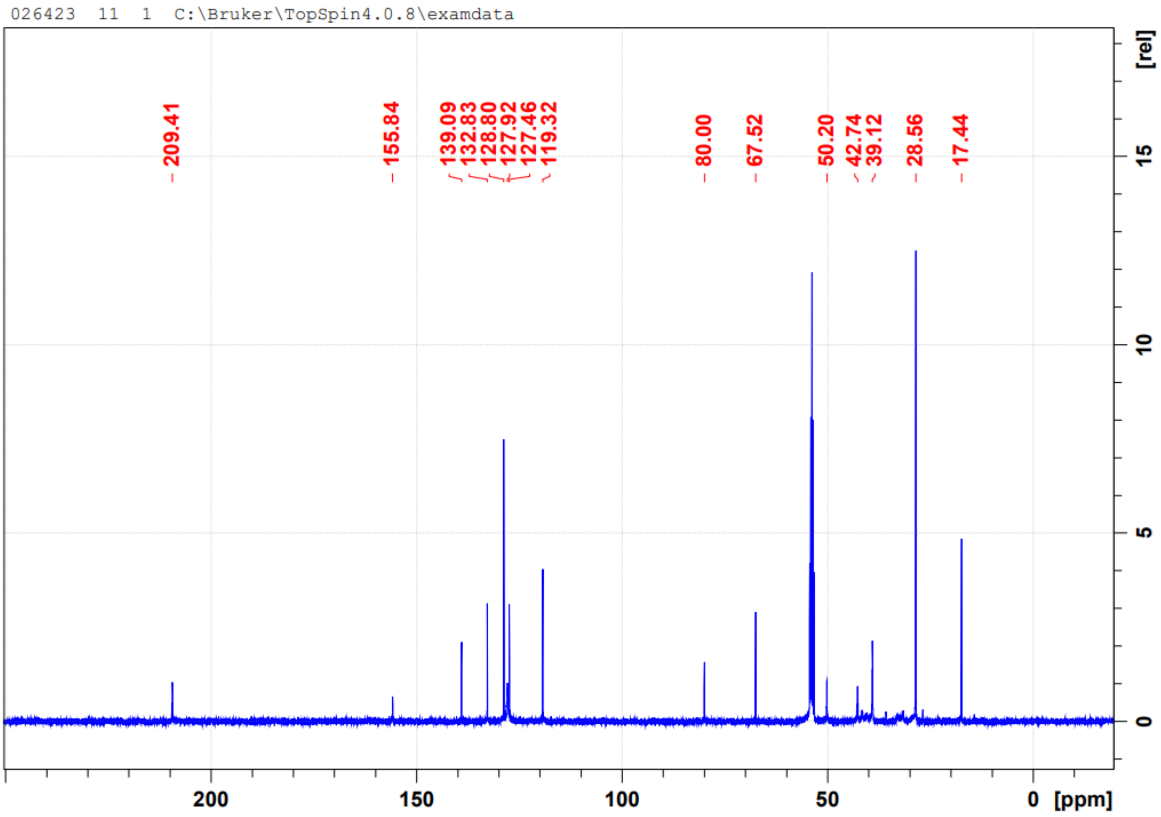

Compound 10a

^{1}H-NMR

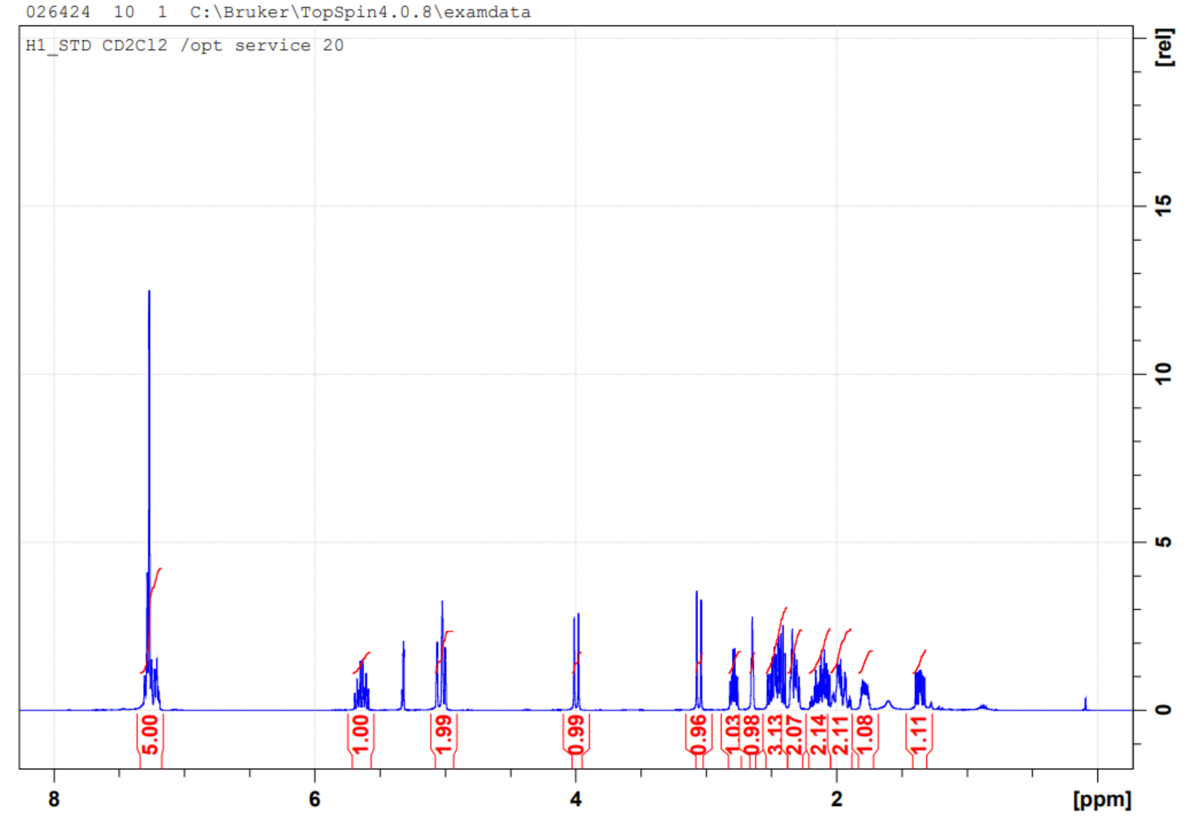

^{13}C-NMR

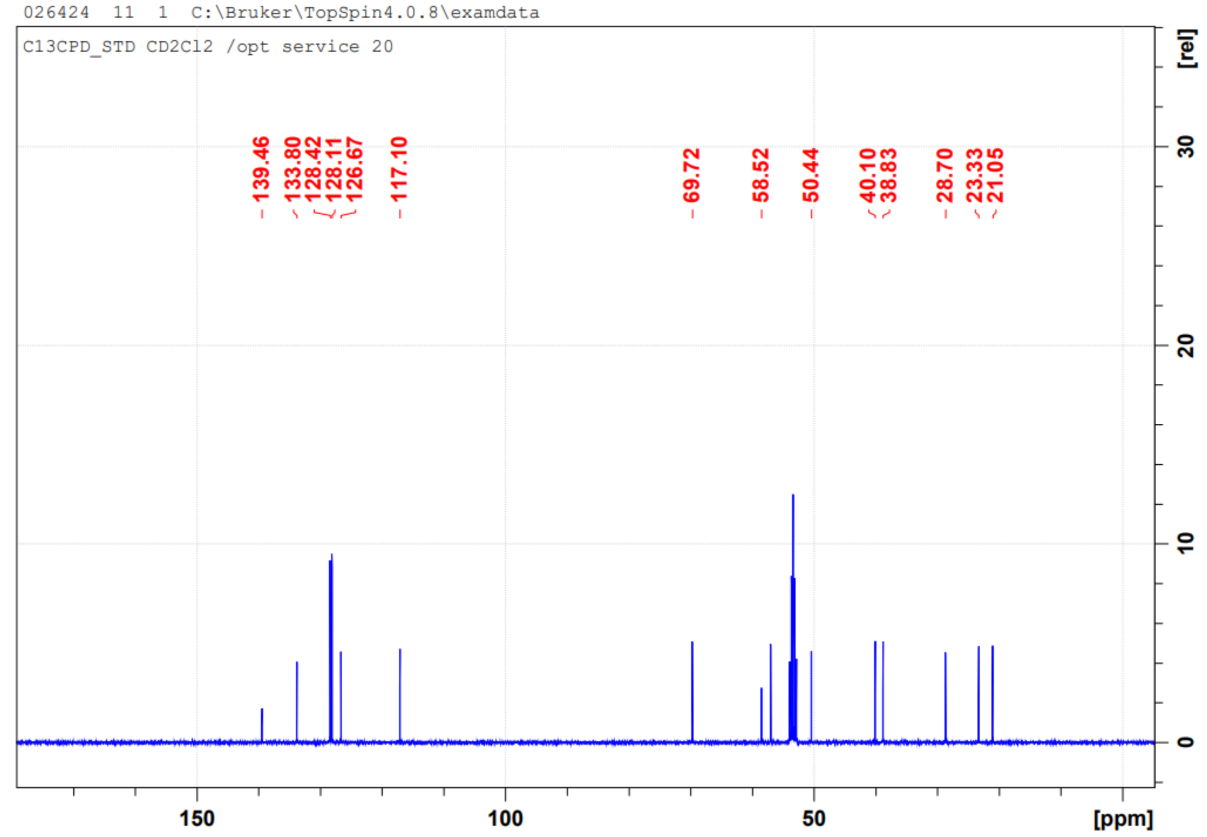

Compound (11a)
^{1}H-NMR

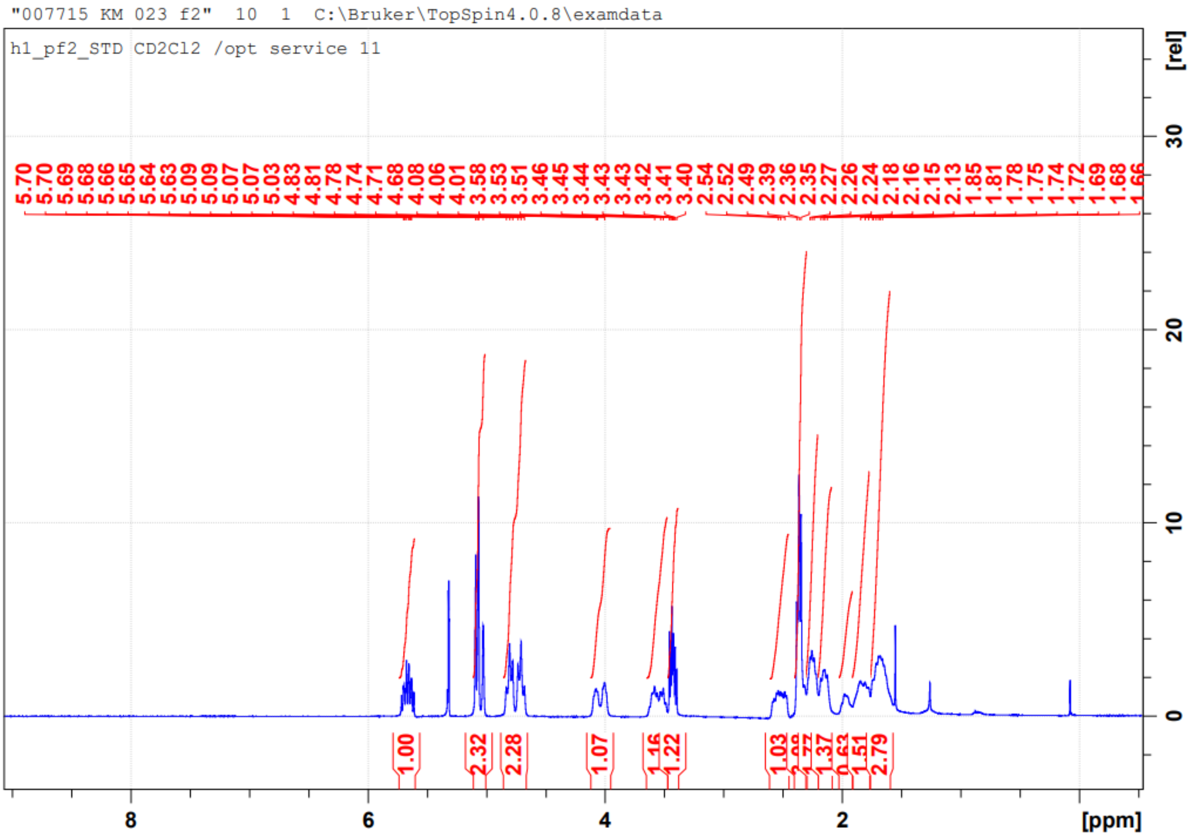

^{13}C-NMR

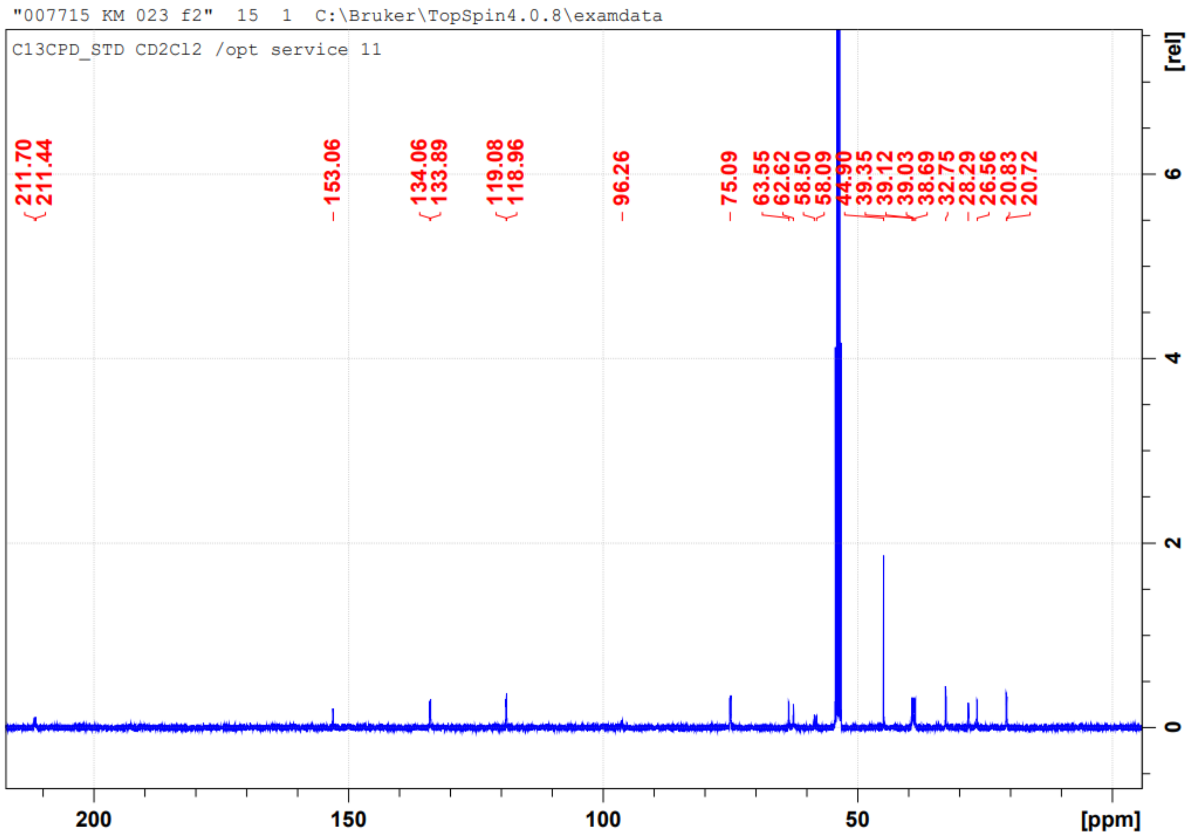

Compound (12a)

^{1}H-NMR

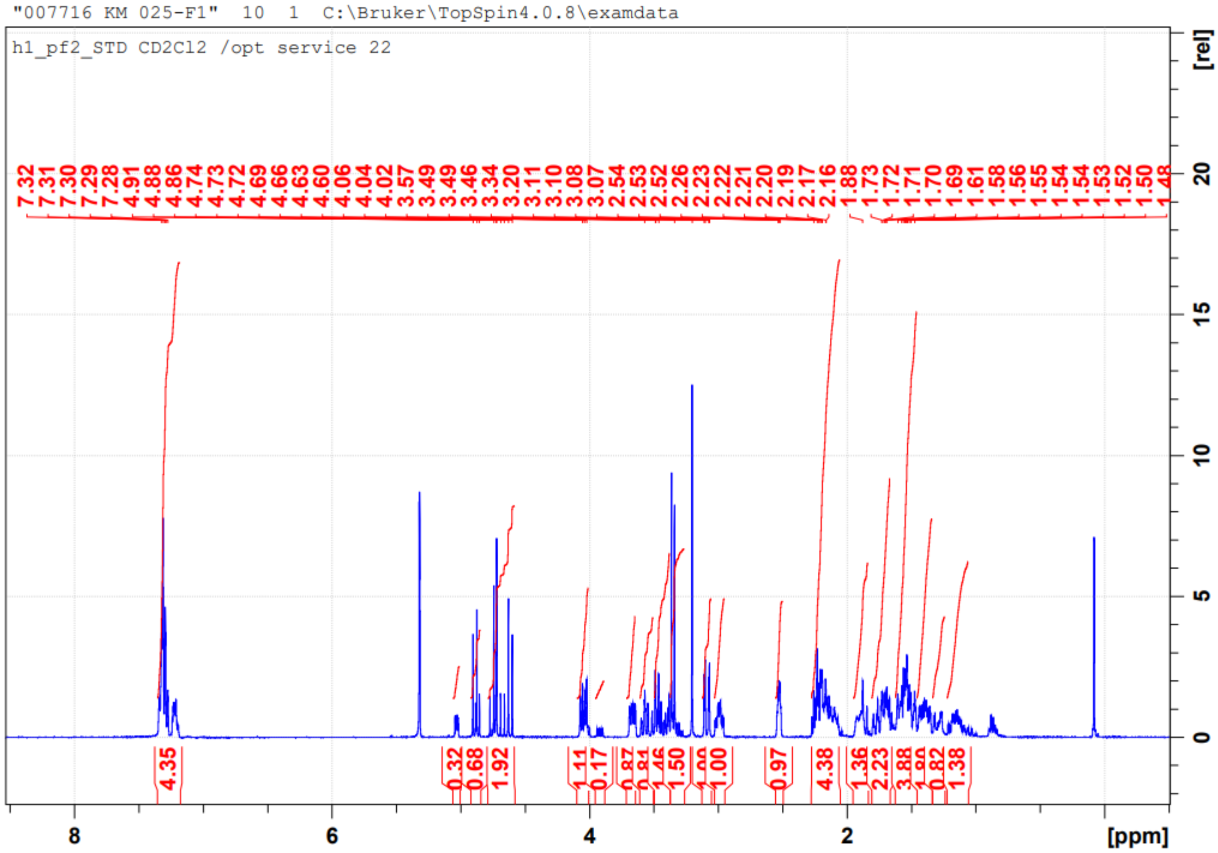

^{13}C-NMR

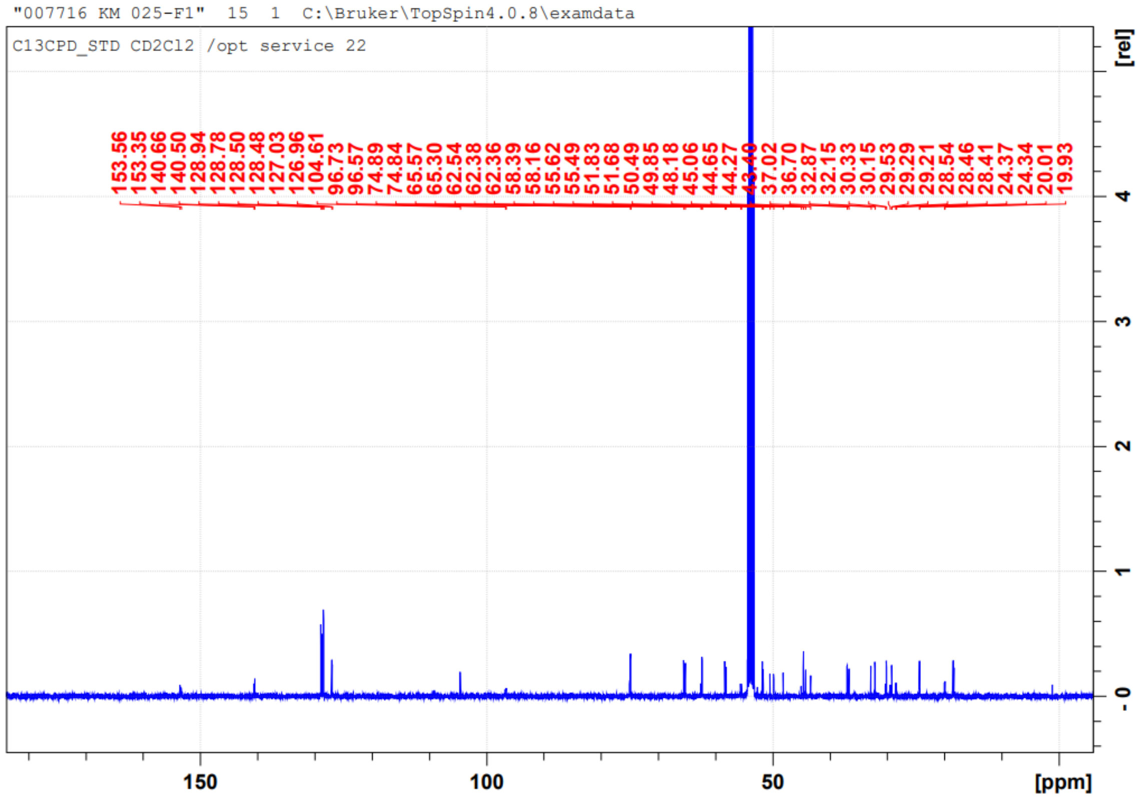

Compound (13a)

^{1}H-NMR

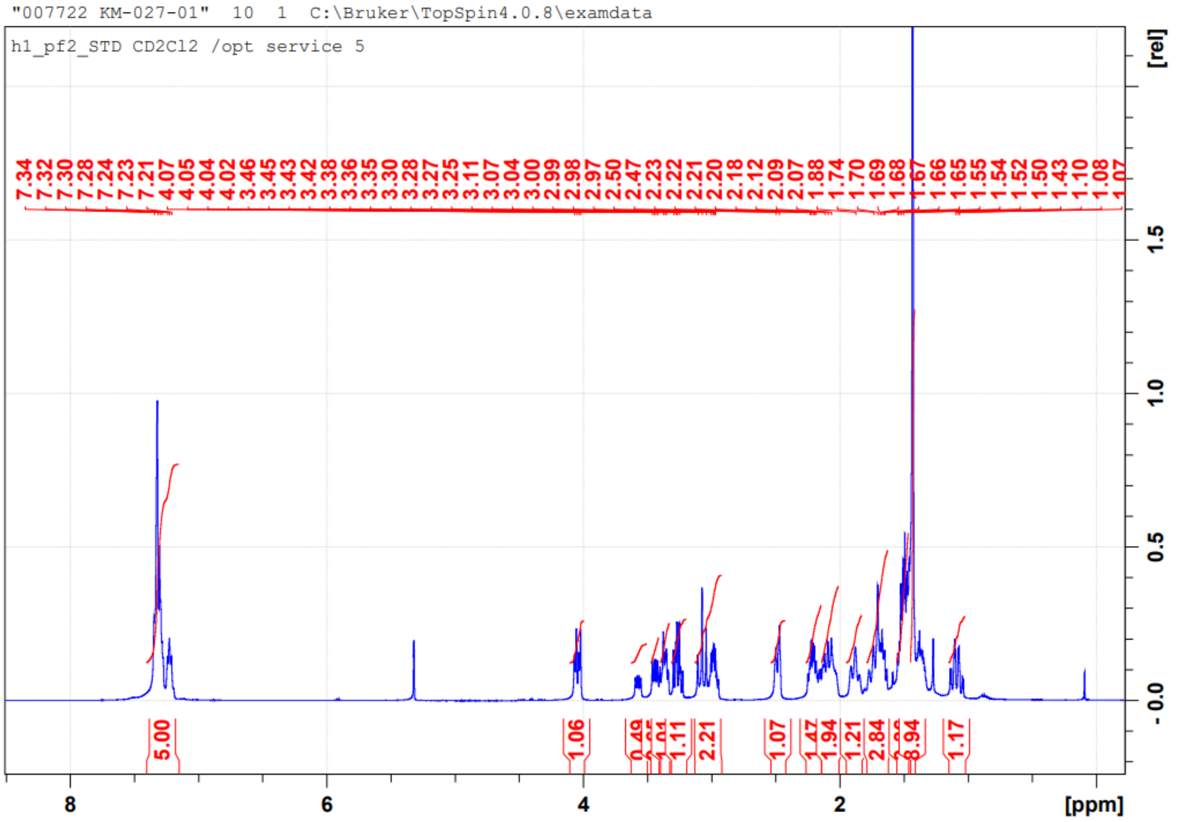

^{13}C-NMR

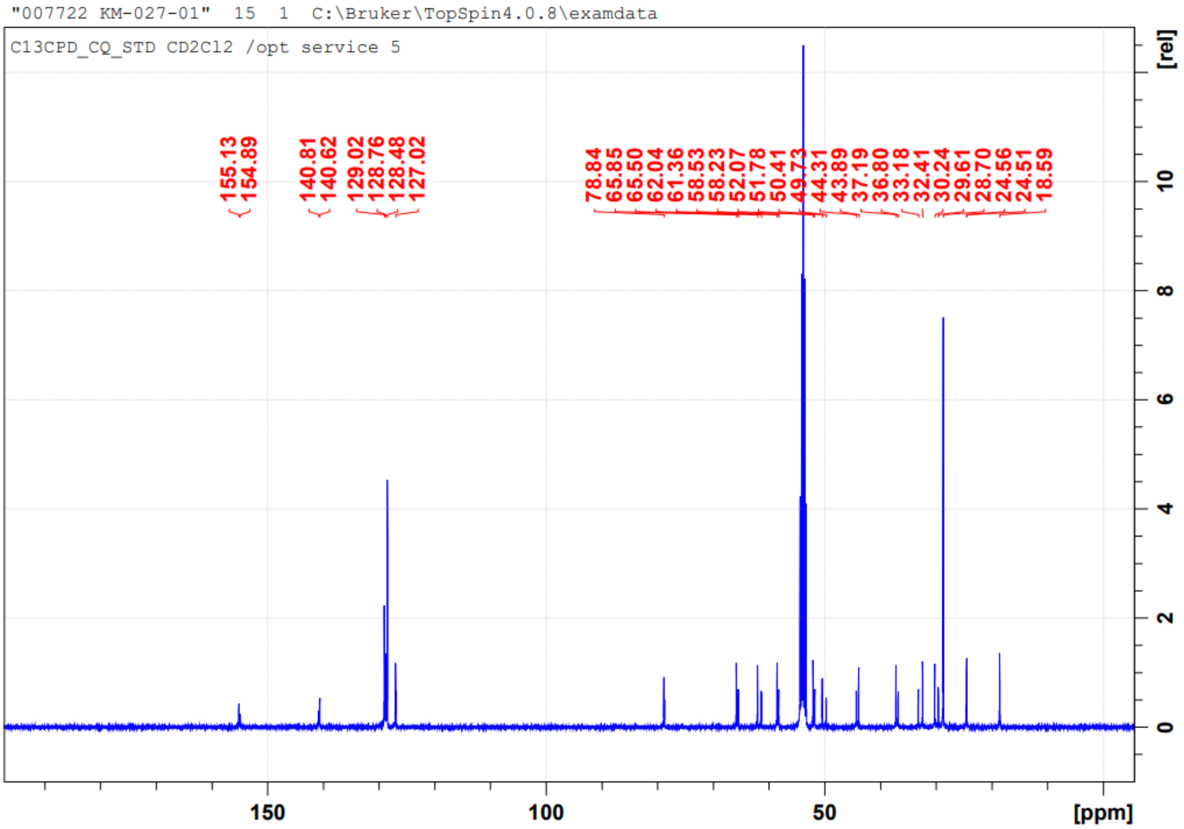

Compound (13b)

^{1}H-NMR

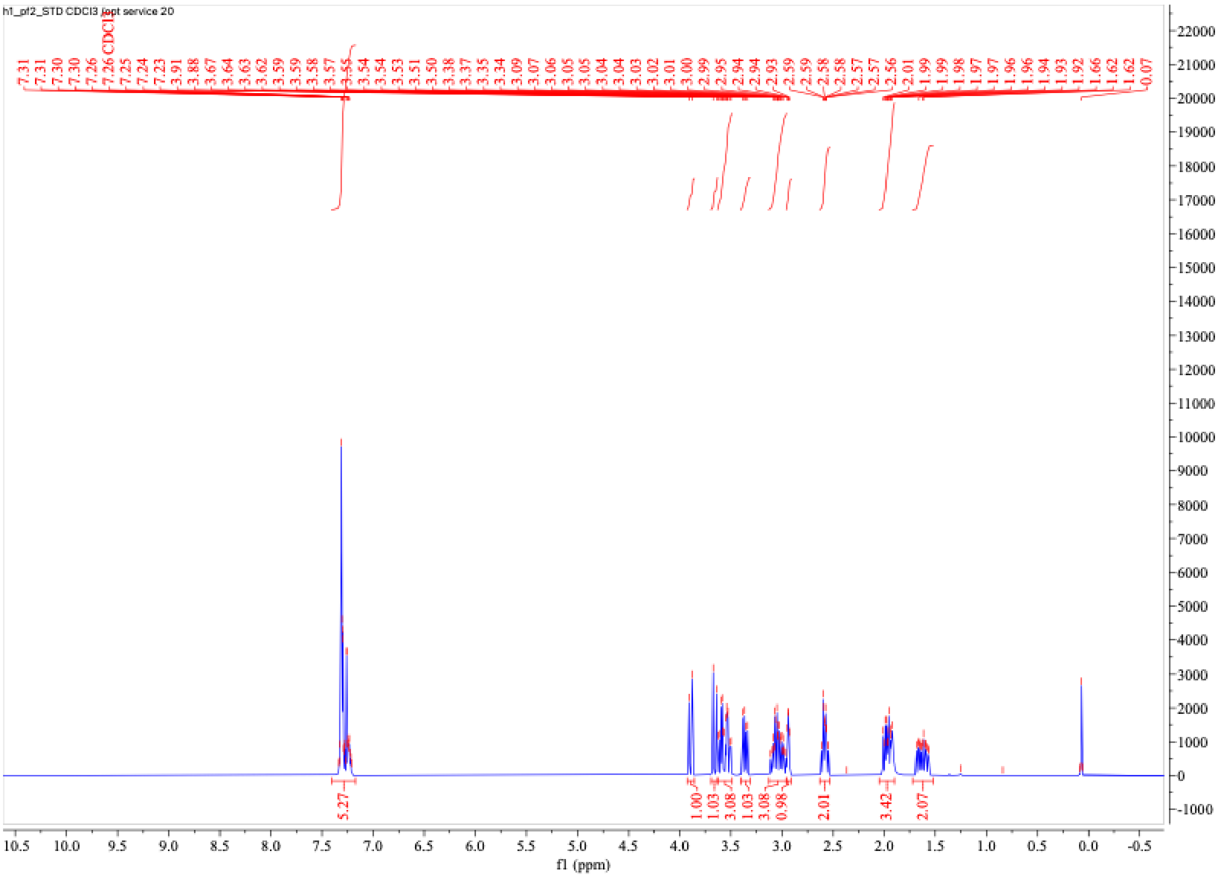

^{13}C-NMR

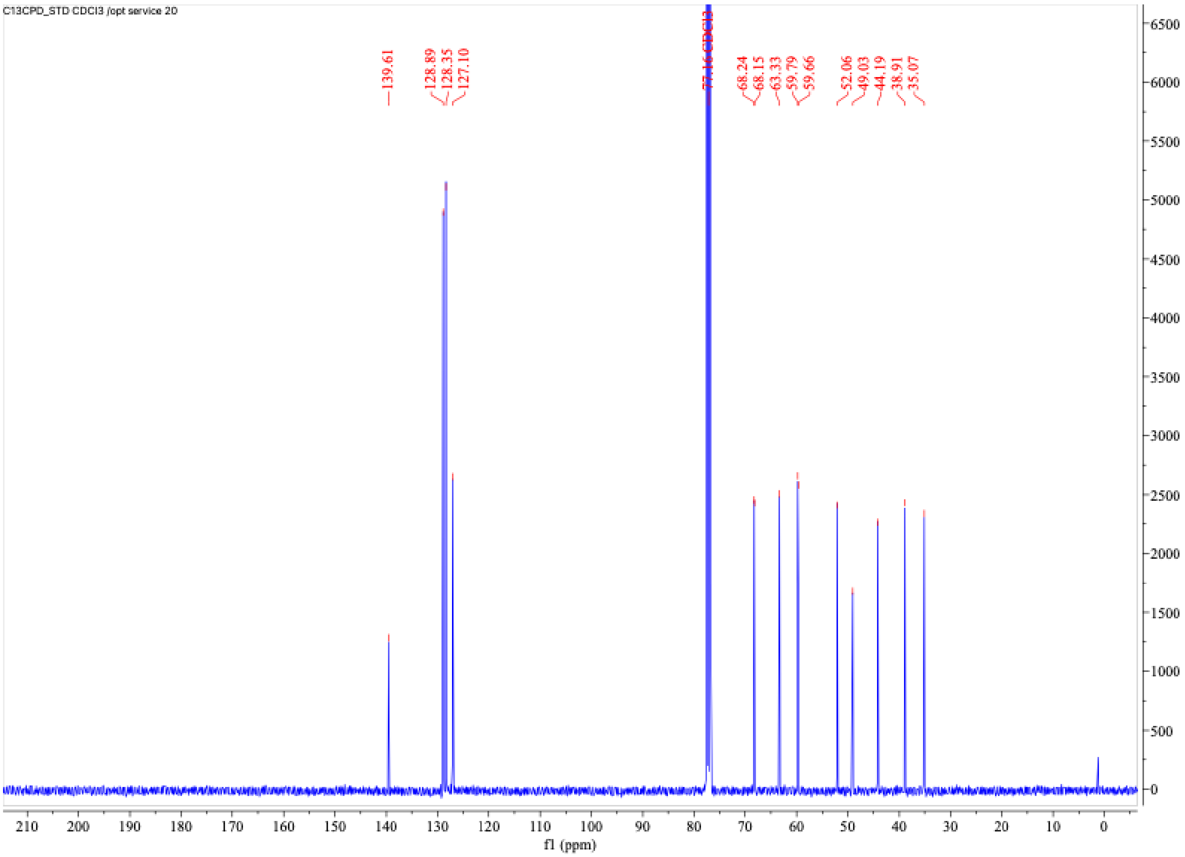

Compound (14)
^{1}H-NMR

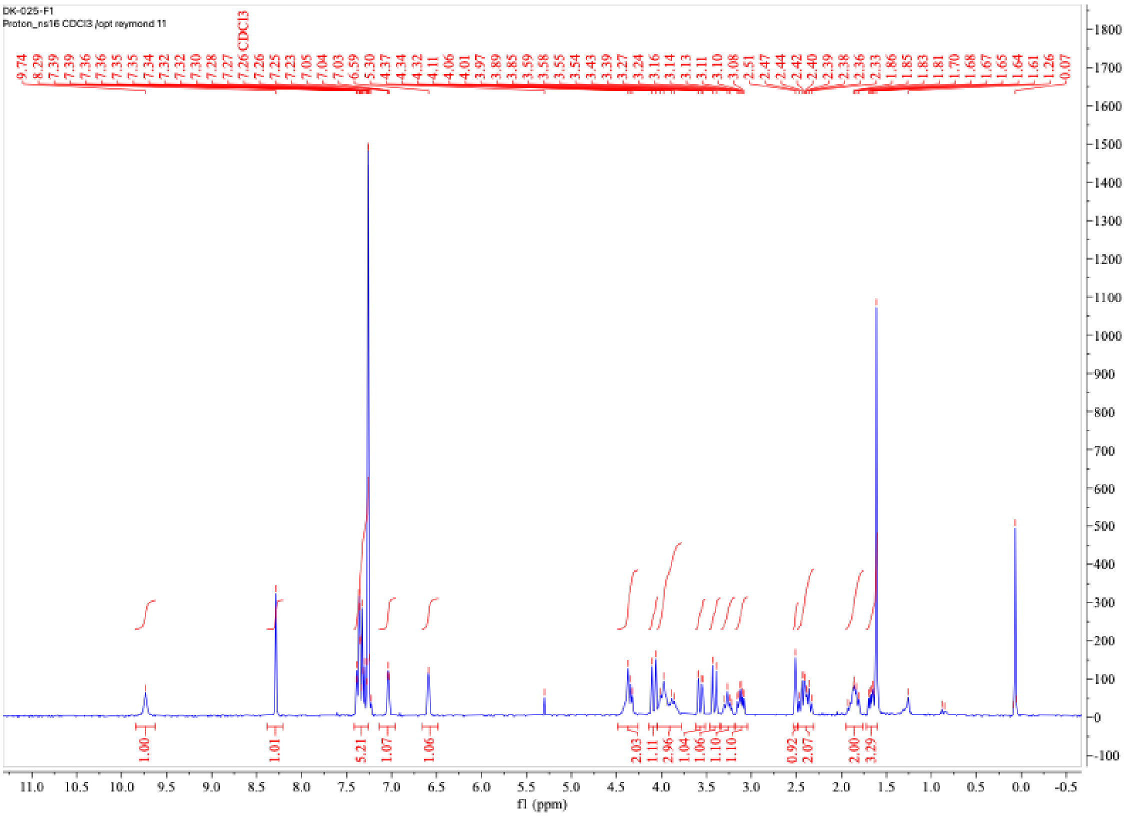

Compound (15)

^{1}H-NMR

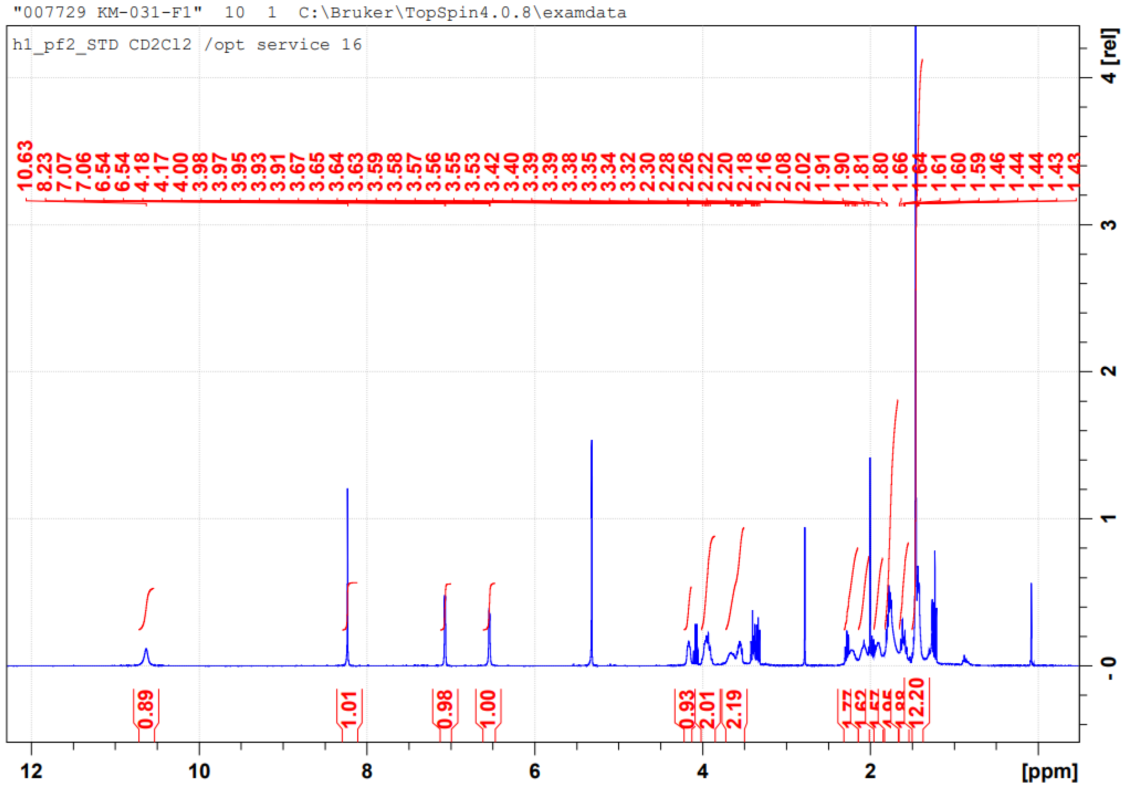

^{13}C-NMR

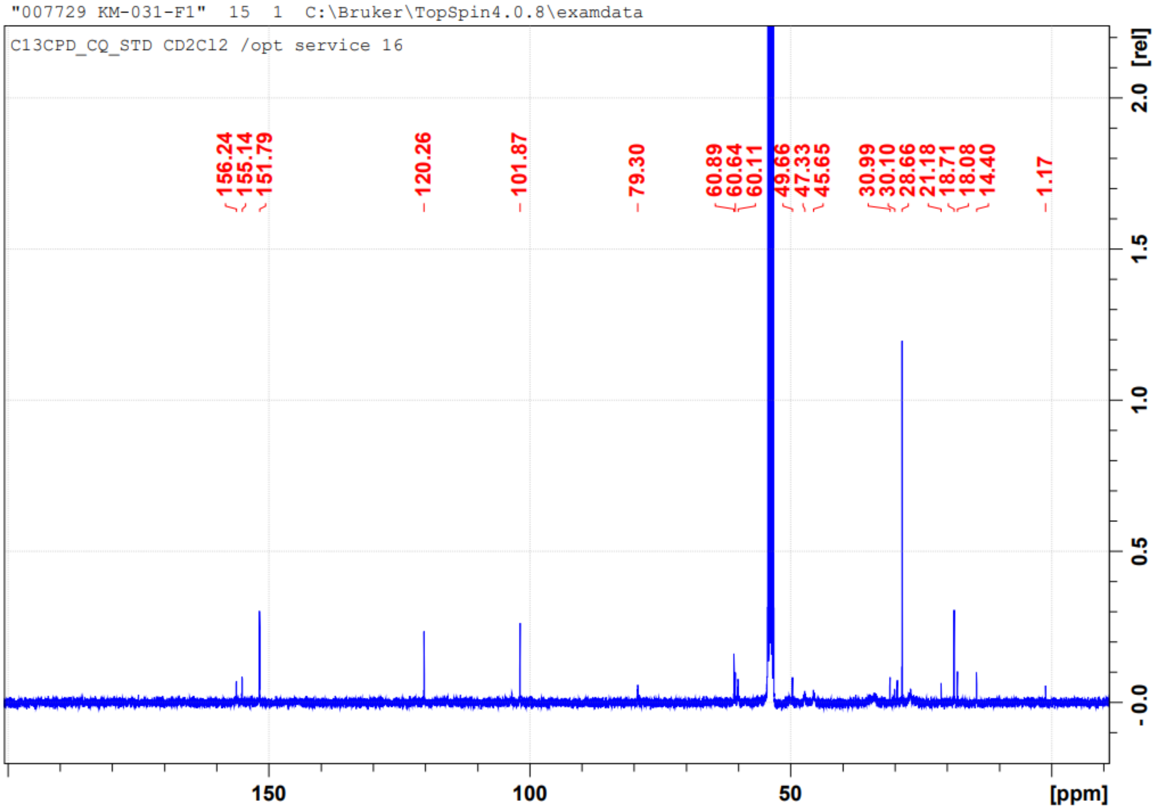

Compound (16a)

^{1}H-NMR

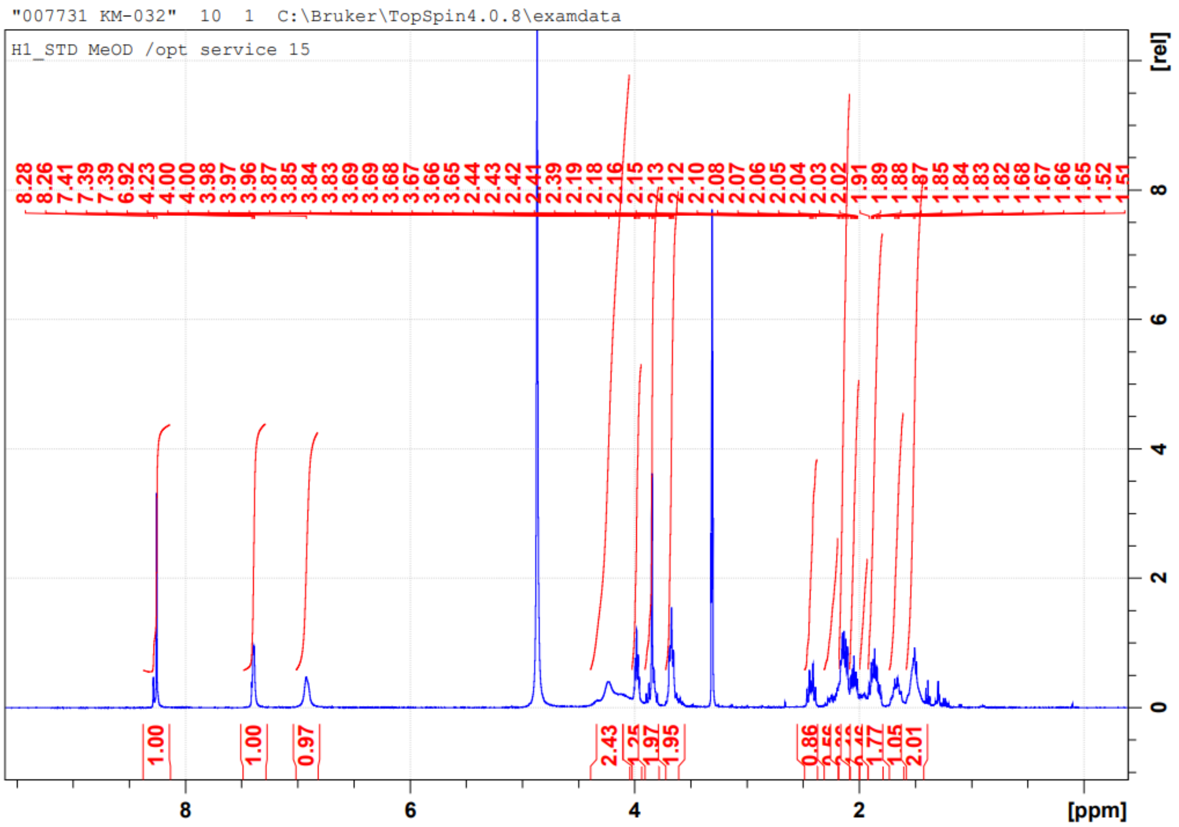

^{13}C-NMR

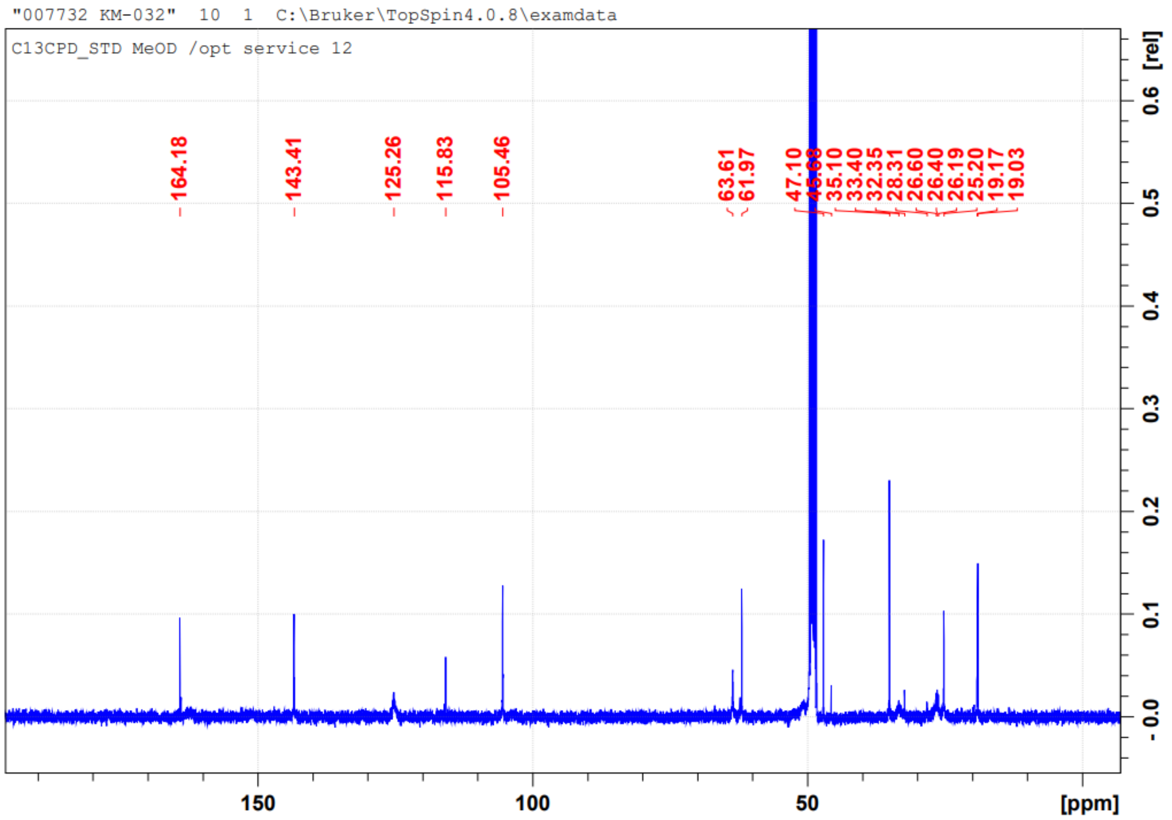

Compound (16b)

^{1}H-NMR

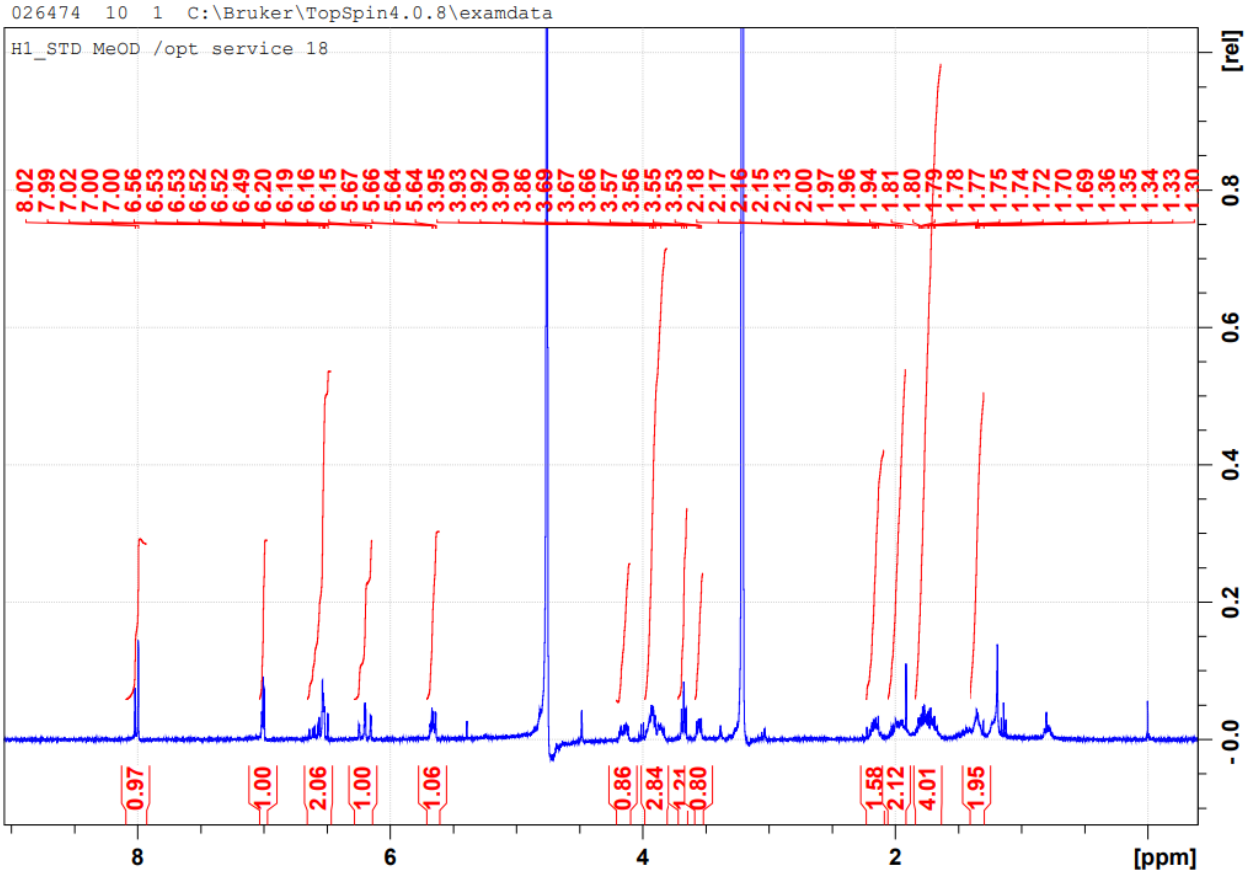

^{13}C-NMR

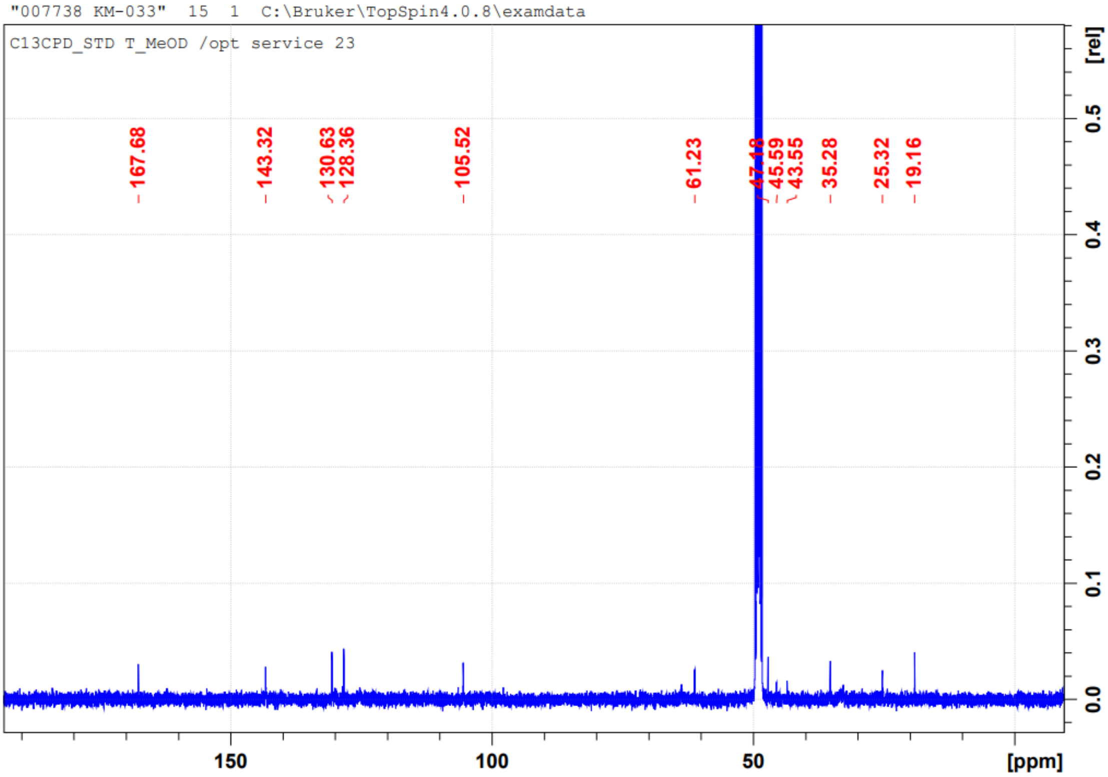

Compound 7b

^{1}H-NMR

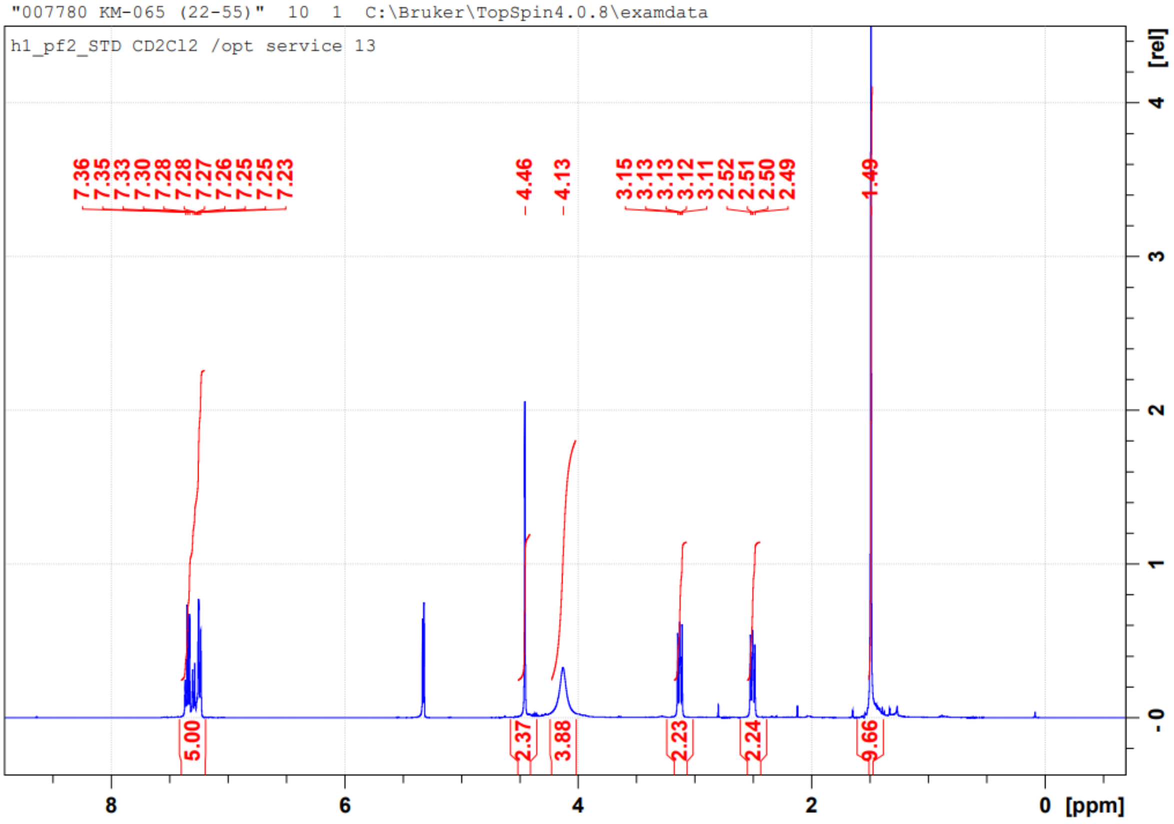

^{13}C-NMR

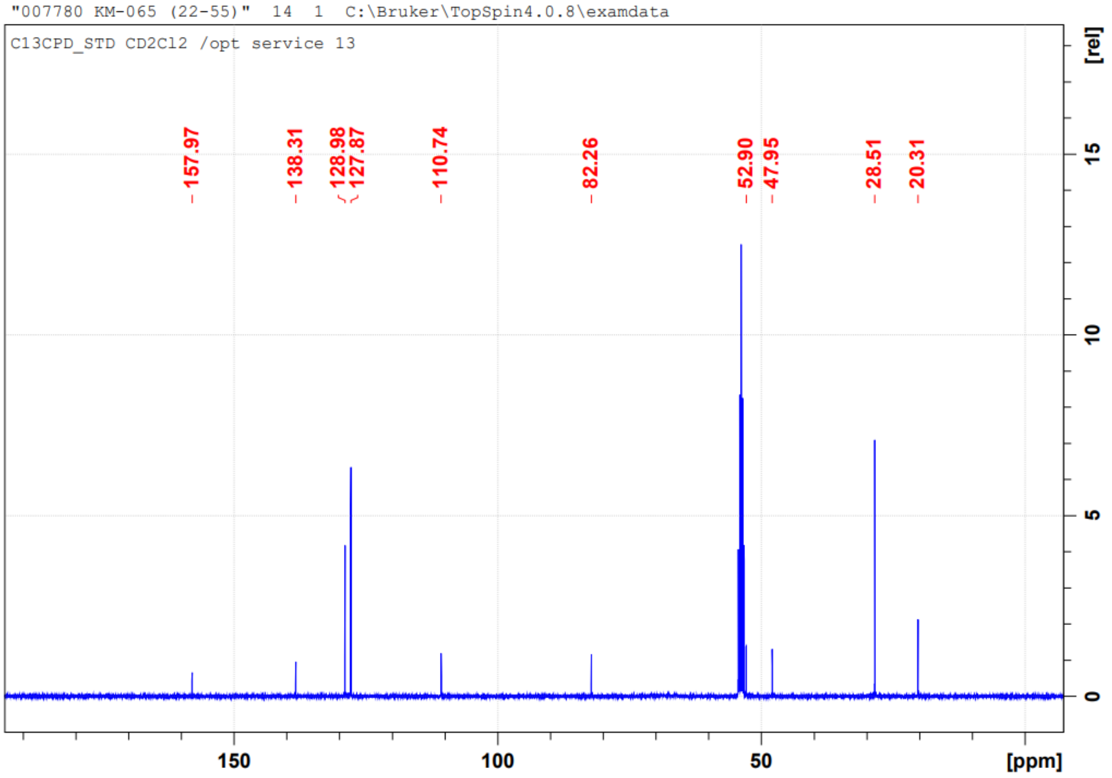

Compound 8b

^{1}H-NMR

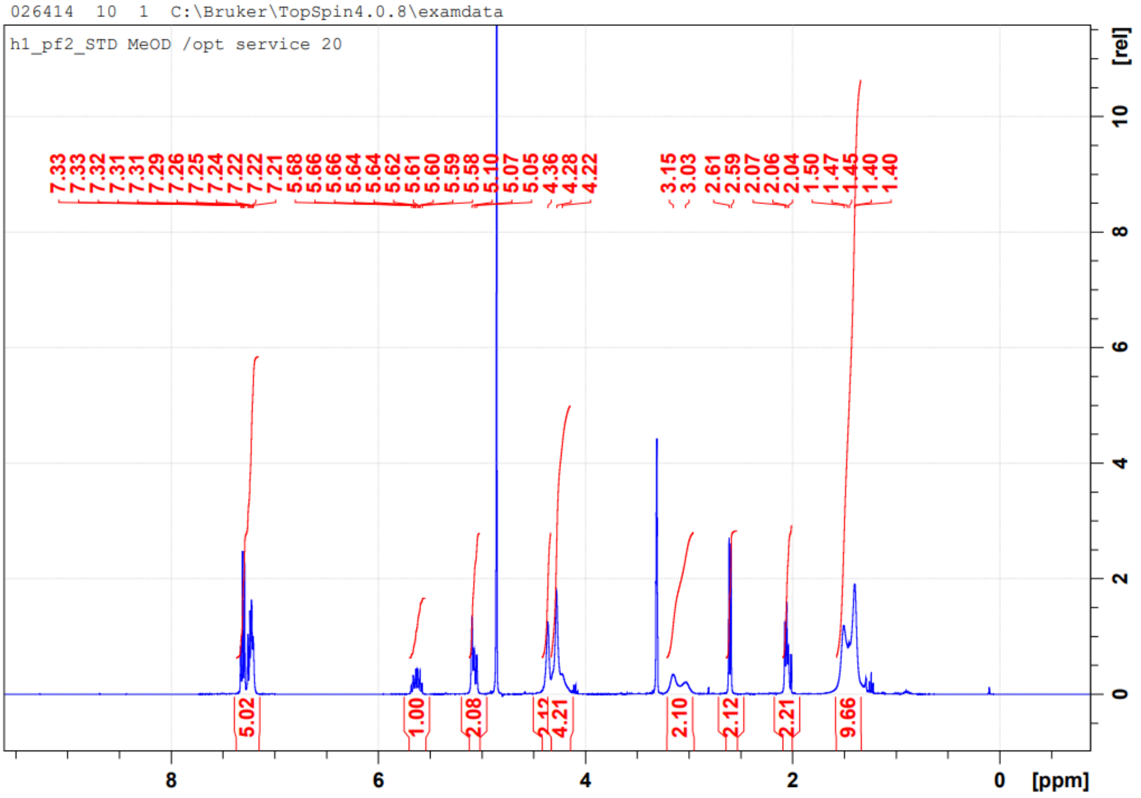

^{13}C-NMR

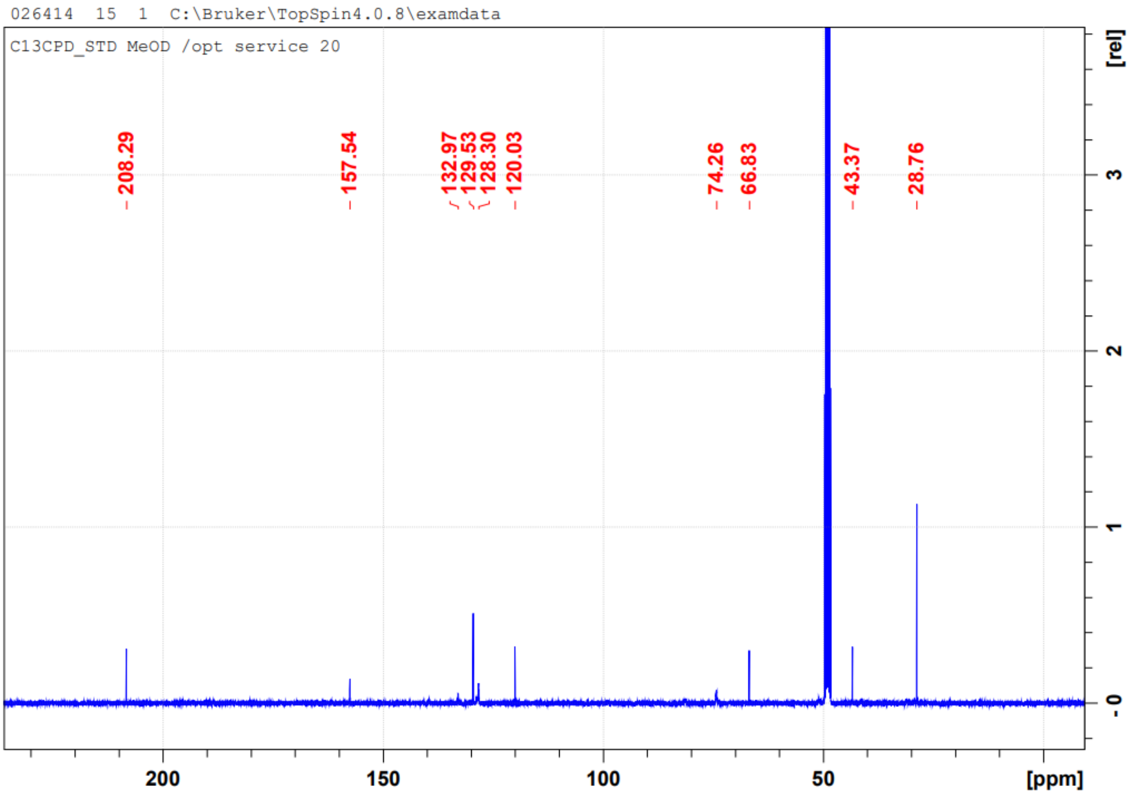

Compound 9b

^{1}H-NMR

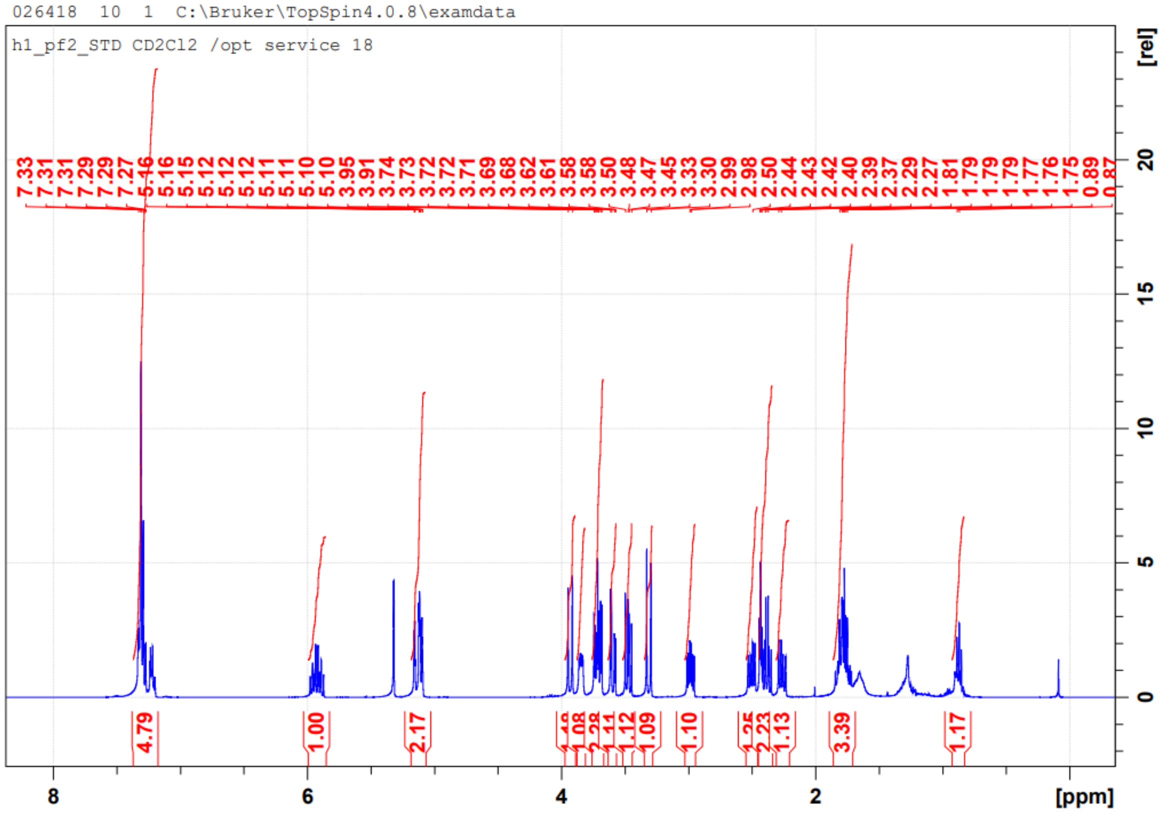

^{13}C-NMR

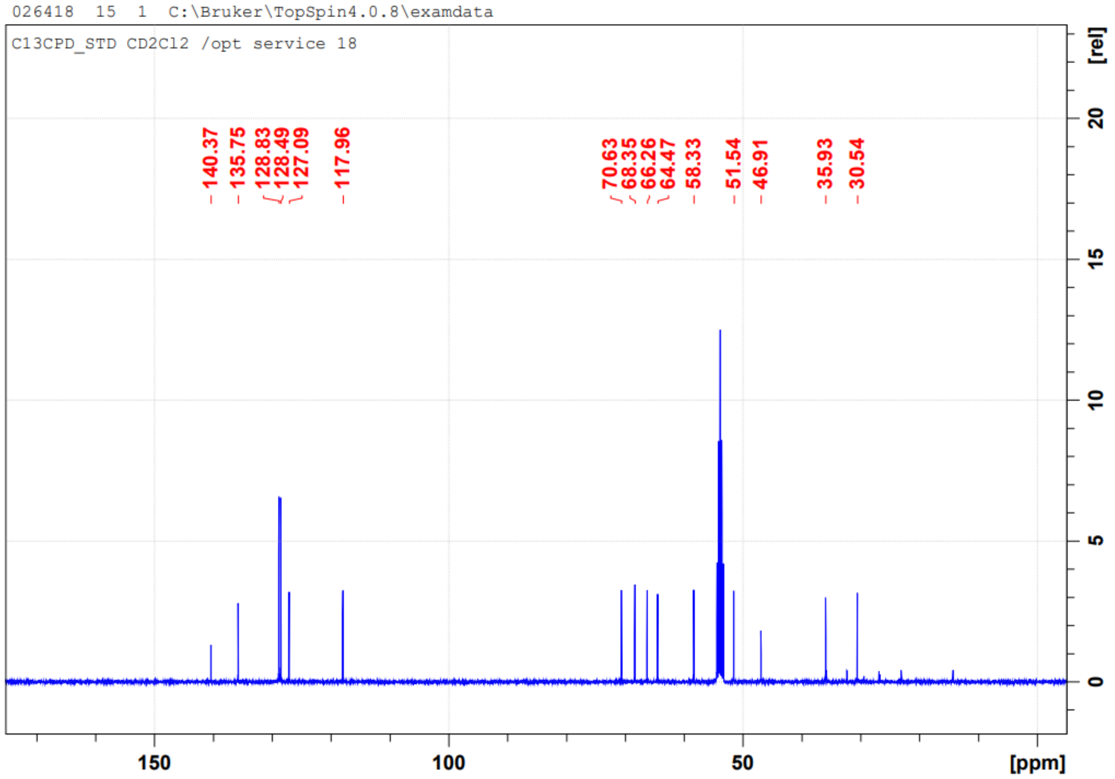

Compound 10b

^{1}H-NMR

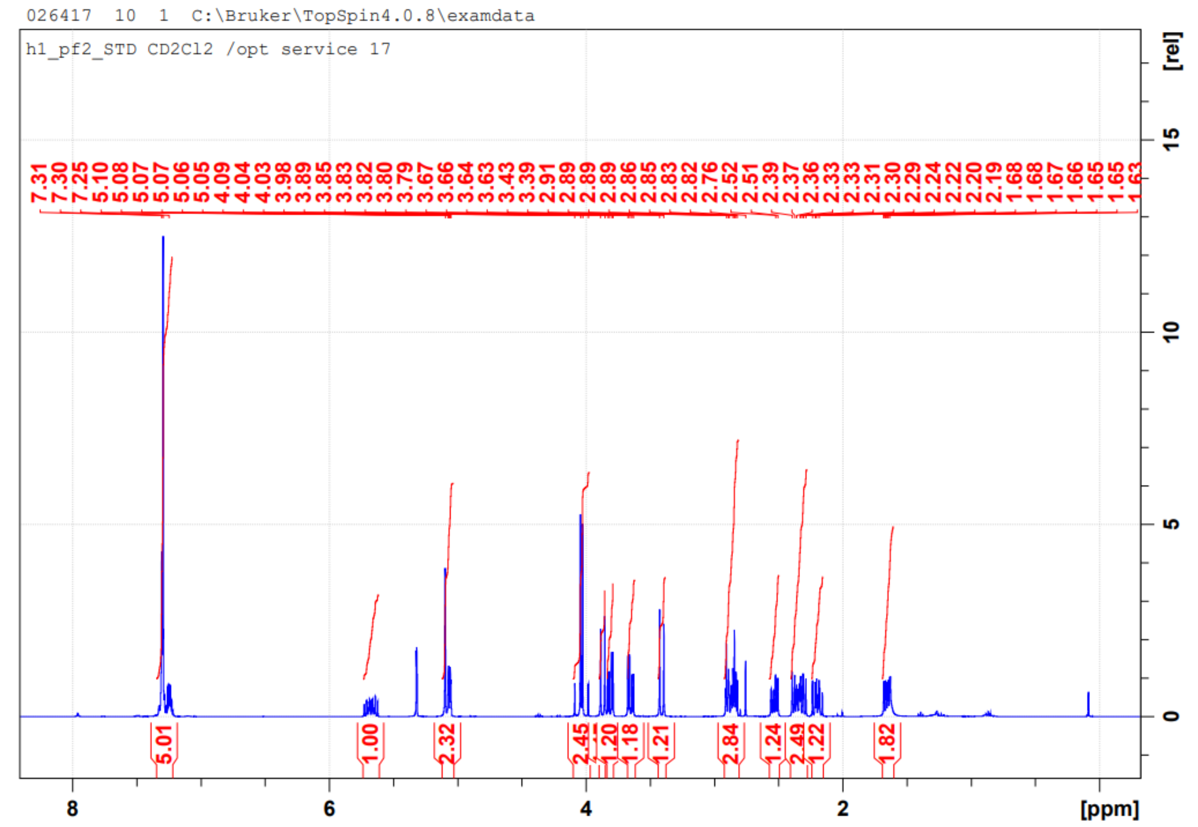

^{13}C-NMR

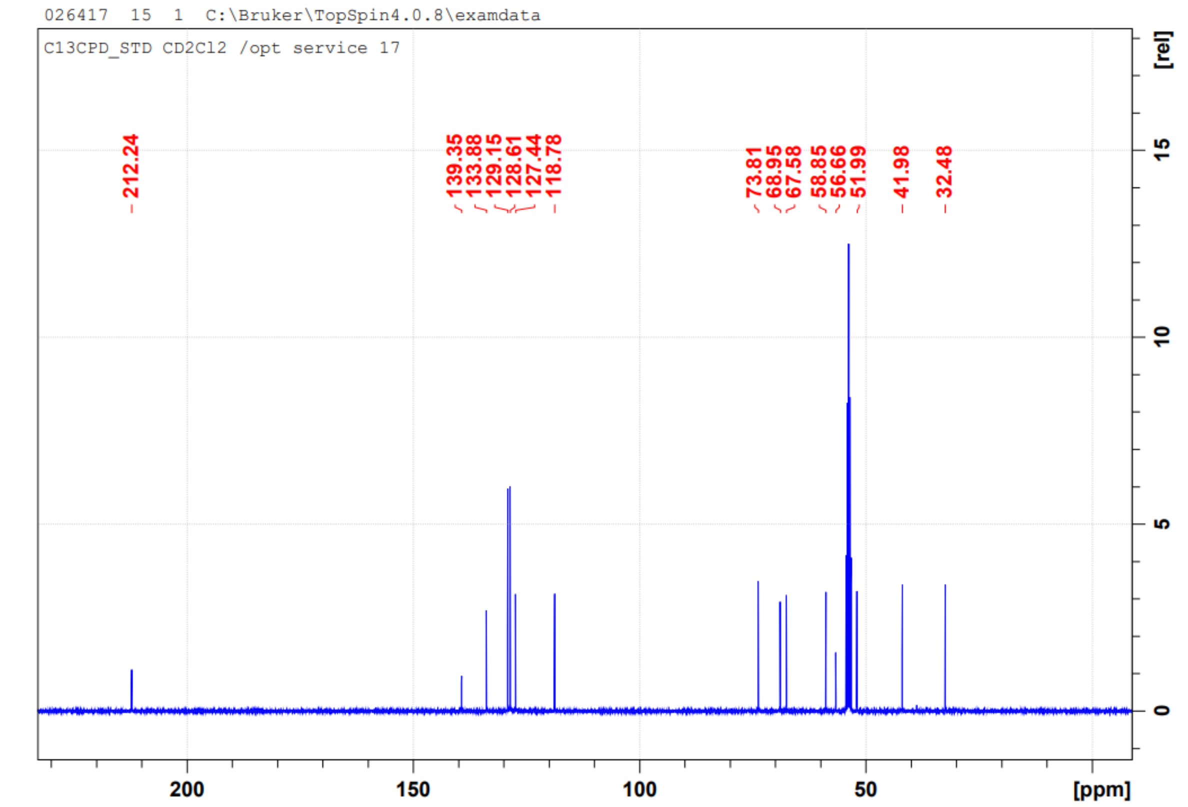

Compound 11b

^{1}H-NMR

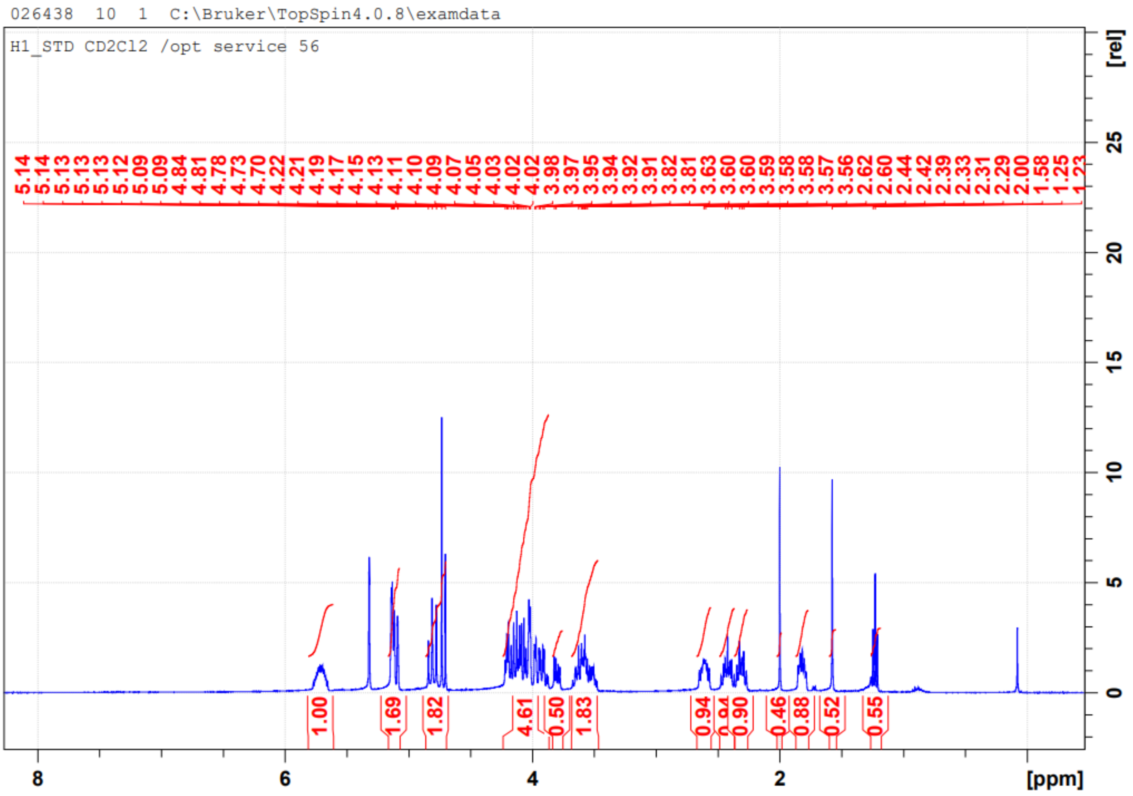

^{13}C-NMR

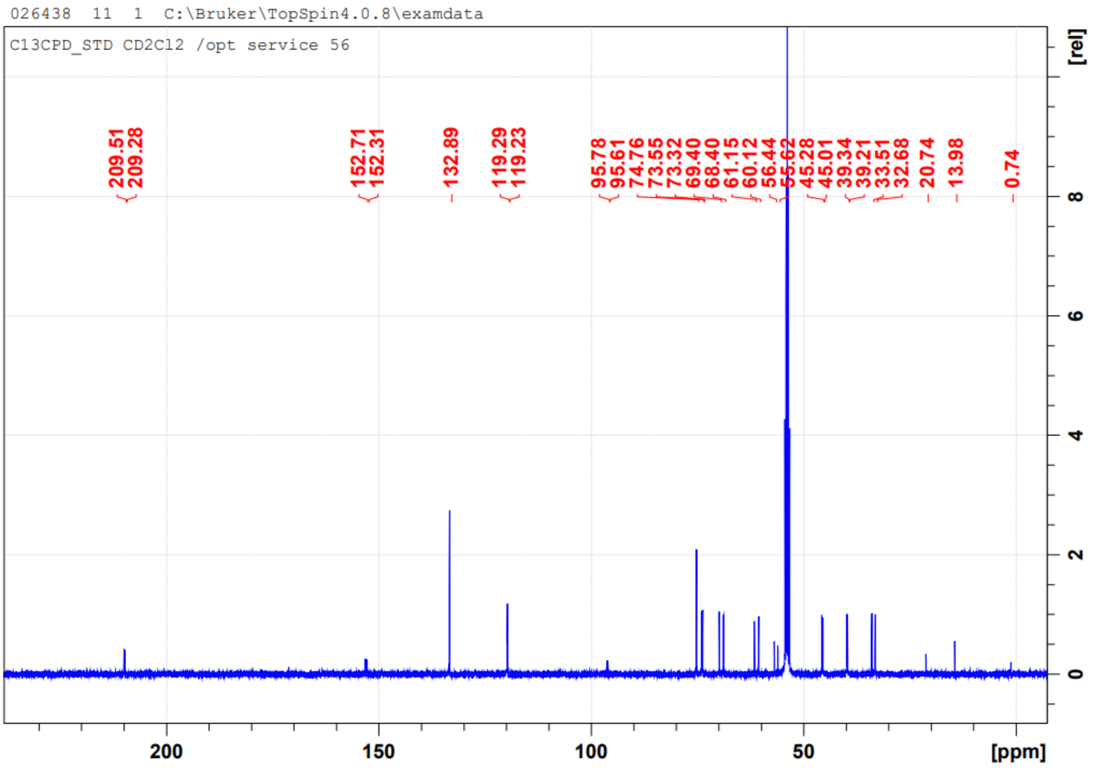

Compound 12b

^{1}H-NMR

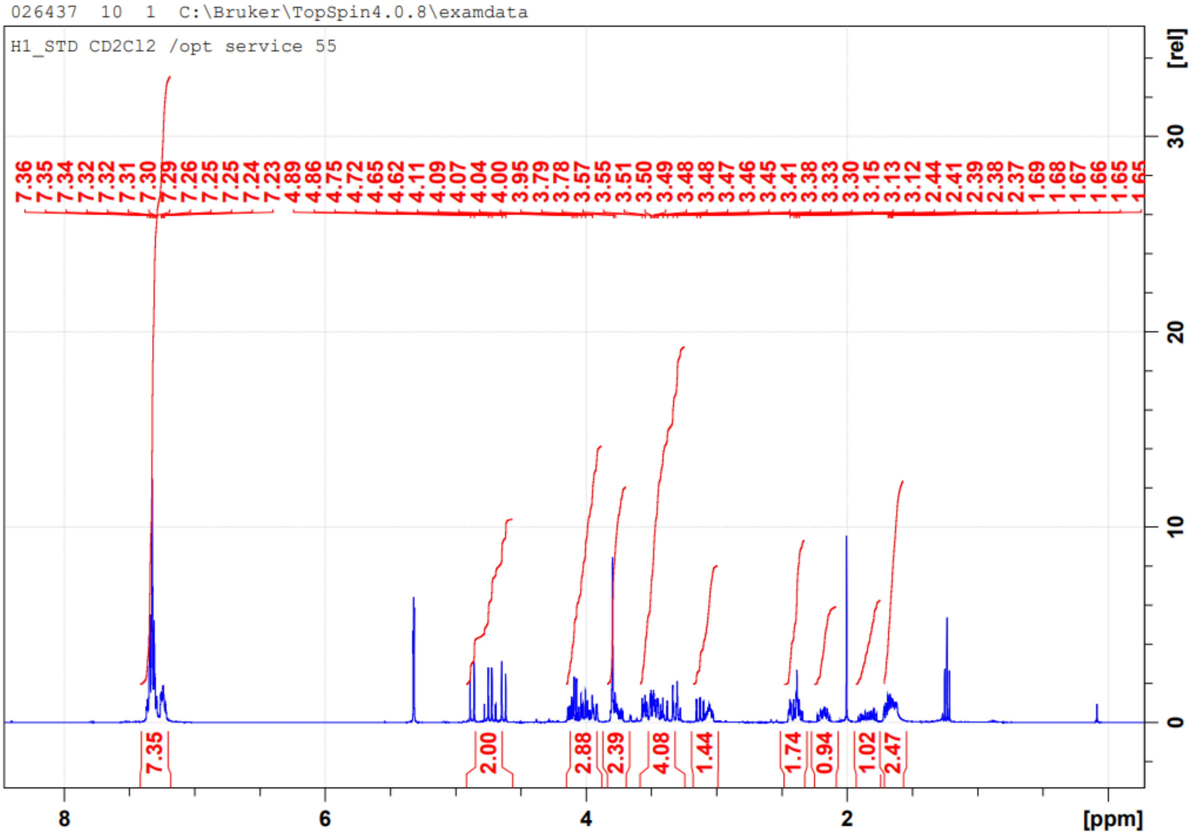

^{13}C-NMR

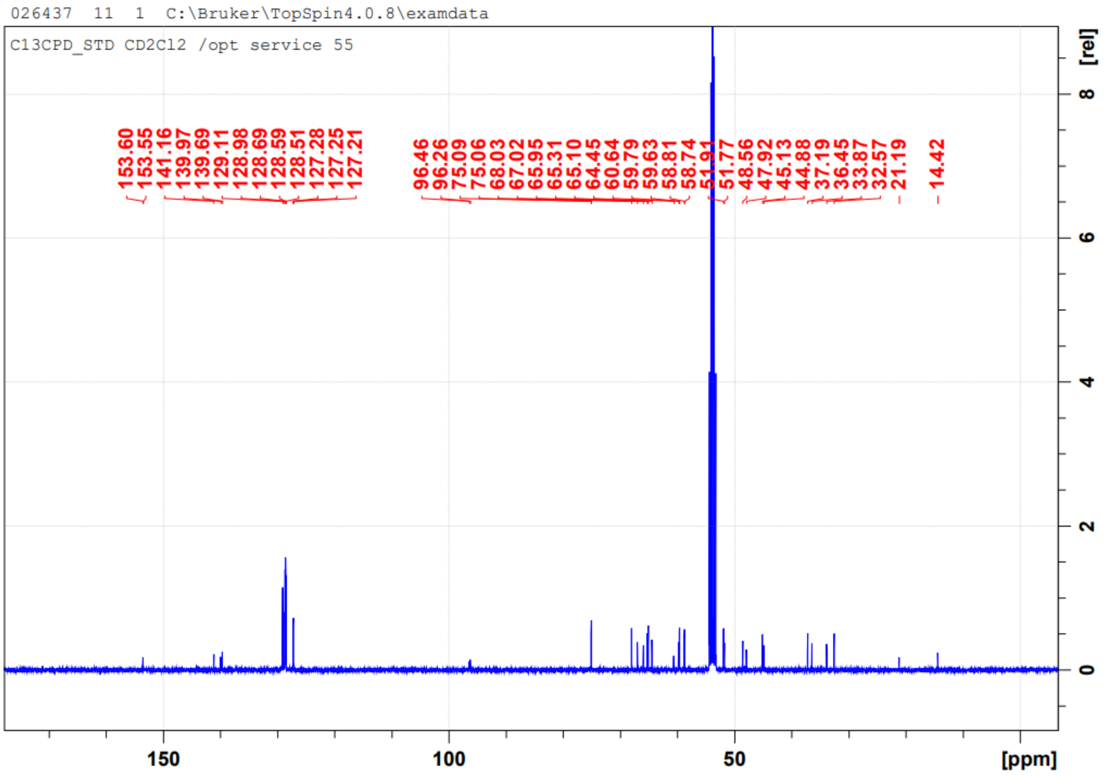

Compound 17a

^{1}H-NMR (40 °C)

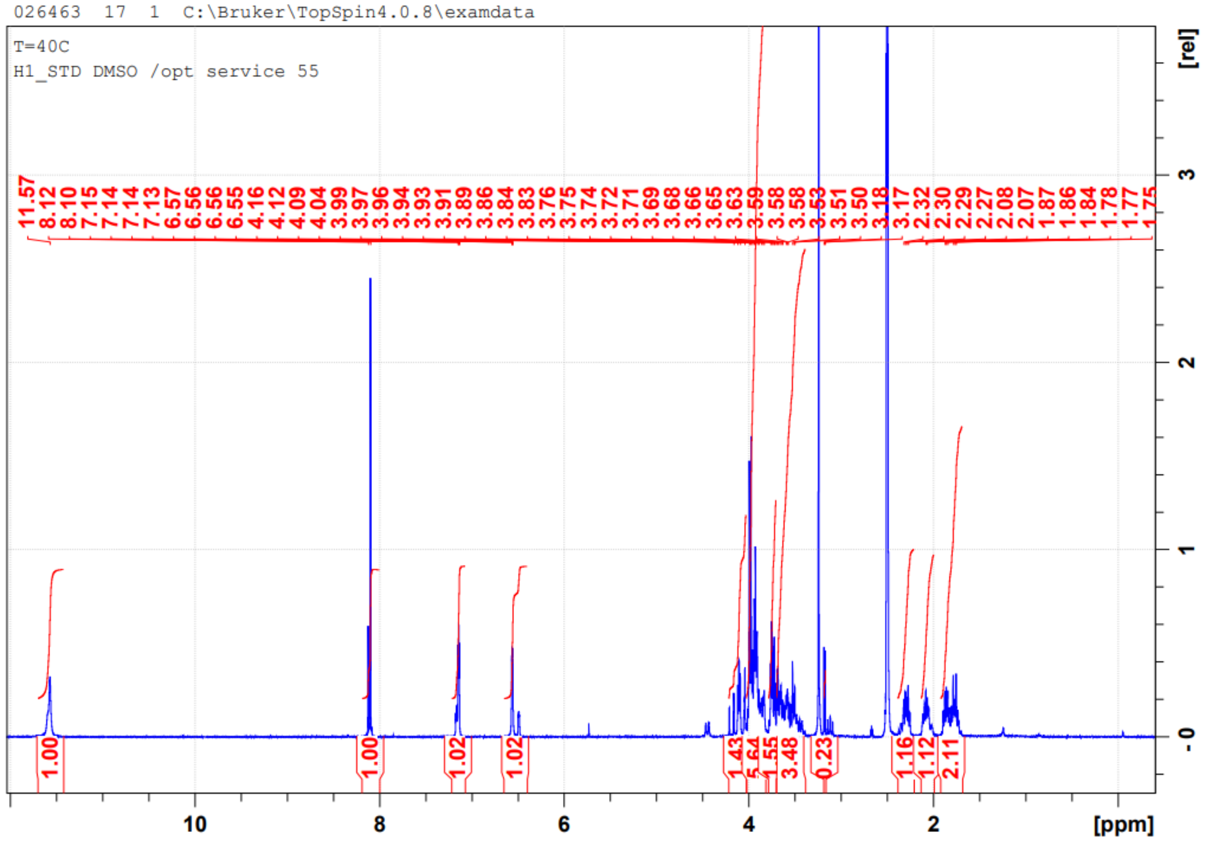

^{1}H-NMR (60 °C)

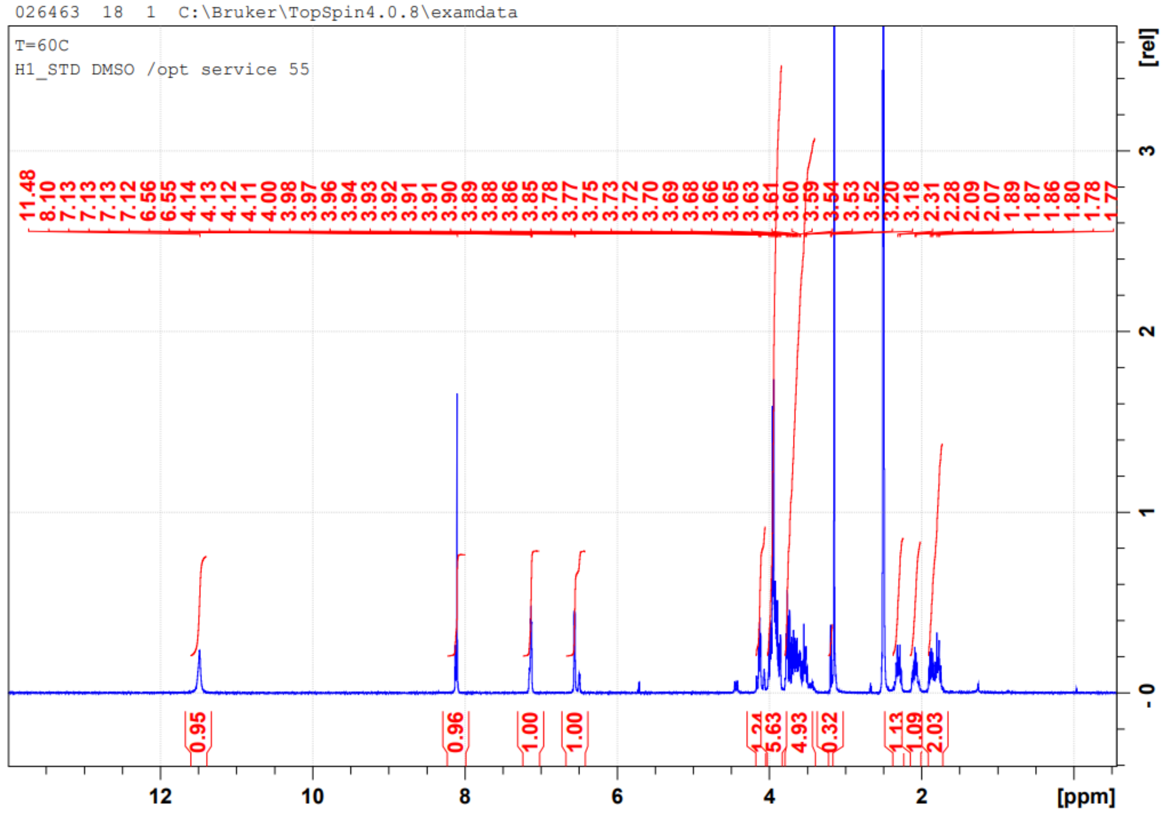

^{1}H-NMR (80 °C)

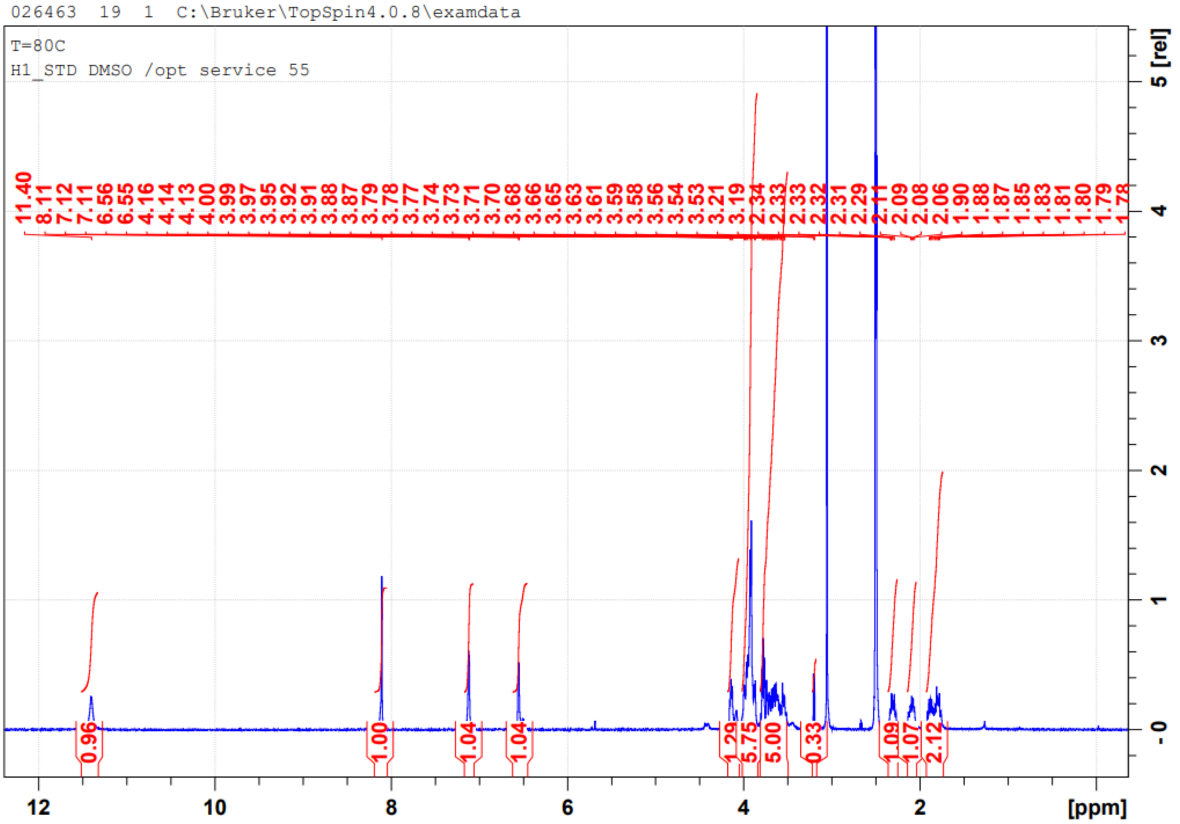

^{13}C-NMR

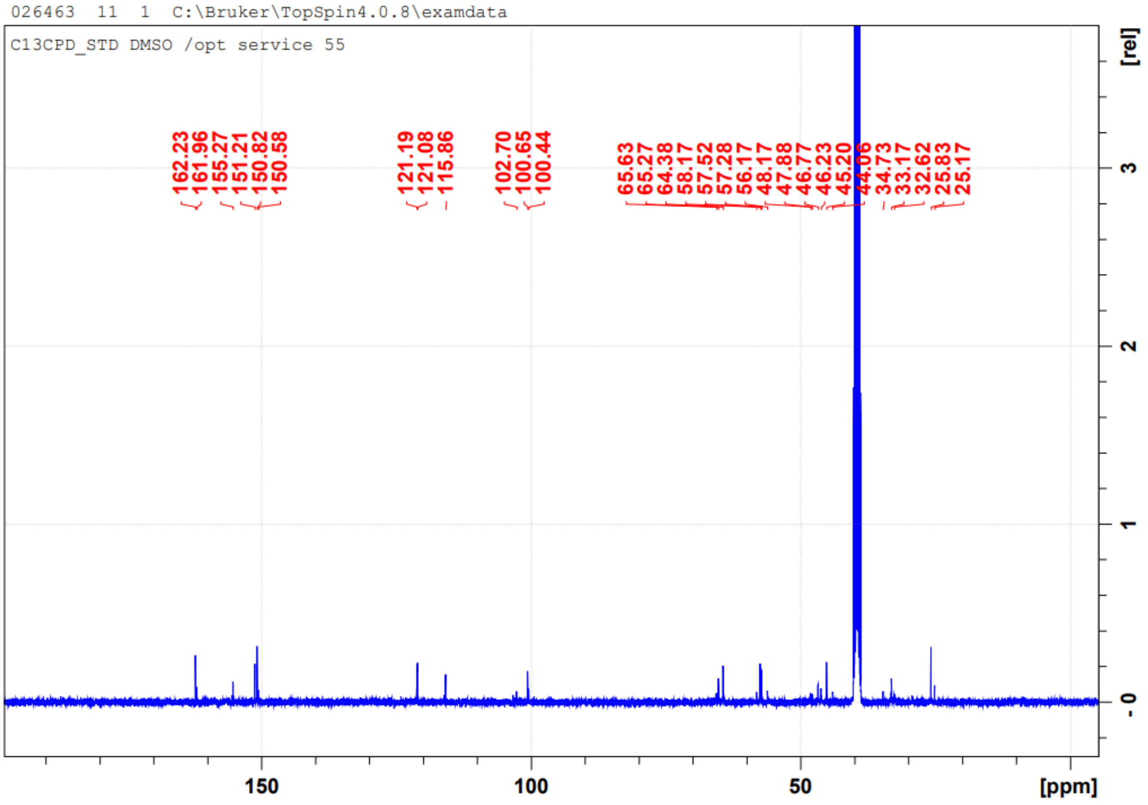

Compound 17c

^{1}H-NMR

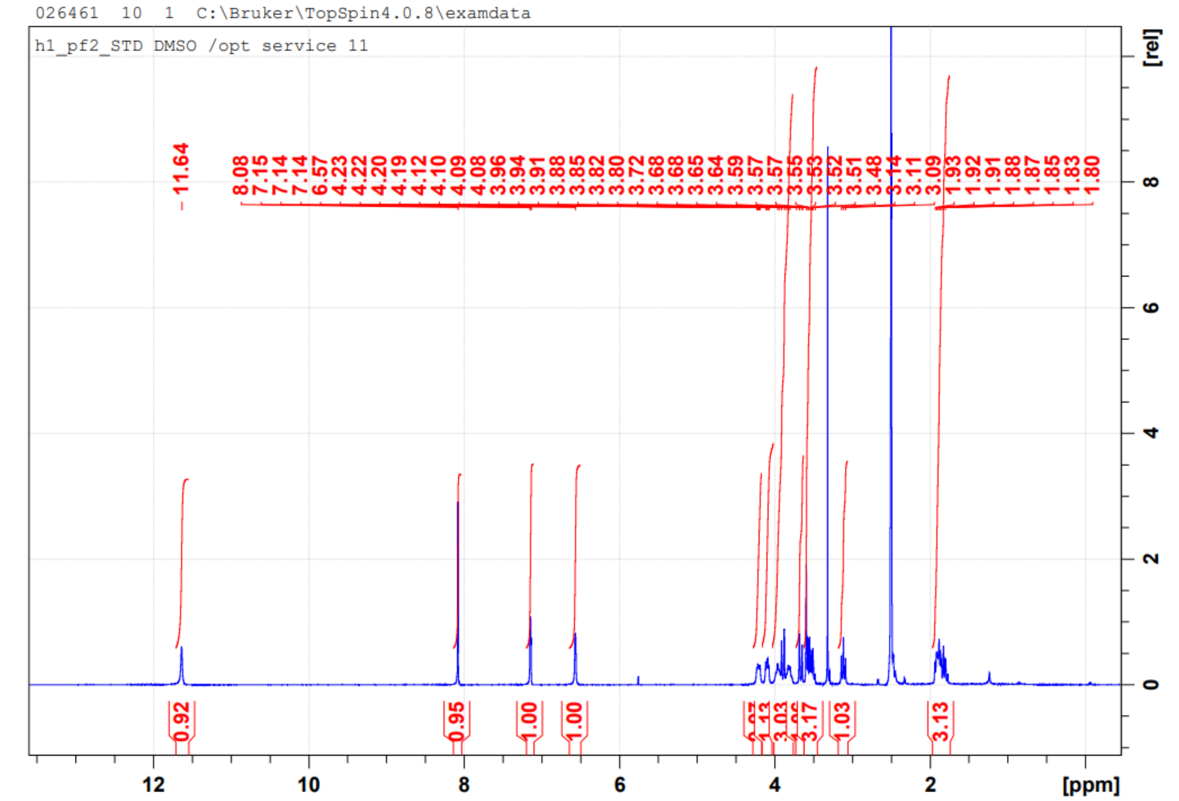

^{13}C-NMR

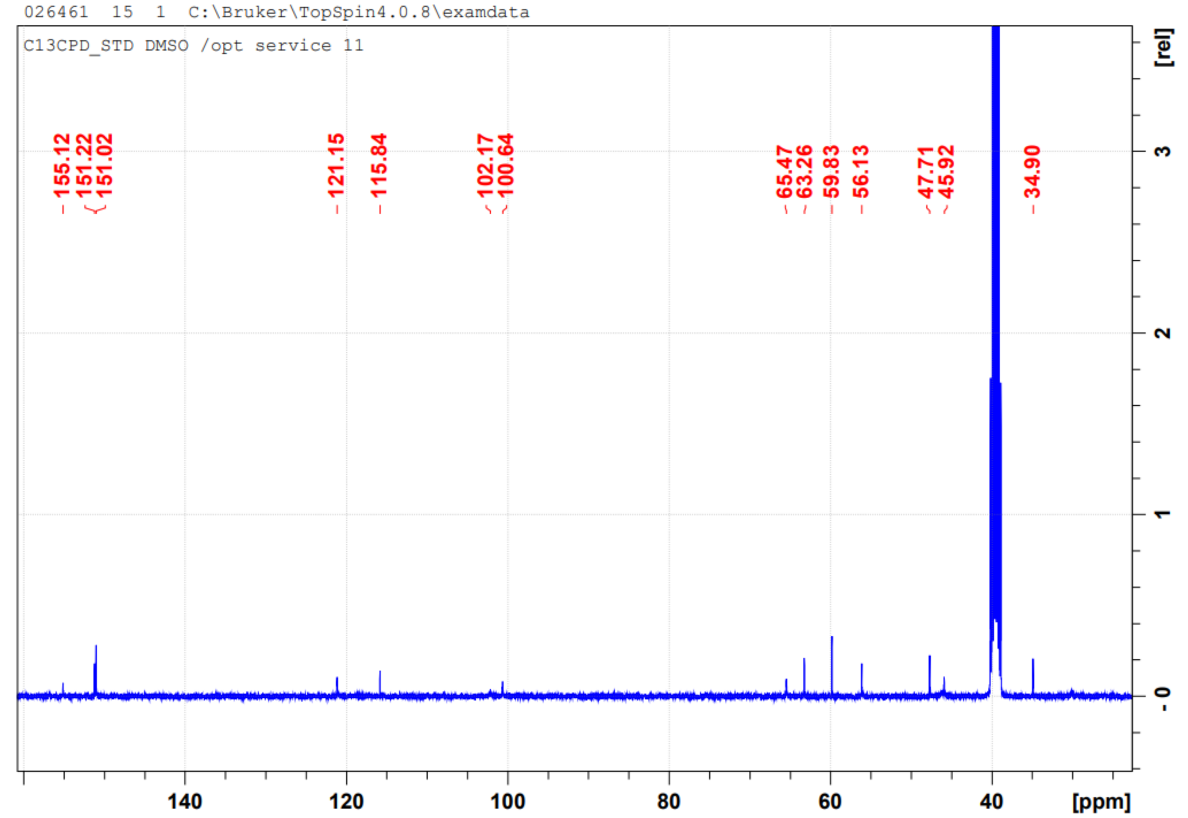

Compound 17d

^{1}H-NMR

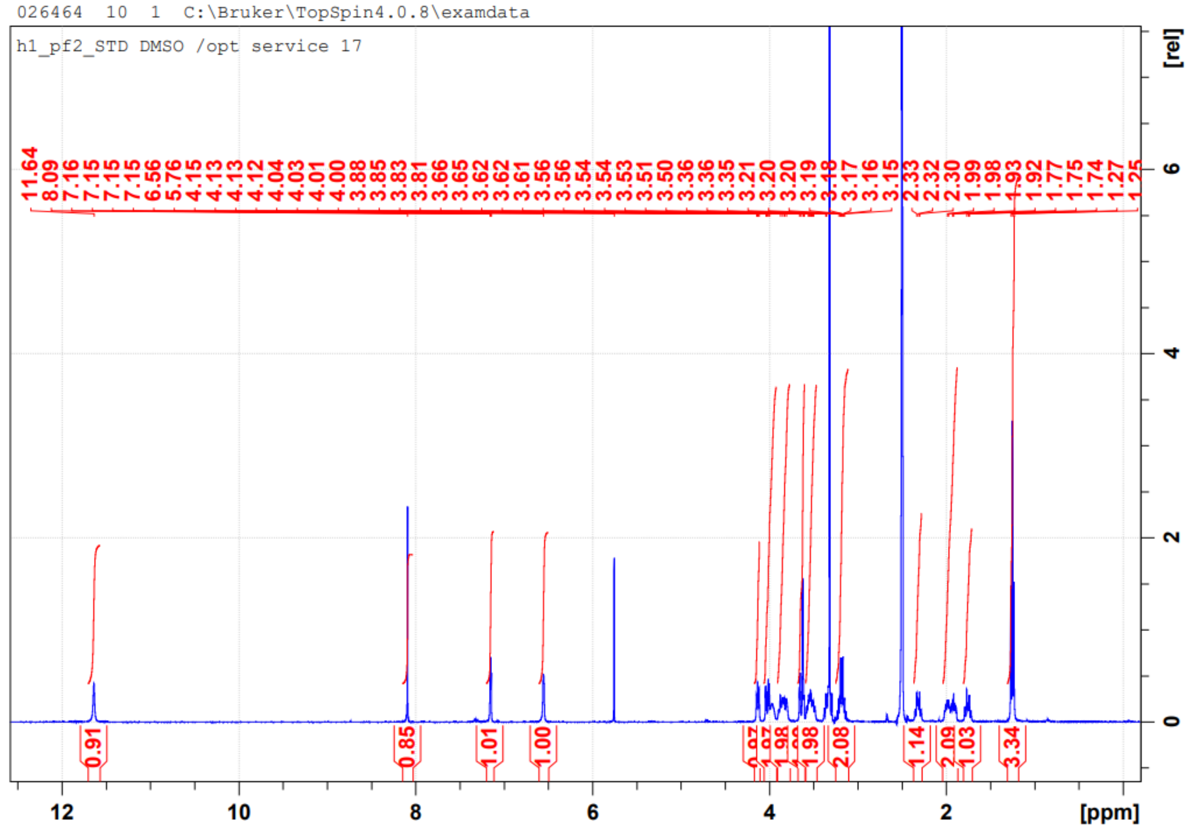

^{13}C-NMR

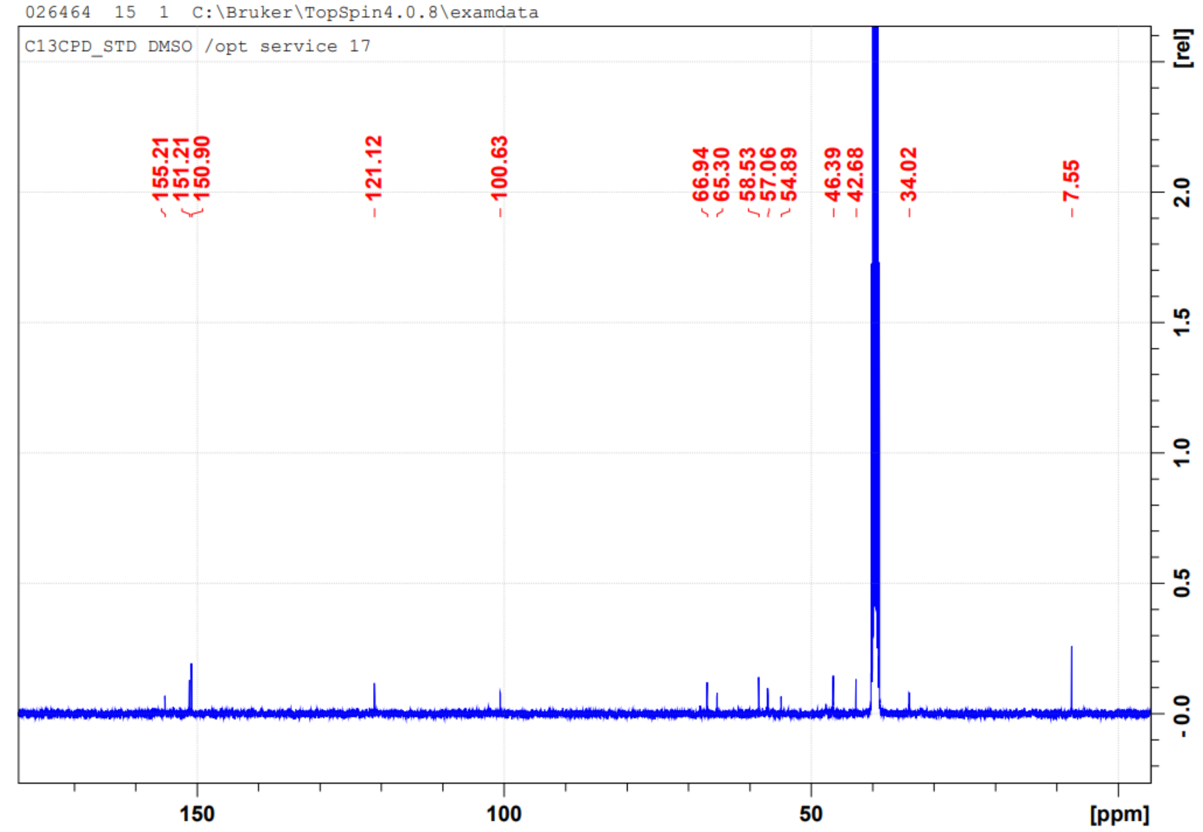

Compound 19

^{1}H-NMR

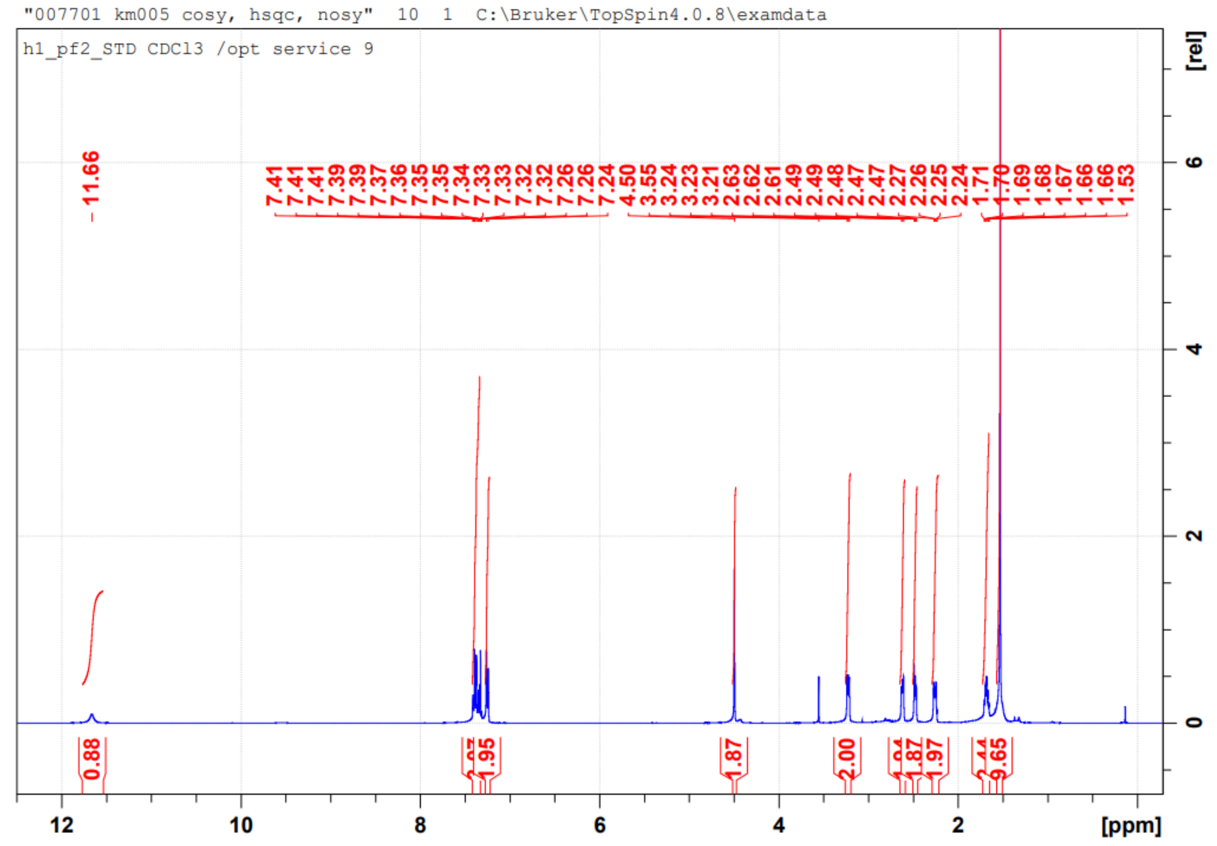

^{13}C-NMR

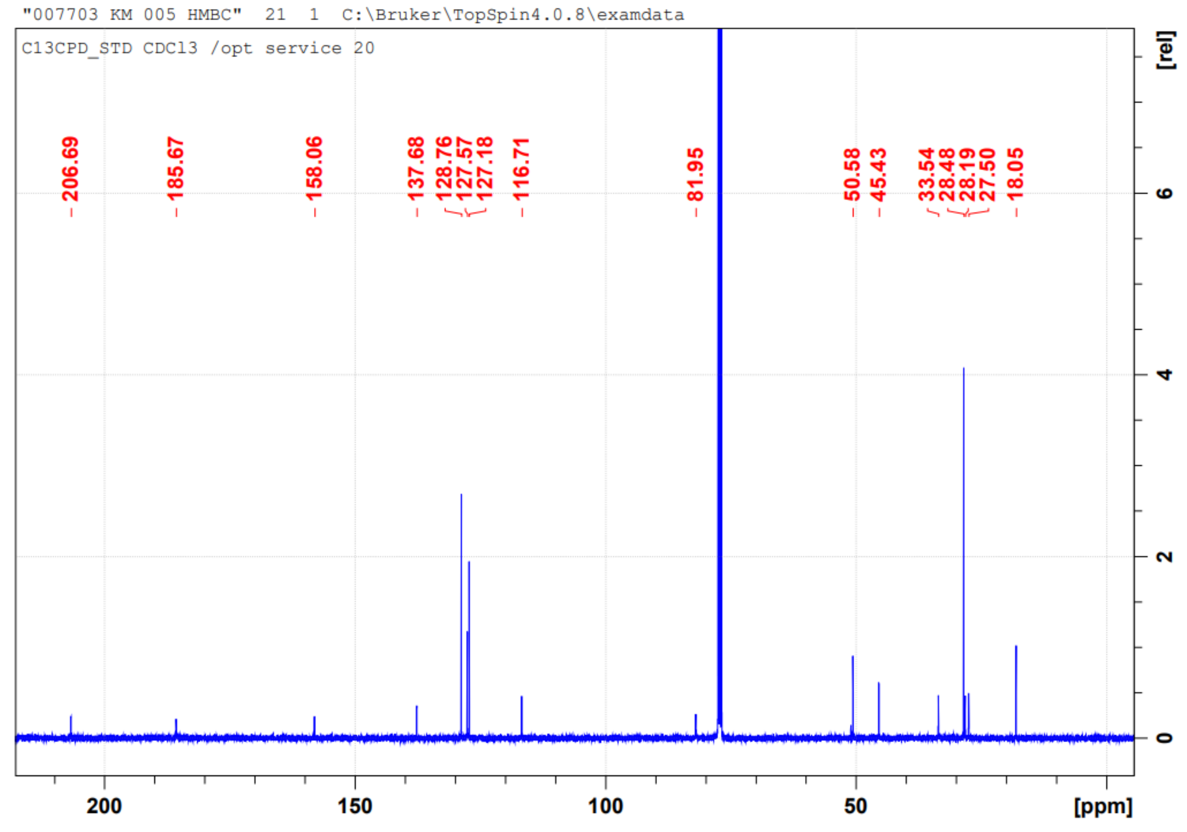

Compound 20

^{1}H-NMR

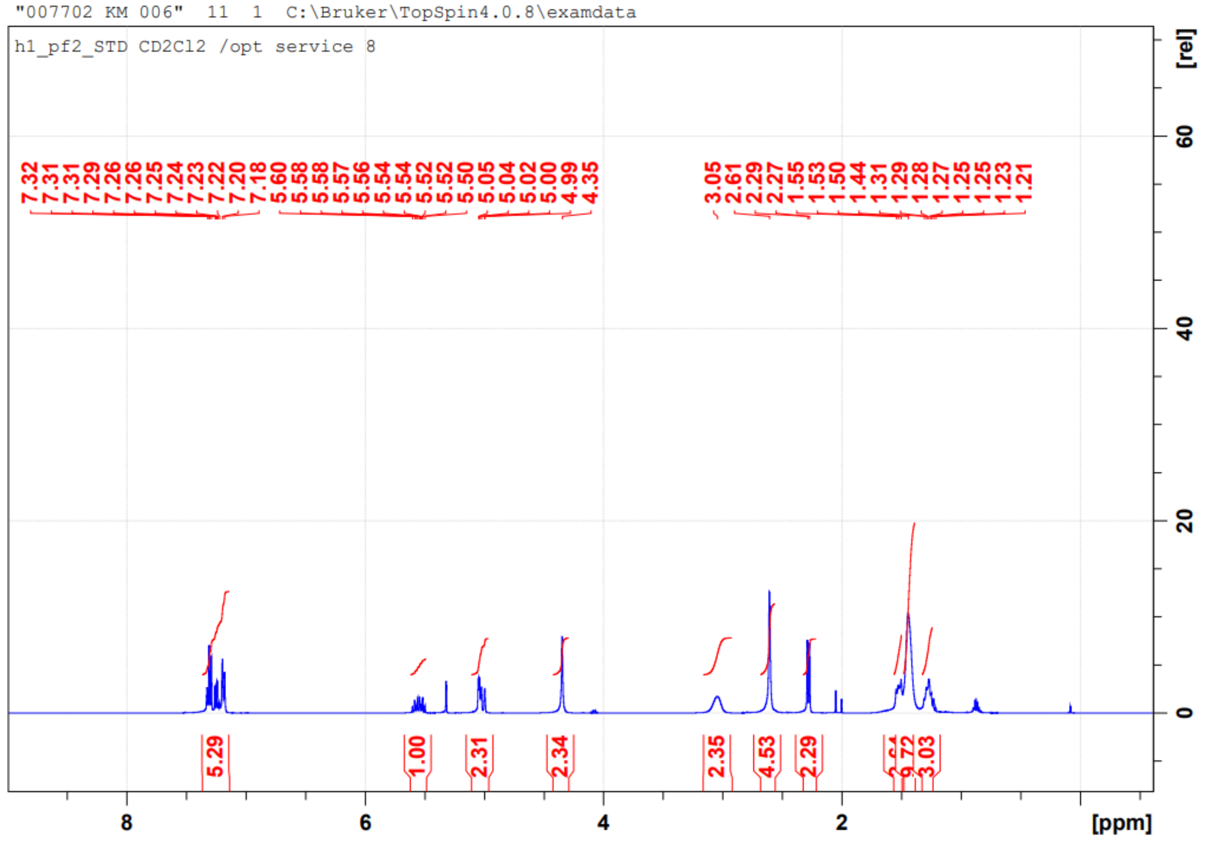

^{13}C-NMR

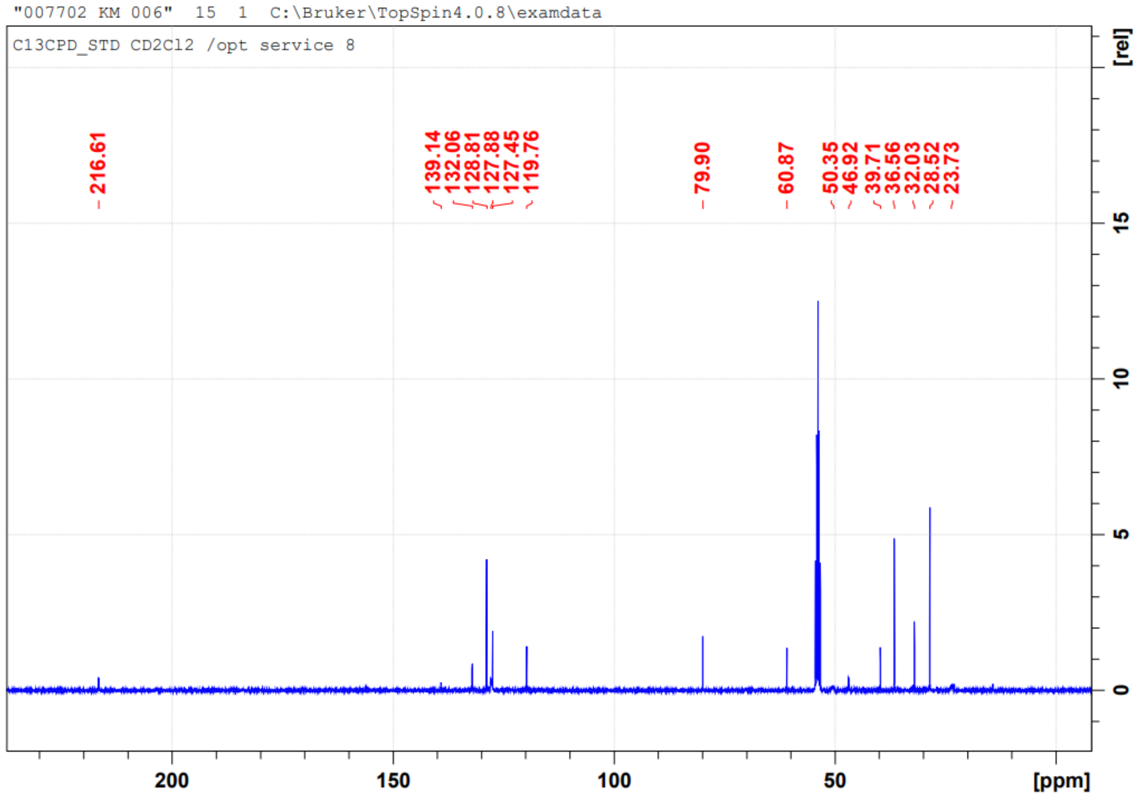

Compound 21

^{1}H-NMR

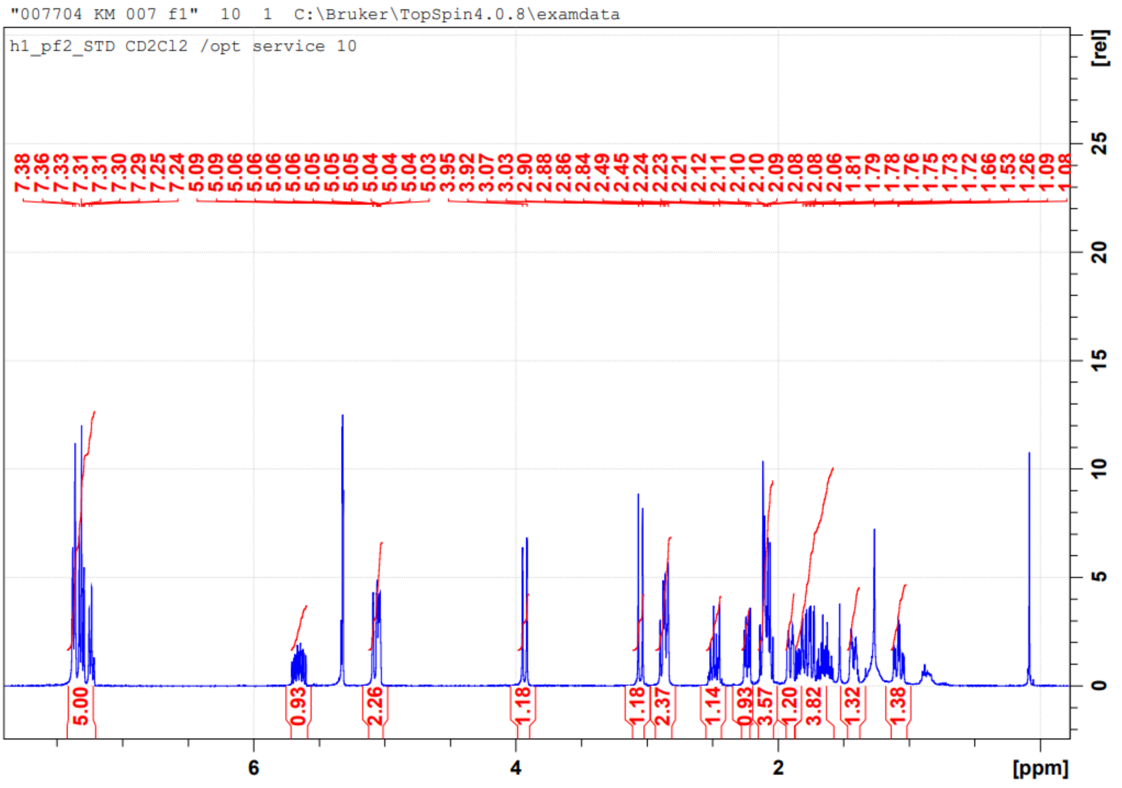

^{13}C-NMR

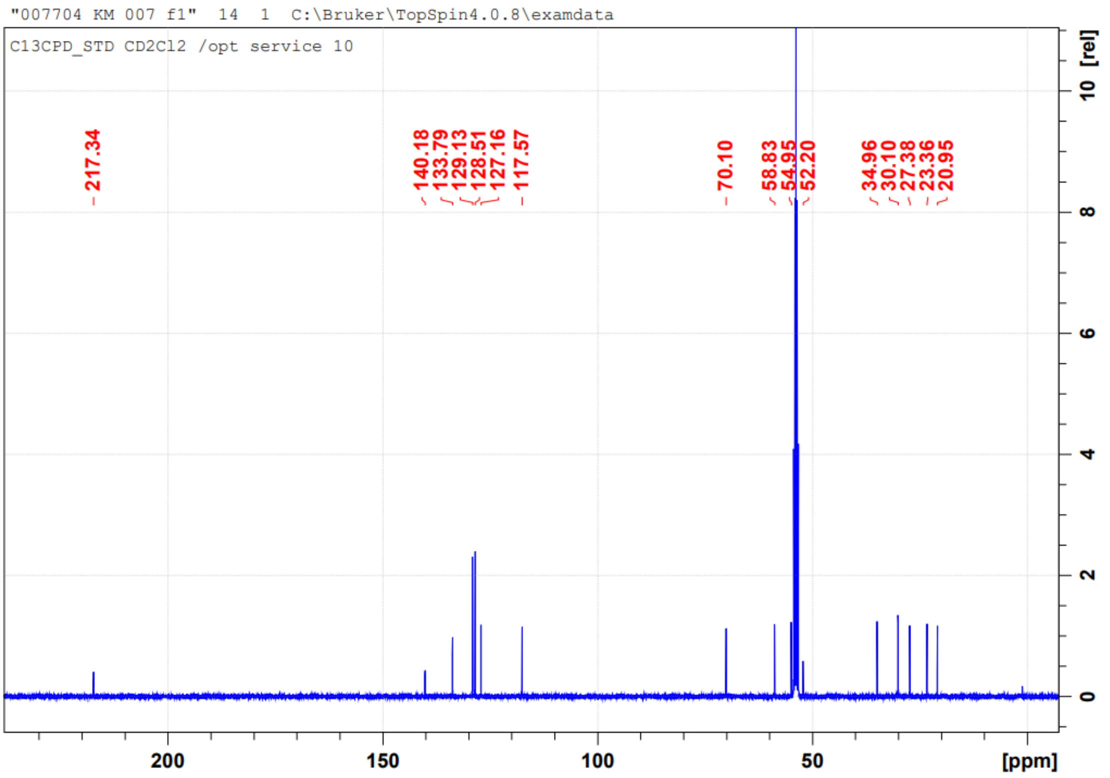

Compound 22
^{1}H-NMR

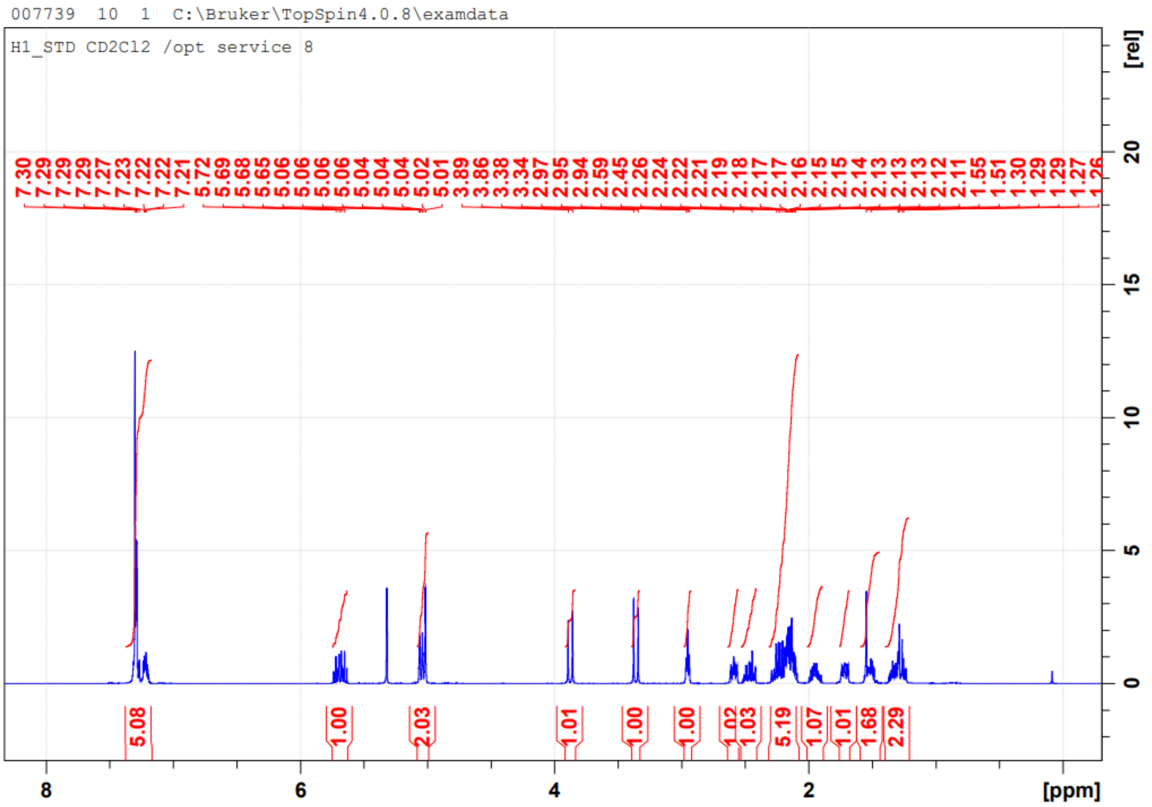

^{13}C-NMR

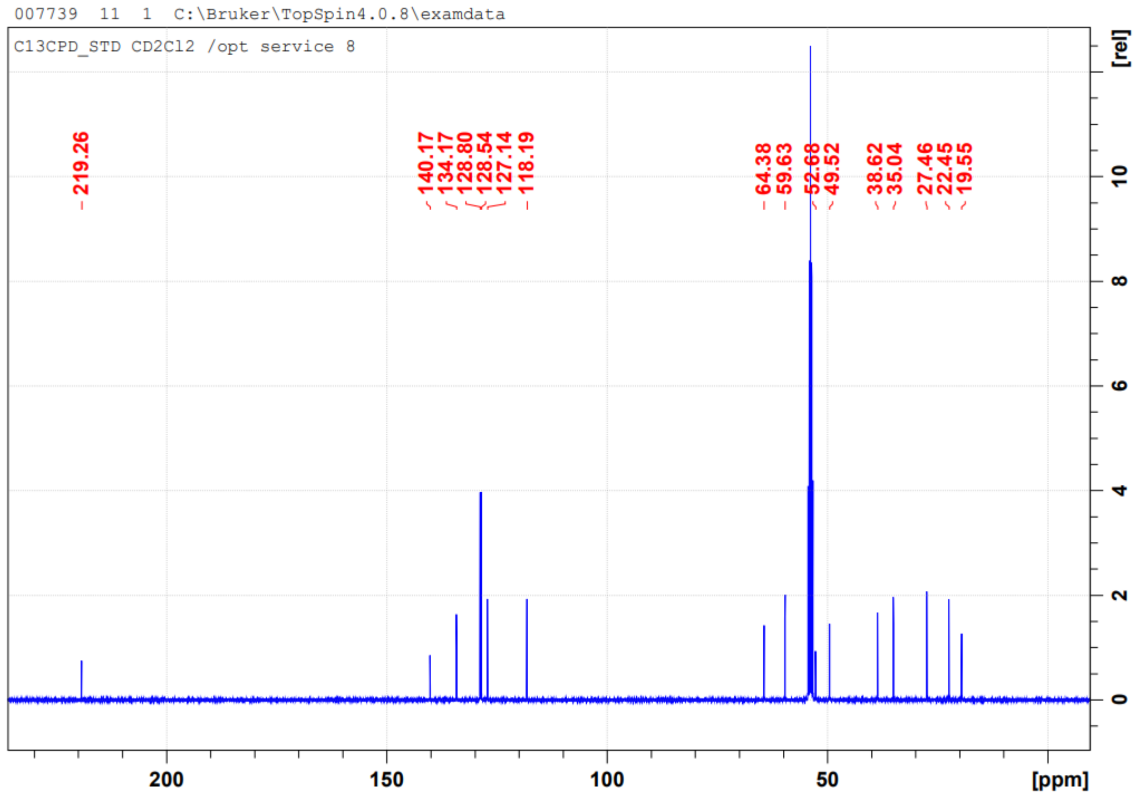

Compound 23

^{1}H-NMR

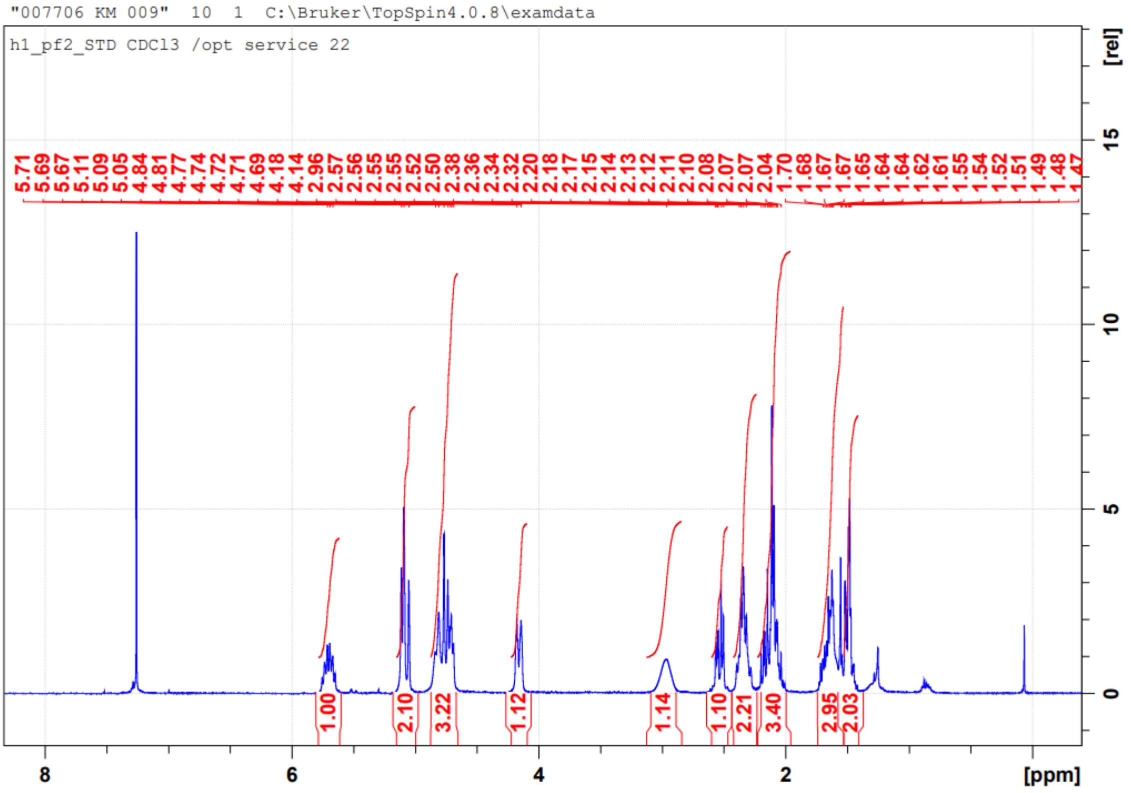

^{13}C-NMR

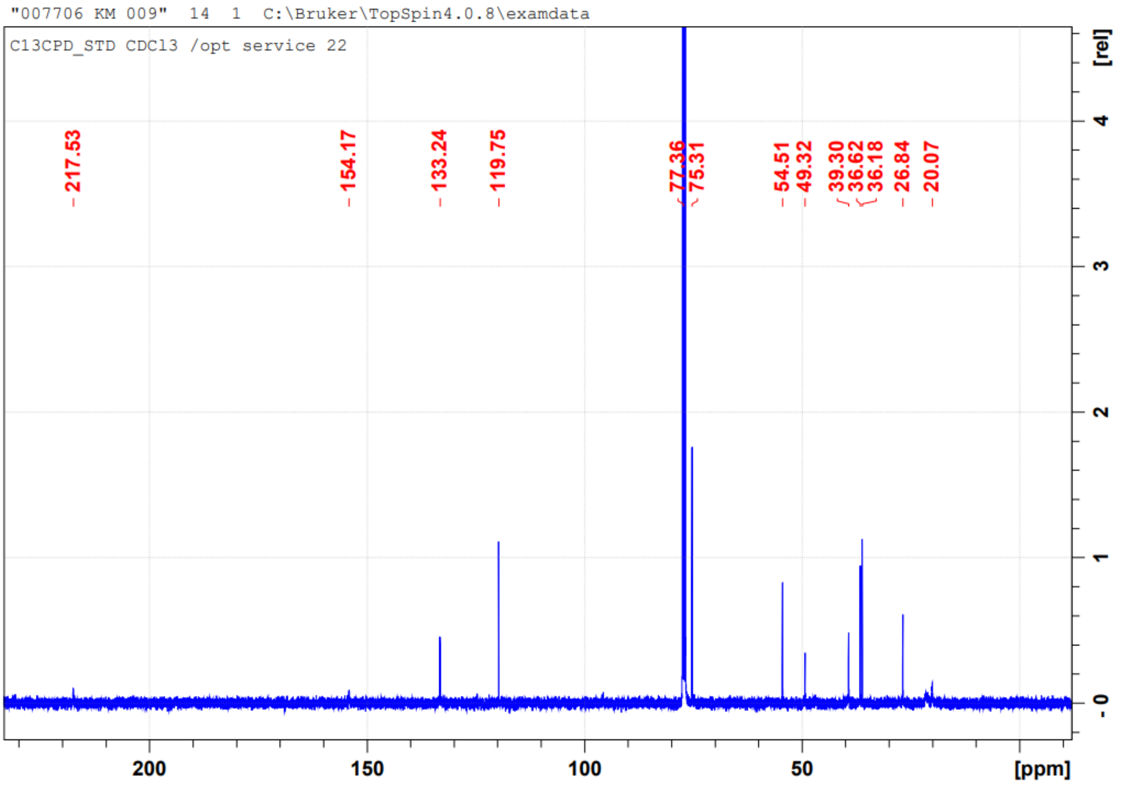

Compound 24

^{1}H-NMR

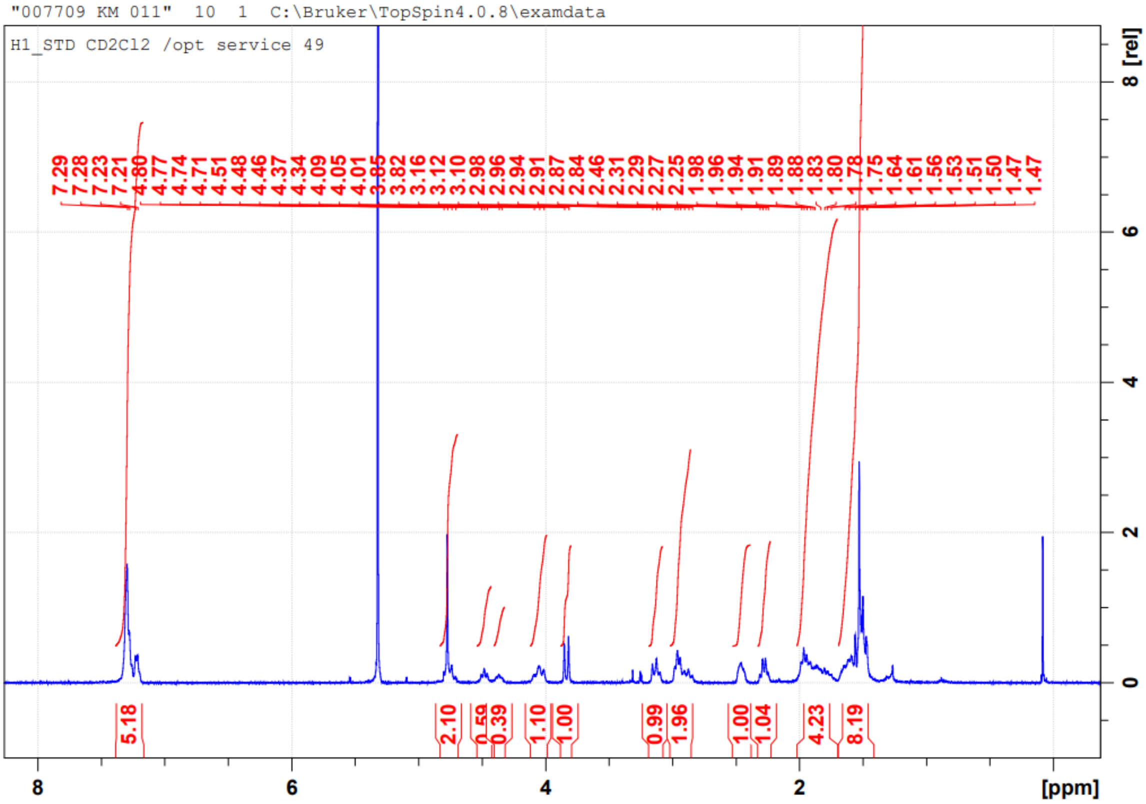

^{13}C-NMR

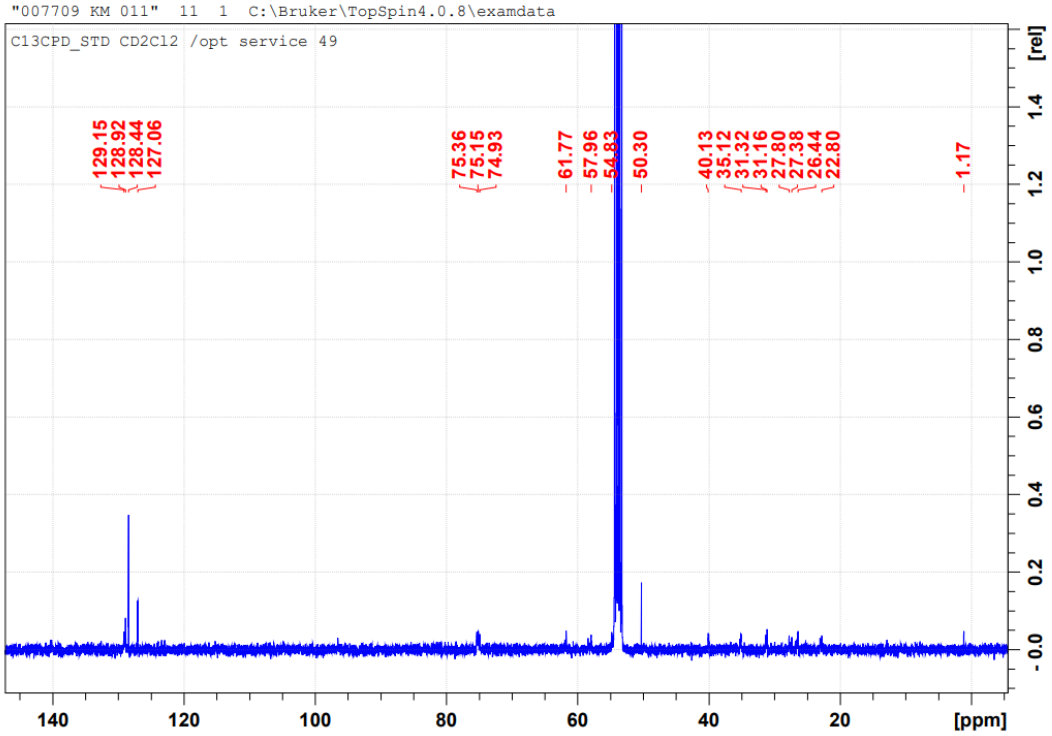

Compound 25

^{1}H-NMR

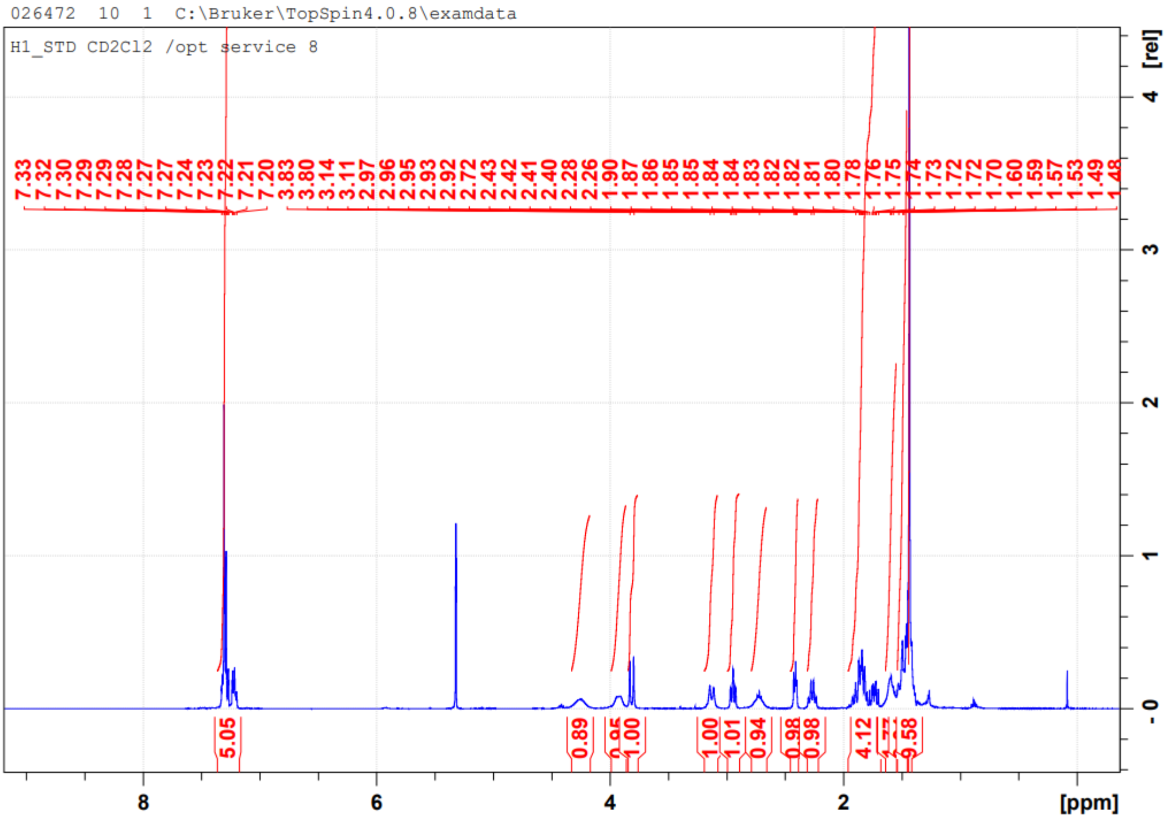

^{13}C-NMR

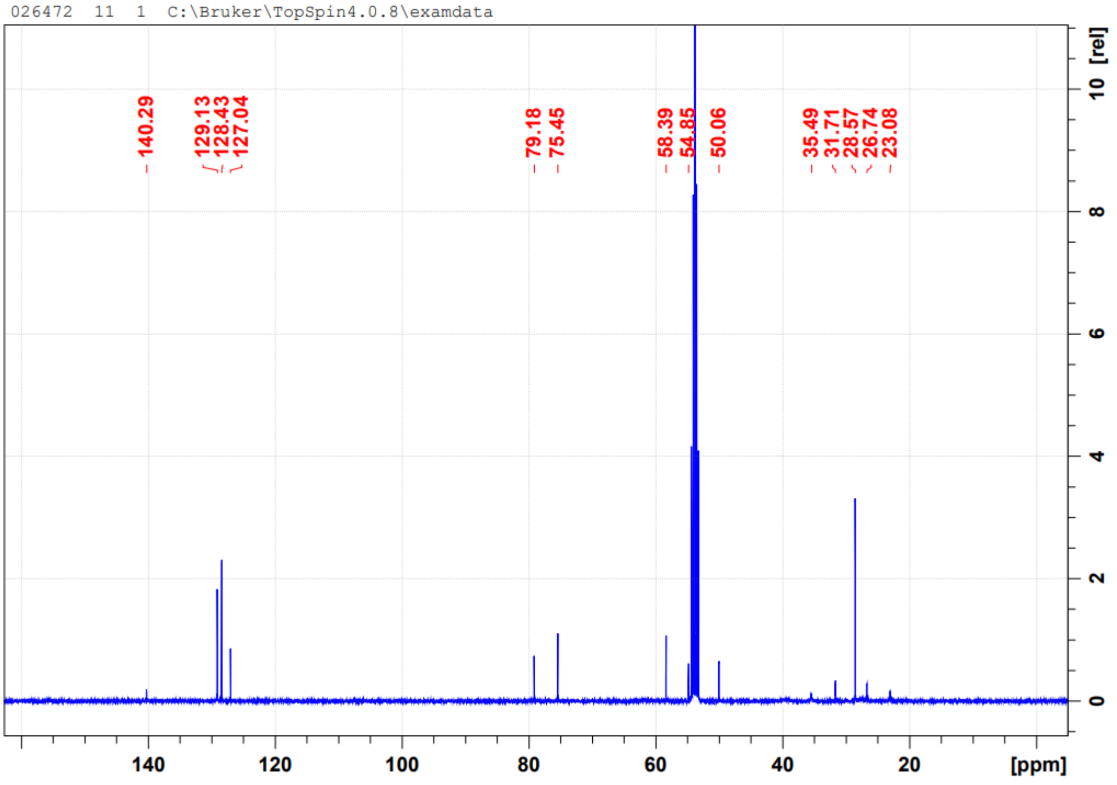

Compounds 26

^{1}H-NMR

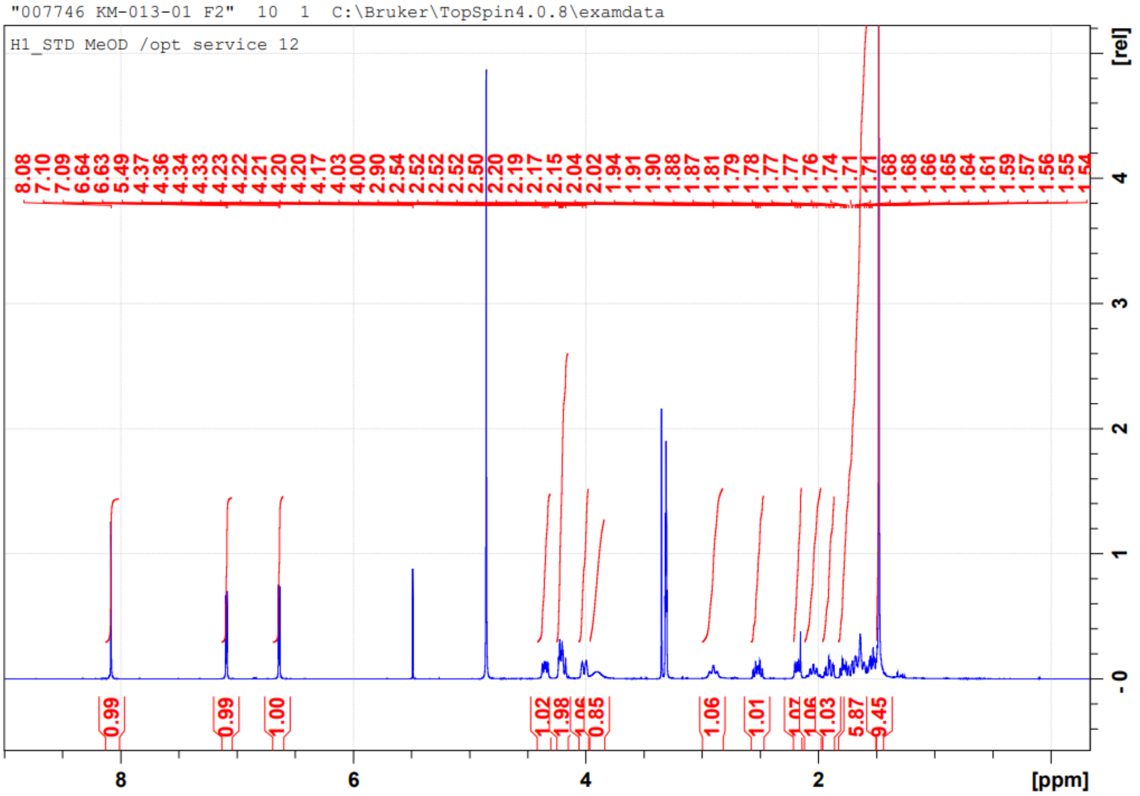

^{13}C-NMR

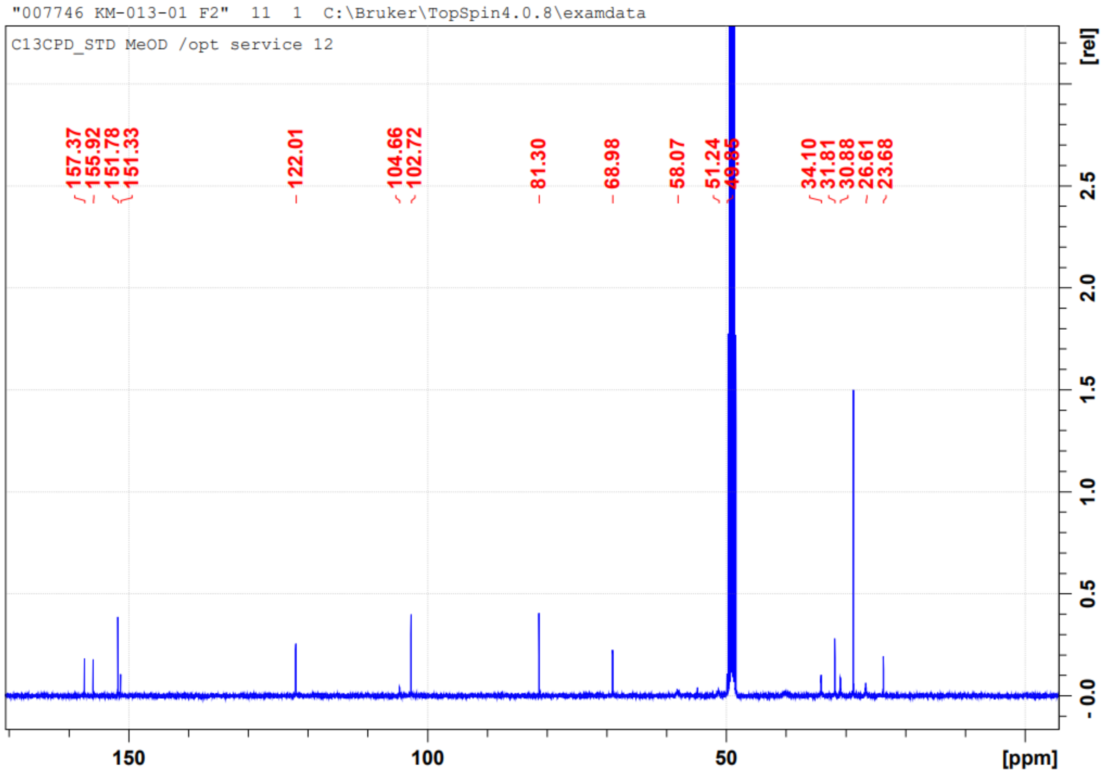

Compound 27

^{1}H-NMR

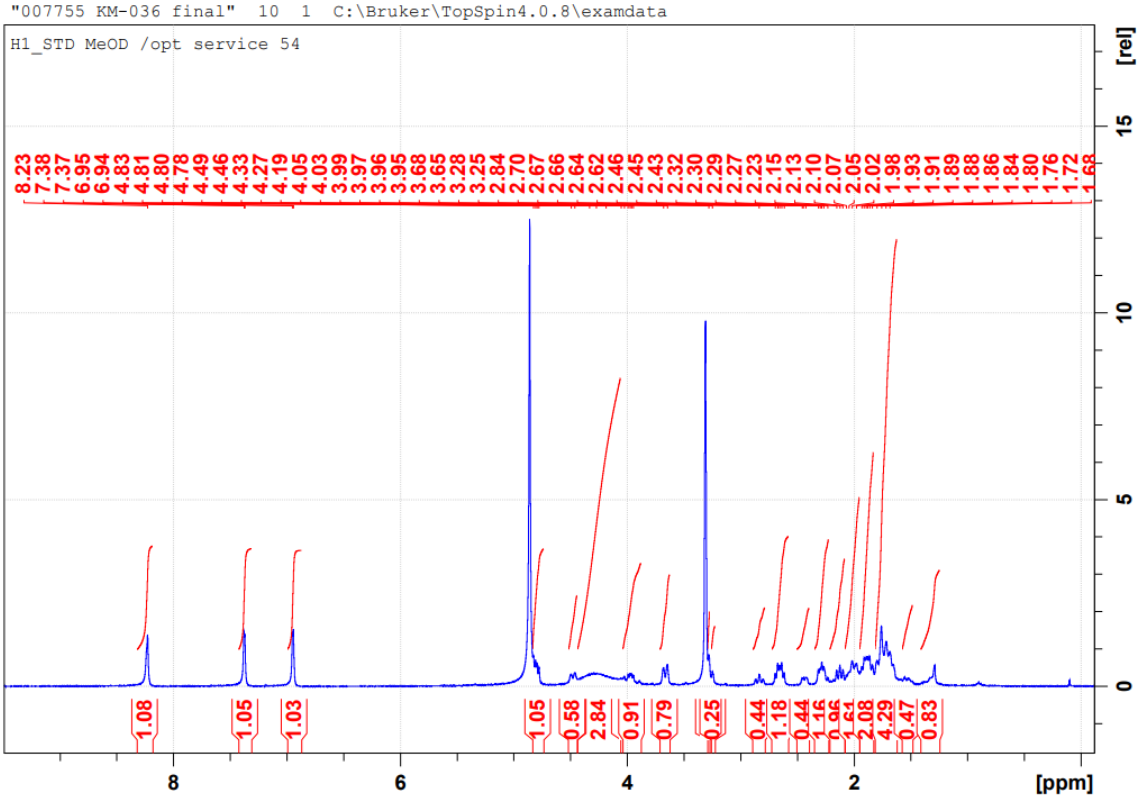

^{13}C-NMR

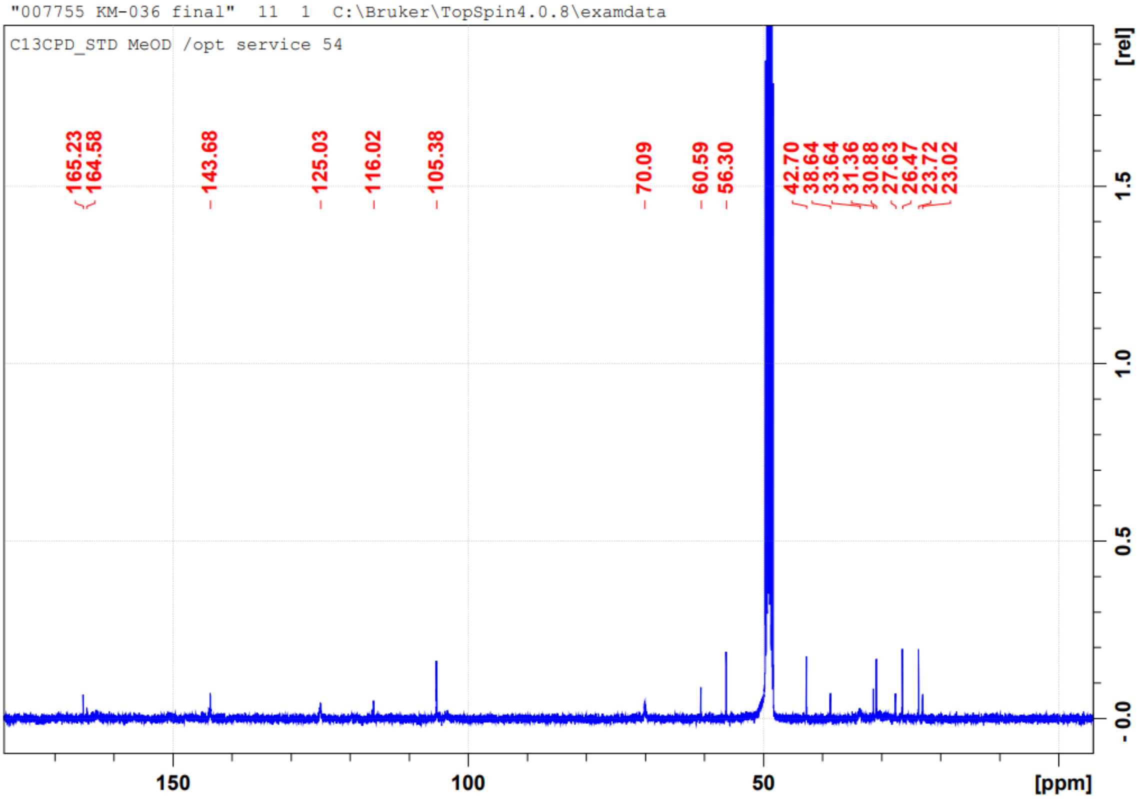

Compound 28

^{1}H-NMR

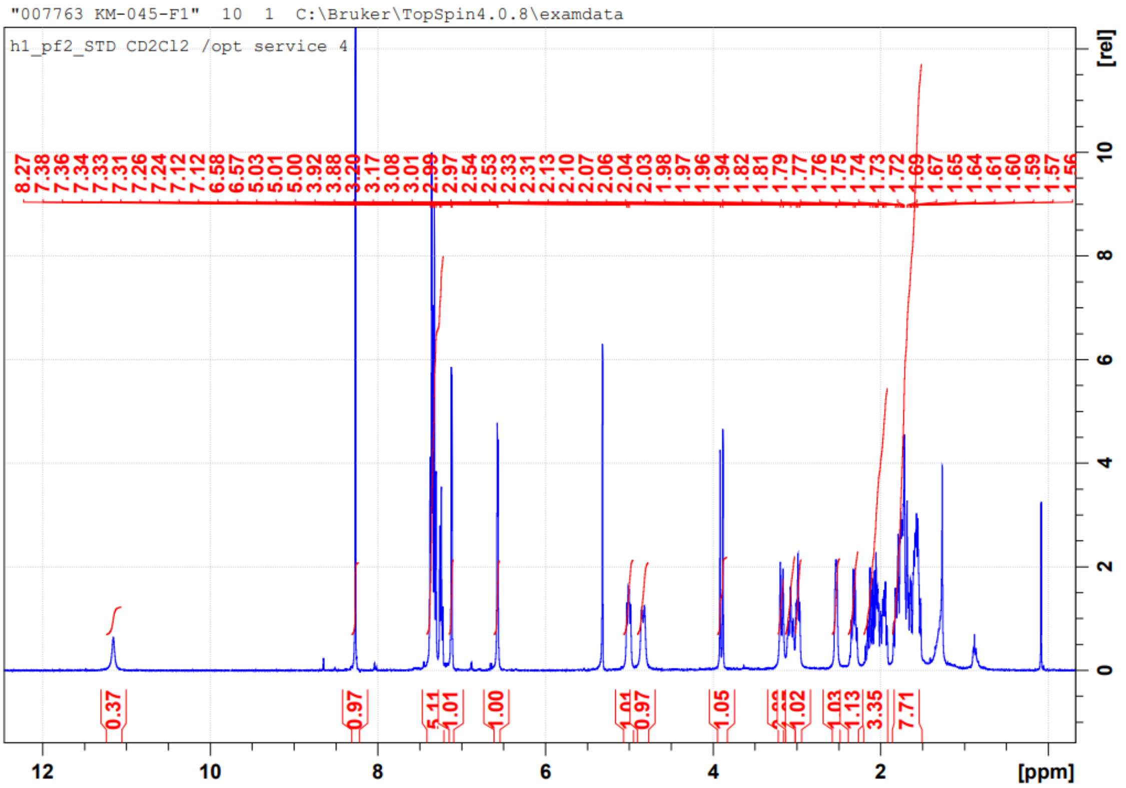

^{13}C-HMR

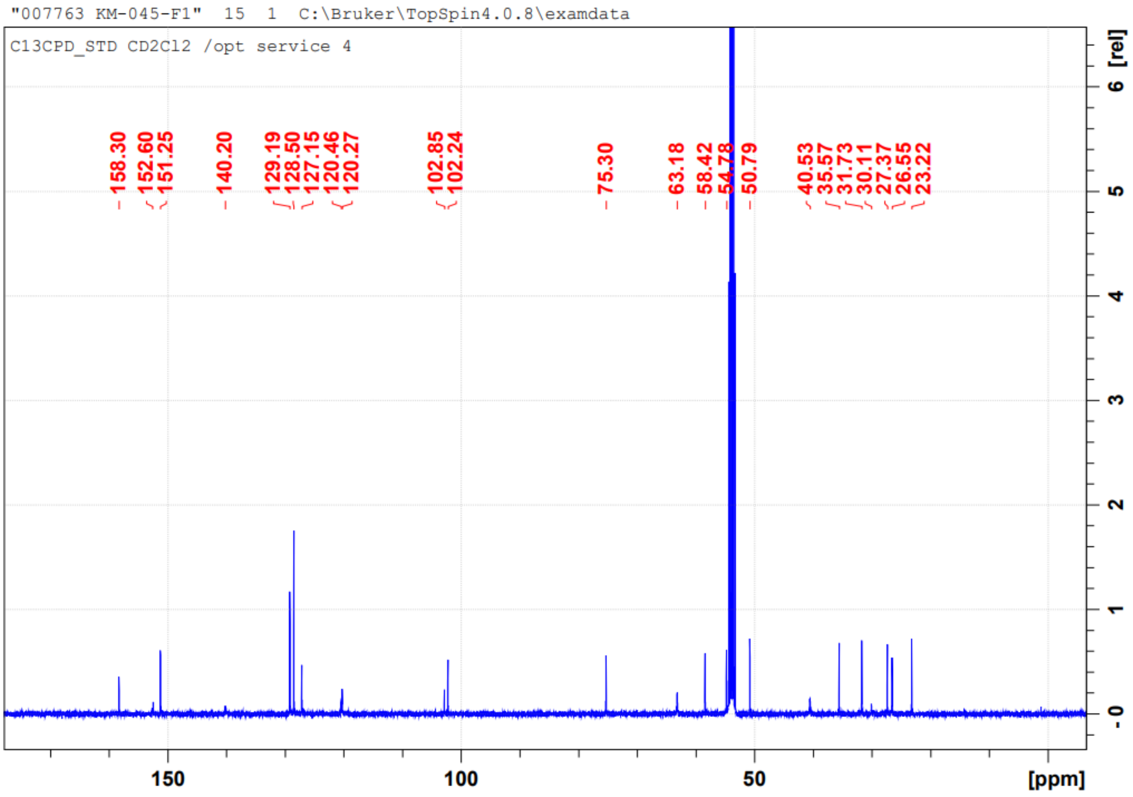

Compound 29

^{1}H-NMR

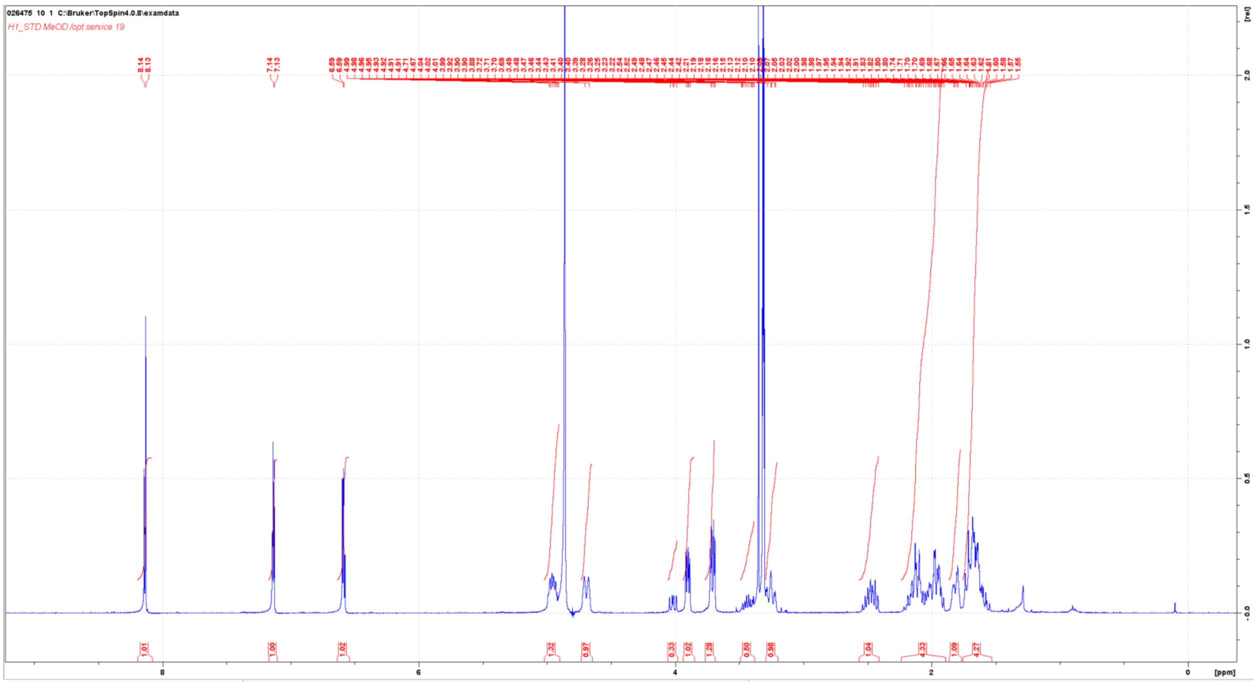

^{13}C-NMR

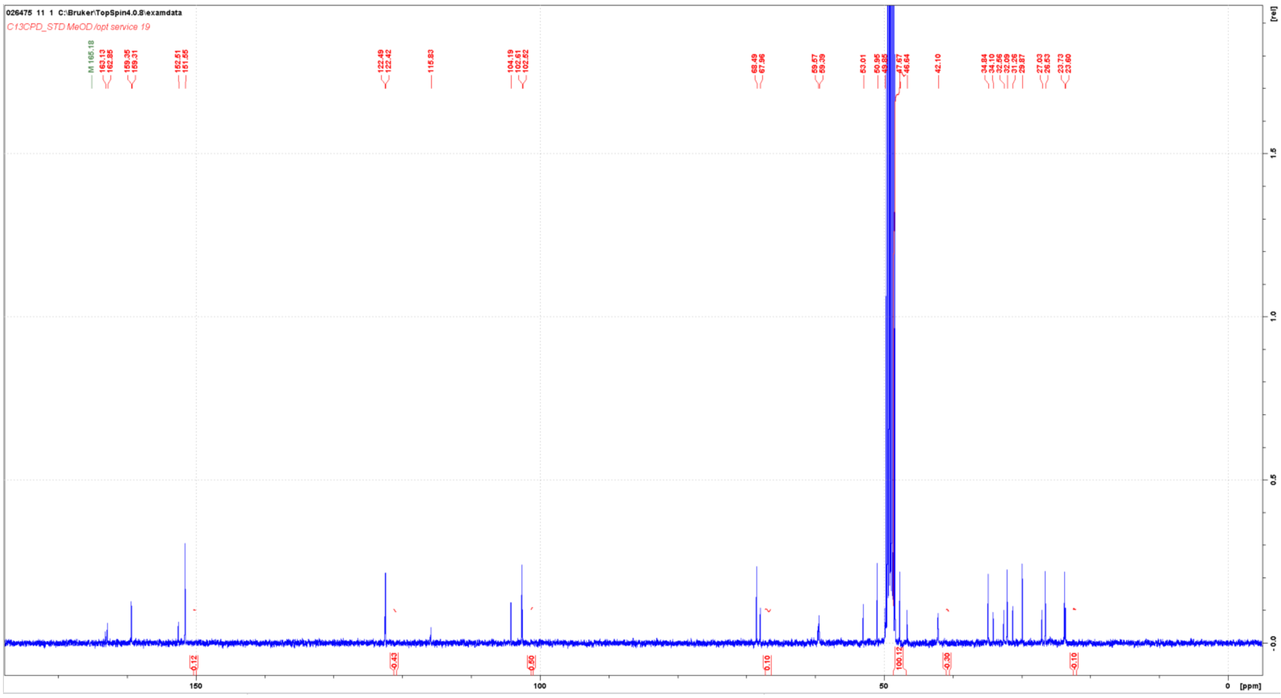

Compound (30)

^{1}H-NMR

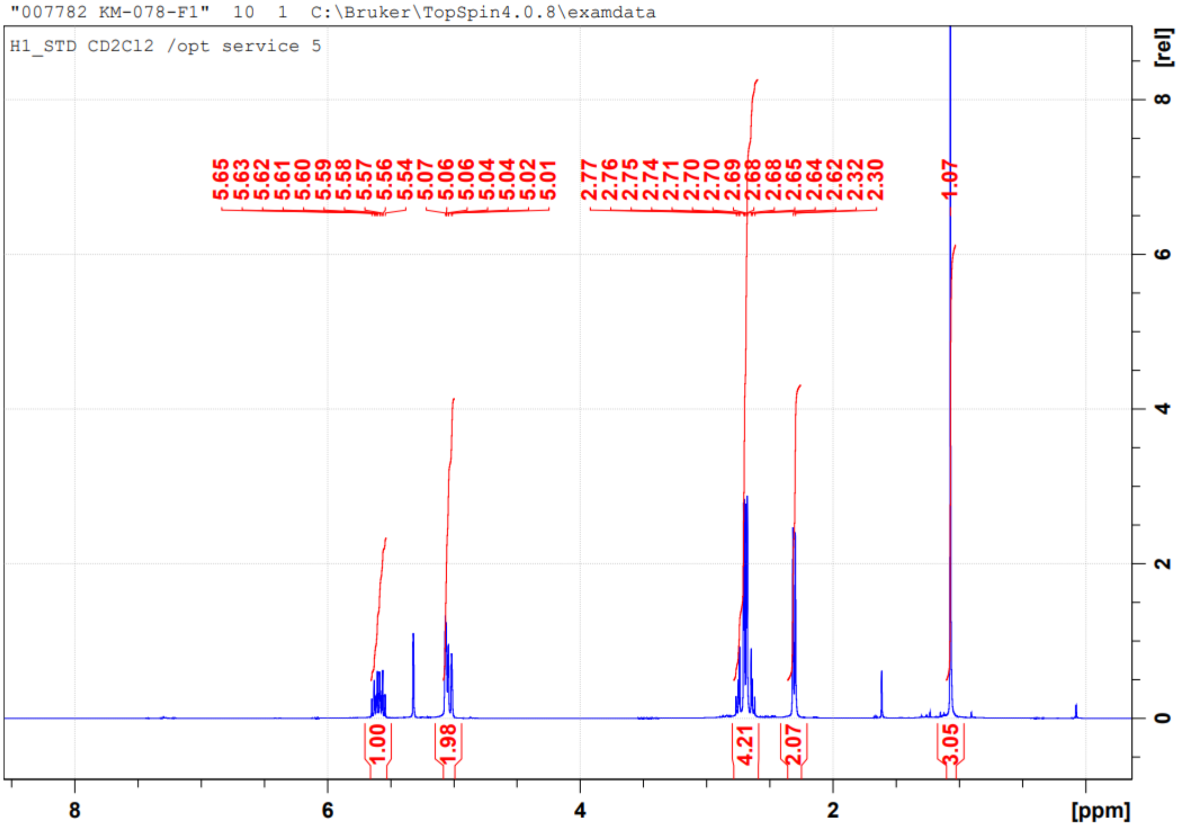

^{13}C-NMR

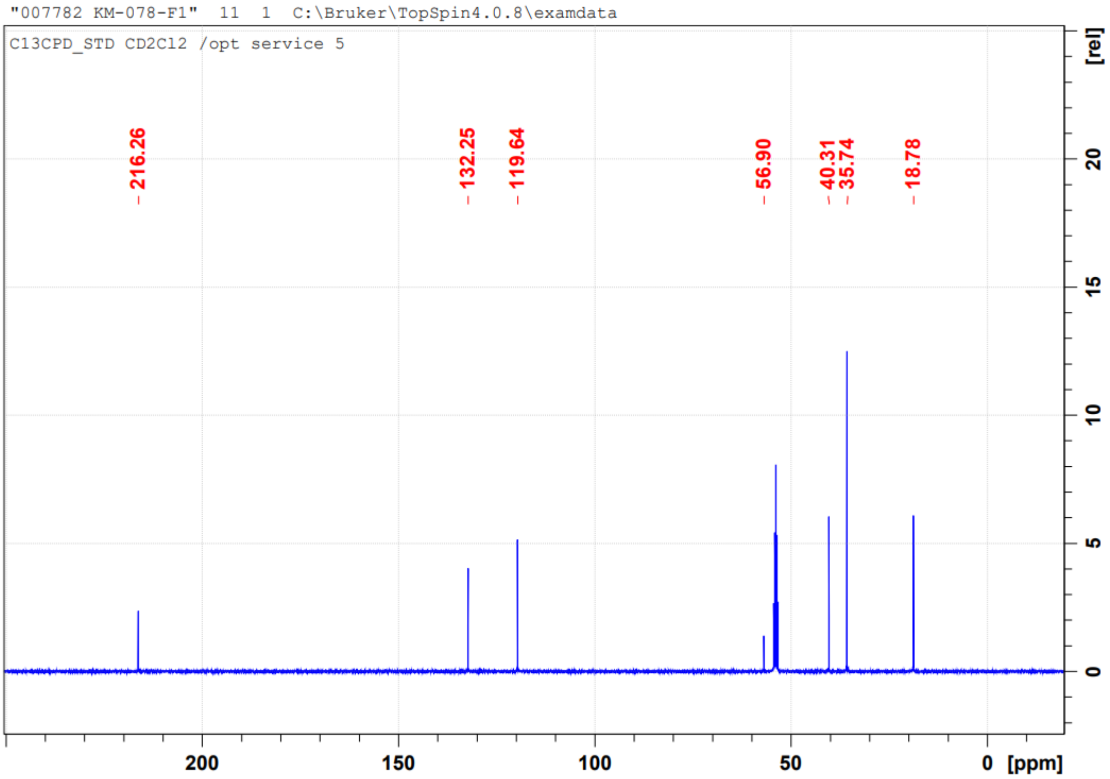

Compound 31

^{1}H-NMR

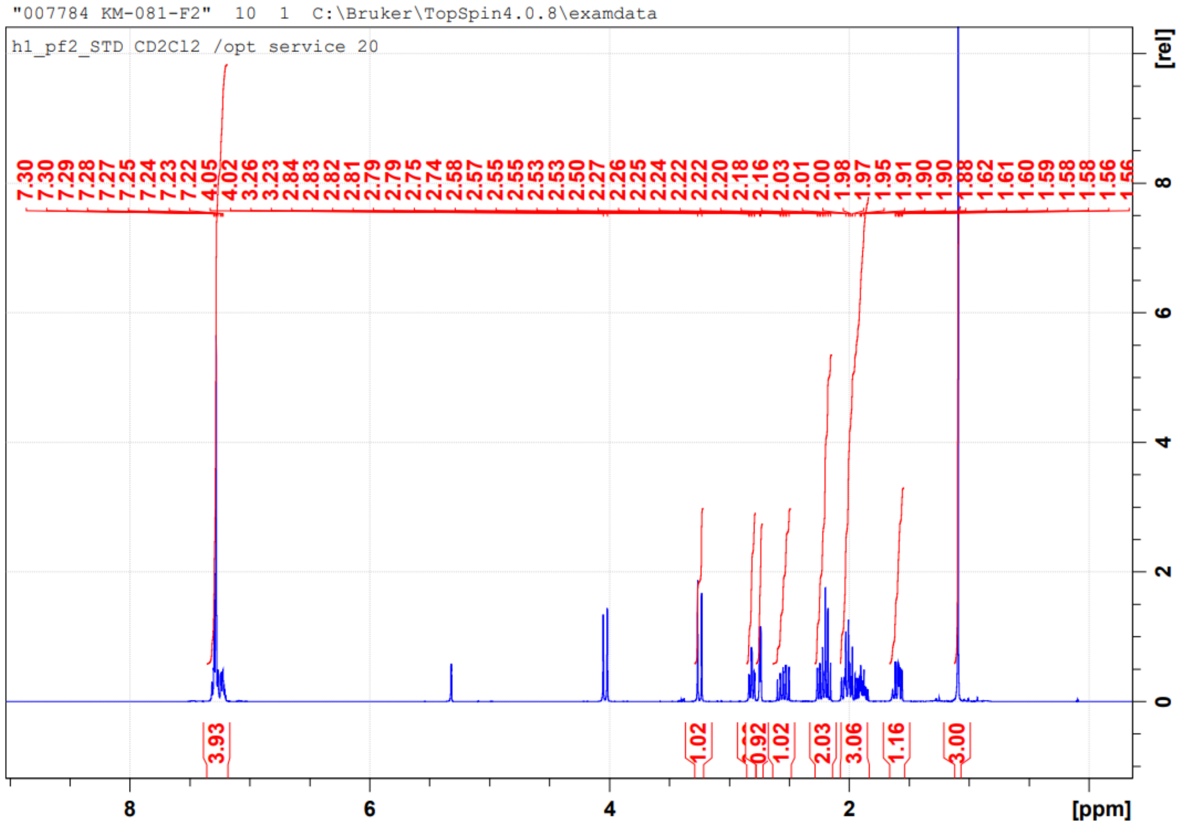

^{13}C-NMR

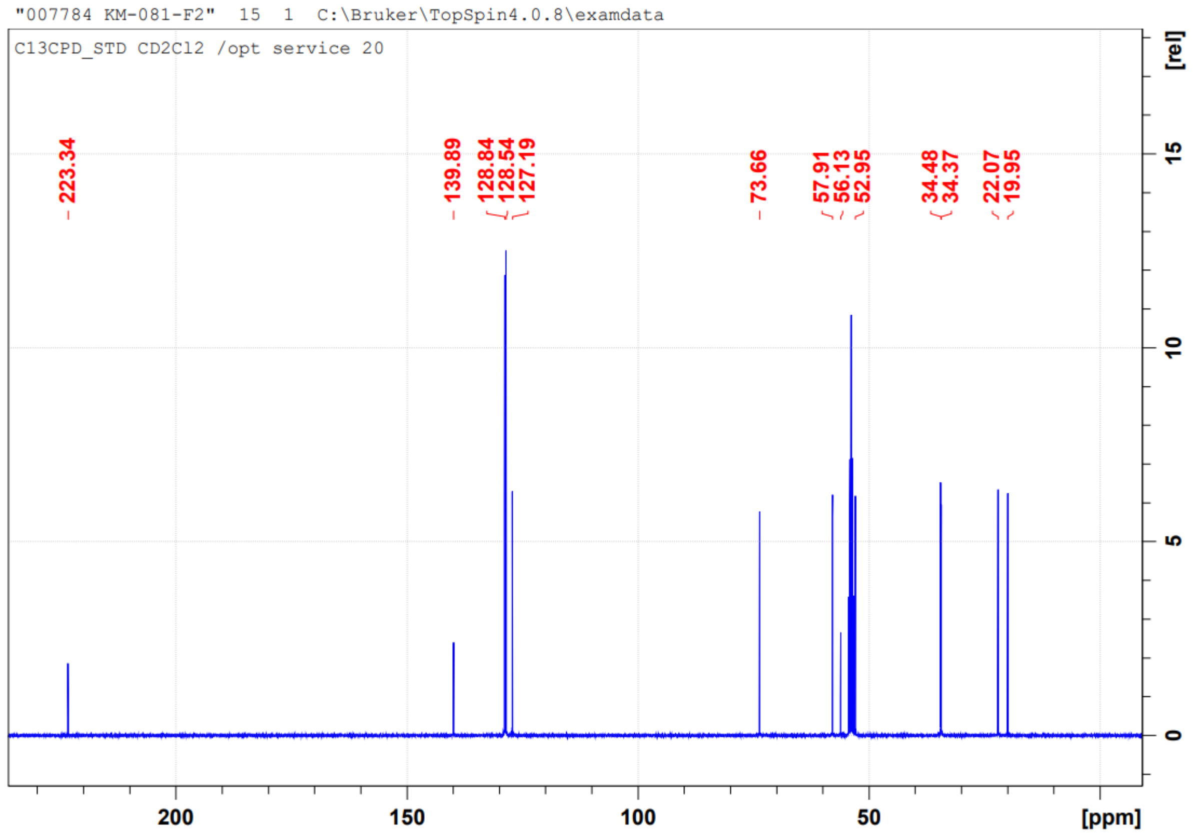

Compound 32

^{1}H-NMR

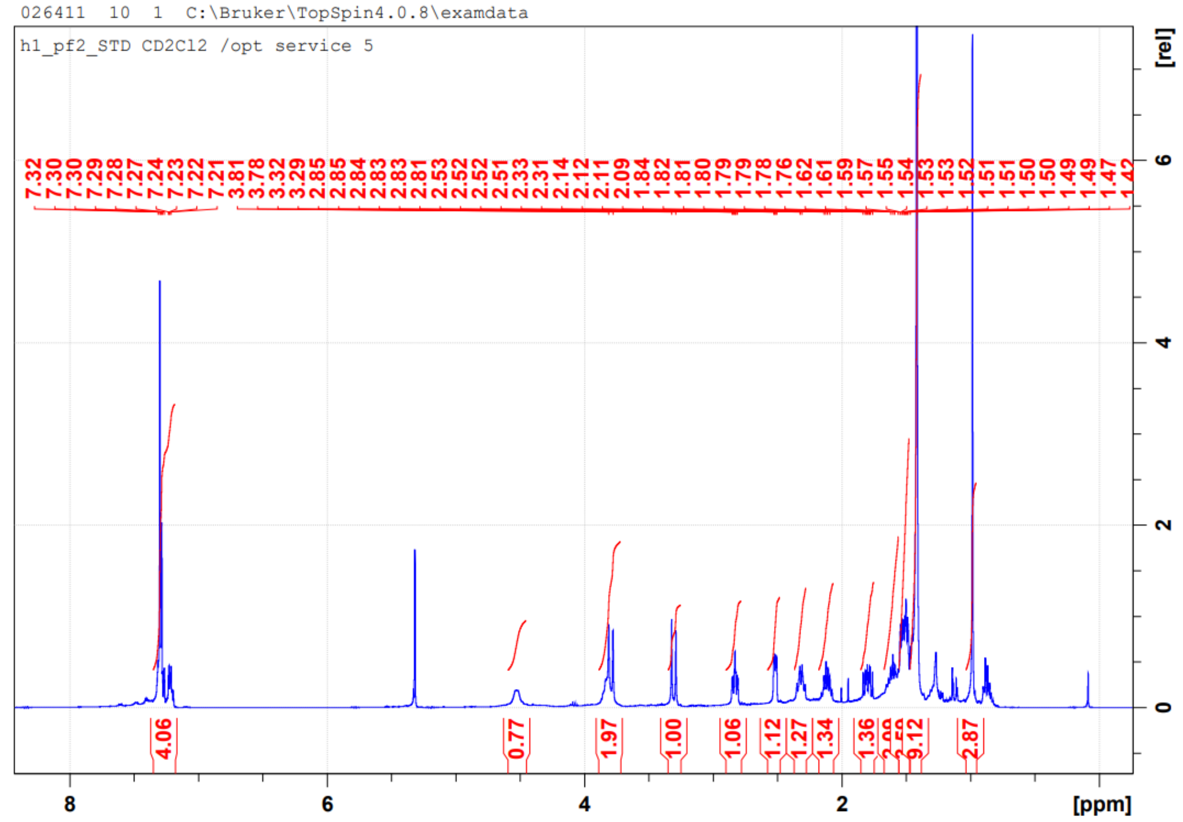

^{13}C-NMR

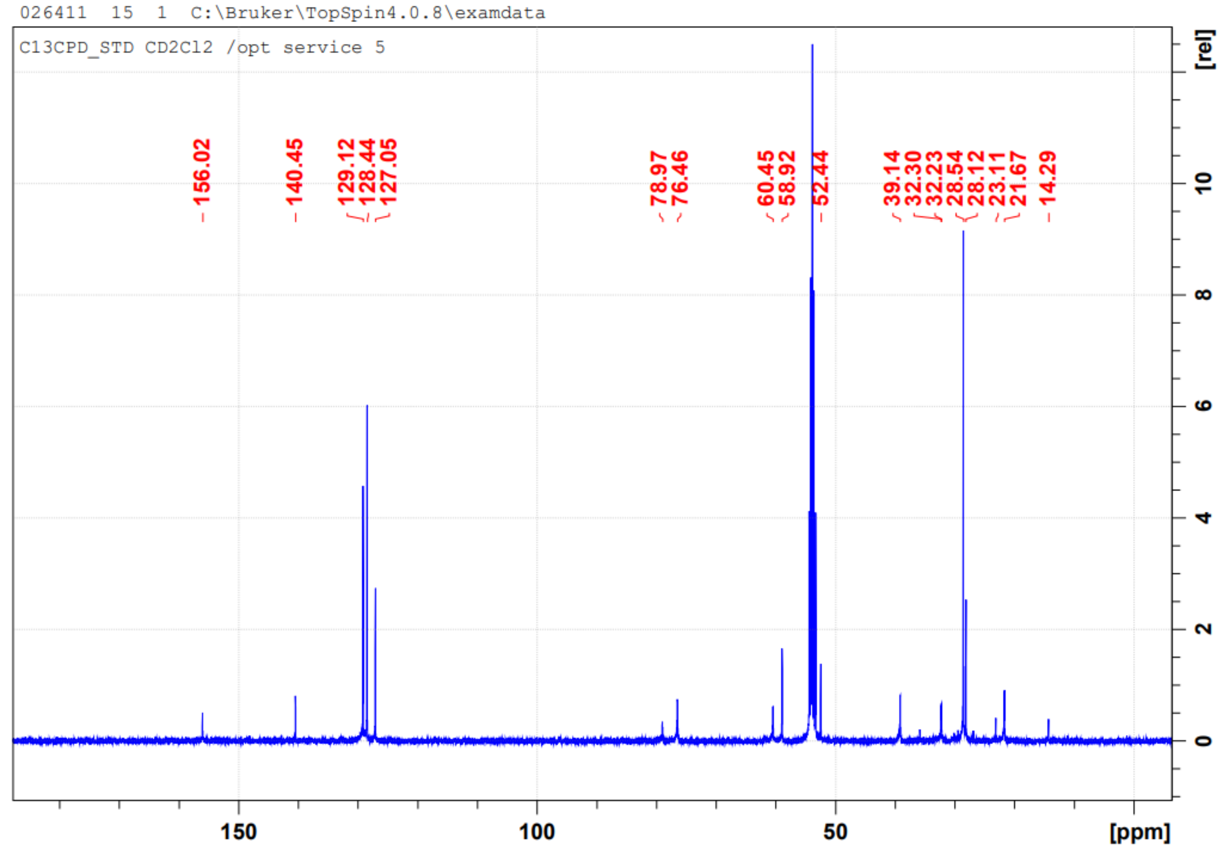

Compound 33

^{1}H-NMR

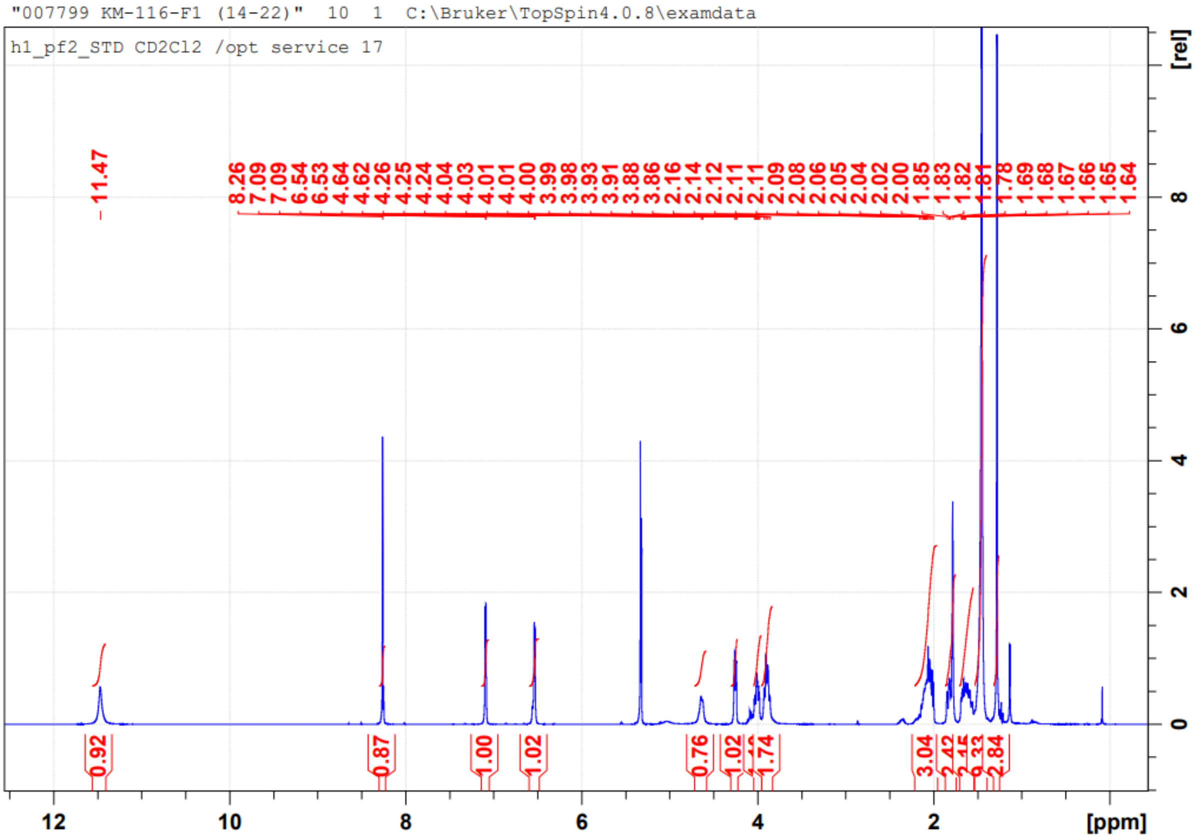

^{13}C-NMR

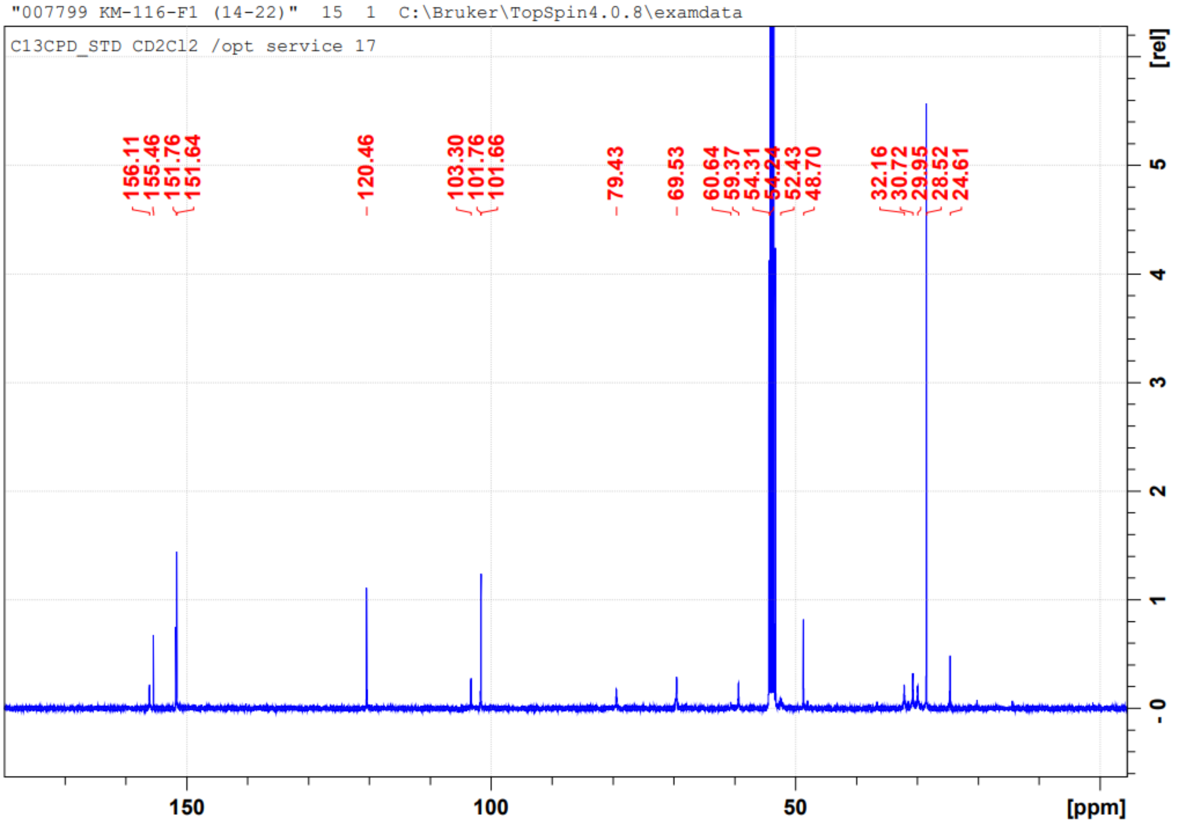

Compound 34a

^{1}H-NMR

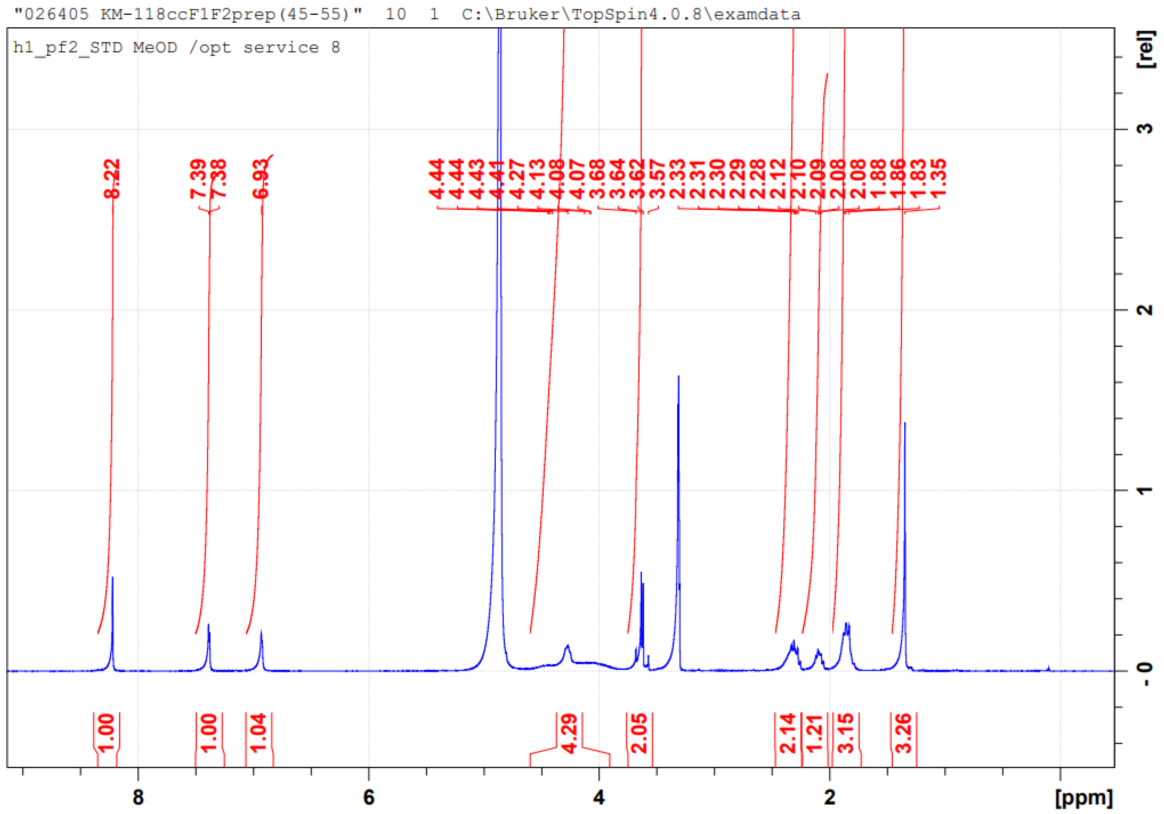

^{13}C-NMR

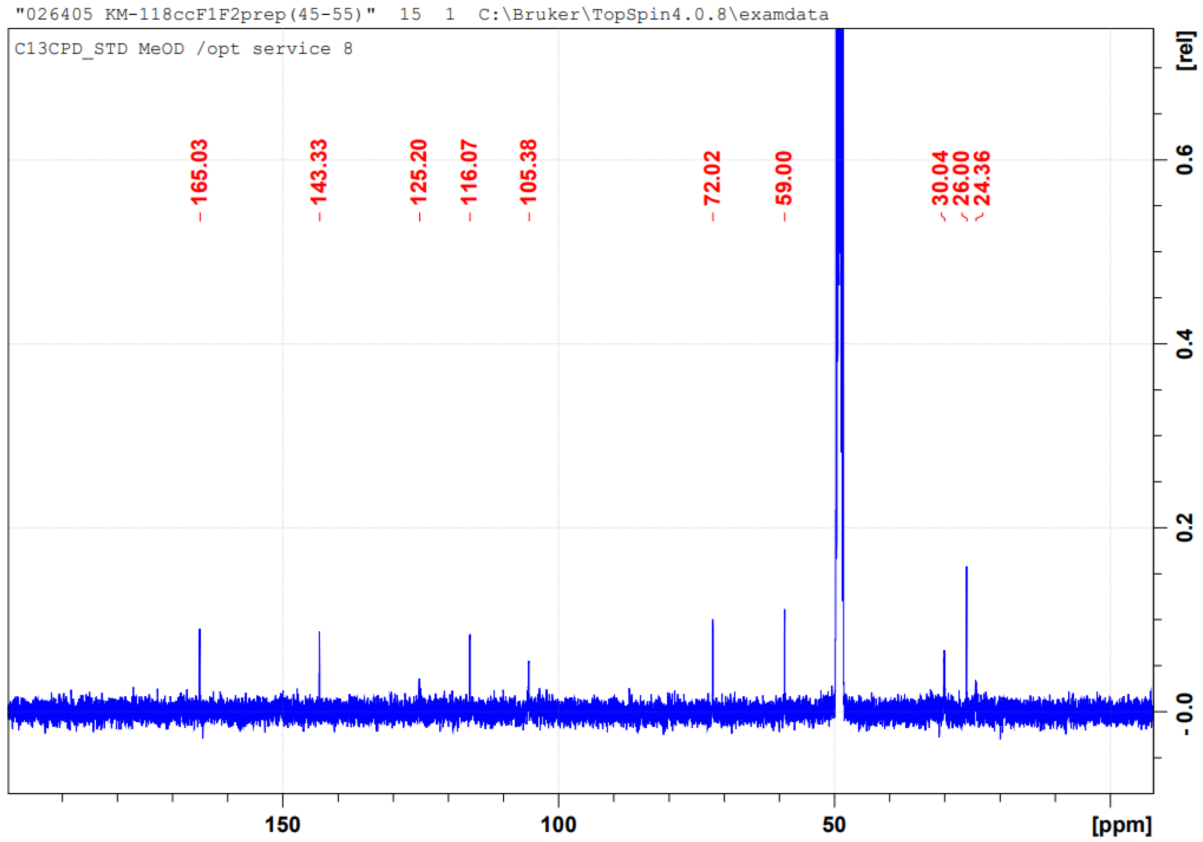

Compound 34b

^{1}H-NMR

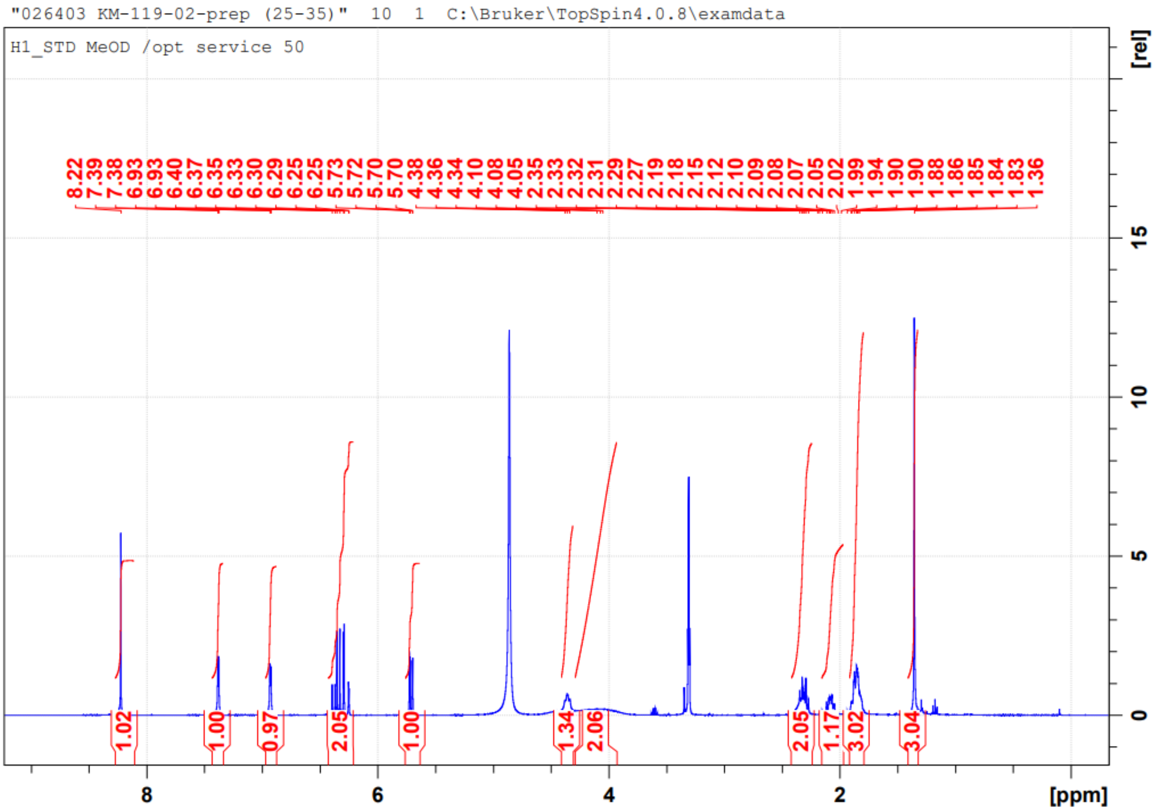

^{13}C-NMR

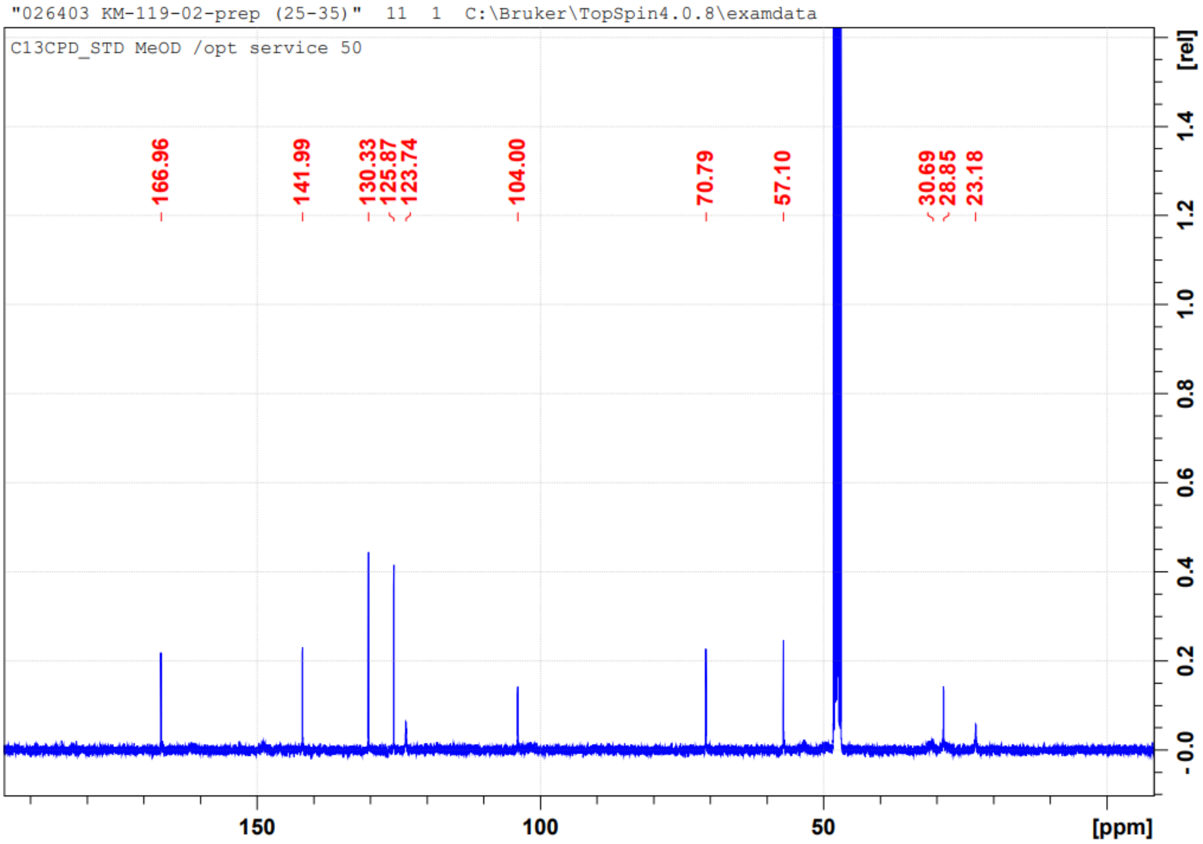

Compound 34c

^{1}H-NMR

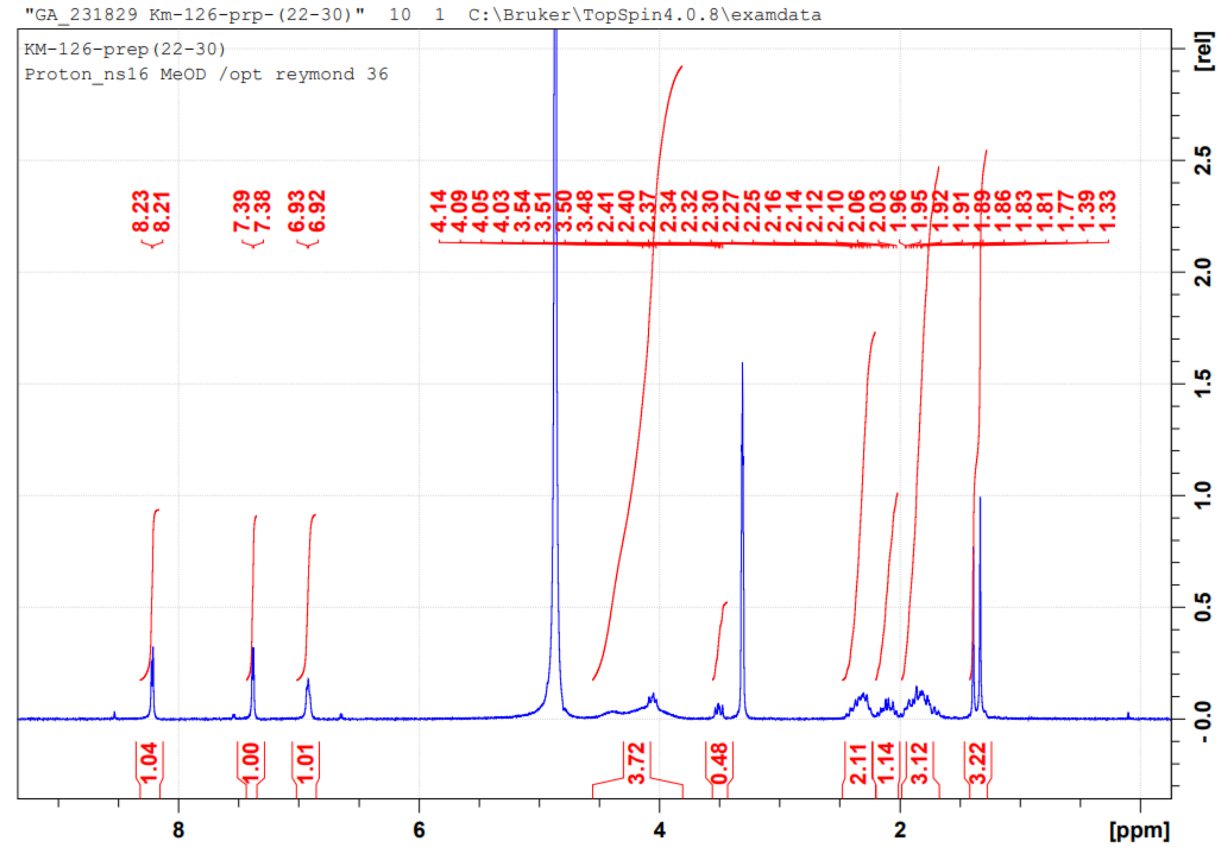

^{13}C- NMR

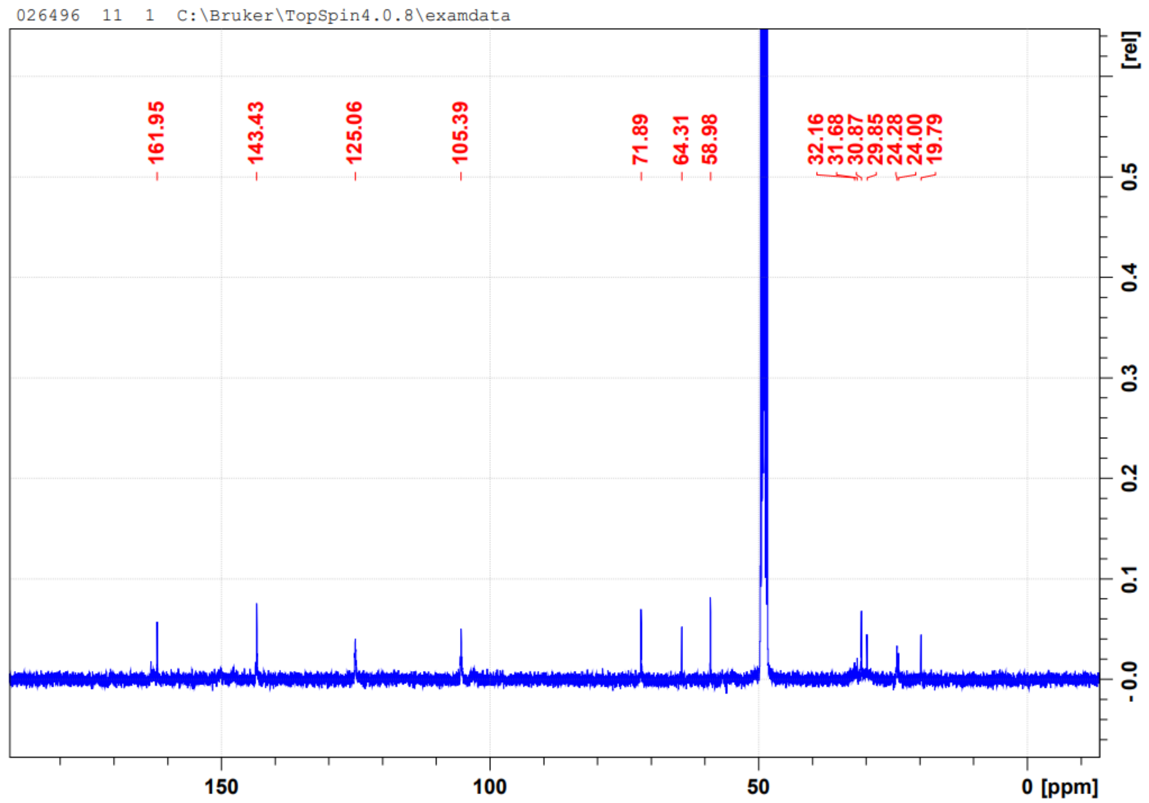

Compound 34d

^{1}H-NMR

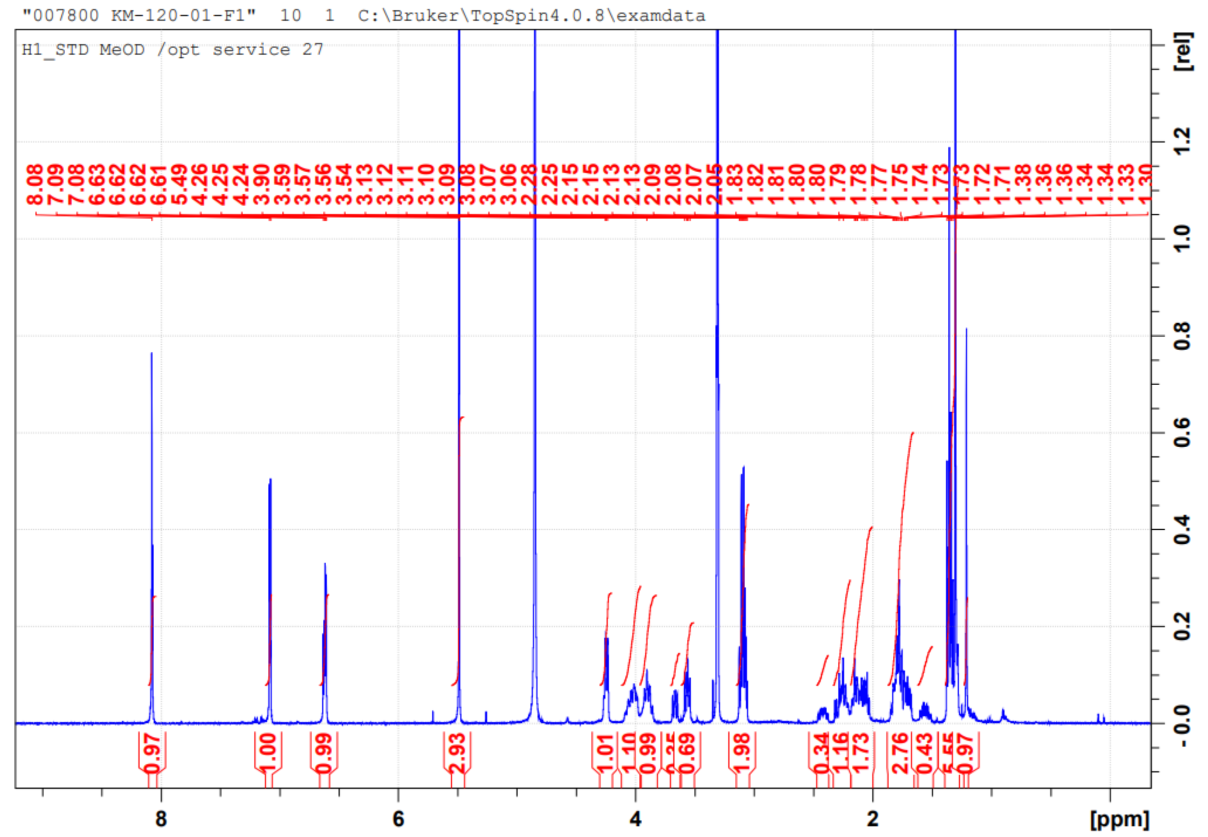

^{13}C-NMR

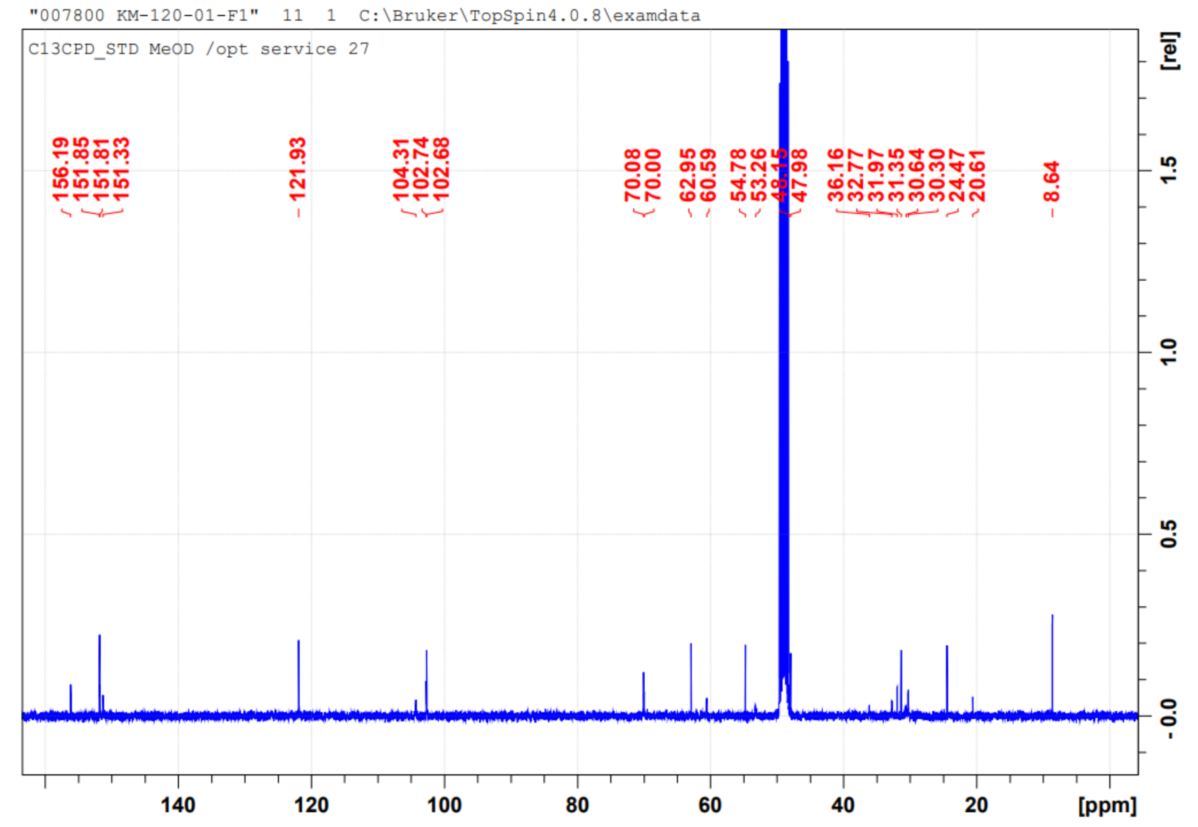

Compound 35

^{1}H-NMR

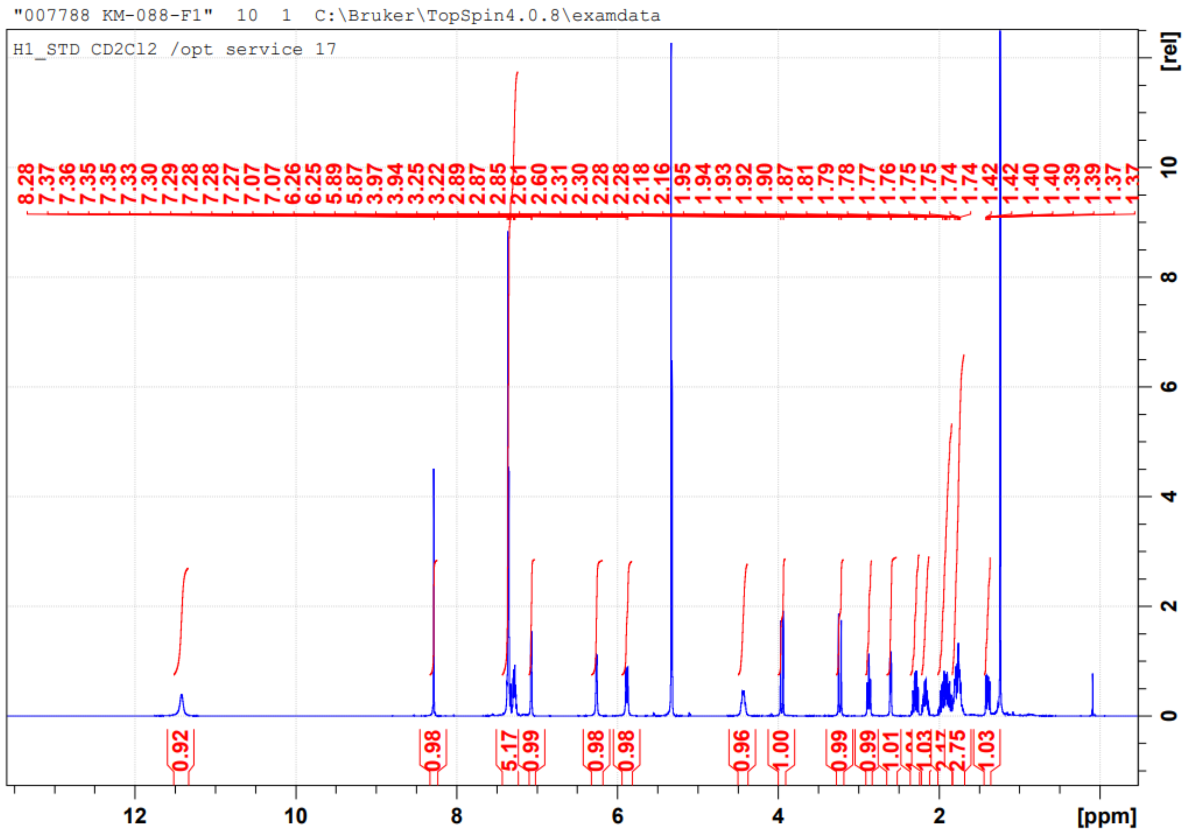

^{13}C-NMR

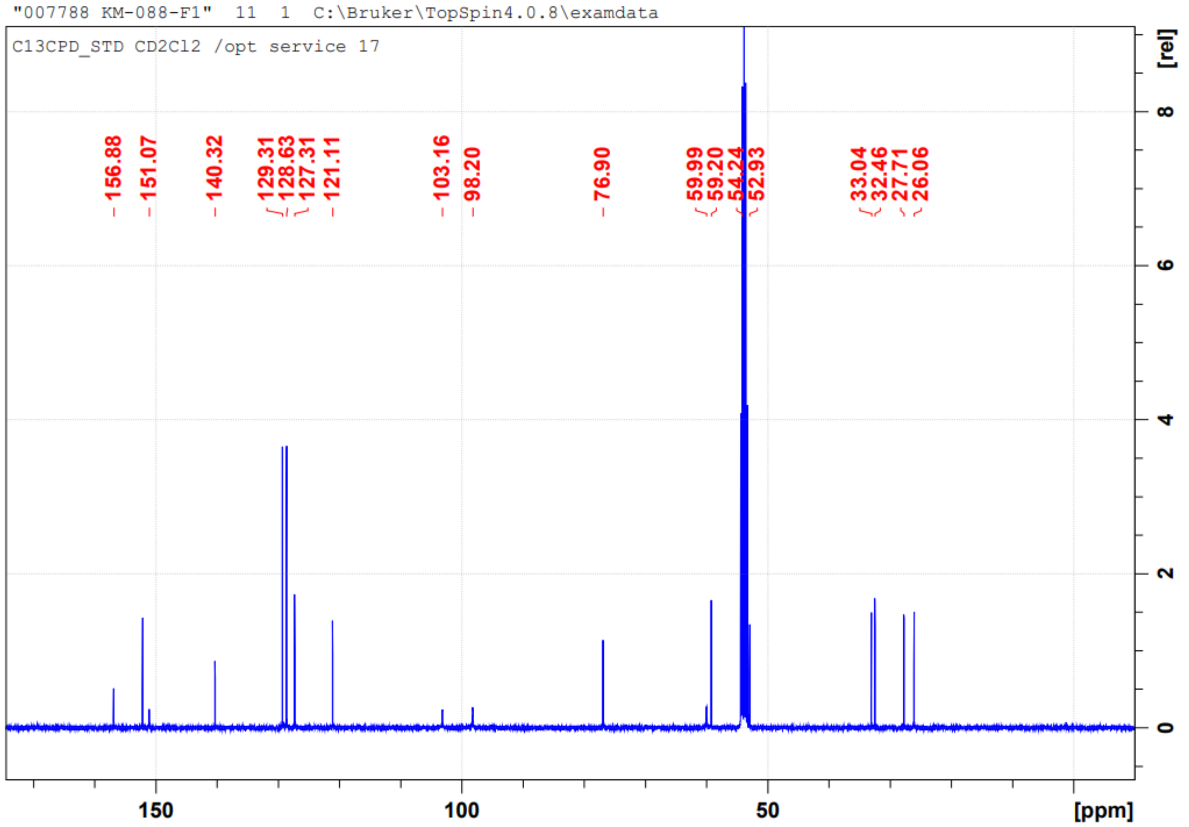

Compound 36a

^{1}H-NMR

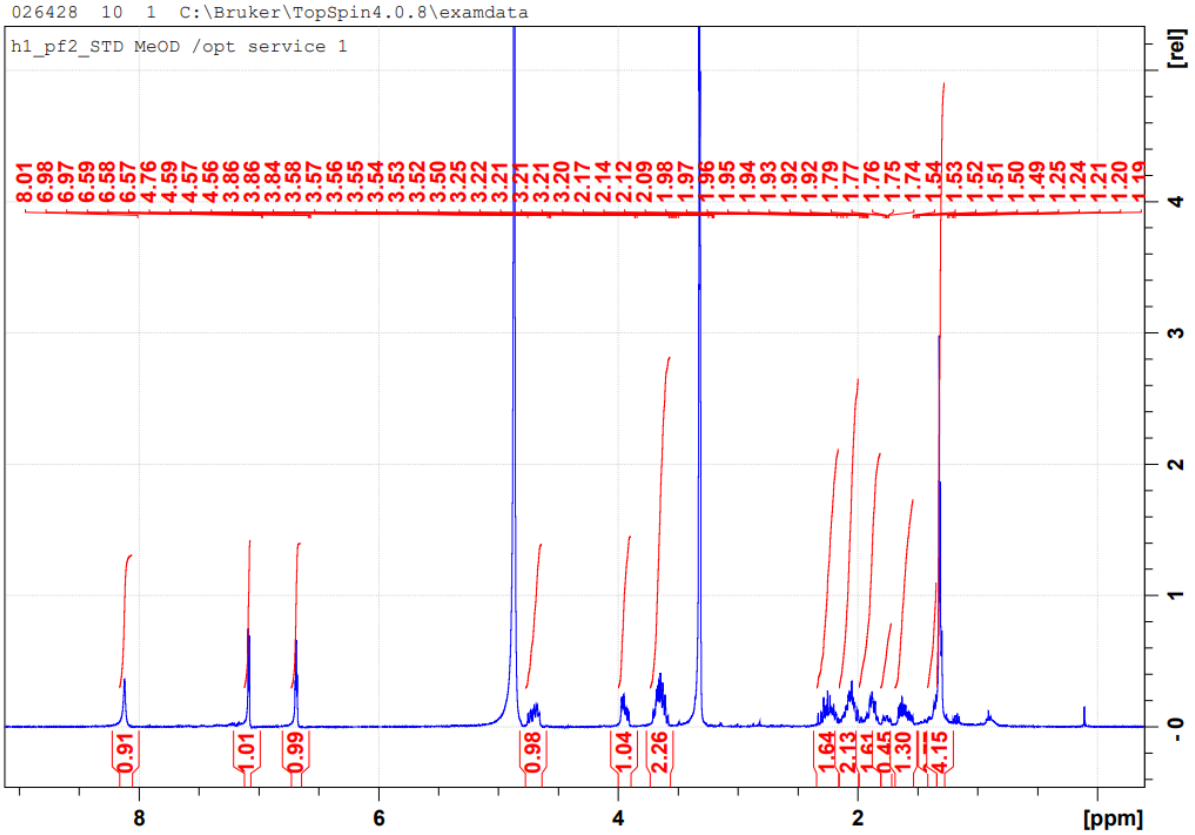

^{13}C-NMR

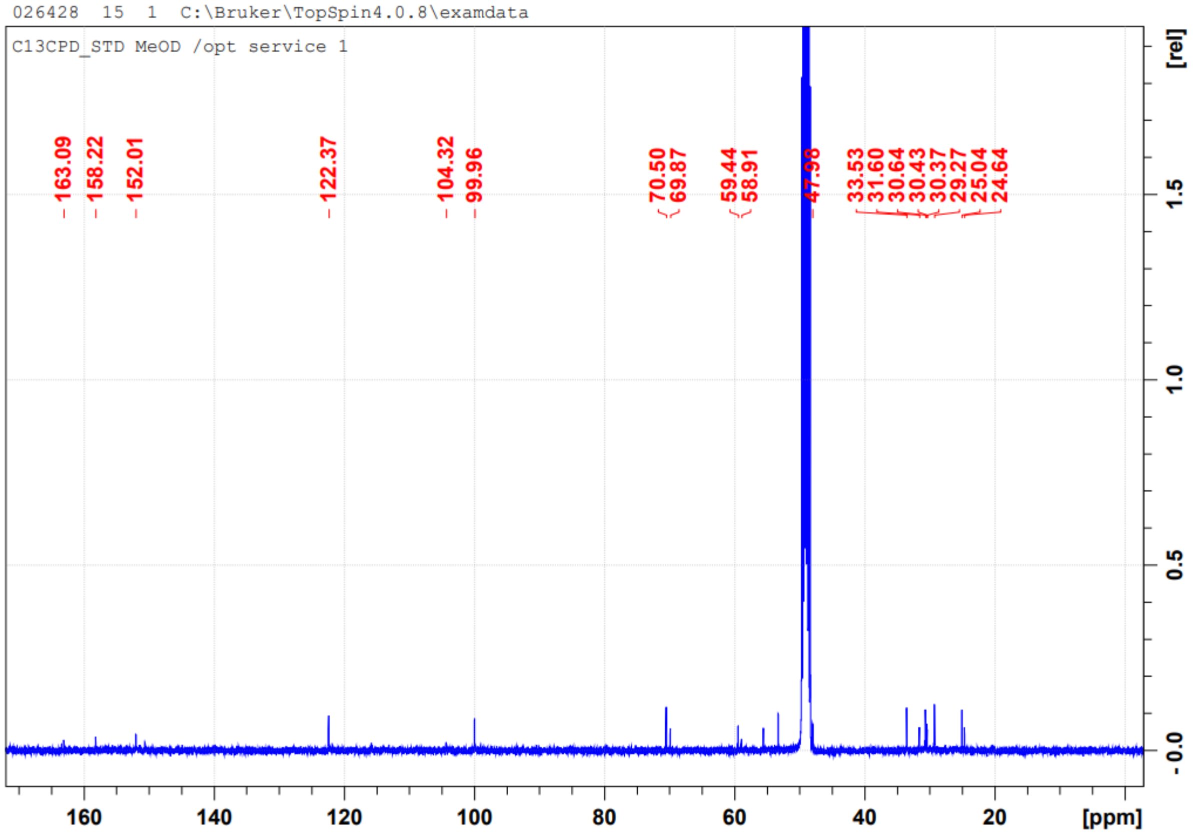

Compound 36b

^{1}H-NMR

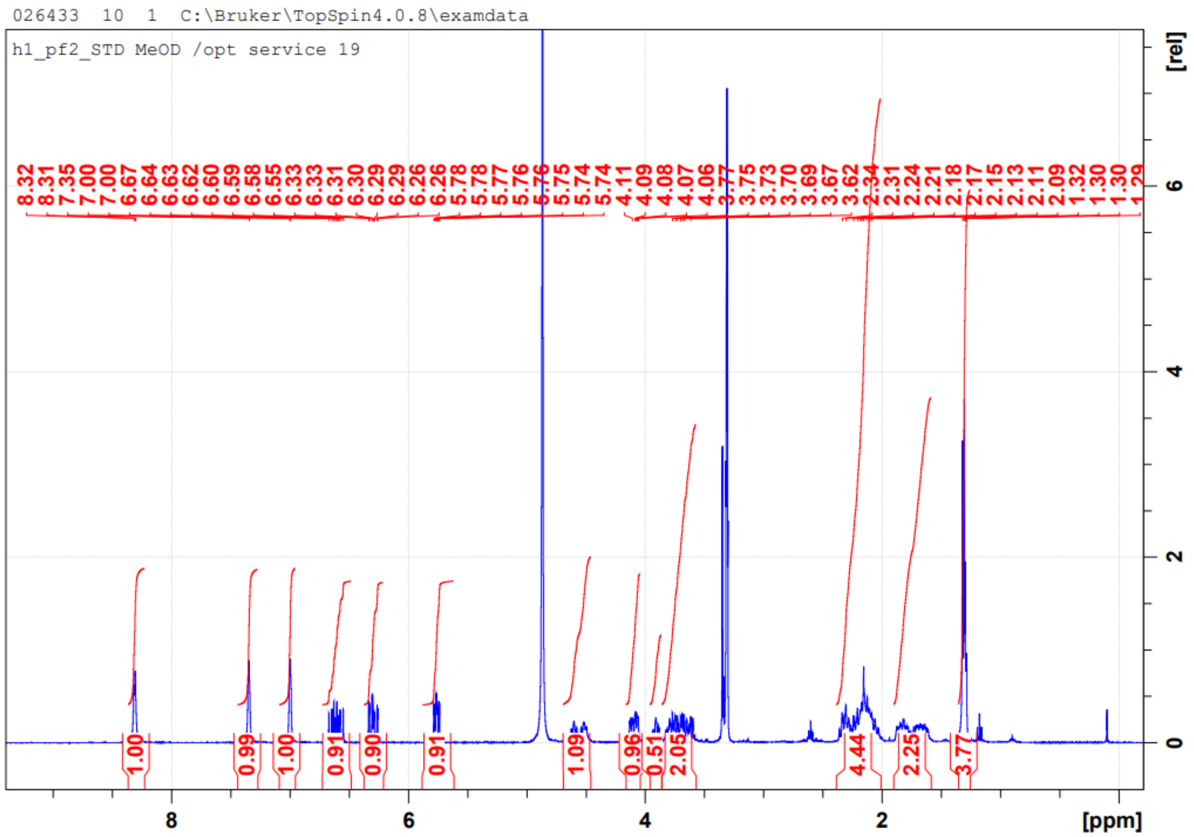

^{13}C-NMR

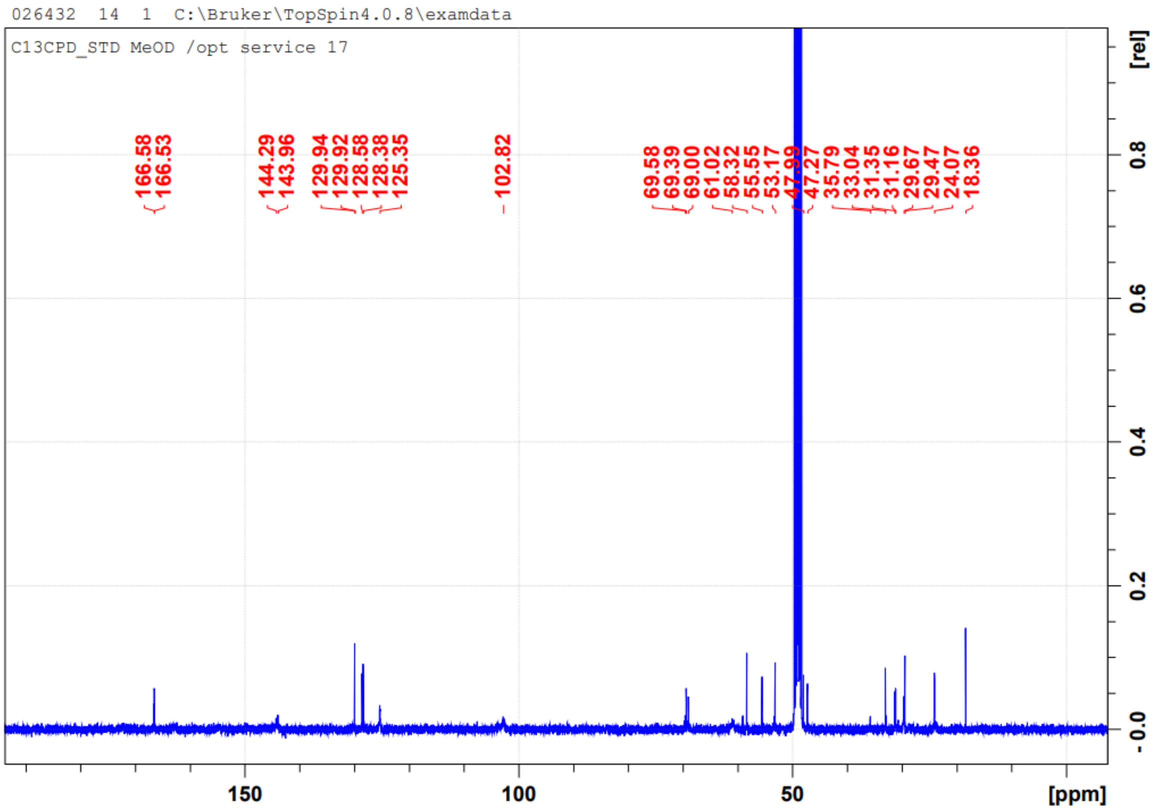

Compound 36c

^{1}H-NMR

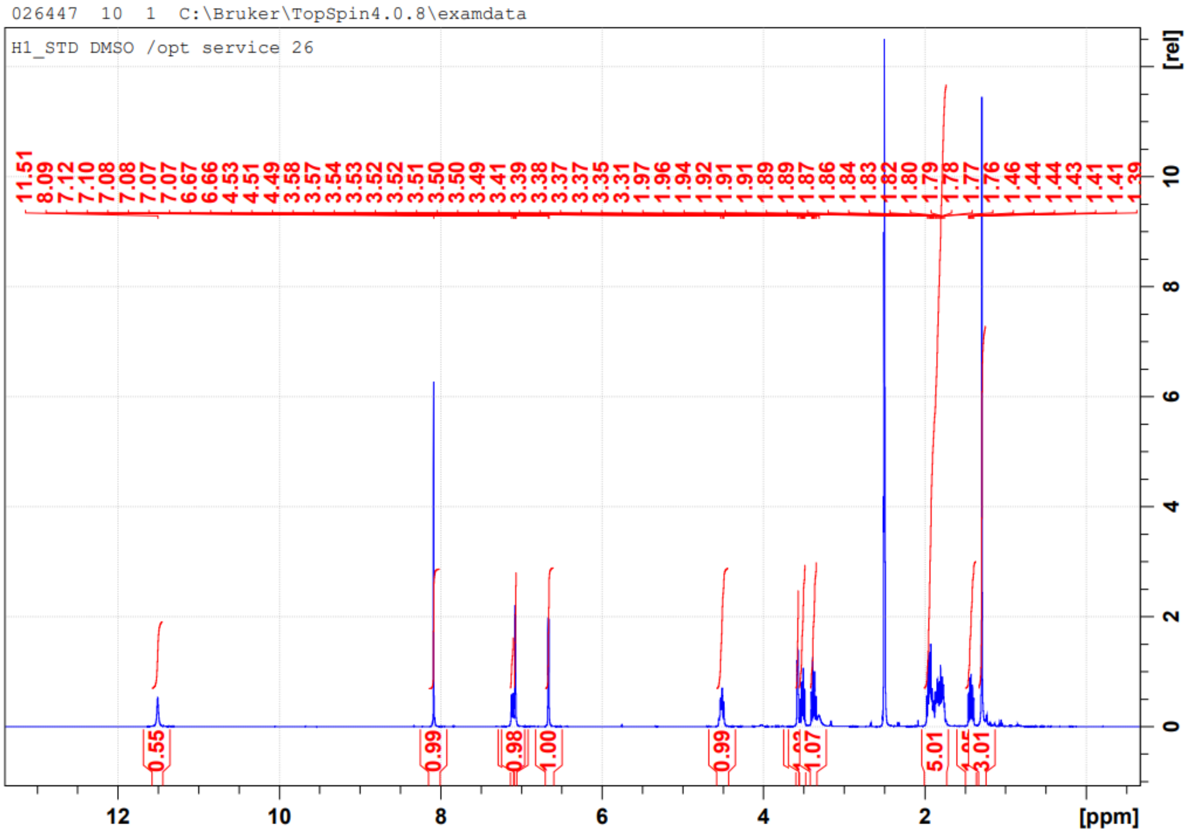

^{13}C-NMR

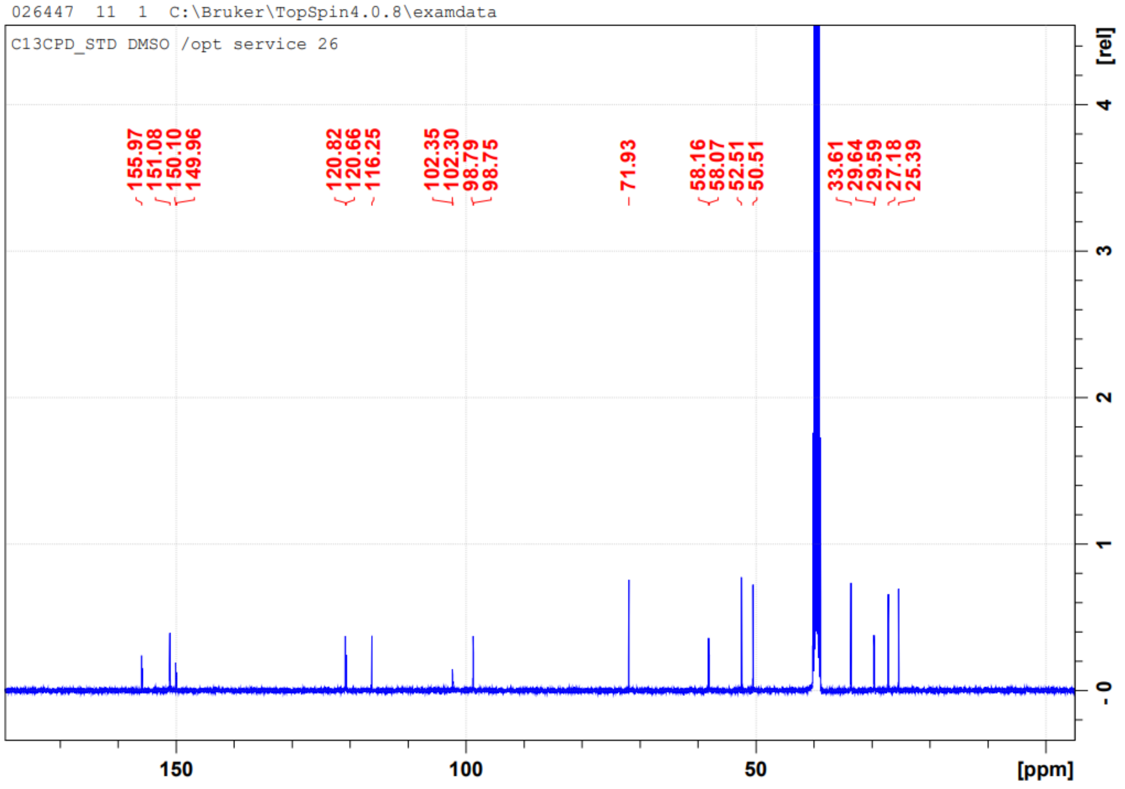

Compound 36d

¹H-NMR

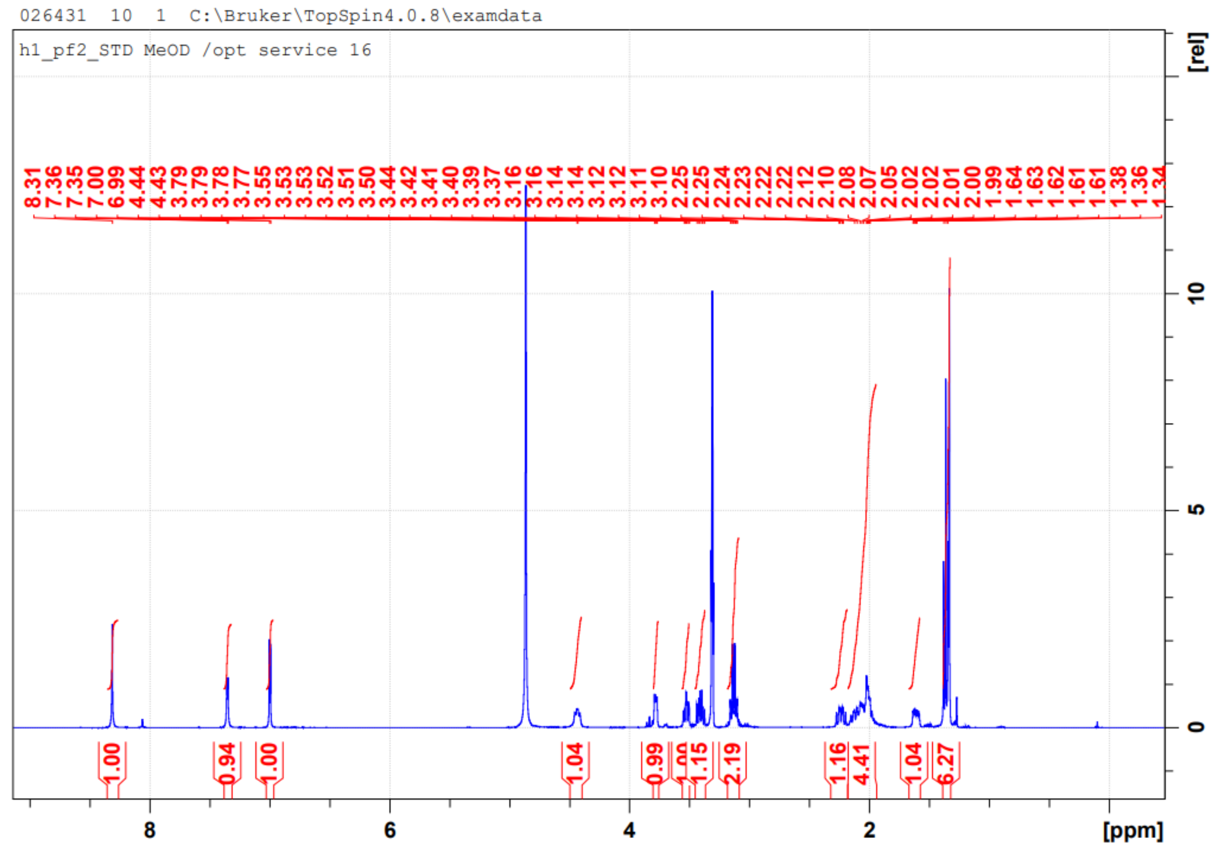

¹³C-NMR

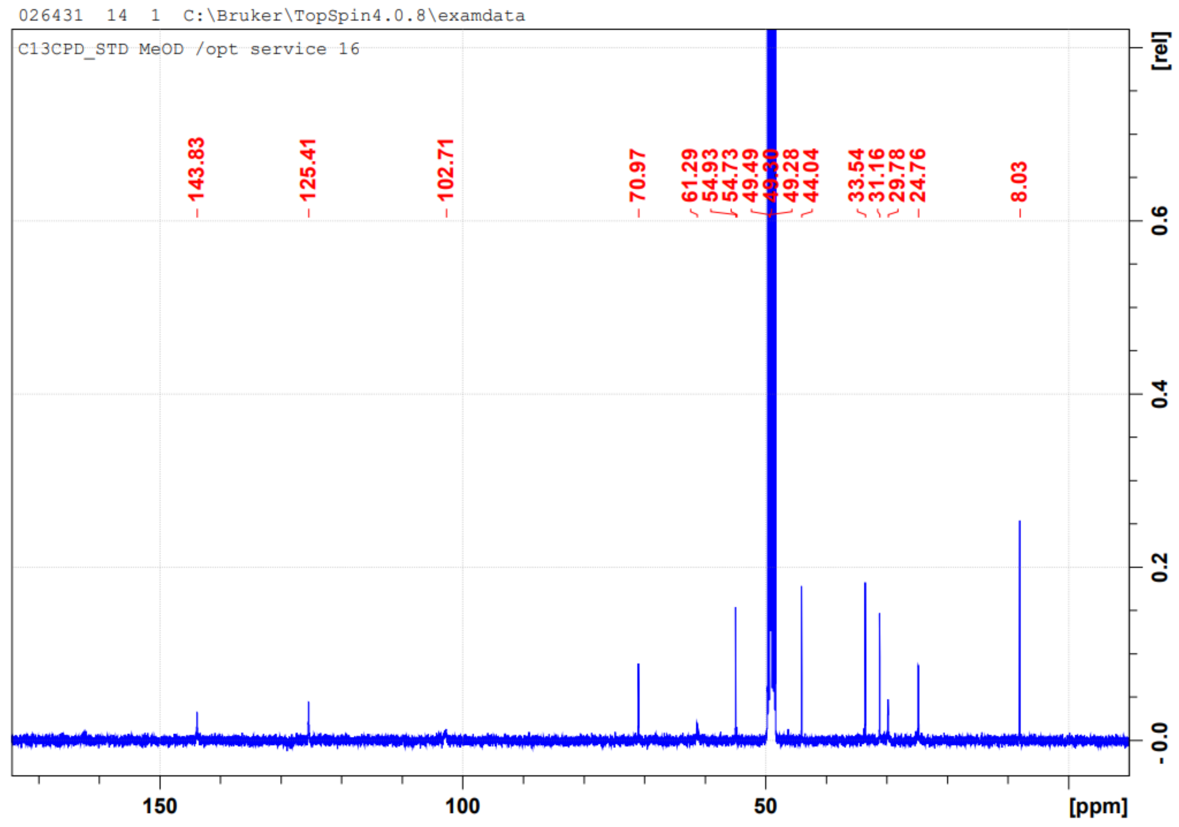

Compound 37

¹H-NMR

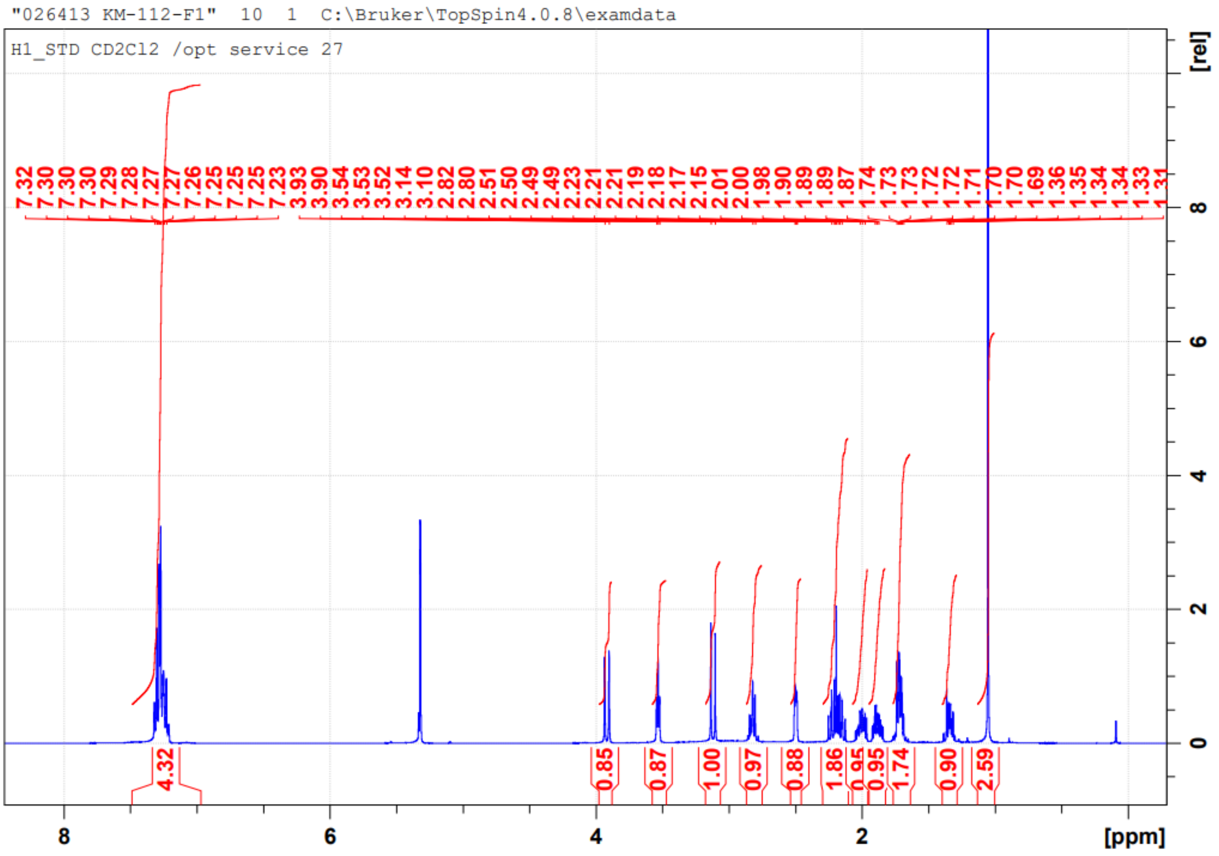

¹³C-NMR

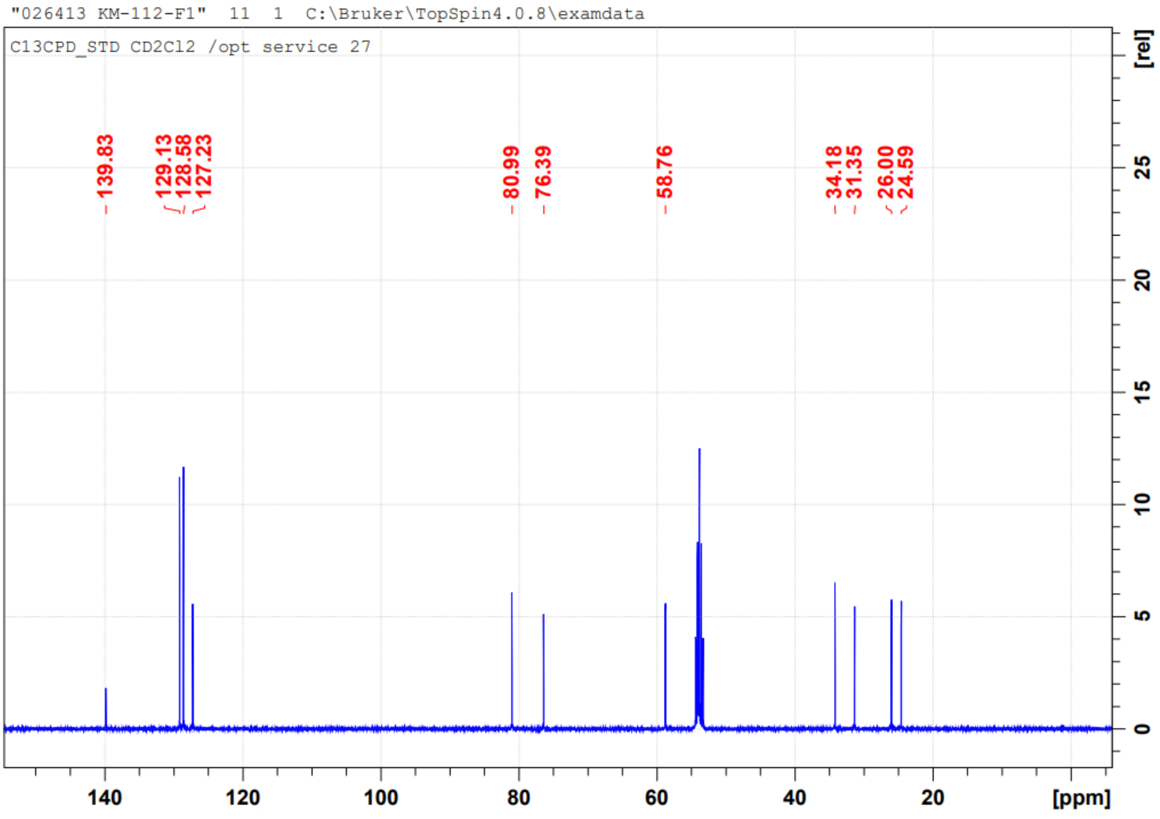

Compound 38

^{1}H-NMR

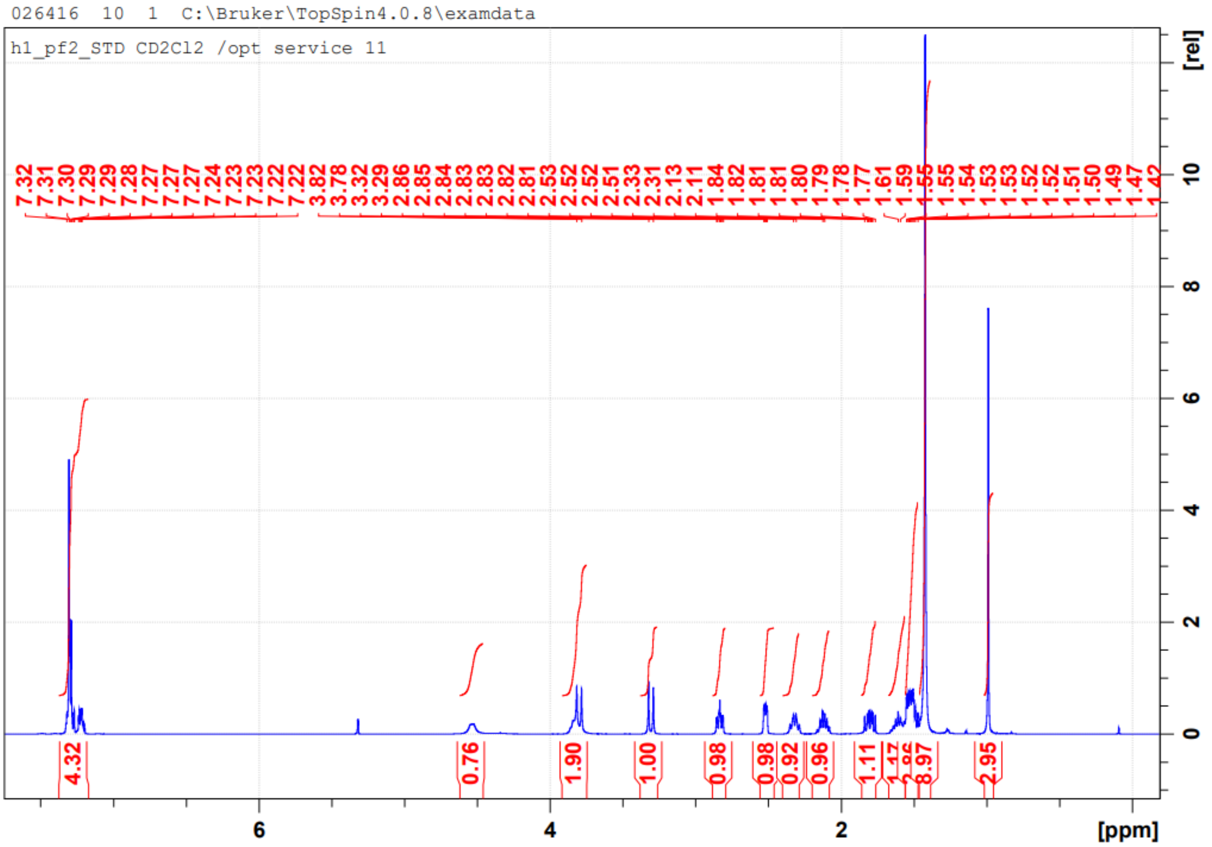

^{13}C-NMR

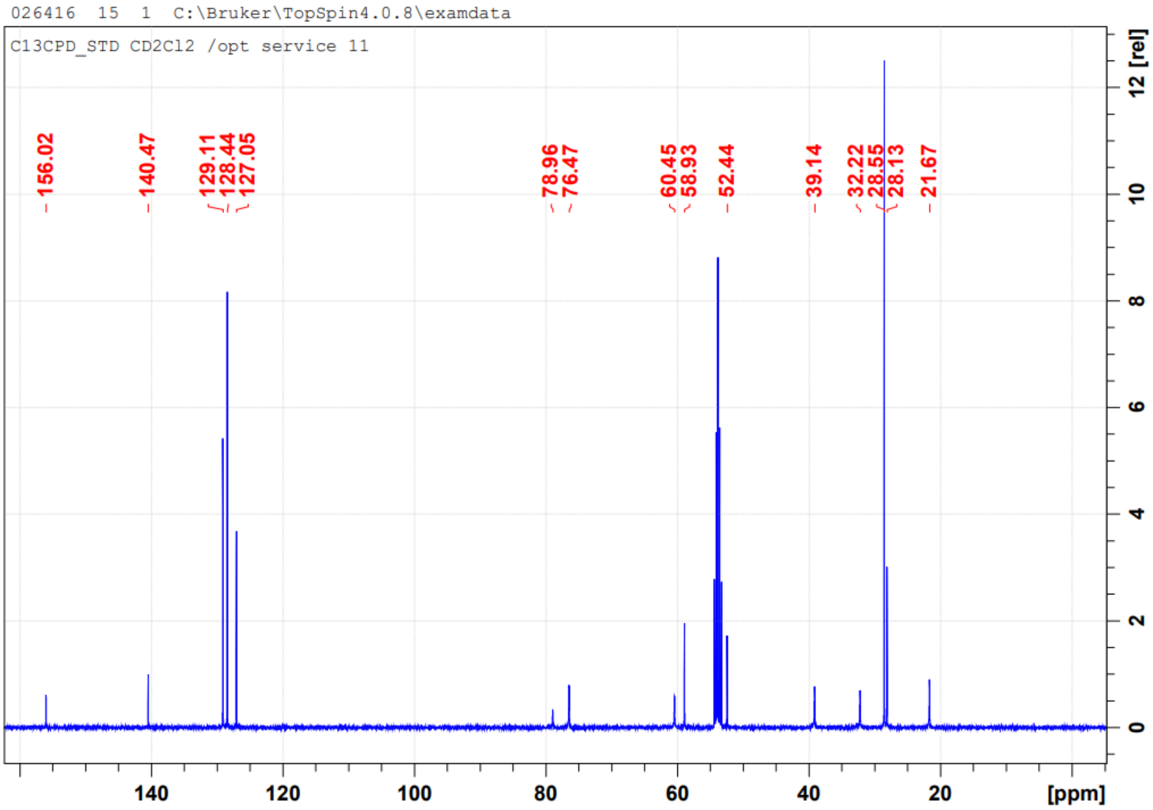

Compound 39

^{1}H-NMR

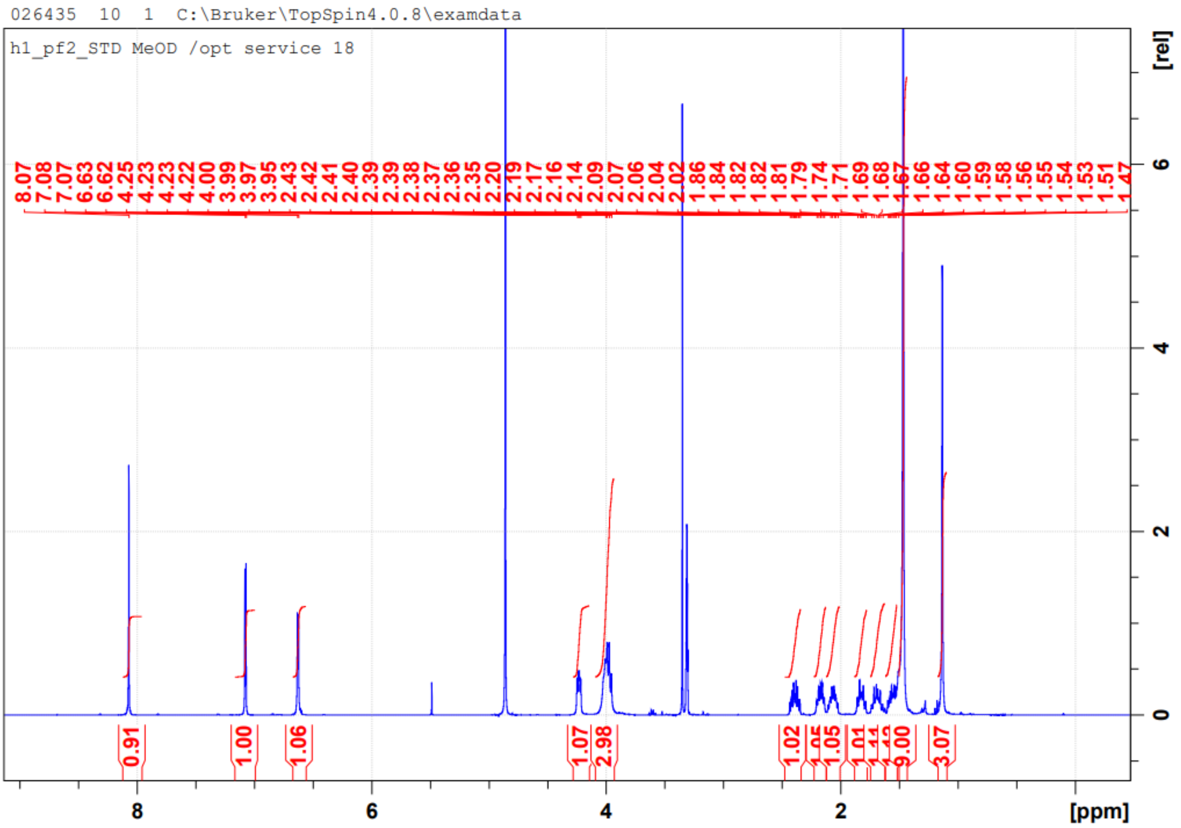

^{13}C-NMR

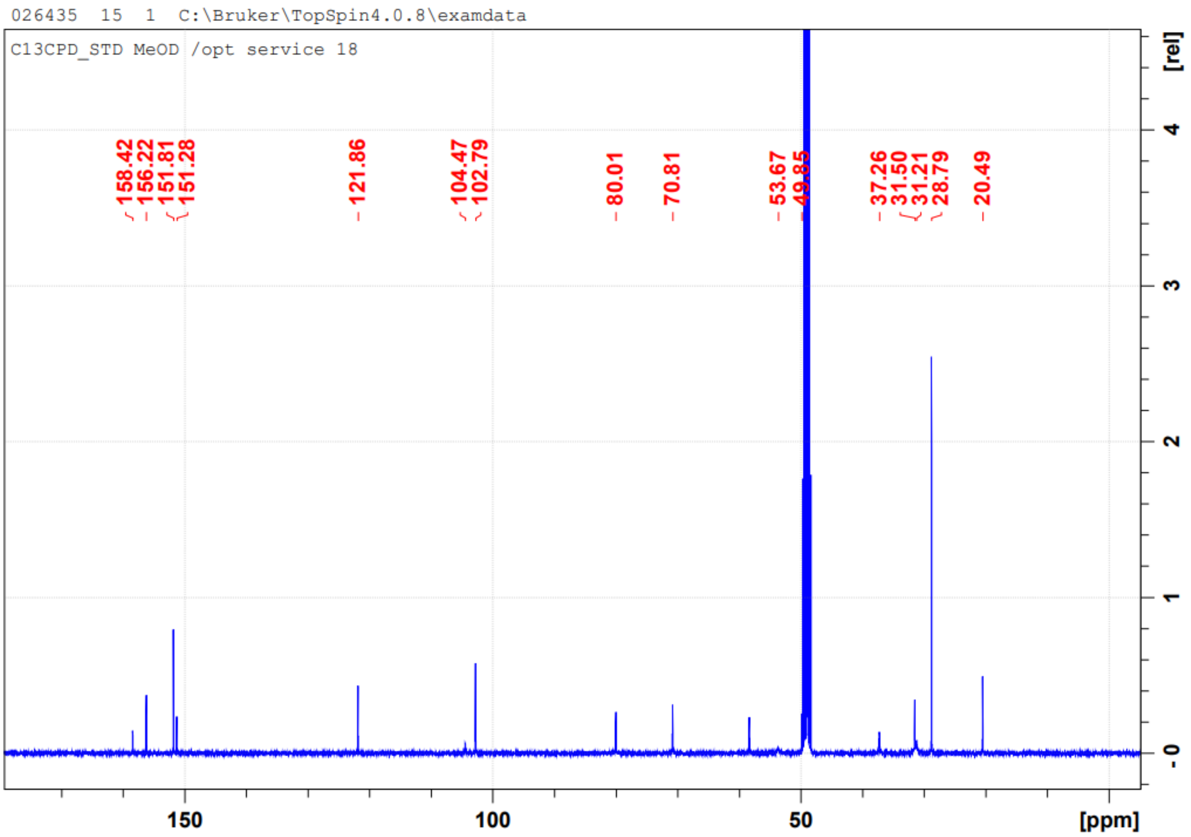

Compound 40a

¹H-NMR

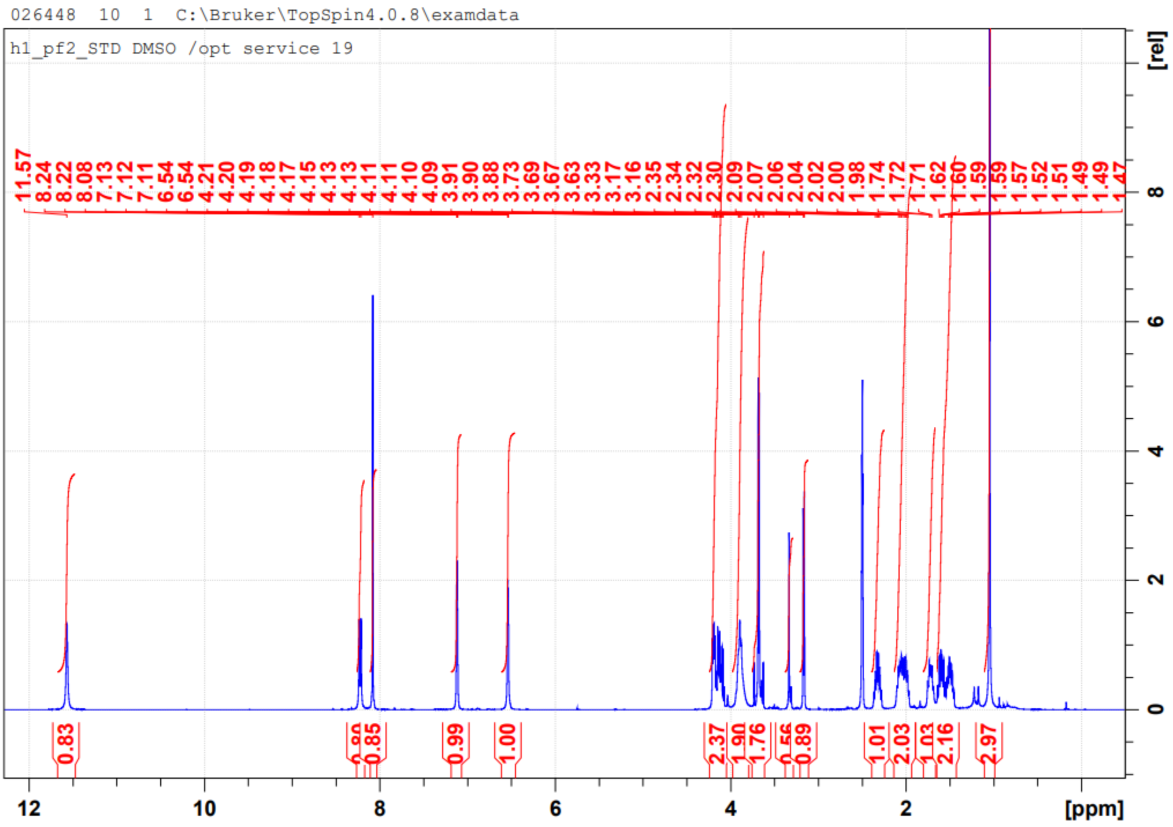

¹³C-NMR

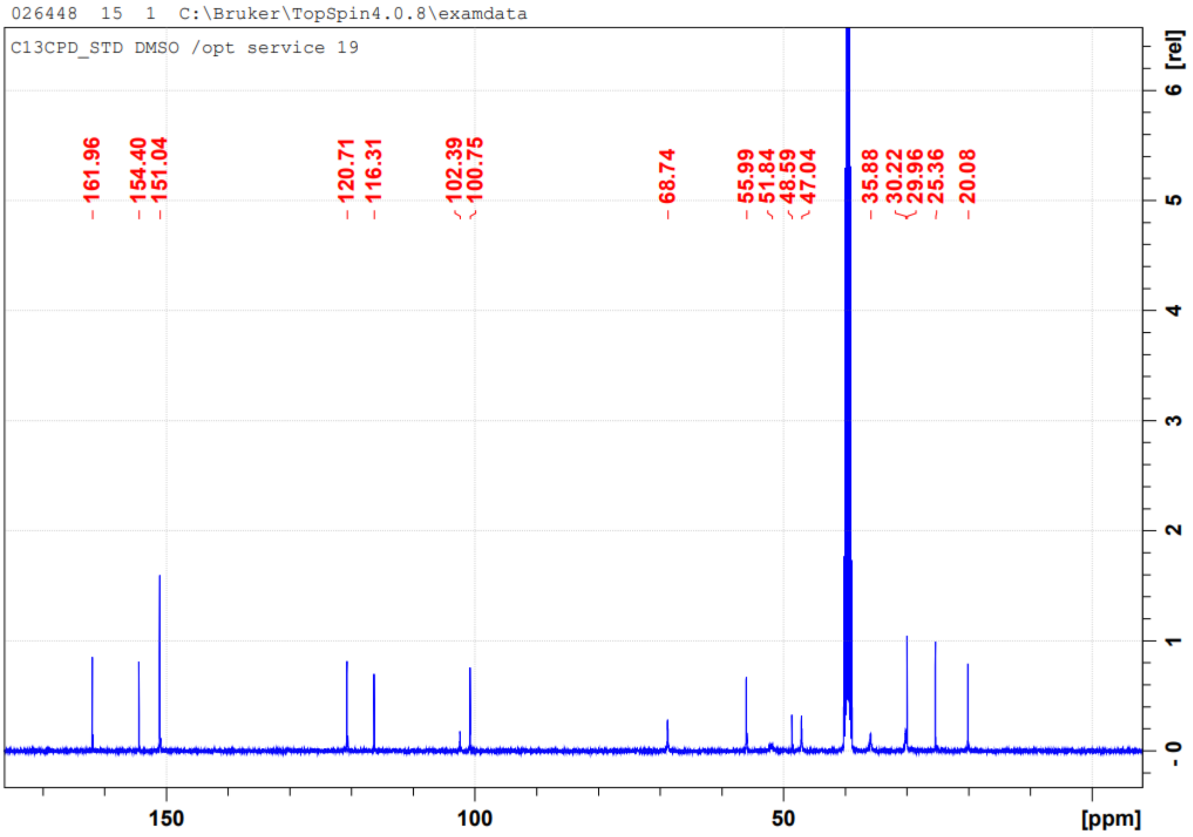

Compound 40b

^{1}H-NMR

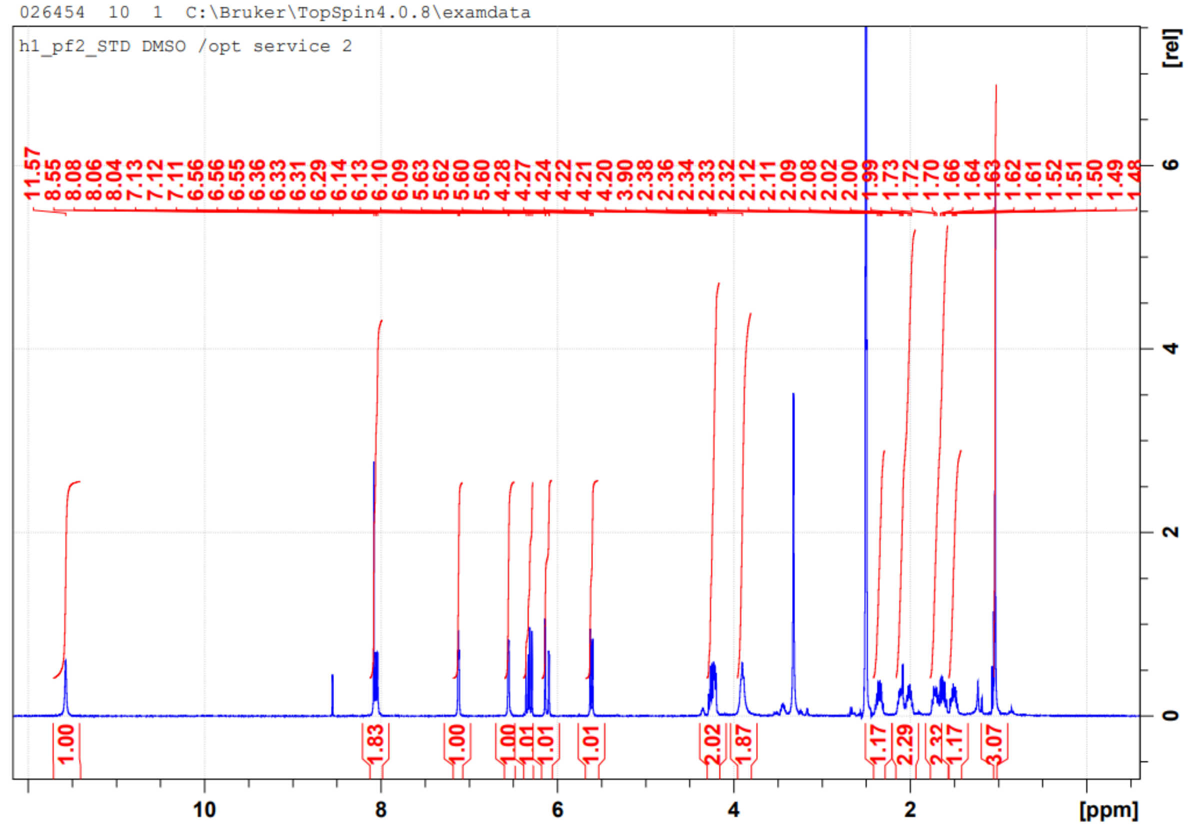

^{13}C-NMR

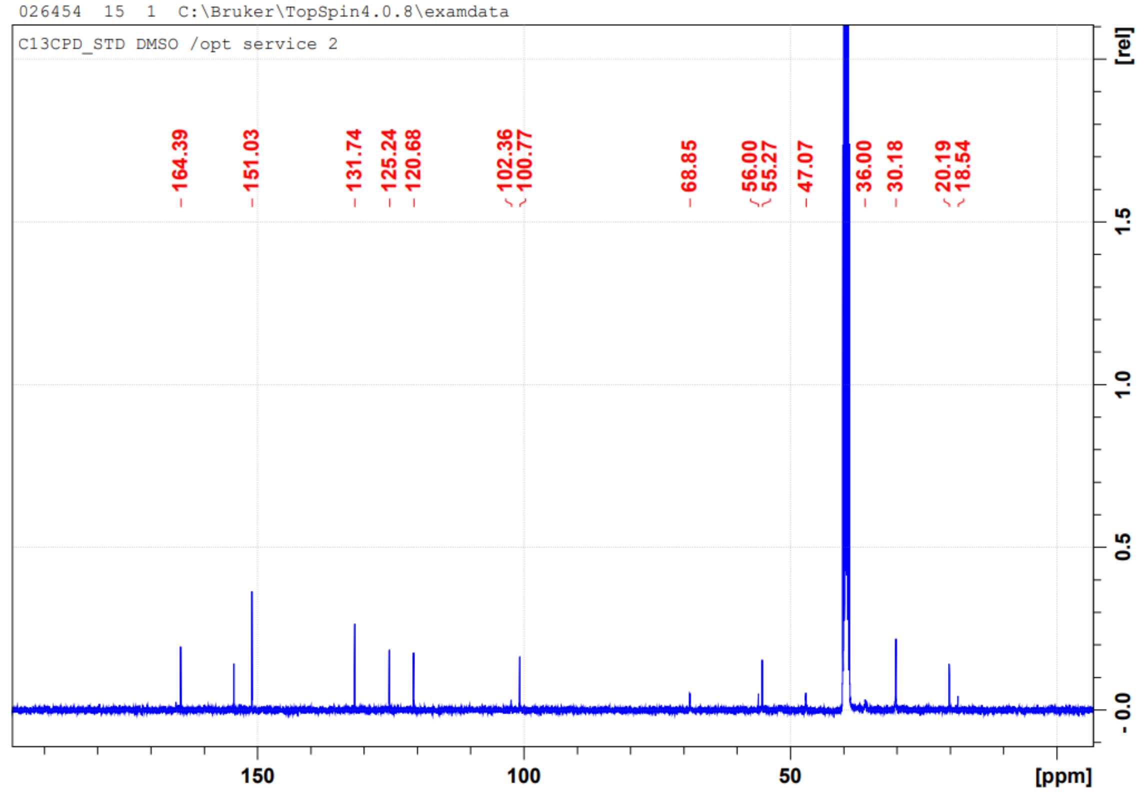

Compound 40c

^{1}H-NMR

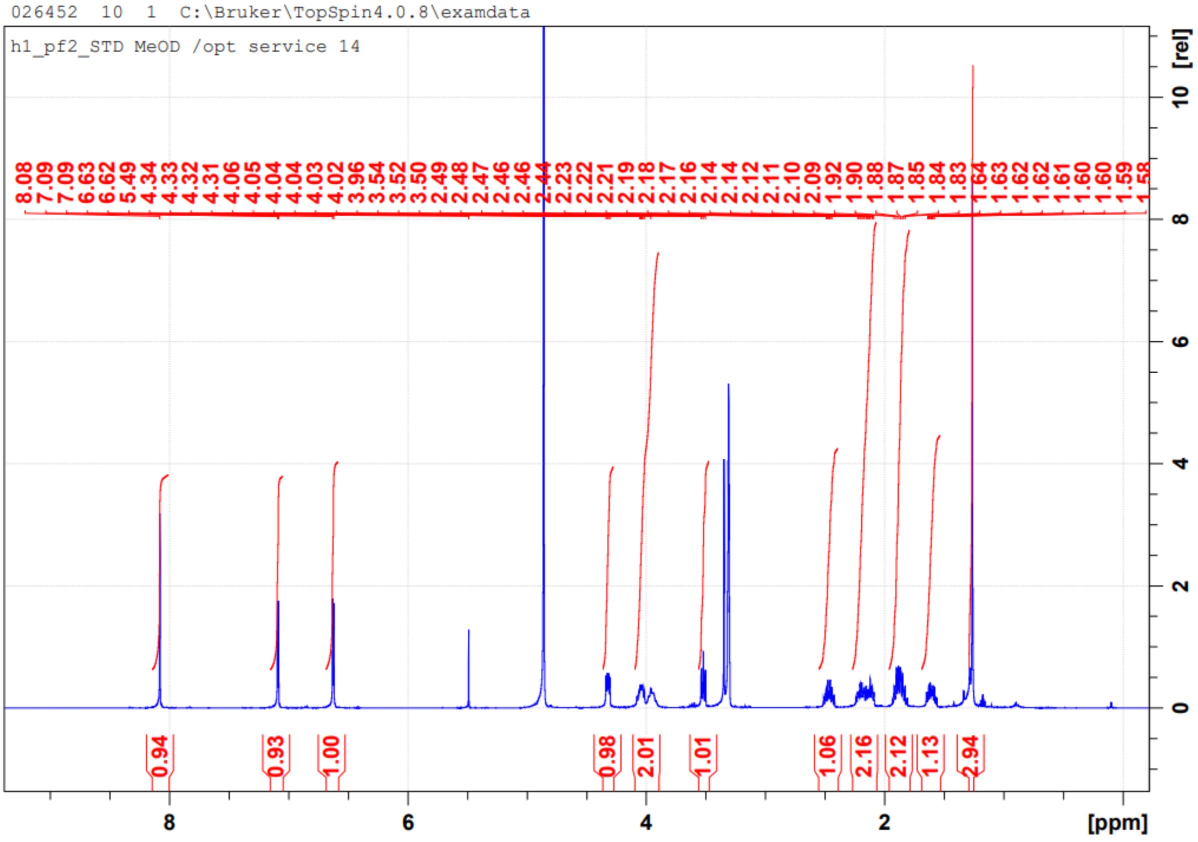

^{13}C-NMR

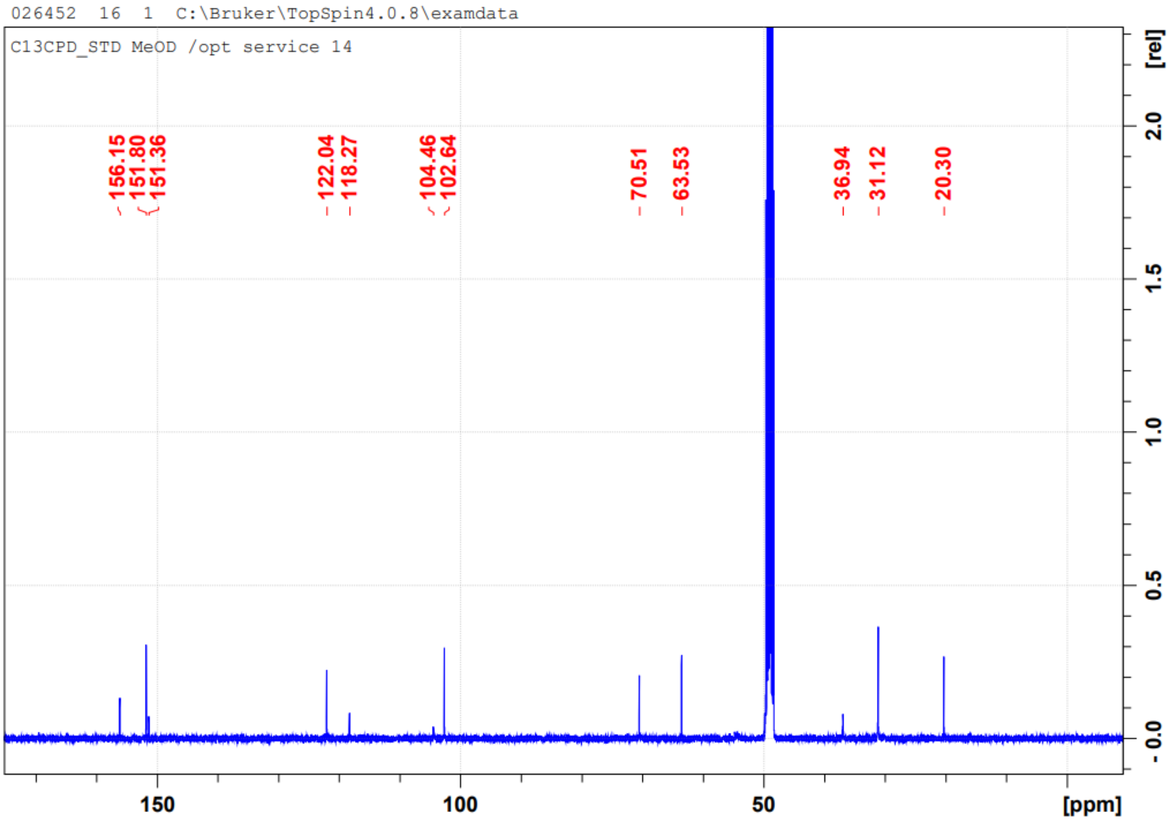

Compound 40d

^{1}H-NMR

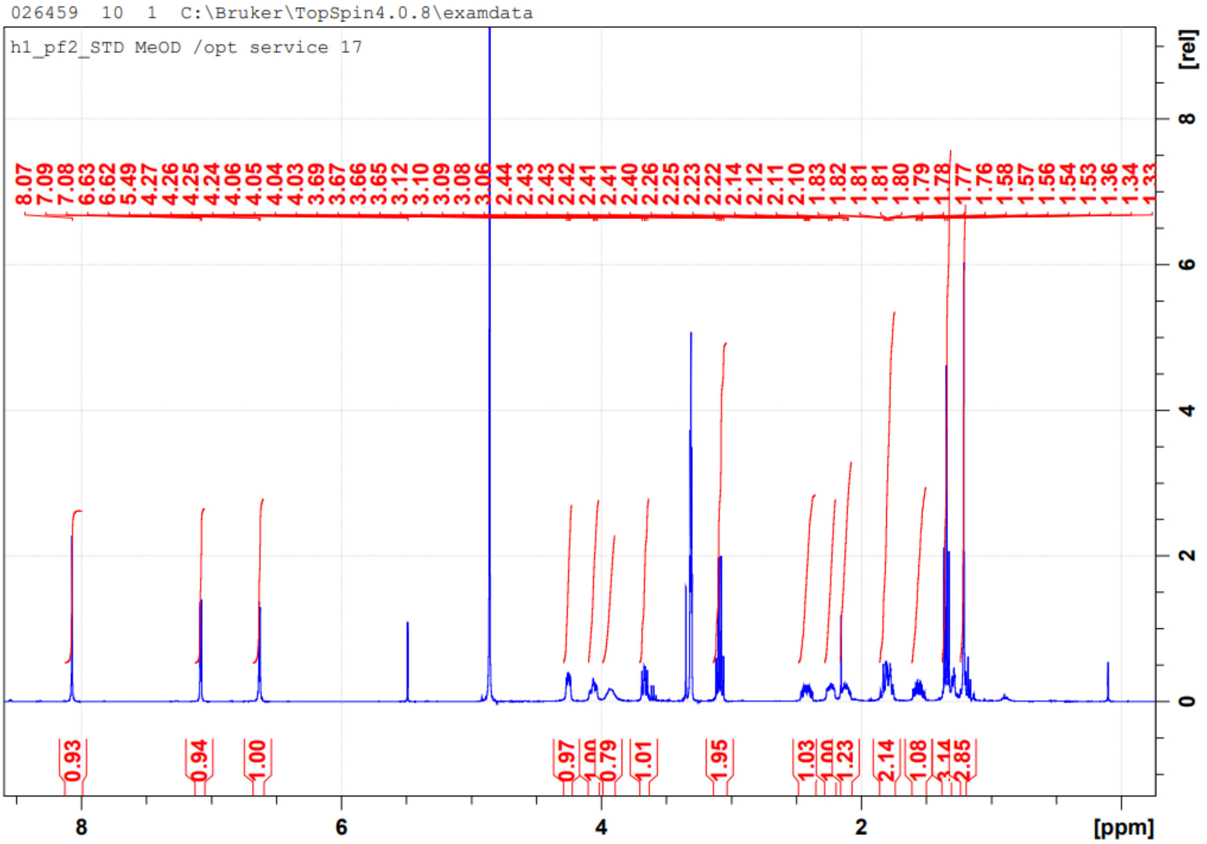

^{13}C-NMR

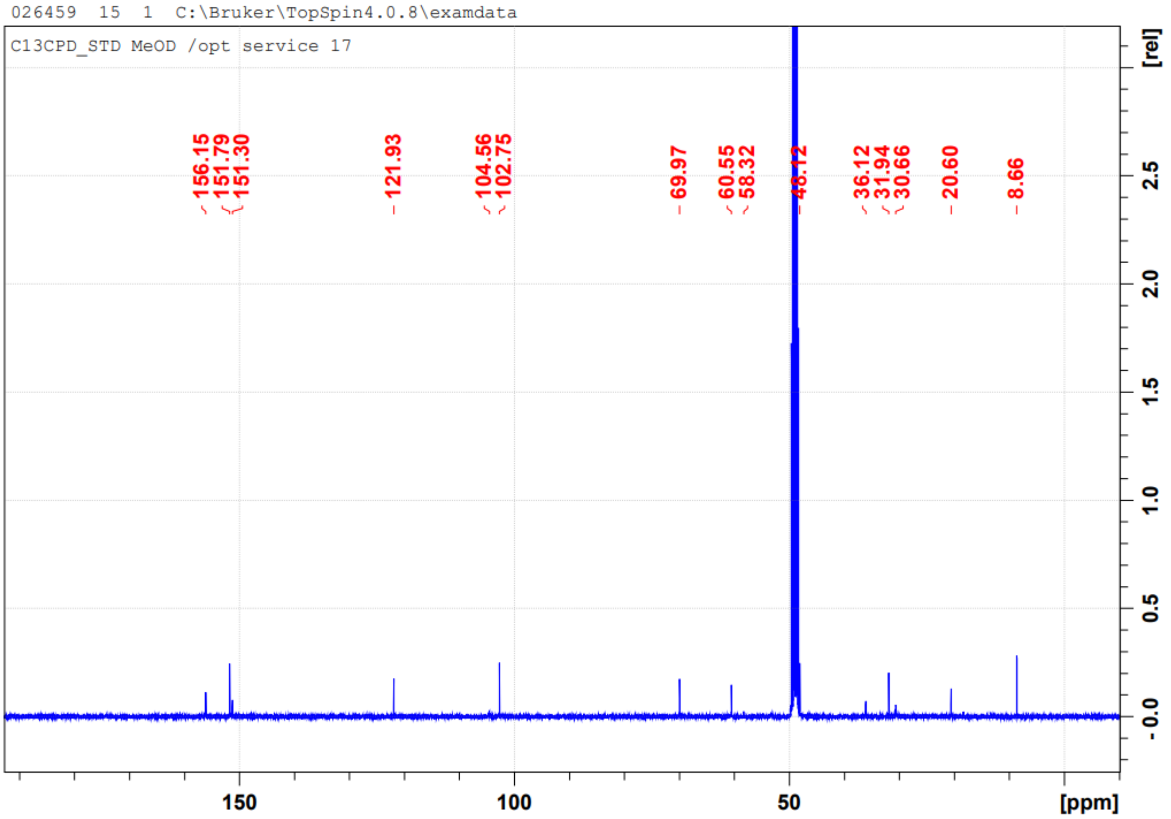

Compound 41

¹H-NMR

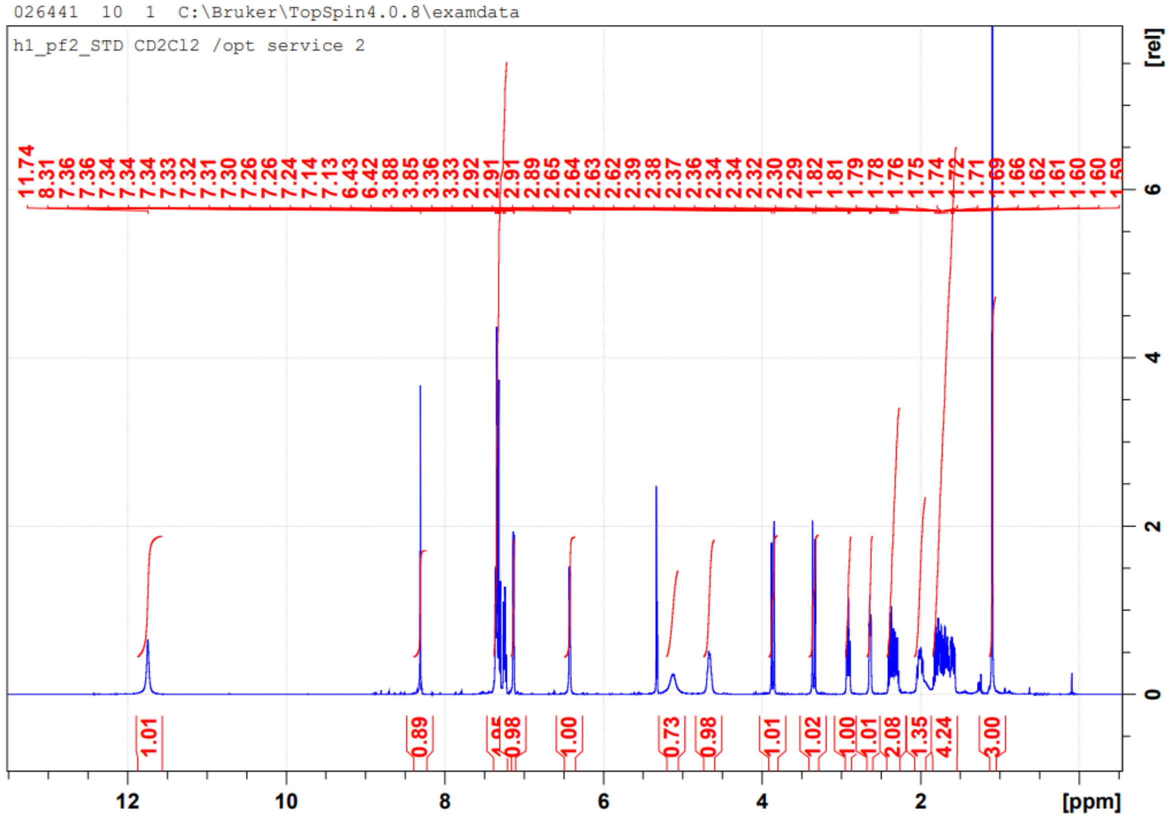

¹³C-NMR

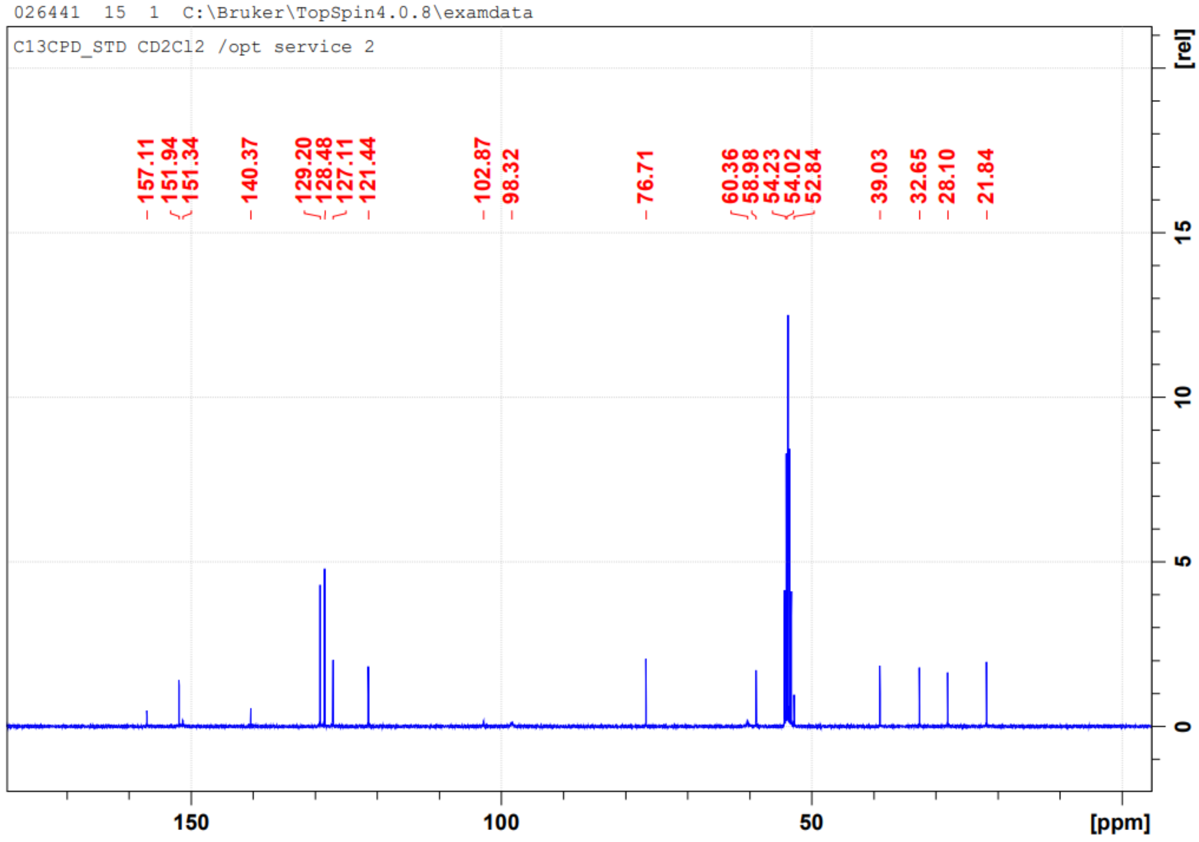

Compound 42a

^{1}H-NMR (353 K)

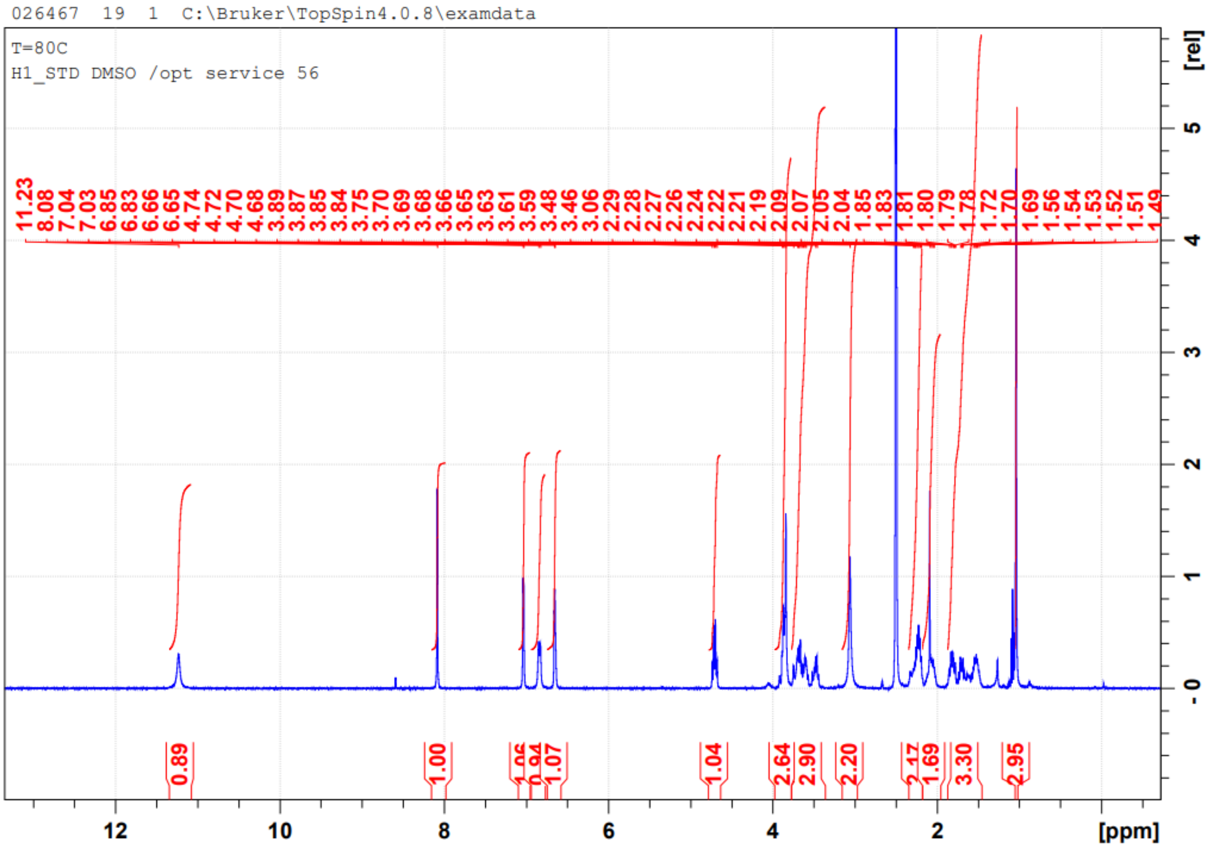

^{13}C-NMR (353 K)

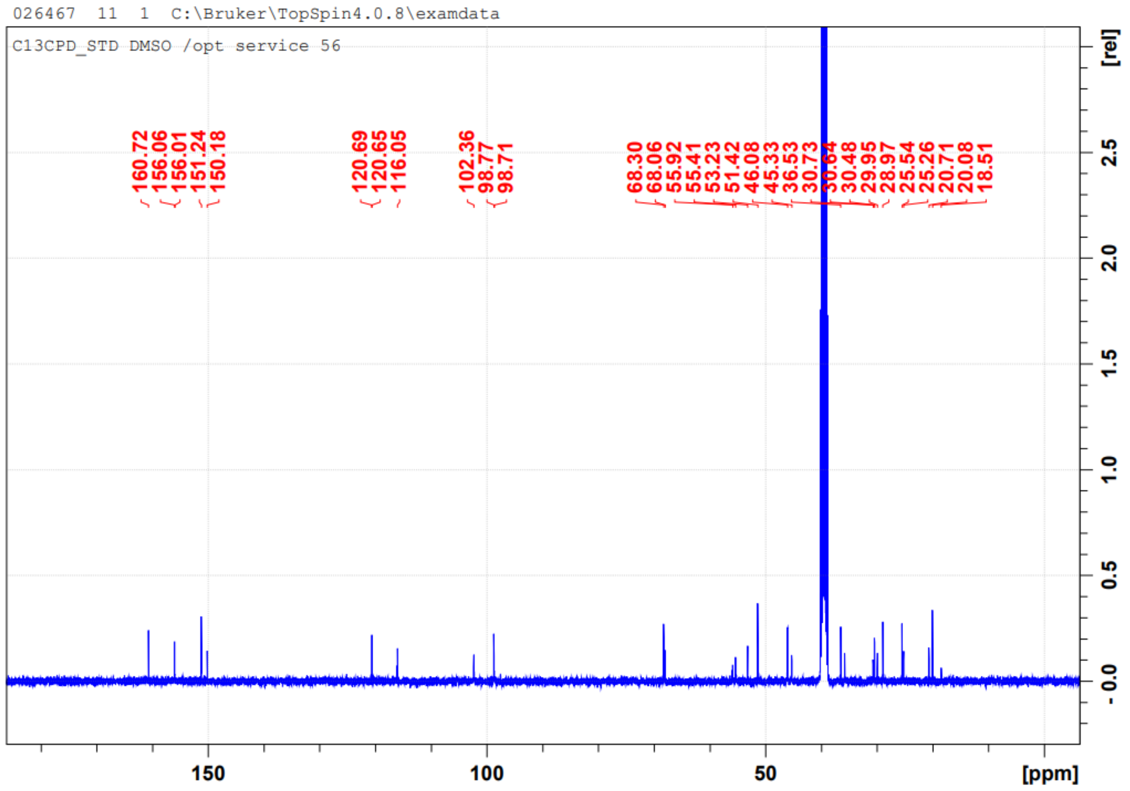

Compound 42b

^{1}H-NMR

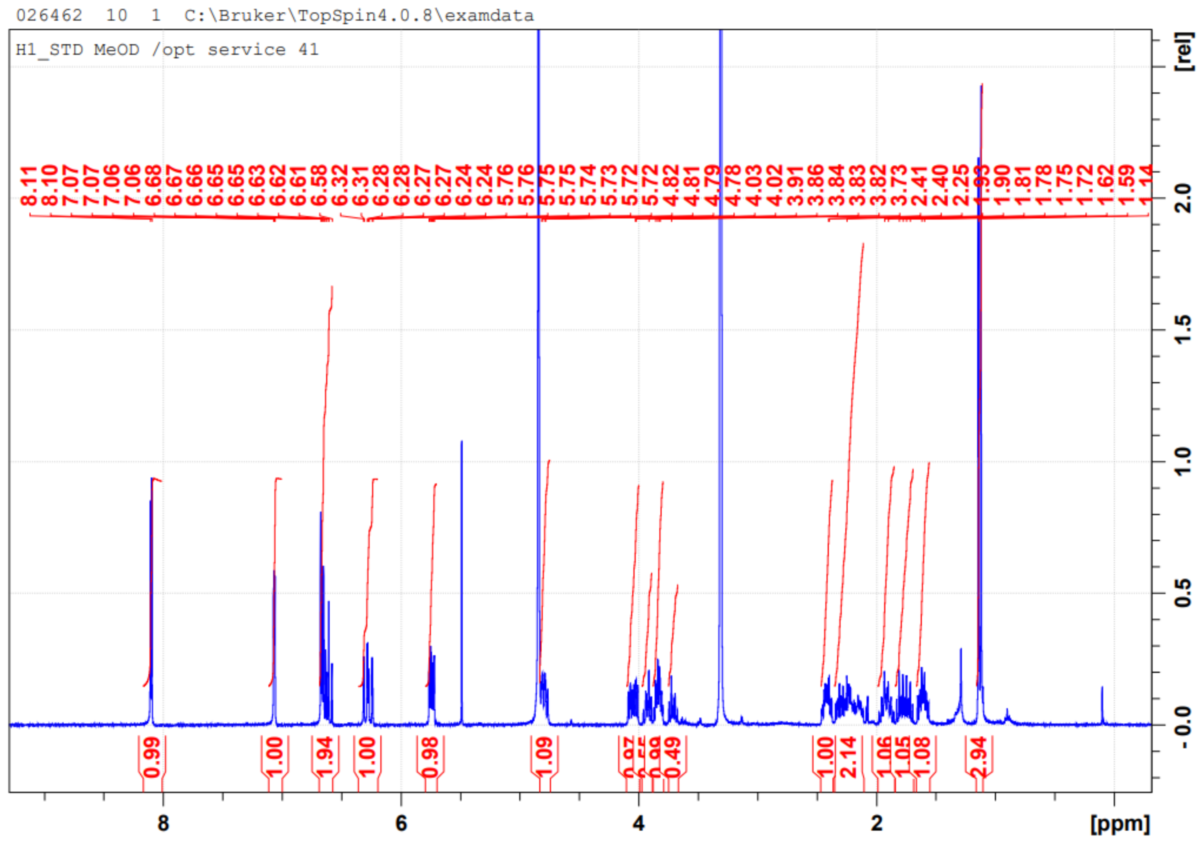

^{13}C-NMR

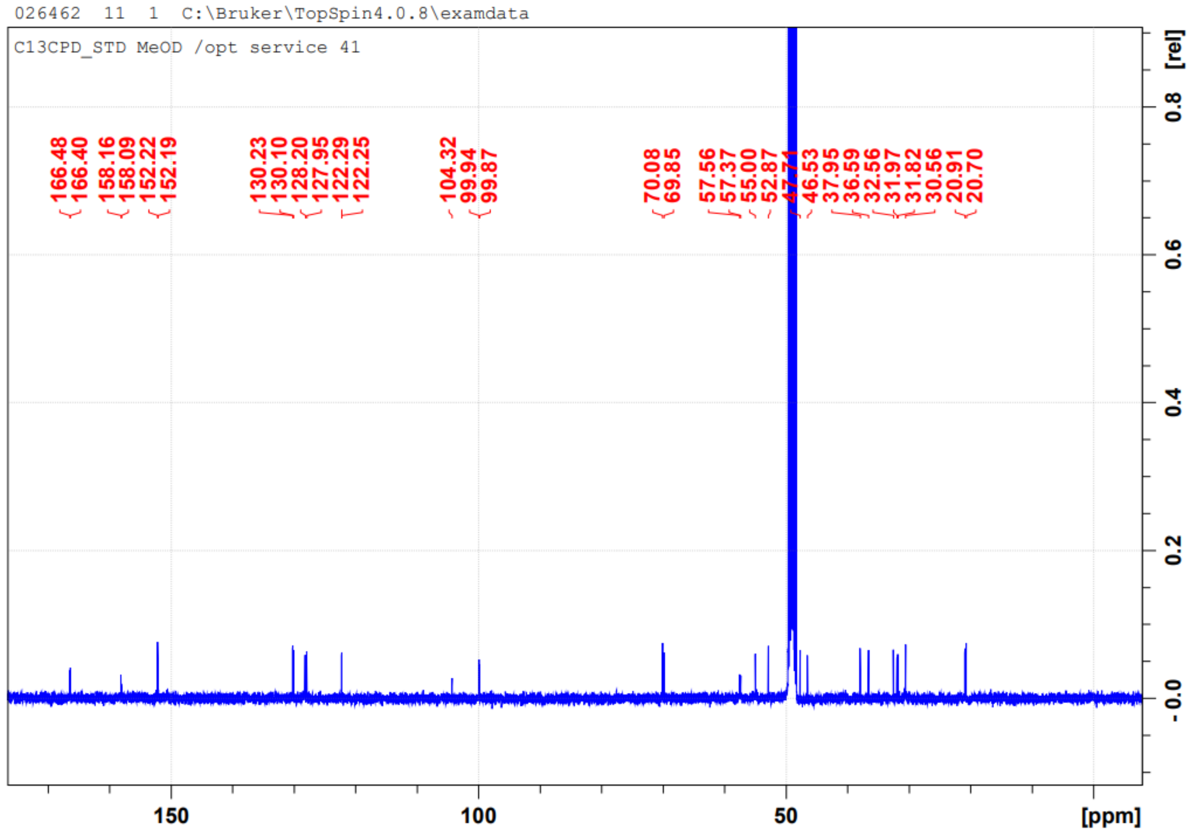

Compound 42c
^{1}H-NMR

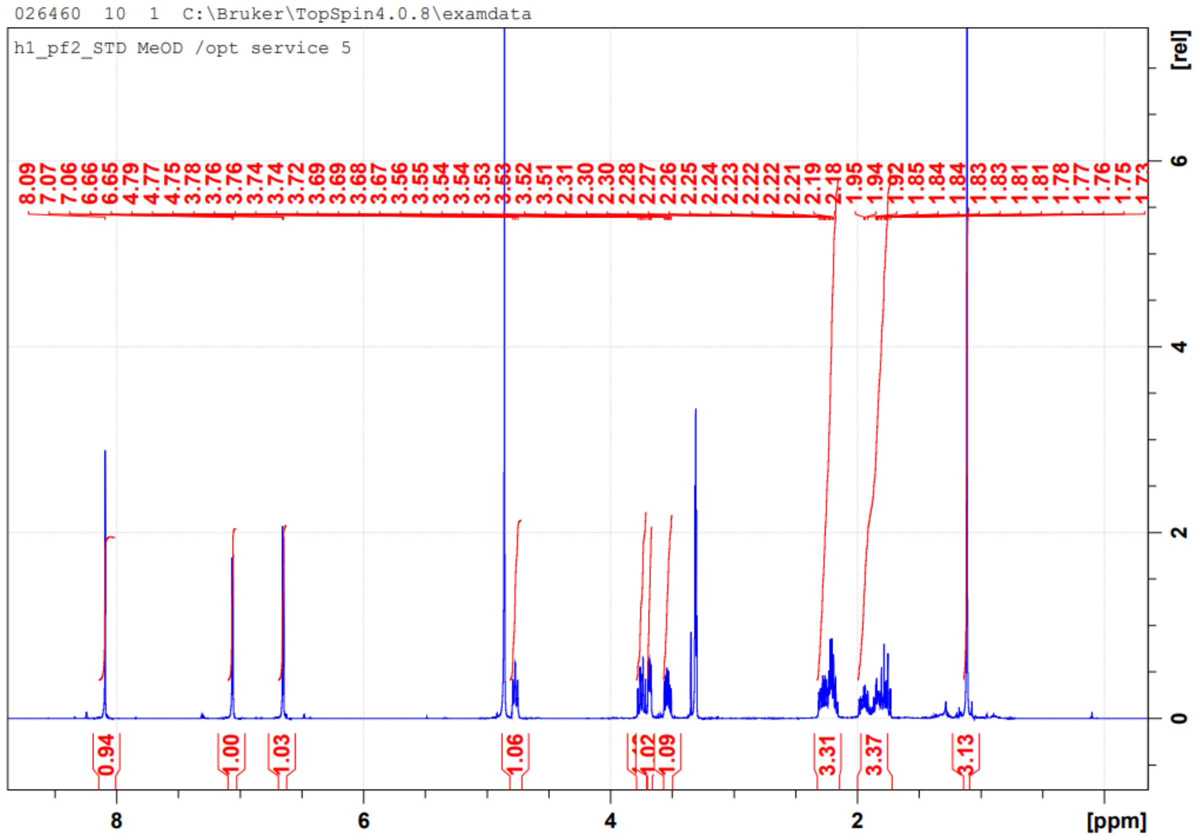

^{13}C-NMR

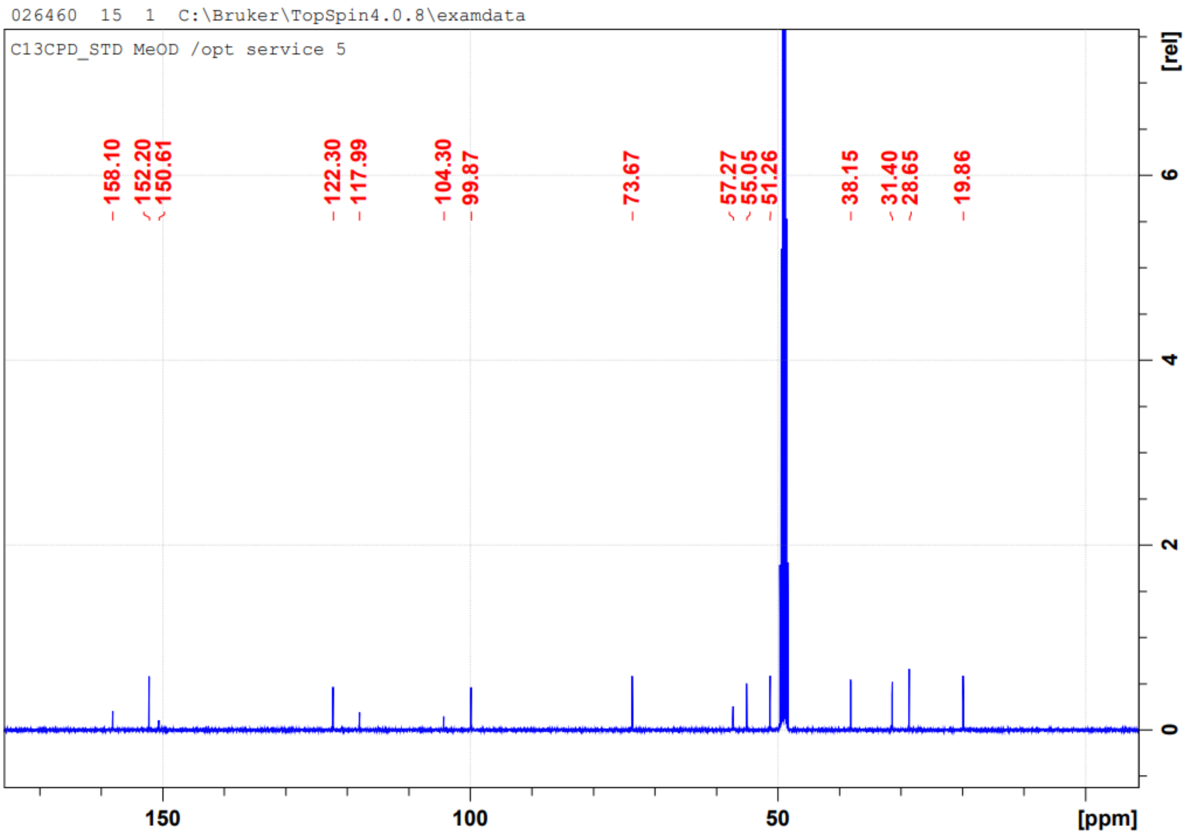

Compound 2S

^{1}H-NMR

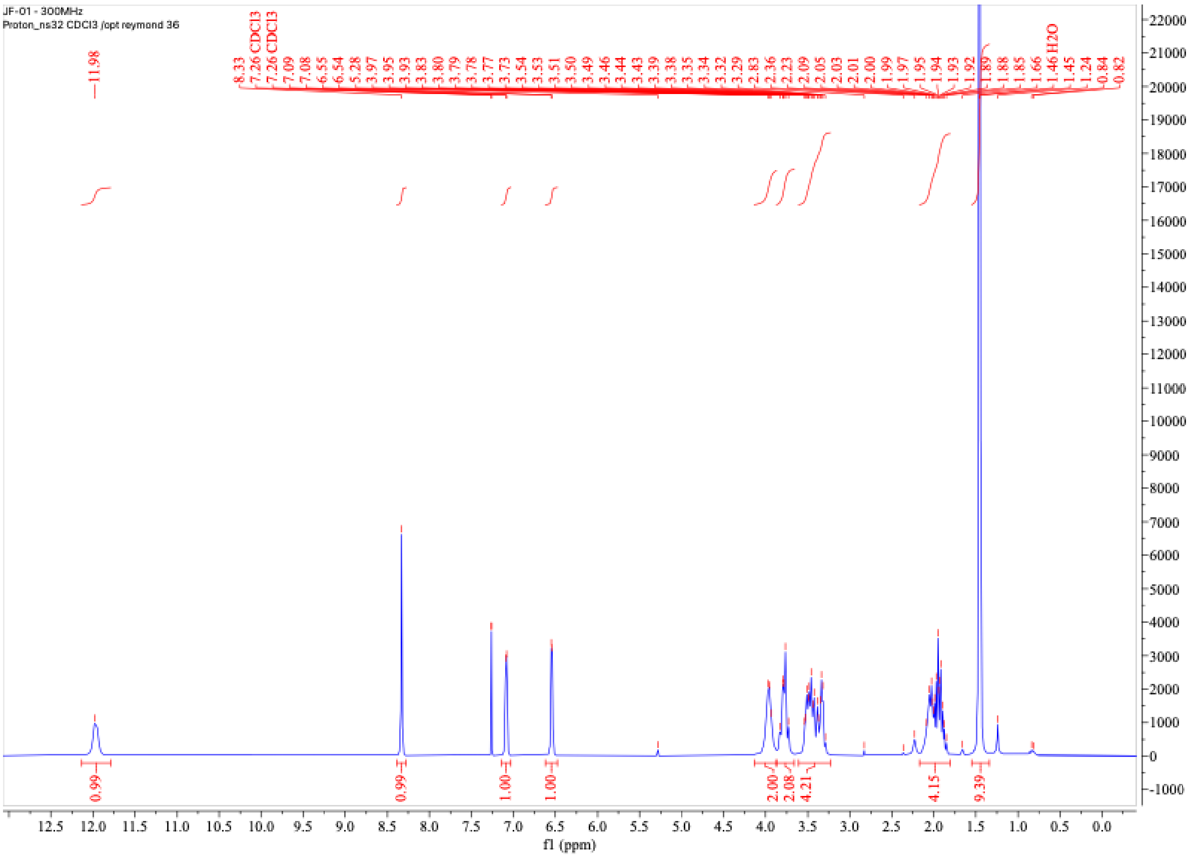

Compound 43a

^{1}H-NMR

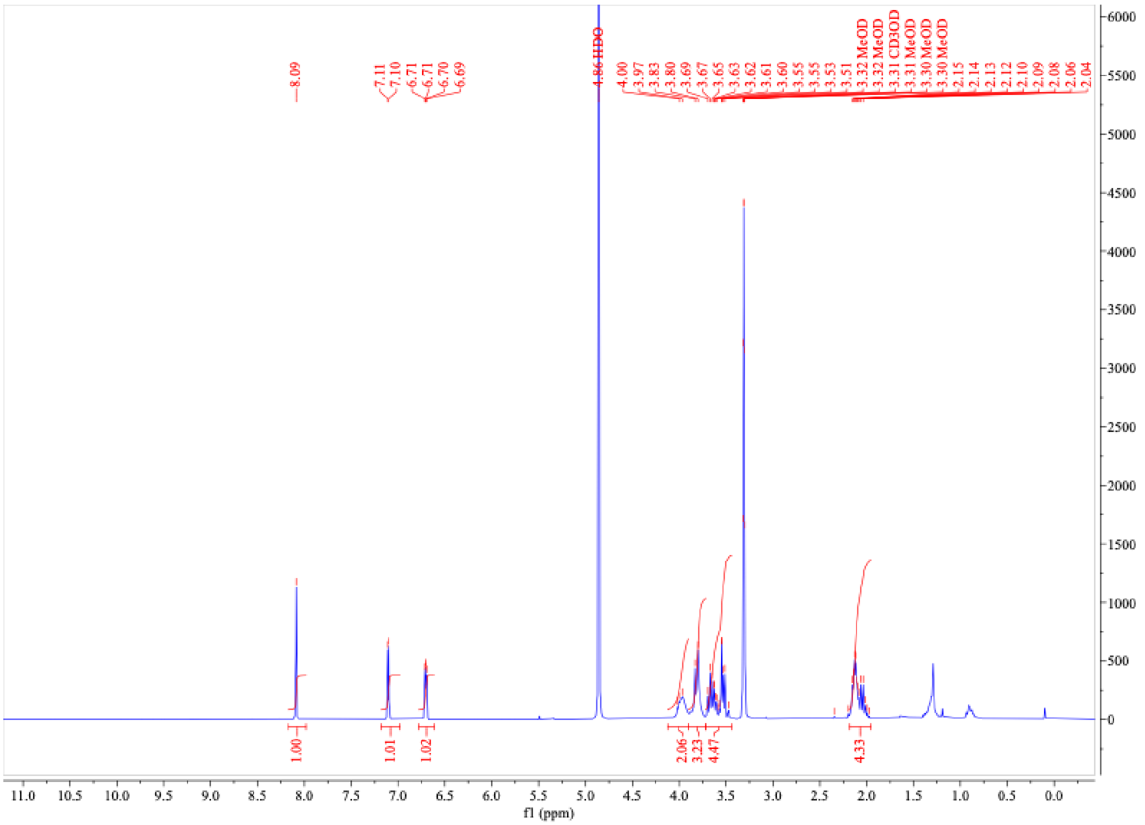

^{13}C-NMR

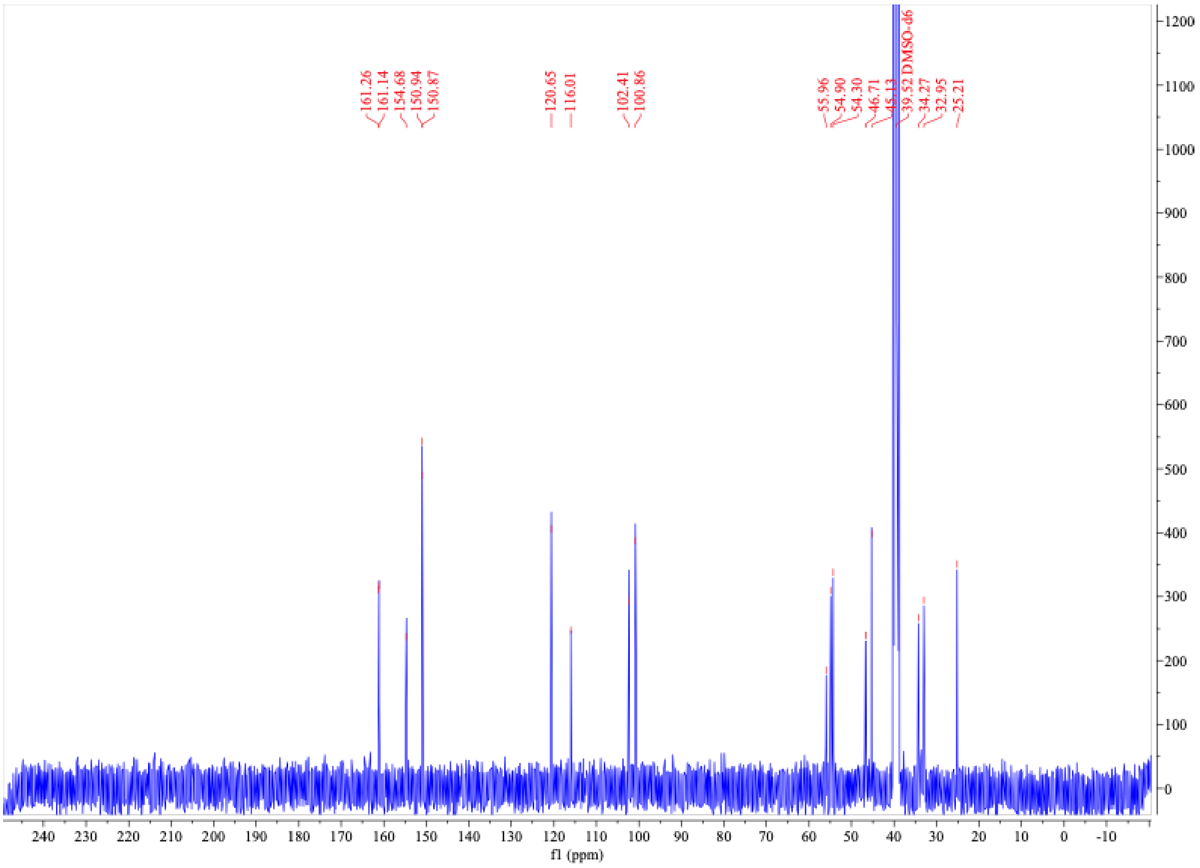

Compound 43b

^{1}H-NMR

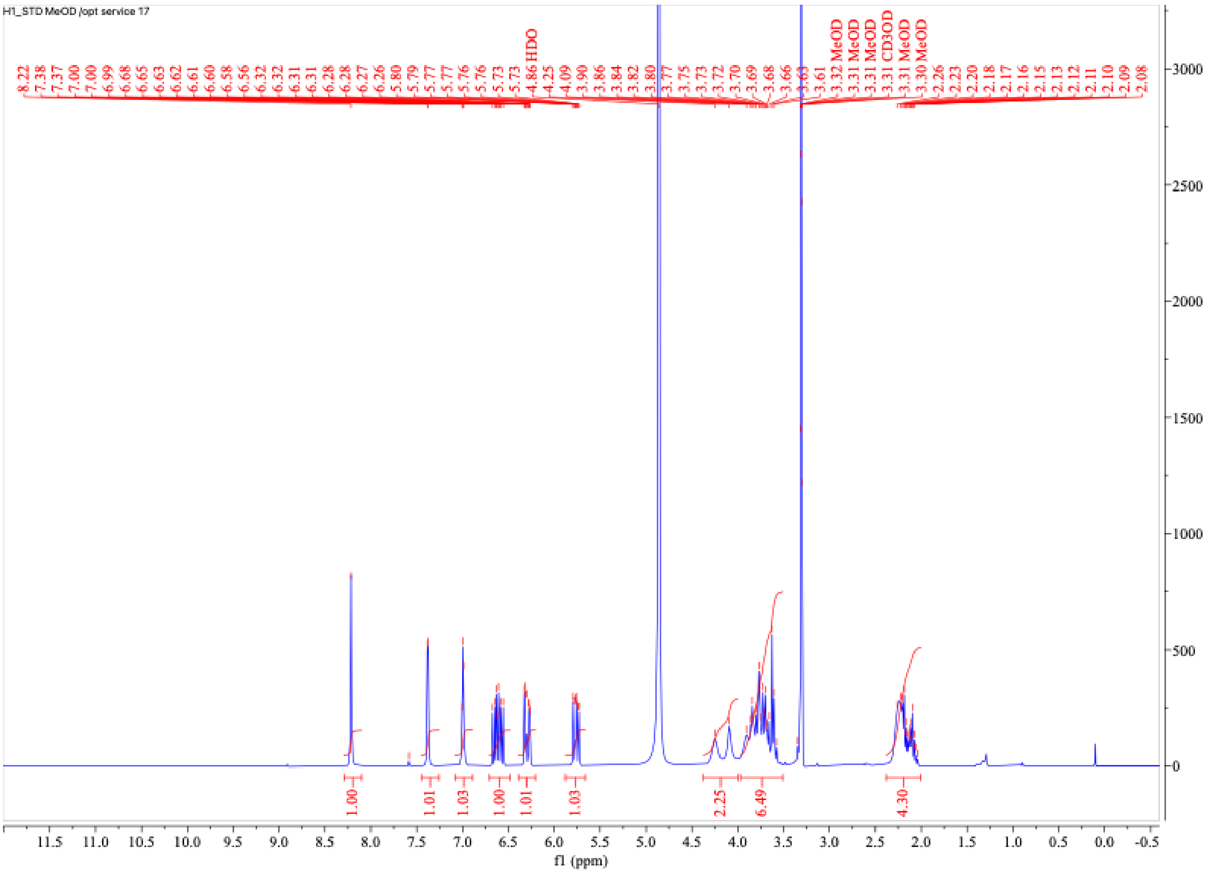

^{13}C-NMR

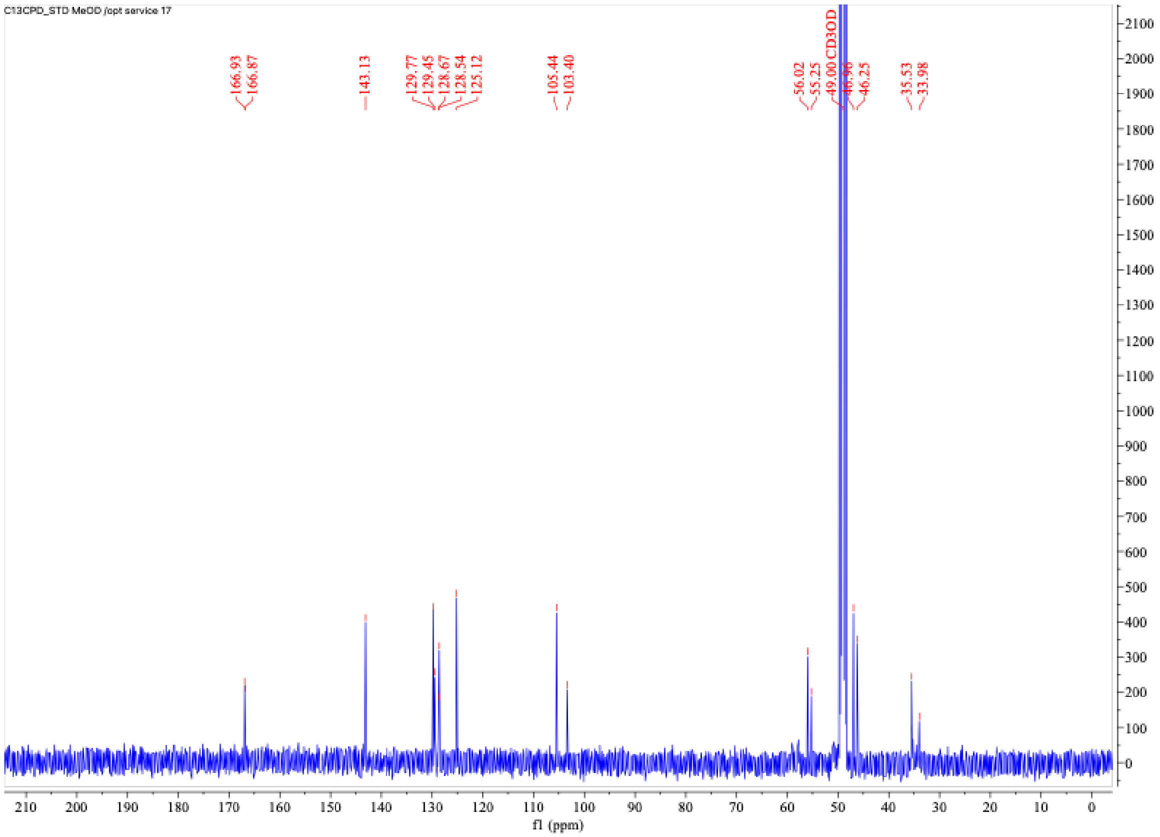

Compound 43c

^{1}H-NMR

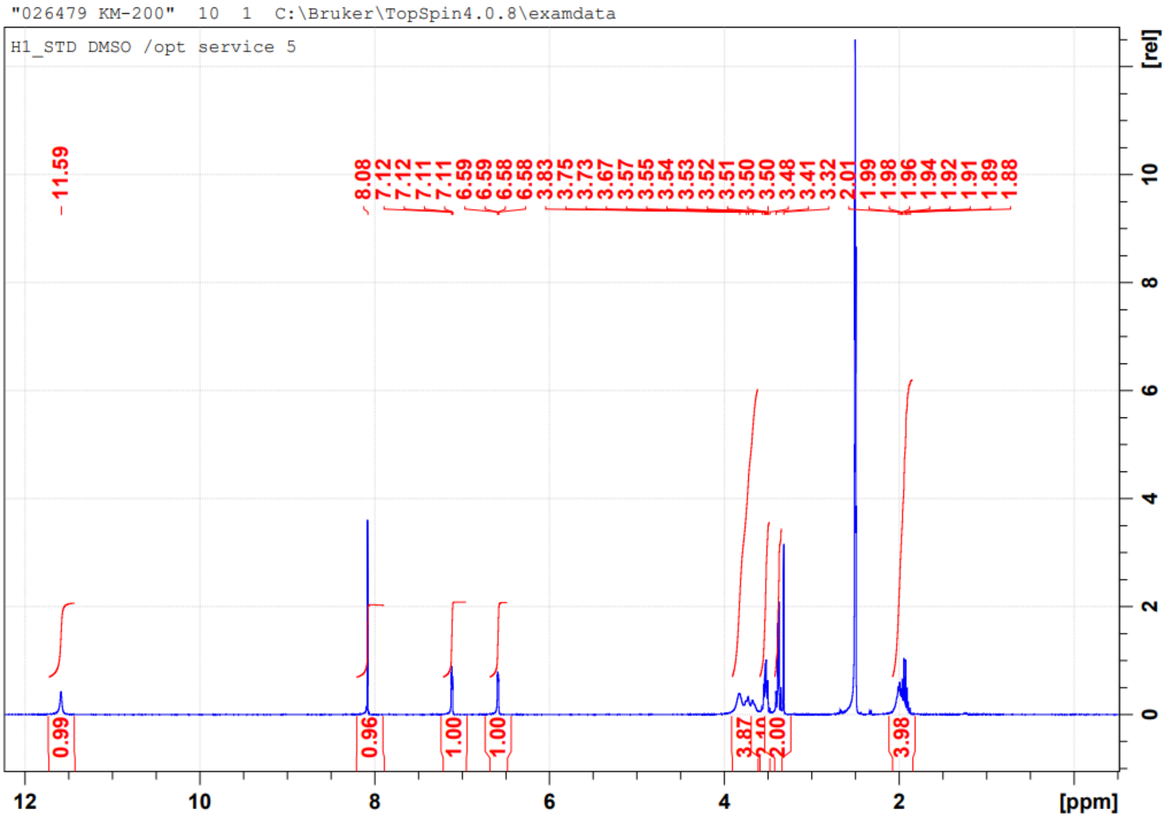

^{13}C-NMR

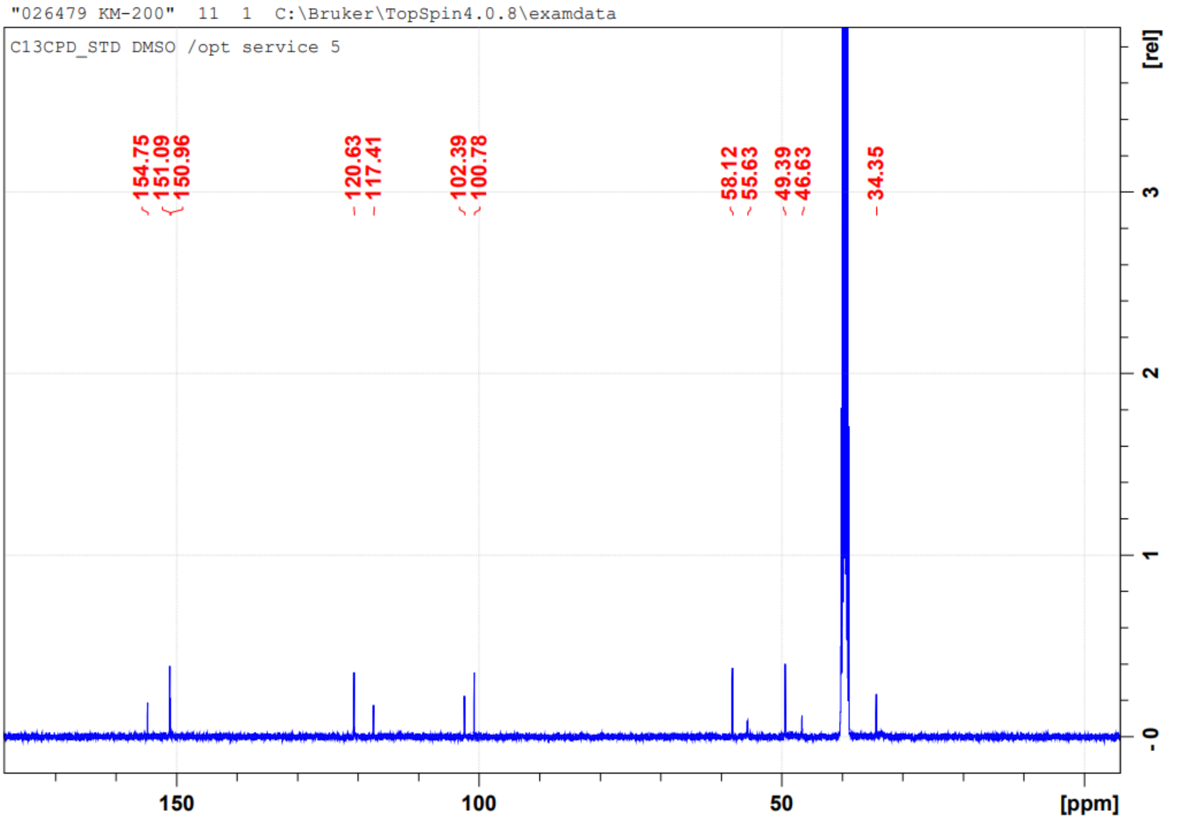

Compound 43d

^{1}H-NMR

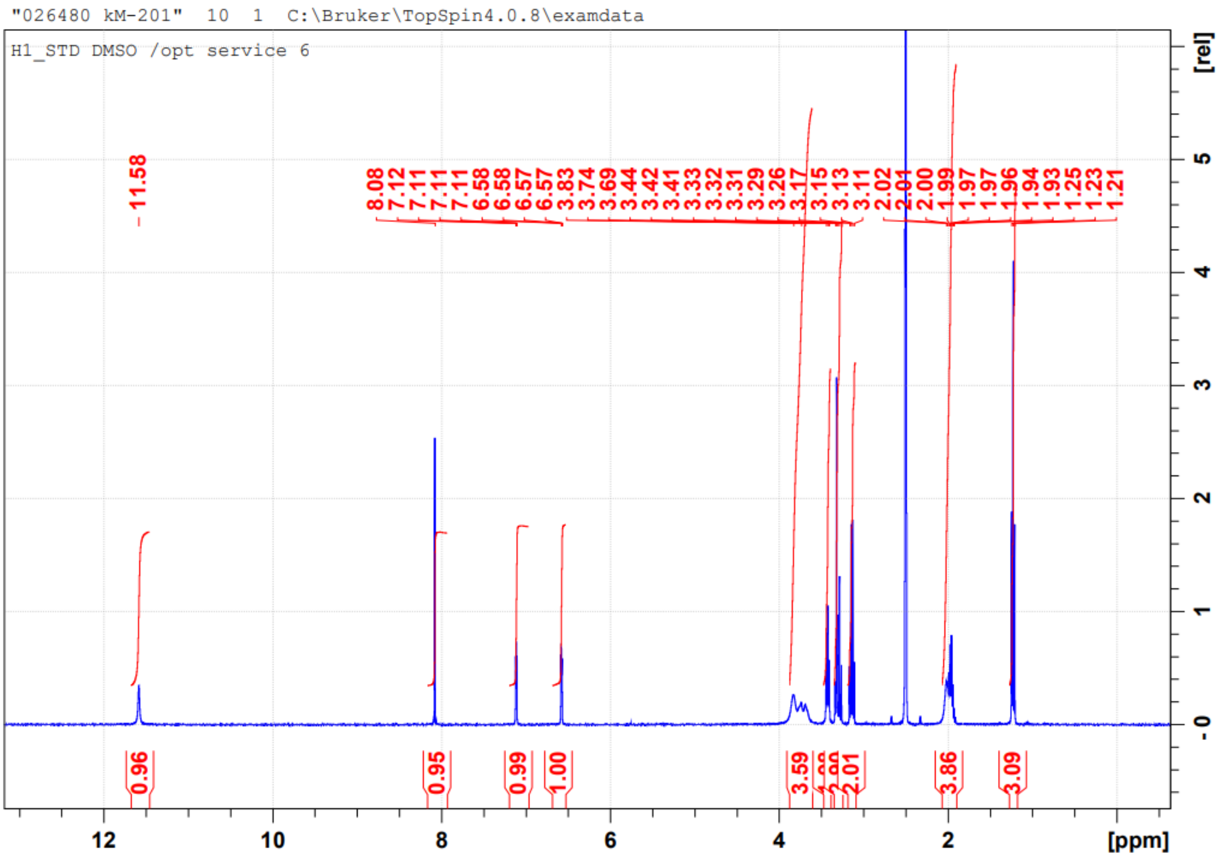

^{13}C-NMR

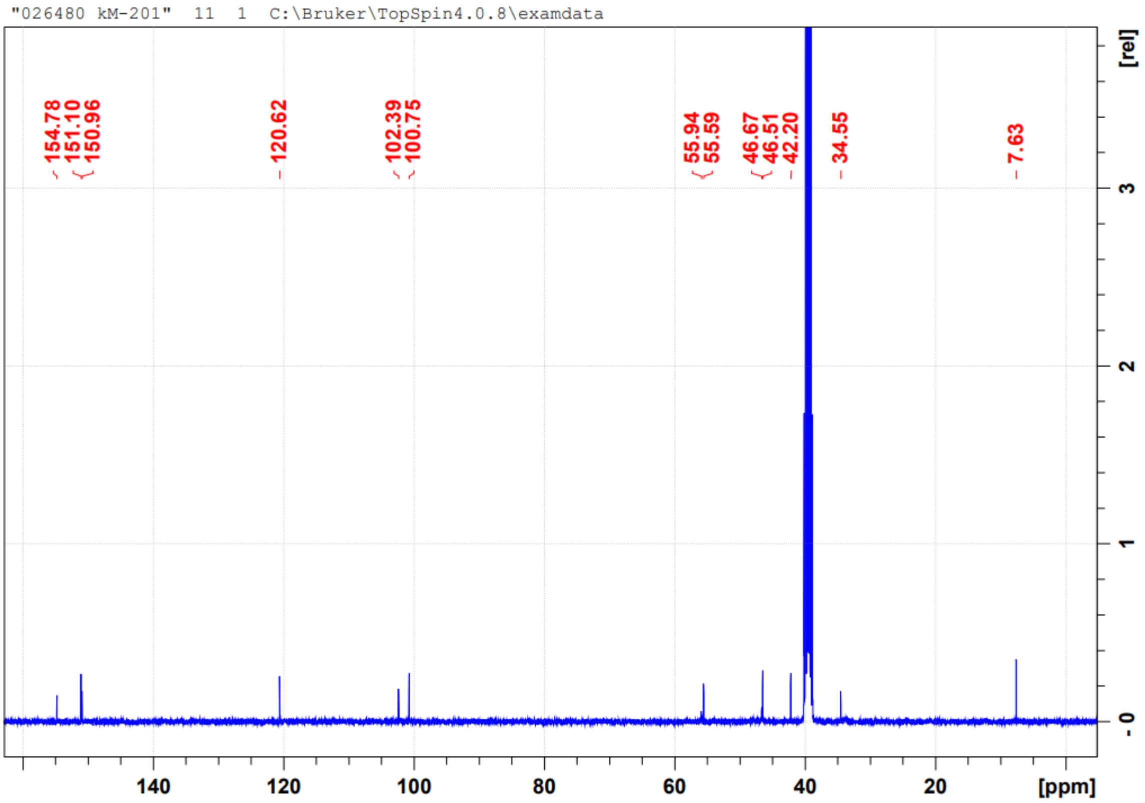

Compound (*R,S,S*)-40a

^{1}H-NMR

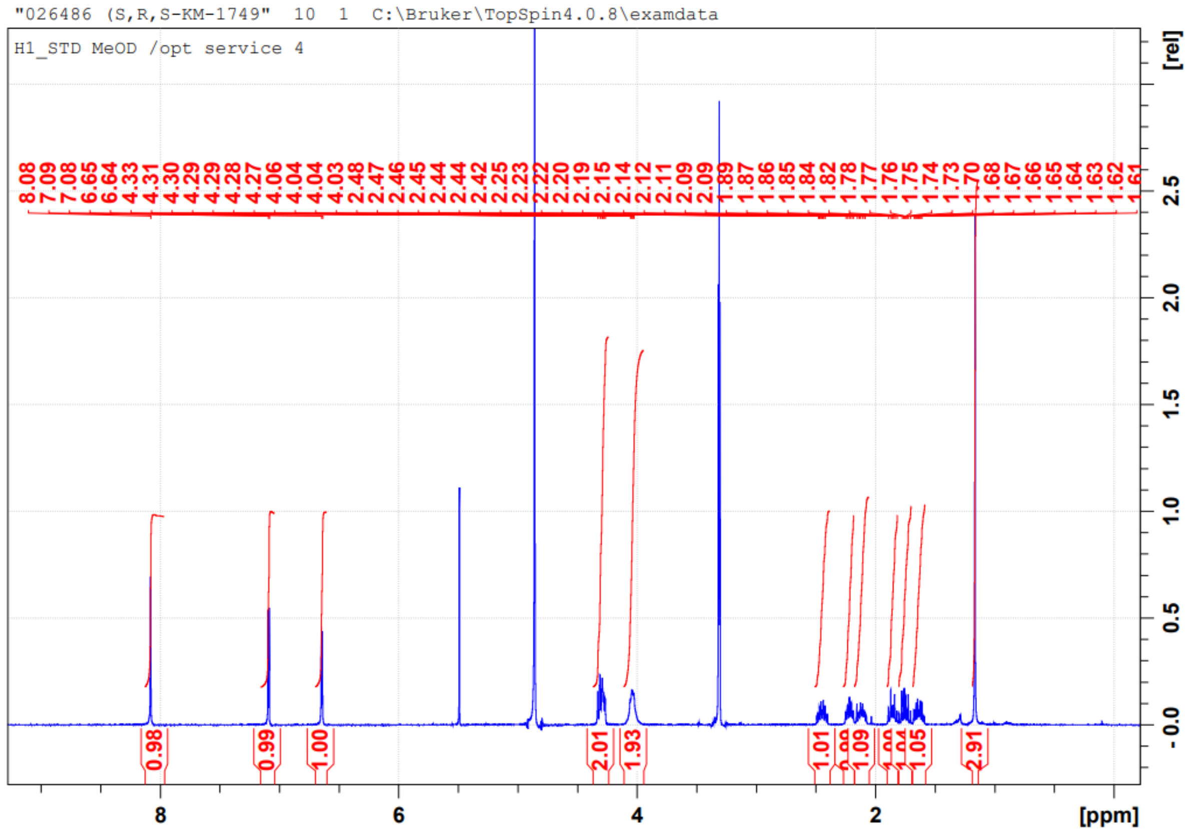

^{13}C-NMR

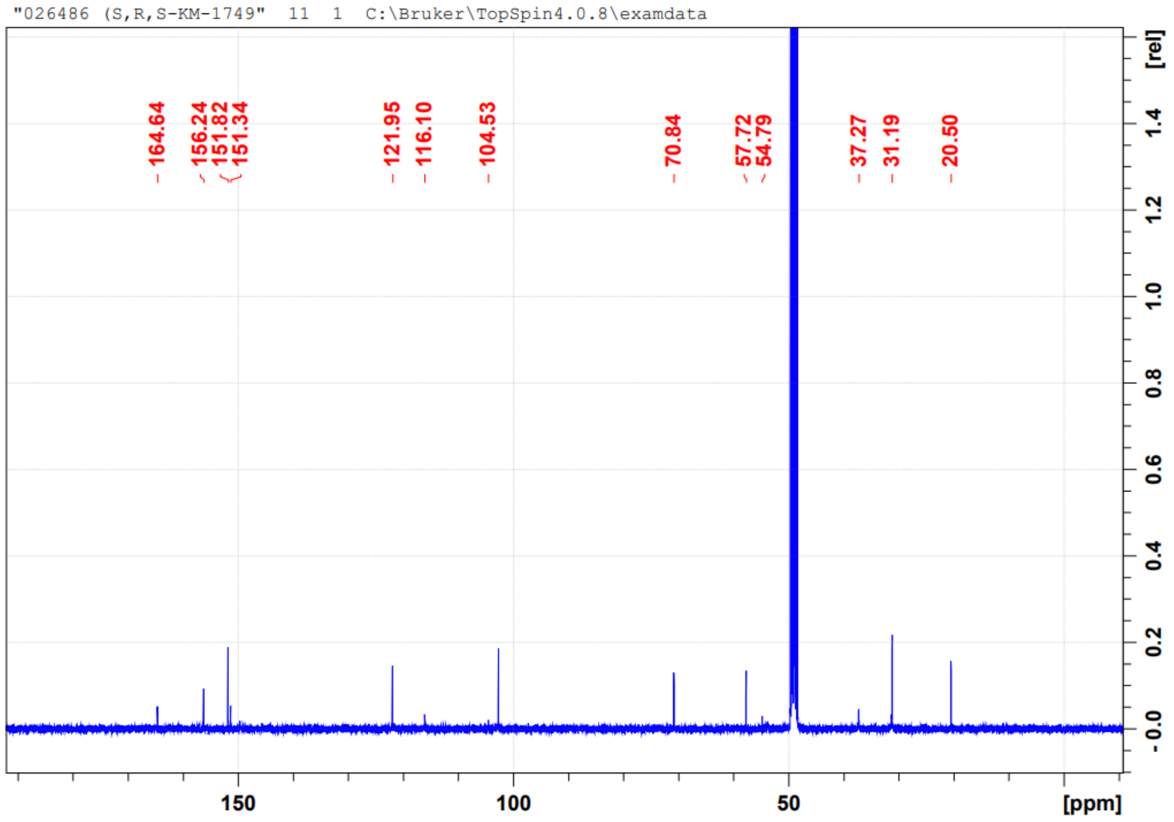

Compound (*S*,*R*,*R*)-40a

1H-NMR

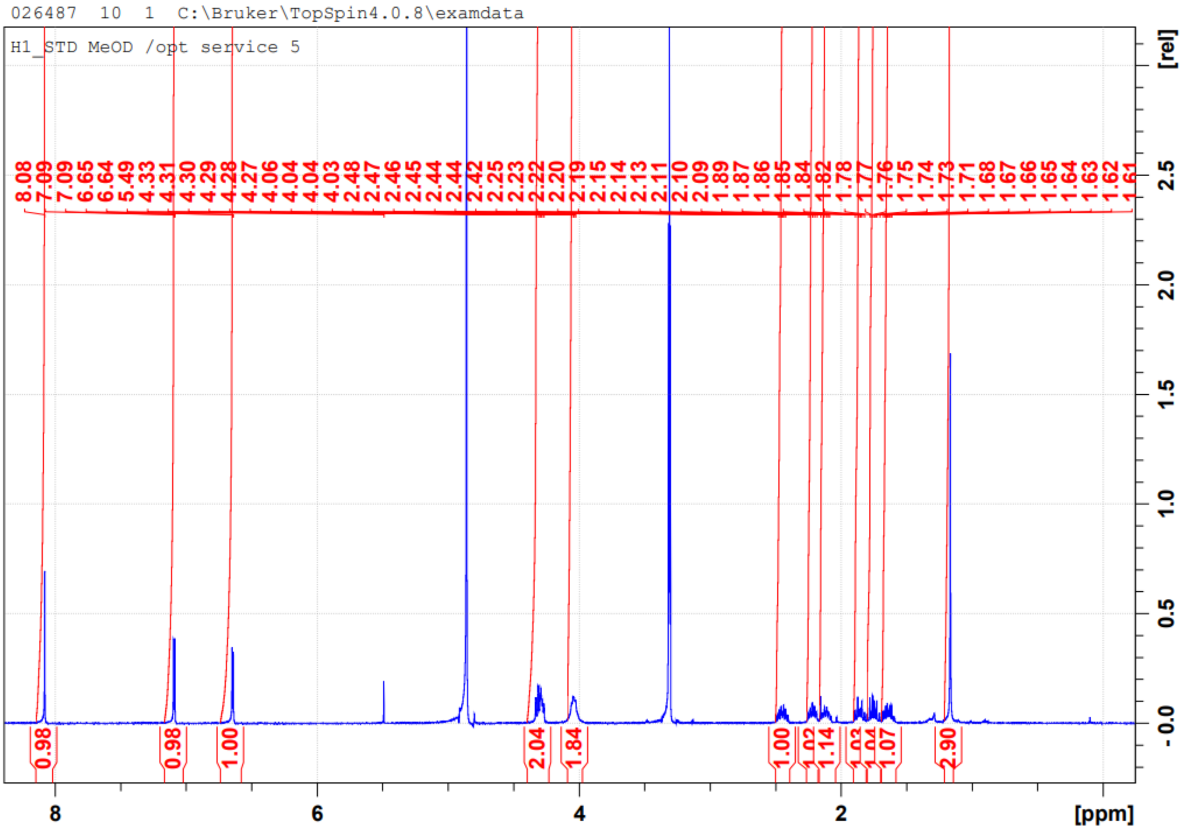

^{13}C-NMR

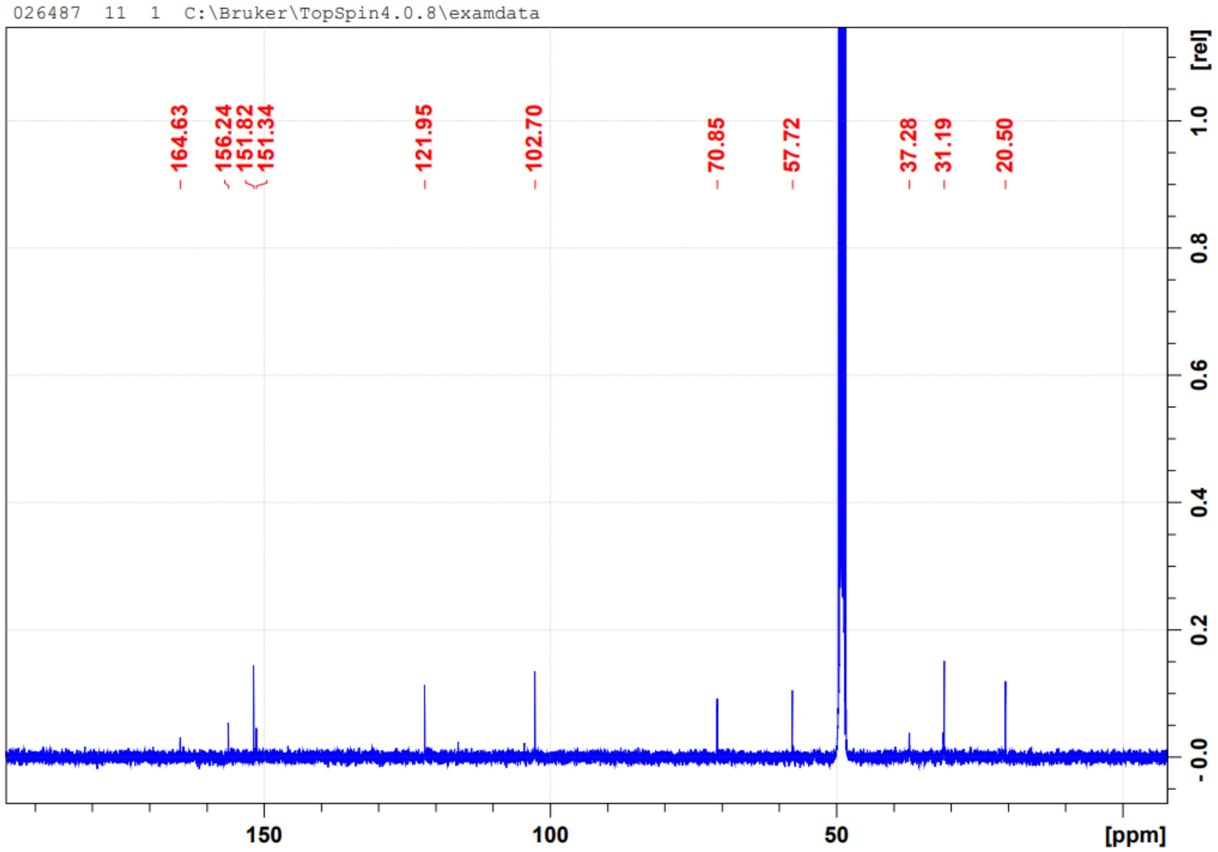

Supplement: MD-017-D5MD00921A-s001 [file MD-017-D5MD00921A-s001.pdf]
